# Supplementary material for: Direct synthesis of partially ethoxylated branched polyethylenimine from ethanolamine
Source: Nat Commun. 2024 Jul 24;15:6253. doi: 10.1038/s41467-024-50403-z (PMC11269587; doi:10.1038/s41467-024-50403-z)
Supplement: Supplementary file 1 — Supplementary Information [file 41467_2024_50403_MOESM1_ESM.pdf]

Supplementary Information for Manuscript:

Direct Synthesis of Partially Ethoxylated Branched Polyethylenimine from Ethanolamine

Claire N. Brodie, Alister S. Goodfellow, Matthew J. Andrews, Aniekan E. Owen, Michael Bühl* and Amit Kumar*

EaStCHEM, School of Chemistry, University of St Andrews, North Haugh, St Andrews, KY16 9ST, UK.

Email: ak366@st-andrews.ac.uk; mb105@st-andrews.ac.uk

Table of Contents

Contents

1. Experimental Details 4

1.1 General Considerations 4

1.2 Procedure for the Polymerisation of Ethanolamine 6

1.3 Ethanolamine Polymerisation Reaction Optimisation 7

1.3.1 IR Spectra 12

1.3.2 GPC Data 16

1.3.3 NMR Spectra 20

1.3.4 TGA data 37

1.3.5 DSC Data 43

1.3.6 HRMS (EI) spectra 49

1.3.7 Powder XRD 50

1.4 Ethanolamine Polymerisation Reaction Progression Monitoring 51

1.4.1 IR Spectra 53

1.4.2 GPC Data 54

1.4.3 NMR Spectra 55

1.5 Catalyst Recyclability 56

1.5.1 Catalyst recyclability without additional base 56

1.5.2 Catalyst recyclability with additional base 61

1.6 Mechanistic Studies 64

1.6.1 Stochiometric experiments 64

1.6.2 In situ NMR spectra 65

1.6.3 Polymerisation of N-(2-Hydroxyethyl)ethylenediamine 66

1.6.4 Hydrogenation of N-benzyl-1-phenylmethanimine 69

1.6.5 Hydrogenation of 1,5-cyclooctadiene 71

1.6.6 Hydrogenation of 1-(1-butenyl)pyrrolidine 73

1.6.7 Headspace gas analysis of the dehydrogenative coupling of ethanolamine 74

1.6.8 Polymerisation under D_2 gas (1.2 bar) 74

1.6.9 Polymerisation using pre-formed catalyst 76

1.7 Polymer Degradation 80

1.8 CHN/O Analyses 81

1.9 Scale Up Procedure 82

1.10 Quantatifying Primary Amines (quantitative $^{13}C\{^1H\}$ NMR and UV-Vis experiments) 86

1.10.1 Calibration using N-(2-hydroxyethyl)ethylenediamine: 88

1.10.2 Quantitative $^{13}C\{^{1}H\}$ NMR spectra 89
1.11 Substrate Scope 91
1.11.1 GC-MS Spectra 92
1.11.2 NMR Spectra 98
1.12 Expanded Substrate Scope 100
2. Computational Details 104
2.1 General Considerations 104
2.2 Driving forces 105
2.3 Barriers for (de)hydrogenation steps 107
2.4 Computational Raw Data 110
3. References 134

1. Experimental Details

1.1 General Considerations

All manipulations, unless otherwise stated, were performed under an argon atmosphere using standard Schlenk line and glove-box techniques. Glassware was oven-dried at 130 °C overnight and flamed under vacuum prior to use. THF and toluene were dried using a Grubbs-type solvent purification system (Innovative Technologies SPS) equipped with a degasser. Pre-catalyst **Mn-1**[S1] was prepared in accordance with the literature procedure. KO^tBu (anhydrous), NaO^tBu (anhydrous) and K_2CO_3 (anhydrous) were stored at 80 °C and dried before use. D_2O, anisole, alcohol-amines and iPr-PN^HP (10 wt% solution in THF) were purchased from Sigma Aldrich, Merck or Strem Chemicals respectively and used as received. Toluene-d_8 was purchased from Merck, dried over CaH_2 and degassed before use.

NMR spectra were recorded on a Bruker AVIII-HD 500 MHz NMR spectrometer at 298 K unless otherwise specified. Residual protio solvent was used as reference for ^1H spectra in deuterated solvent samples. All chemical shifts (δ) are quoted in ppm and coupling constants (*J*) in Hz. ESI-MS spectra were collected as solutions in acetonitrile using a Micromass LCT spectrometer.

Gel permeation chromatography (GPC) was performed on an Agilent 1260 InfinityLab II GPC fitted with a refractive index (RI) detector (35 °C). The single (plus guard column) Agilent PolarGel column setup was contained within an oven (35 °C). H_2O was used as the eluent at a flow rate of 1.0 mL min^{-1}. Samples were dissolved in the eluent (2.0 mg mL^{-1}), filtered (0.2 μm pore size) and run immediately. The calibration was conducted using a series of monodisperse poly(ethylene glycol) (M_n = 194 – 20,000 g mol^{-1}) and poly(ethylene oxide) (M_n = 30,000 – 50,000 g mol^{-1}) standards obtained from Agilent Technologies.

Infrared spectra (ATR-FTIR) were collected using a Shimadzu IRAffinity-1. EI-MS spectra were collected as solutions in acetonitrile using a Micromass LCT spectrometer.

Elemental analyses were performed by Orla McCullough at London Metropolitan University or by Joe Casillo at the University of Edinburgh.

Powder-XRD data were obtained through loading a sample of **PEI-1** into a 0.3 mm diameter glass capillary and data were collected on a STOE STADIP diffractometer operating in Debye–Scherrer geometry using Mo $K\alpha_1$ (λ = 0.71075 Å) radiation and a Mythen 2 K detector. Data were collected in the range of 2 – 43° for 12 hours.

TGA was performed using an Stanton Redcroft STA-780 Series Thermal Analyser between 25 – 600 °C or an STA449 DSC 217C between 30 – 600 °C at a heating rate of 10 °C/min under a flow of nitrogen gas (25 mL/min). Decomposition temperature (T_d) is defined as 5% mass loss after removal of residual volatiles *e.g*. solvents, ethanolamine.

DSC analyses were performed using a Netzsch DSC204 between –80 – 600°C at a heating rate of 10 °C/min under a flow of nitrogen gas (20 mL/min) after an initial heat/cool cycle (25 – 120 °C at 10 °C/min with a 20 minute isothermal at 120 °C) to remove the thermal history of the sample.

TGA-MS was performed using a Netzsch STA 449F5 Jupiter between 35 – 300°C at a heating rate of 10 °C/min under a flow of argon gas (20 mL/min).

GC-MS spectra were collected as solutions in HPLC grade DCM using an Agilent 8860 GC system fitted with an agilent 30m HP-5 column, coupled to an Agilent 5977B EI instrument.

Headspace analysis was performed using an Agilent GC8860 with TCD. Gas separation is performed using dual columns (Agilent porous Polymer and Agilent Mol sieve). Gas sample (2.5 mL) is introduced to the columns using a gas tight syringe through a sample loop (0.25 mL) and run as follows: 5 minute isothermal at 60 °C, ramp to 150 °C (20 °C/min) followed by an isothermal at 150 °C for 5.5 minutes.

1.2 Procedure for the Polymerisation of Ethanolamine

General procedure: A 100 mL ampoule equipped with a J-Young's valve was charged with pre-catalyst (**Mn-1**; 10 mg, 0.02 mmol, 1 mol%) and base (*e.g.* KO^tBu, 22.4 mg, 0.20 mmol, 10 mol%). Solvent (4 mL) and ethanolamine (0.12 mL, 2.0 mmol) were added and the flask was sealed under an argon atmosphere before heating to the desired temperature (*e.g.* 150 °C) for the desired length of time (*e.g.* 24 hours) with stirring (400 rpm). After this period, the reaction vessel was allowed to cool to room temperature. The product was extracted into distilled water (5 mL) and any volatile components were removed under reduced pressure at 110 °C.

1.3 Ethanolamine Polymerisation Reaction Optimisation

Supplementary Table 1 Reaction optimisation for ethanolamine polymerisation catalysed by **Mn-1**.[a]

Entry	Cat. loading / mol %	Base	Base loading / mol %	Solvent	Temp. / °C	Time / h	Product(s)	Yield[b] / %
1	1	KO^{t}Bu	10	Toluene	150	24	PEI-1	81
2	1	KO^{t}Bu	10	THF	150	24	u-PEI-1	78
3	1	KO^{t}Bu	10	Anisole	150	24	u-PEI-1	72
4	1	K_2CO_3	10	Toluene	150	24	PEI-1	13
5	1	NaO^{t}Bu	10	Toluene	150	24	PA, u-PEI-1	75
6	0.5	KO^{t}Bu	10	Toluene	150	24	u-PEI-1	52
7	0.5	KO^{t}Bu	5	Toluene	150	24	u-PEI-1	54
8	1	KO^{t}Bu	10	Toluene	120	24	PA, PEI-1	29
9	-	KO^{t}Bu	10	Toluene	150	24	DEA	37
10	1	-	-	Toluene	150	24	-	-
11	2	KO^{t}Bu	20	Toluene	150	24	u-PEI-1	86
12	1	KO^{t}Bu	5	Toluene	150	24	u-PEI-1	49
13	1	KO^{t}Bu	15	Toluene	150	24	u-PEI-1	76
14[c]	1	KO^{t}Bu	10	Toluene	150	24	-	-
15[d]	1	KO^{t}Bu	10	Toluene	150	24	DEA	41
16[e]	1	KO^{t}Bu	10	Toluene	150	24	u-PEI-1	90
17[f]	1	KO^{t}Bu	10	Toluene	150	24	u-PEI-1	84

[a] Reaction conditions: 2 mmol ethanolamine, [0.5 M] in toluene (4 mL), 1 mol% **Mn-1**, 150 °C, 24h in a sealed 100 cm^3 system. [b] isolated yield. [c] Reaction carried out under 20 bar H_2 atmosphere. [d] $Mn(CO)_5Br$ precatalyst. [e] 0.25 equiv. ethylenediamine added. [f] 0.50 equiv. ethylenediamine added. DEA = diethanolamine.

Entry 1:

^{1}H NMR (500 MHz, D_2O): δ_H 8.40 (s, CH=N), 3.59 (br s), 3.11 (br s), 2.94 (br s), 2.77 (br s), 2.71 (br s).

^{13}C{^{1}H} NMR (126 MHz, D_2O): δ_C 61.8, 61.3, 60.0, 59.5, 51.7, 49.9, 49.6, 43.1, 42.0, 41.7, 41.1.

IR (ATR-FTIR, cm^{-1}): ν 3256m (br, O-H,/N-H), 2932m (C-H), 2843m (C-H), 1578s (N-H), 1458s (C-H), 1341s (O-H), 1057m (C-O).

DSC (N_2, °C): T_g –8.2 °C

GPC (H_2O, 30 °C, g mol^{-1}): 38,700 (Đ 1.21).

Entry 2:

^{1}H NMR (500 MHz, D_2O): δ_H 8.42 (s, C(H)=N), 8.06 (s, C(H)=N), 3.66 (br s), 3.57 (br s), 3.33 (s), 3.23 (s), 3.12 (s), 2.84 (s), 2.76 (s).

^{13}C{^{1}H} NMR (126 MHz, D_2O): δ_C 163.8 (C=N), 61.3, 61.0, 59.8, 51.5, 49.6, 43.1, 41.8.

IR (ATR-FTIR, cm^{-1}): ν 3271m (O-H/N-H), 2932m (C-H), 2866m (C-H), 1655m (C=N), 1578m (C-H), 1449s (C-H), 1387s (O-H), 1061s (C-O).

DSC (N_2, °C): T_g 0.5 °C

GPC (H_2O, 30 °C, g mol^{-1}): 22,500 (Đ 1.45).

Entry 3:

^{1}H NMR (500 MHz, D_2O): δ_H 8.50 (s, C(H)=N), 8.42 (s, C(H)=N), 8.05 (s, C(H)=N), 3.64 (s), 3.56 (s), 3.34 (br s), 3.22 (s), 3.12 (s), 2.83 (s), 2.73 (br s).

^{13}C{^{1}H} NMR (126 MHz, D_2O): δ_C 171.0 (C=N), 164.7 (C=N), 164.0 (C=N), 61.3, 61.1, 59.8, 49.6, 43.1, 41.8.

IR (ATR-FTIR, cm^{-1}): ν 3256m (O-H/N-H), 2930w (C-H), 2876w (C-H), 1570s (N-H), 1460m (C-H), 1383m (O-H), 1309s (C-N), 1061 (C-O).

DSC (N_2, °C): T_g –0.9 °C

GPC (H_2O, 30 °C, g mol^{-1}): 34,500 (Đ 1.21).

Entry 4:

Due to the low yield obtained for this reaction, infrared spectroscopy and thermal analysis were not carried out.

^{1}H NMR (500 MHz, D_2O): δ_H 3.61 (br s), 3.10 (br s), 3.01 (br s).

^{13}C{^{1}H} NMR (126 MHz, D_2O): δ_C 61.3, 59.8, 59.1, 43.2, 41.4.

GPC (H_2O, 30 °C, g mol^{-1}): 30,100 (Đ 1.26).

Entry 5:

^{1}H NMR (500 MHz, D_2O): δ_H 8.50 (s, CHCO), 8.41 (C(H)=N), 3.90 (s), 3.64 (s), 3.59-3.55 (m), 3.36-3.32 (m), 3.20 (s), 3.16 (s), 3.12 (t, J = 5.7 Hz), 2.95-2.91 (m), 2.73 (t, J = 5.6 Hz), 2.69-2.65 (m), 1.87 (s).

^{13}C{^{1}H} NMR (126 MHz, D_2O): δ_C 179.4 (C=O), 164.8 (C=N), 144.6, 143.9, 142.1, 62.5, 61.3, 60.2, 60.1, 60.0, 51.9, 49.7, 43.1, 42.1, 23.2.

IR (ATR-FTIR, cm^{-1}): ν 3273m (O-H/N-H), 2926m (C-H), 2857m (C-H), 1572s (N-H), 1431s (C-H), 1339m (O-H), 1057s (C-O).

GPC (H_2O, 30 °C, g mol^{-1}): 31,800 (Đ 1.28).

Entry 6:

^{1}H NMR (500 MHz, D_2O): δ_H 8.42 (s, C(H)=N), 8.03 (s, C(H)=N), 3.69 (br), 3.55 (br), 3.28 (s), 3.12 (br), 2.91-2.72 (br m/overlapping signals).

^{13}C{^{1}H} NMR (126 MHz, D_2O): δ_C 171.0 (C=N), 164.6 (C=N), 162.3 (C=N), 61.3, 60.0, 49.8, 46.4, 43.1, 41.6.

IR (ATR-FTIR, cm^{-1}): ν 3269w (O-H/N-H), 2931w (C-H), 2860w (C-H), 1655m (C=N), 1620m (C=O, amide), 1560m (N-H), 1396m (O-H), 1236s (C-O, amide).

DSC (N_2, °C): T_g –1.3 °C

GPC (H_2O, 30 °C, g mol^{-1}): 50,000 (Đ 1.08).

Entry 7:

^{1}H NMR (500 MHz, D_2O): δ_H 8.41 (s, C(H)=N), 8.05 (s, C(H)=N), 3.72 (br), 3.64 (s), 3.57 (s), 3.47 (br s), 3.35 (br s), 3.12 (s), 2.82-2.70 (br m/overlapping signals).

^{13}C{^{1}H} NMR (126 MHz, D_2O): δ_C 171.0 (C=N), 164.5 (C=N), 62.3, 61.3, 59.8, 58.0, 49.7, 43.1, 41.9, 40.0.

IR (ATR-FTIR, cm^{-1}): ν 3258m (O-H/N-H), 2934w (C-H), 2874m (C-H), 1647m (C=N), 1570s (N-H), 1458s (C-H), 1308s (C-N), 1057s (C-O).

GPC (H_2O, 30 °C, g mol^{-1}): 50,400 (Đ 1.19).

Entry 8:

^{1}H NMR (500 MHz, D_2O): δ_H 8.42 (s, CHCO), 8.05 (C(H)=N), 3.70 - 3.56 (br overlapping signals), 3.15 (s), 2.91 (br s), 2.69 (br s).

^{13}C{^{1}H} NMR (126 MHz, D_2O): δ_C 179.6 (C=O), 171.0 (C=N), 63.0, 60.1, 52.0, 49.7, 42.2.

IR (ATR-FTIR, cm^{-1}): ν 3250m (O-H/N-H), 2930m (C-H), 2845m (C-H), 1582s (N-H), 1454s (C-H), 1373s (O-H), 1292s (C-N), 1059 (C-O).

DSC (N_2, °C): T_g –1.1 °C

GPC (H_2O, 30 °C, g mol^{-1}): 36,400 (Đ 1.21).

Entry 9:

Heating ethanolamine (0.12 mL, 2 mmol) with KO^{t}Bu (0.2 mmol, 22.4 mg, 10 mol%) to 150 °C with stirring under inert conditions gave rise to an unexpected side reaction: the generation of *N*,*N*-diethanolamine (37% conversion by ^{1}H NMR).

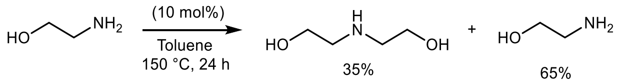

^{1}H NMR (500 MHz, D_2O): δ_H 3.57 (t, 1H, $^3J_{HH}$ 5.6 Hz, C*H*$_2$OH), 3.13 (t, 1H, $^3J_{HH}$ 5.6 Hz, C*H*$_2$NH$_2$).

^{13}C{^{1}H} NMR (126 MHz, D_2O): δ_C 61.3, 43.1.

Entry 11:

^{1}H NMR (500 MHz, D_2O): δ_H 8.42 (s, C(H)=N), 8.03 (s, C(H)=N), 3.63 (br), 3.35 (br), 3.13 (br), 2.93 (br), 2.79 (br), 2.73 (br) and 1.88 (br).

^{13}C{^{1}H} NMR (126 MHz, D_2O): δ_C 164.8 (C=N), 59.4, 49.6, 47.7, 41.4.

IR (ATR-FTIR, cm^{-1}): ν 3271m (O-H/N-H), 2938w (C-H), 2845w (C-H), 1575s (N-H), 1383s (O-H), 1344s (C-N), 1121m (C-O), 1057m (C-O).

GPC (H_2O, 30 °C, g mol^{-1}): 49,800 (Đ 1.09).

Entry 12:

^{1}H NMR (500 MHz, D_2O): δ_H 8.43 (s, C(H)=N), 8.04 (s, C(H)=N), 3.65 (br), 3.36 (br), 3.13 (br), 2.87 (br).

^{13}C{^{1}H} NMR (126 MHz, D_2O): δ_C 159.3 (C=N), 61.3, 60.7, 59.6, 58.3, 57.9.

IR (ATR-FTIR, cm^{-1}): ν 3283m (O-H/N-H), 2938w (C-H), 2872w (C-H), 1647s (C=N), 1570s (N-H), 1458s (C-H), 1386s (O-H), 1319s (C-N), 1059s (C-O).

GPC (H_2O, 30 °C, g mol^{-1}): M_n 50,700 g mol^{-1} (Đ 1.14).

Entry 13:

^{1}H NMR (500 MHz, D_2O): δ_H 8.42 (s, C(H)=N), 8.04 (s, C(H)=N), 3.66 (br), 3.57 (br), 3.35 (br), 3.12 (br), 2.89 (br).

^{13}C{^{1}H} NMR (126 MHz, D_2O): δ_C 163.0 (C=N), 61.3, 60.4, 49.6, 43.1.

IR (ATR-FTIR, cm^{-1}): ν 3260m (O-H/N-H), 2932m (C-H), 2870m (C-H), 1655w (C=N), 1570s (N-H), 1458s (C-H), 1389s (O-H), 1296s (C-N), 1059m (C-O).

GPC (H_2O, 30 °C, g mol^{-1}): M_n >55,000 g mol^{-1}.

Entry 15:

In line with the control experiment, carrying out the reaction in the presence of $Mn(CO)_5Br$ (1 mol%) and KO^{t}Bu results in the production of *N*,*N*-diethanolamine (41% conversion by ^{1}H NMR).

^{1}H NMR (500 MHz, D_2O): δ_H 3.68 (t, 1H, $^3J_{HH}$ 5.4 Hz, C*H*$_2$OH), 2.90 (t, 1H, $^3J_{HH}$ 5.4 Hz, C*H*$_2$NH$_2$).

^{13}C{^{1}H} NMR (126 MHz, D_2O): δ_C 60.2, 41.7.

HR-MS (EI, MeCN): *m/z* 106.0862 Da ([M+H]$^+$, expt. for $C_4H_{12}NO_2$ 106.0863 Da).

Entry 16:

^{1}H NMR (500 MHz, D_2O): δ_H 8.41 (s, CH=N), 3.64 (br s), 3.57 (br s), 3.27 (br s), 3.15 (br s), 3.04 (br s), 2.89 (br s), 2.66 (br s), 2.51 (br s).

^{13}C{^{1}H} NMR (126 MHz, D_2O): δ_C 171.0 (C=N), 60.0, 59.2, 51.9, 49.5, 44.8, 42.8.

IR (ATR-FTIR, cm^{-1}): ν 3261m (O-H/N-H), 2932m (C-H), 2866m (C-H), 1578s (N-H), 1458s (C-H), 1341s (C-N), 1051s (C-O).

GPC (H_2O, 30 °C, g mol^{-1}): M_n 47,600 g mol^{-1} (Đ 1.11).

Entry 17:

^{1}H NMR (500 MHz, D_2O): δ_H 8.41 (CH=N), 3.63 (br s), 3.56 (br s), 3.13 (br s), 2.98 (br s), 2.89 (br s), 2.69 (br s), 2.46 (br s).

^{13}C{^{1}H} NMR (126 MHz, D_2O): δ_C 171.0 (C=N), 168.3 (C=N), 60.3, 60.1, 51.9, 50.3, 49.9, 42.9.

IR (ATR-FTIR, cm^{-1}): ν 3258m (O-H/N-H), 2945w (C-H), 2853w (C-H), 1578s (N-H), 1377s (C-N), 1061w (C-O).

GPC (H_2O, 30 °C, g mol^{-1}): M_n 41,200 g mol^{-1} (Đ 1.12).

1.3.1 IR Spectra

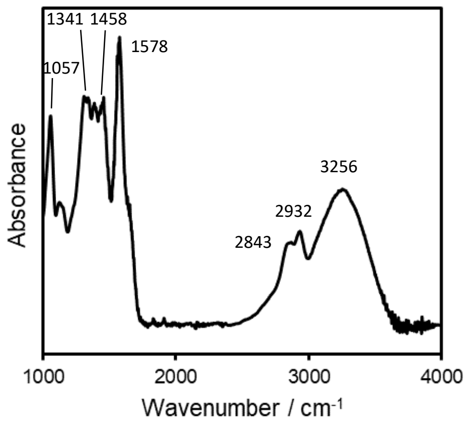

Supplementary Figure 1 IR (ATR-FTIR) spectrum corresponding to product of Supplementary Table 1, Entry 1.

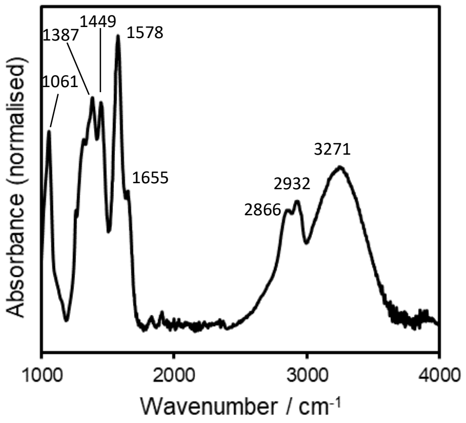

Supplementary Figure 2 IR (ATR-FTIR) spectrum corresponding to product of Supplementary Table 1, Entry 2.

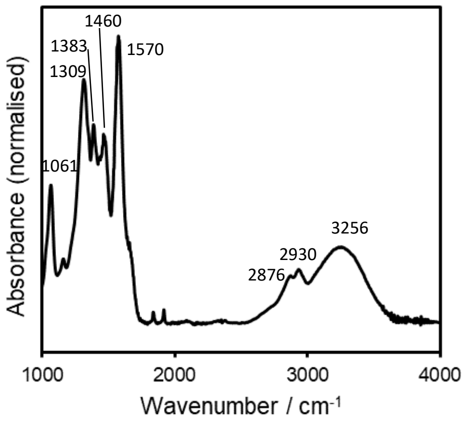

Supplementary Figure 3 IR (ATR-FTIR) spectrum corresponding to product of Supplementary Table 1; Entry 3.

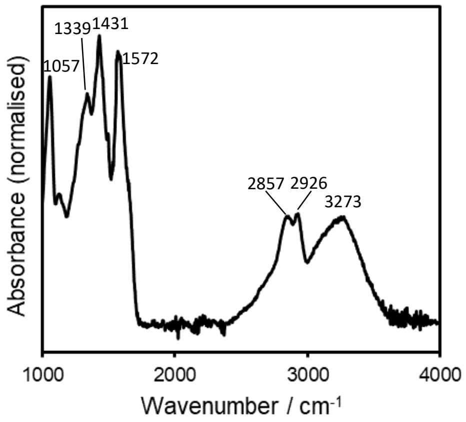

Supplementary Figure 4 IR (ATR-FTIR) spectrum corresponding to product of Supplementary Table 1; Entry 5.

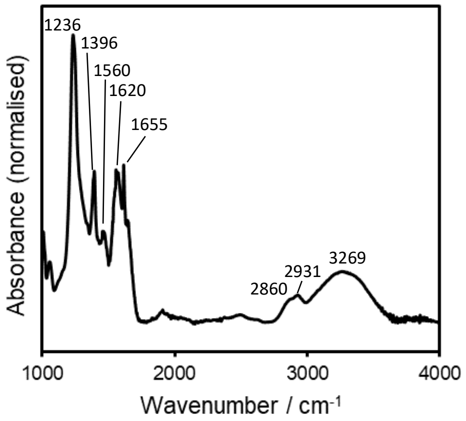

Supplementary Figure 5 IR (ATR-FTIR) spectrum corresponding to product of Supplementary Table 1; Entry 6.

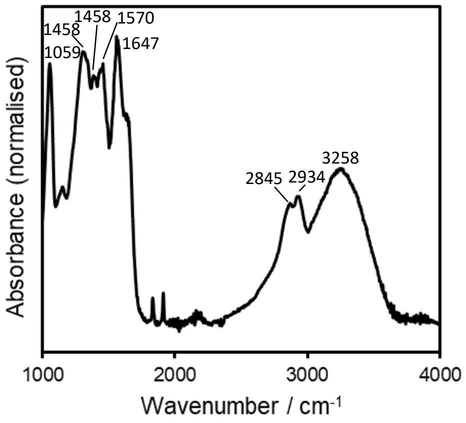

Supplementary Figure 6 IR (ATR-FTIR) spectrum corresponding to product of Supplementary Table 1; Entry 7.

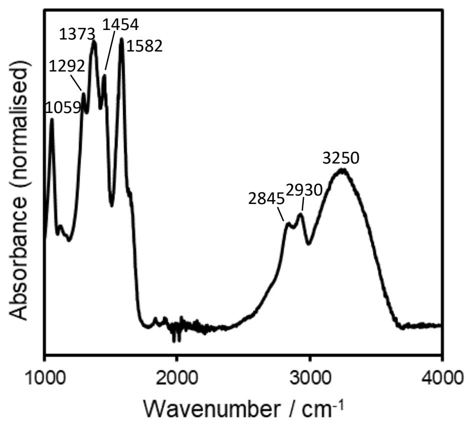

Supplementary Figure 7 IR (ATR-FTIR) spectrum corresponding to product of Supplementary Table 1; Entry 8.

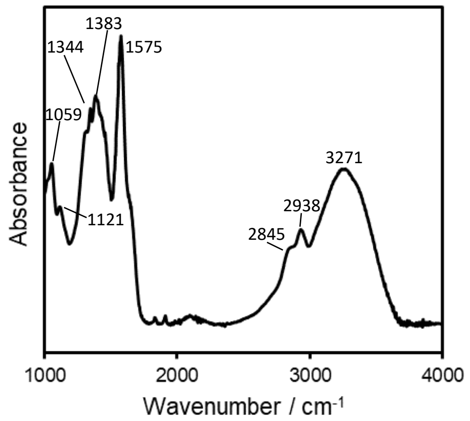

Supplementary Figure 8 IR (ATR-FTIR) spectrum corresponding to product of Supplementary Table 1; Entry 11.

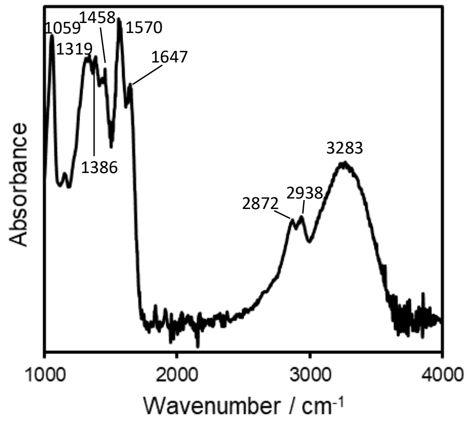

Supplementary Figure 9 IR (ATR-FTIR) spectrum corresponding to product of Supplementary Table 1; Entry 12.

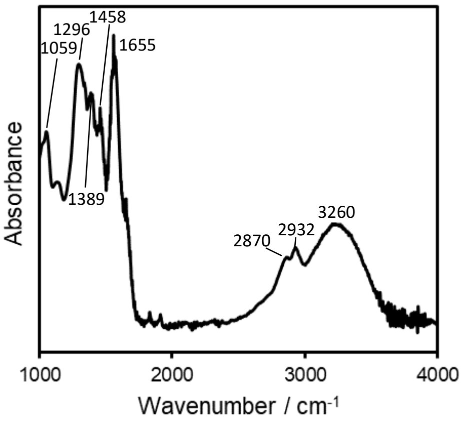

Supplementary Figure 10 IR (ATR-FTIR) spectrum corresponding to product of Supplementary Table 1; Entry 13.

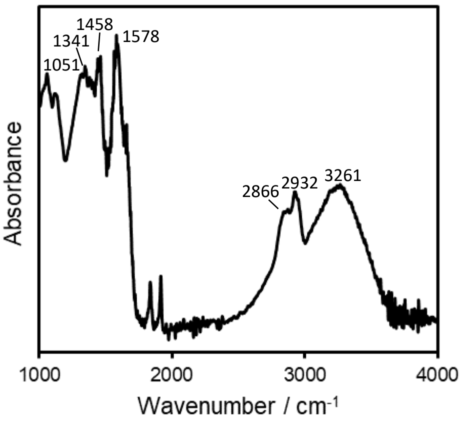

Supplementary Figure 11 IR (ATR-FTIR) spectrum corresponding to product of Supplementary Table 1; Entry 16.

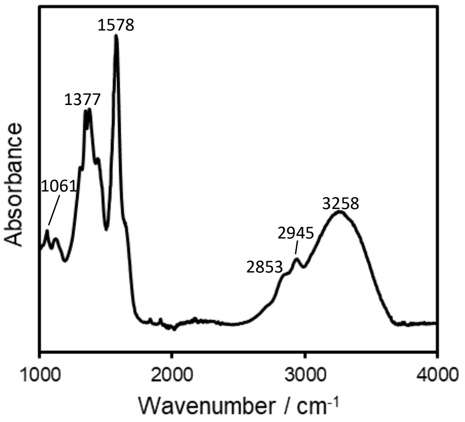

Supplementary Figure 12 IR (ATR-FTIR) spectrum corresponding to product of Supplementary Table 1; Entry 17.

1.3.2 GPC Data

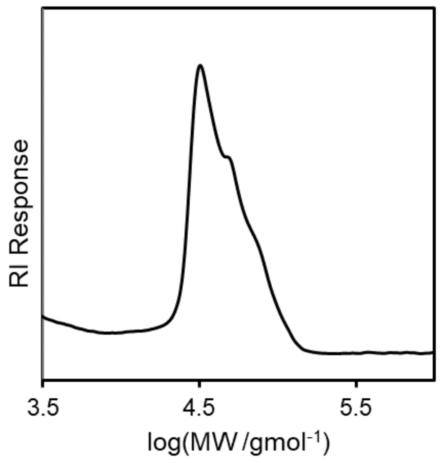

Supplementary Figure 13 GPC chromatograph of product corresponding to Supplementary Table 1; Entry 1.

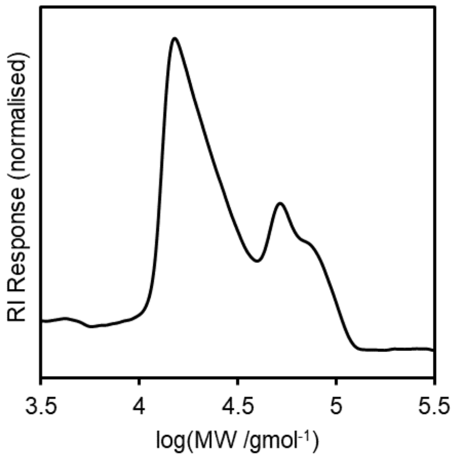

Supplementary Figure 14 GPC chromatograph of product corresponding to Supplementary Table 1; Entry 2.

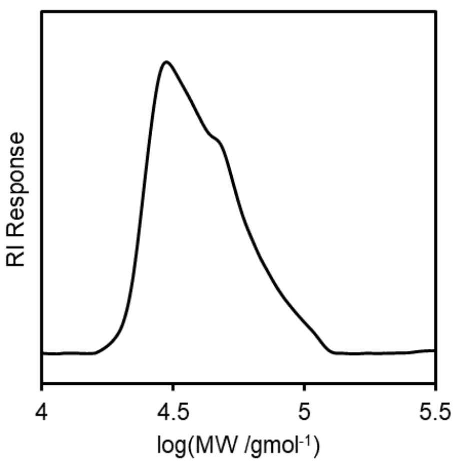

Supplementary Figure 15 Measured GPC data for product corresponding to Supplementary Table 1; Entry 3.

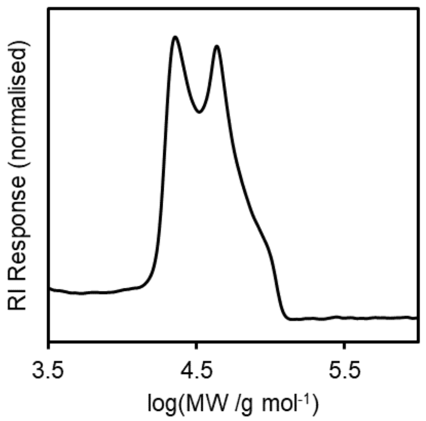

Supplementary Figure 16 GPC chromatograph of product corresponding to Supplementary Table 1; Entry 5.

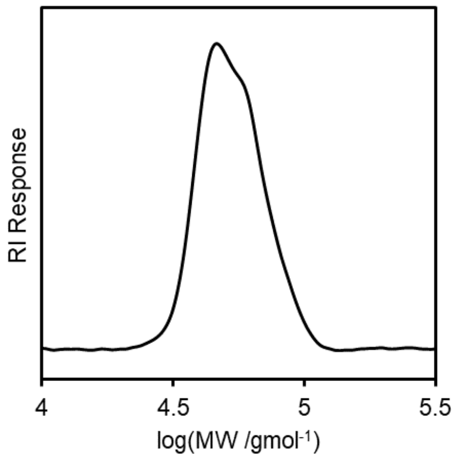

Supplementary Figure 17 Measured GPC data for product corresponding to Supplementary Table 1; Entry 6.

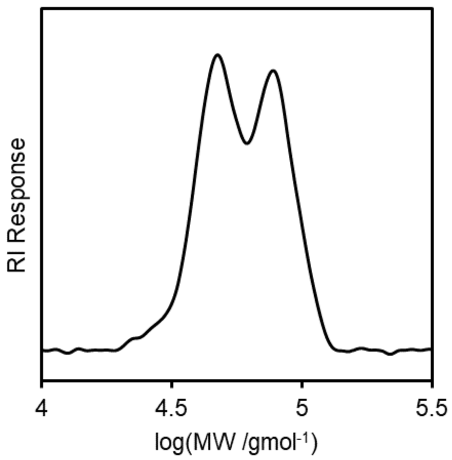

Supplementary Figure 18 Measured GPC data for product corresponding to Supplementary Table 1; Entry 7.

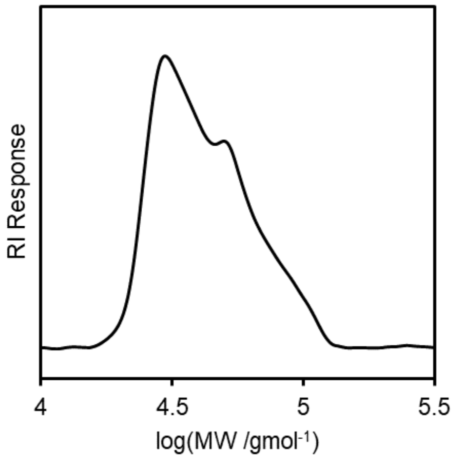

Supplementary Figure 19 Measured GPC data for product corresponding to Supplementary Table 1; Entry 8.

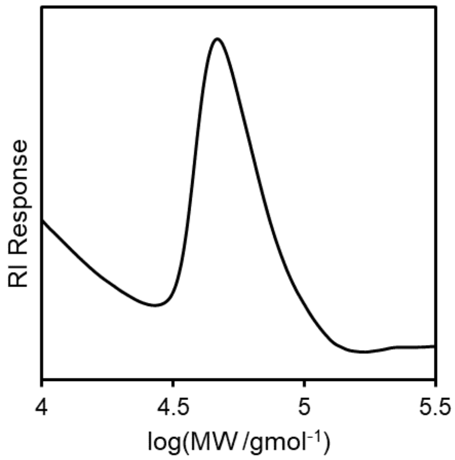

Supplementary Figure 20 Measured GPC data for product corresponding to Supplementary Table 1; Entry 11.

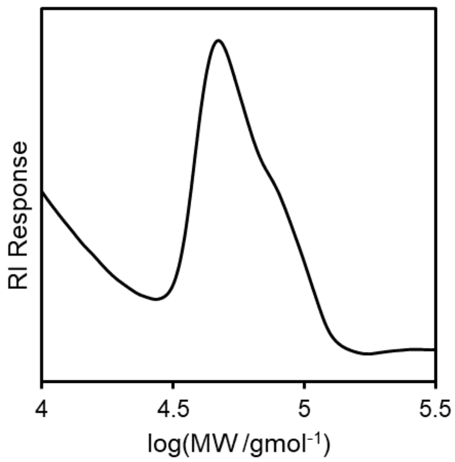

Supplementary Figure 21 Measured GPC data for product corresponding to Supplementary Table 1; Entry 12.

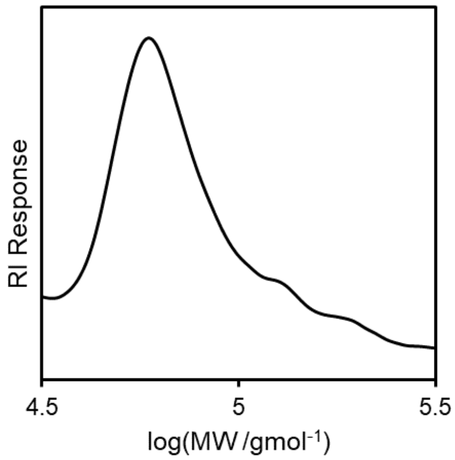

Supplementary Figure 22 Measured GPC data for product corresponding to Supplementary Table 1; Entry 13.

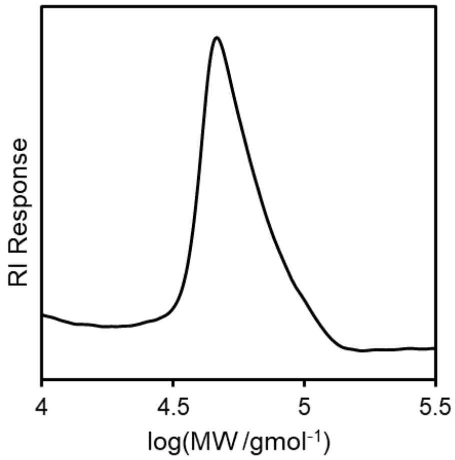

Supplementary Figure 23 Measured GPC data for product corresponding to Supplementary Table 1; Entry 16.

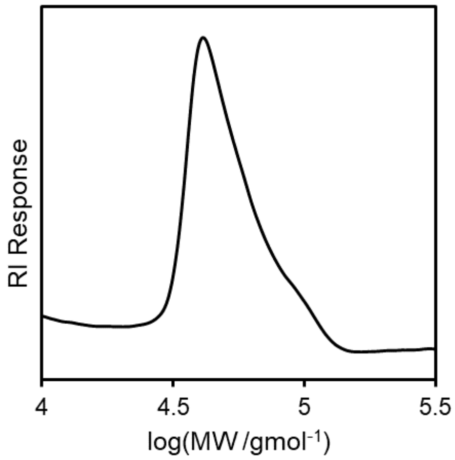

Supplementary Figure 24 Measured GPC data for product corresponding to Supplementary Table 1; Entry 17.

1.3.3 NMR Spectra

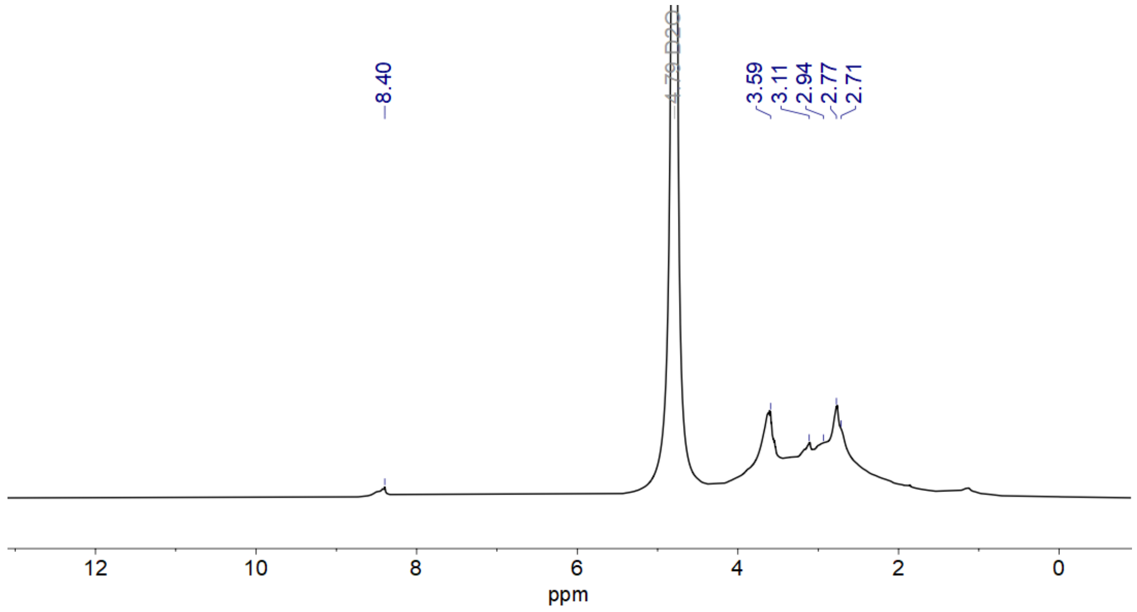

Supplementary Figure 25 ^{1}H NMR (500 MHz, D_2O) spectrum corresponding to product of Supplementary Table 1; Entry 1.

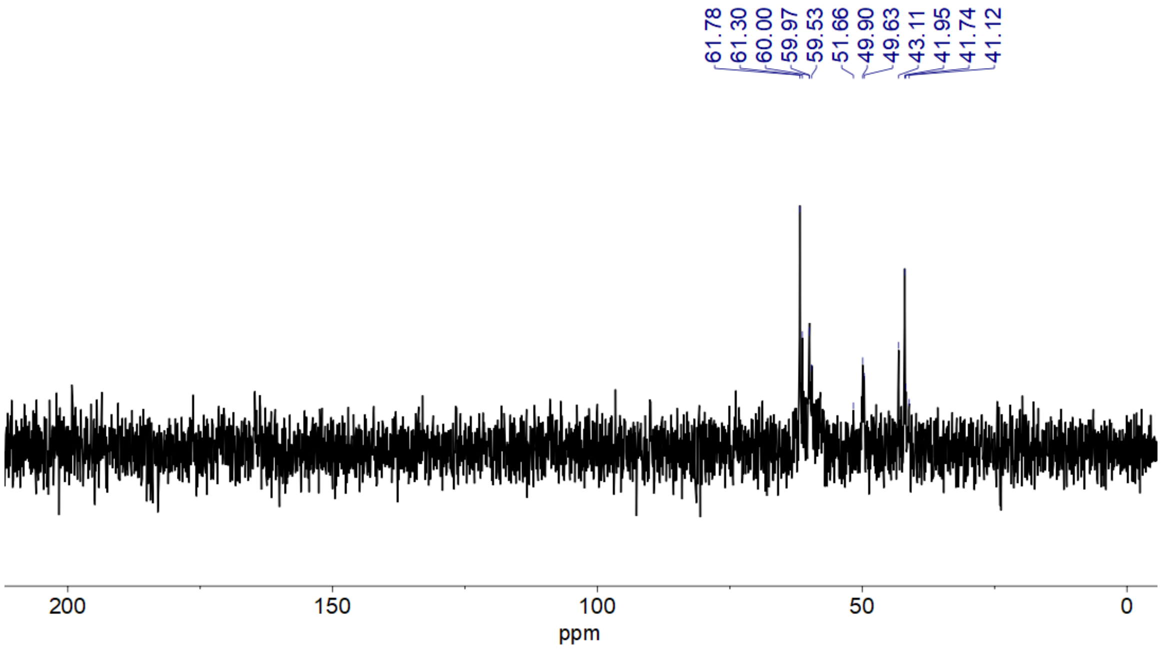

Supplementary Figure 26 $^{13}C\{^{1}H\}$ NMR (126 MHZ, D_2O) spectrum corresponding to product of Supplementary Table 1; Entry 1.

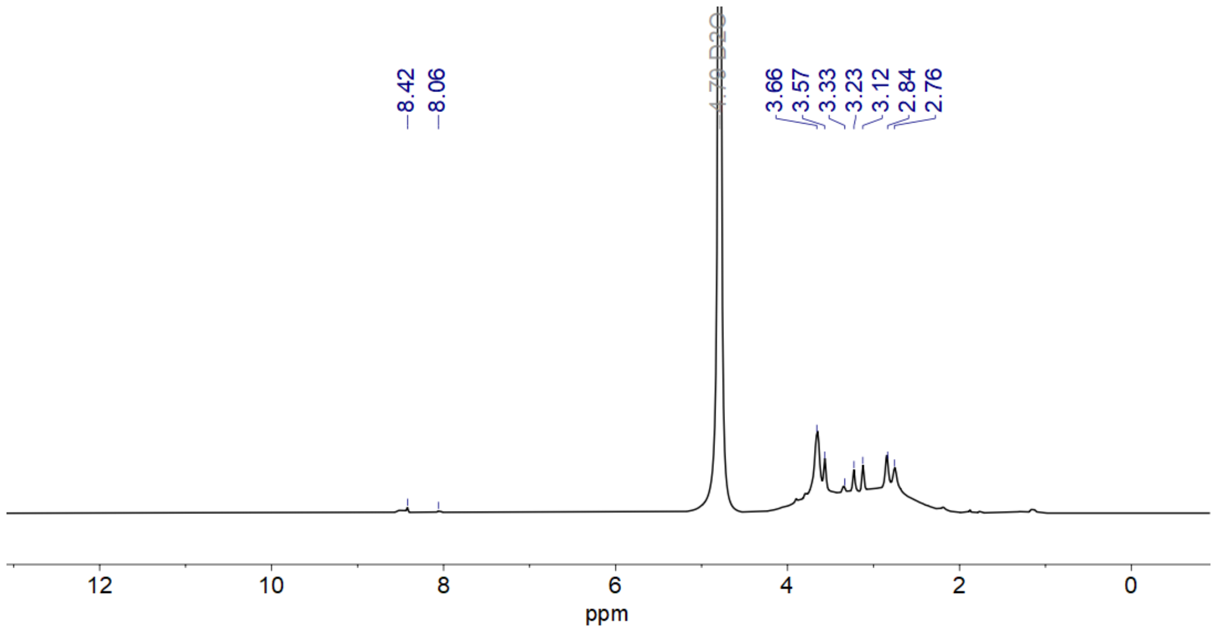

Supplementary Figure 27 ^{1}H NMR (500 MHz, D_2O) spectrum corresponding to product of Supplementary Table 1; Entry 2.

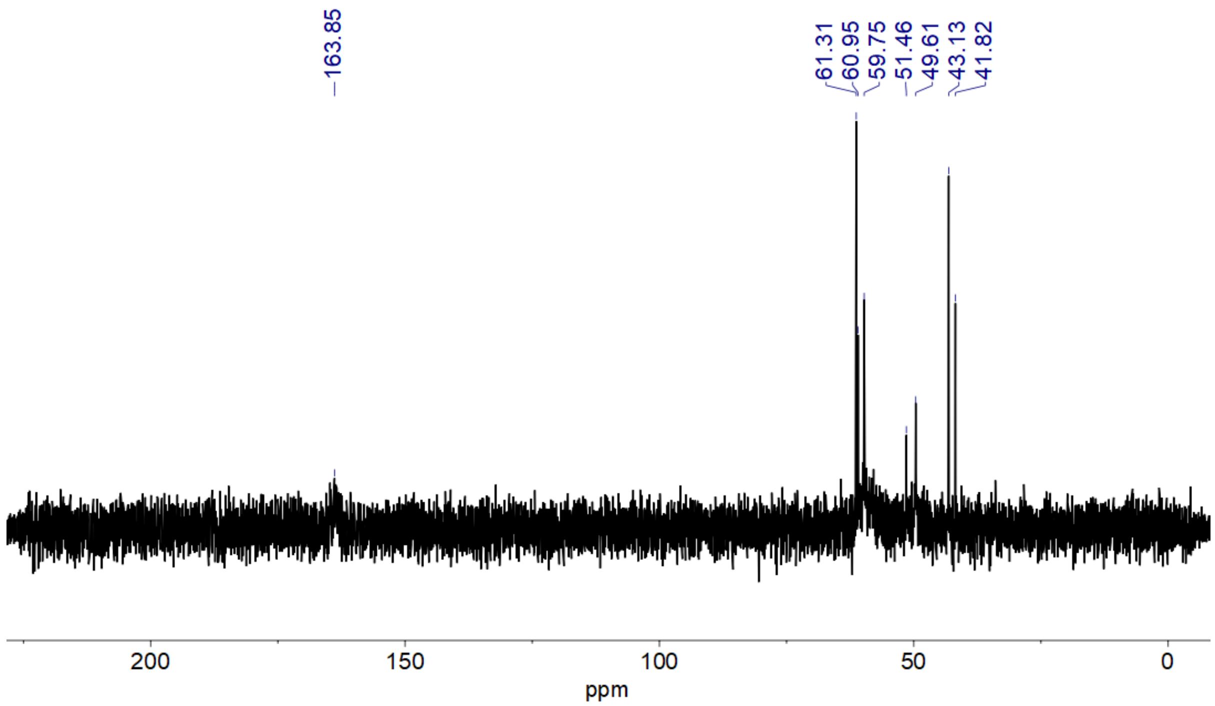

Supplementary Figure 28 $^{13}C\{^1H\}$ NMR (126 MHZ, D_2O) spectrum corresponding to product of Supplementary Table 1; Entry 2.

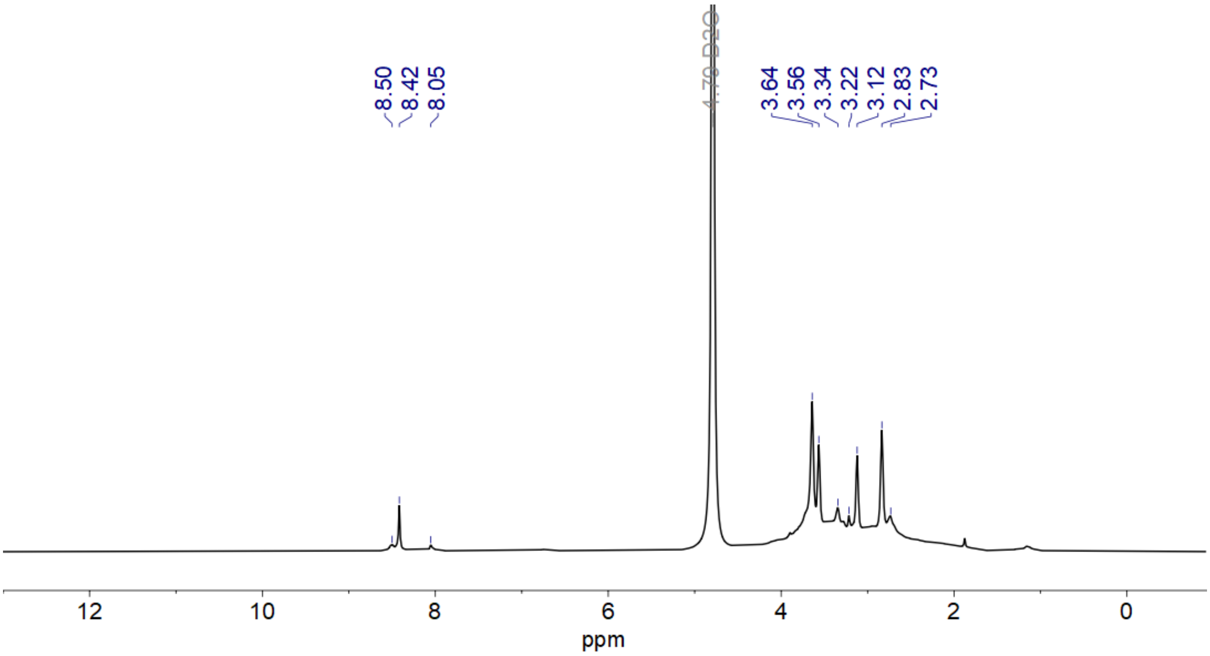

Supplementary Figure 29 ^{1}H NMR (500 MHz, D_2O) spectrum corresponding to product of Supplementary Table 1; Entry 3.

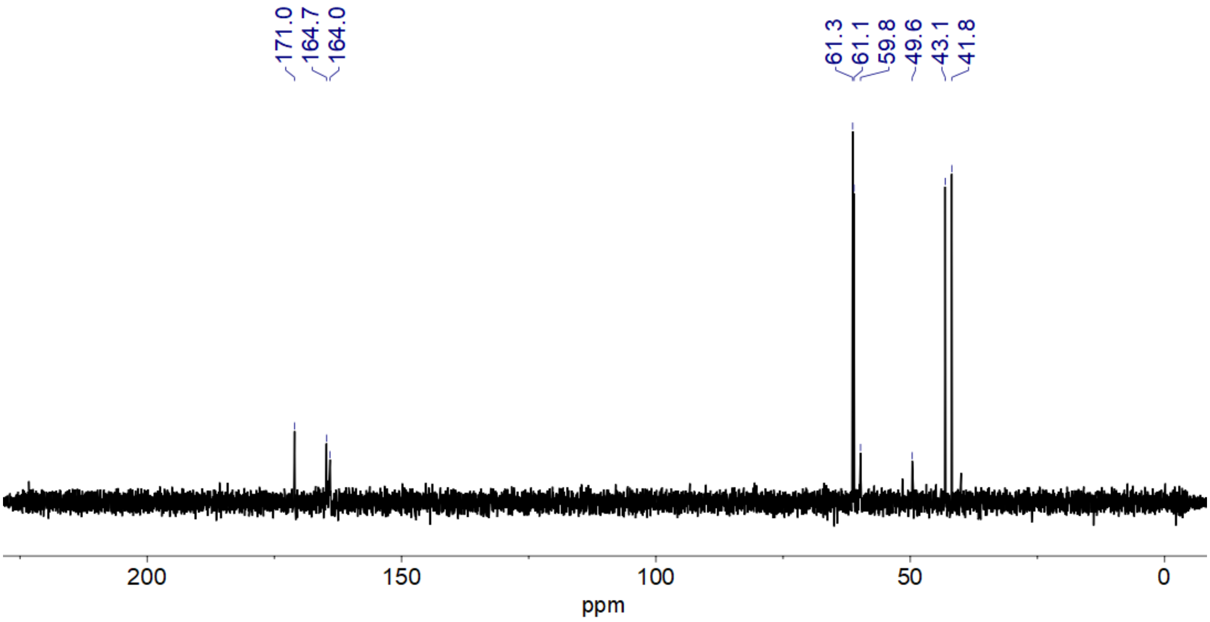

Supplementary Figure 30 $^{13}C\{^{1}H\}$ NMR (126 MHZ, D_2O) spectrum corresponding to product of Supplementary Table 1; Entry 3.

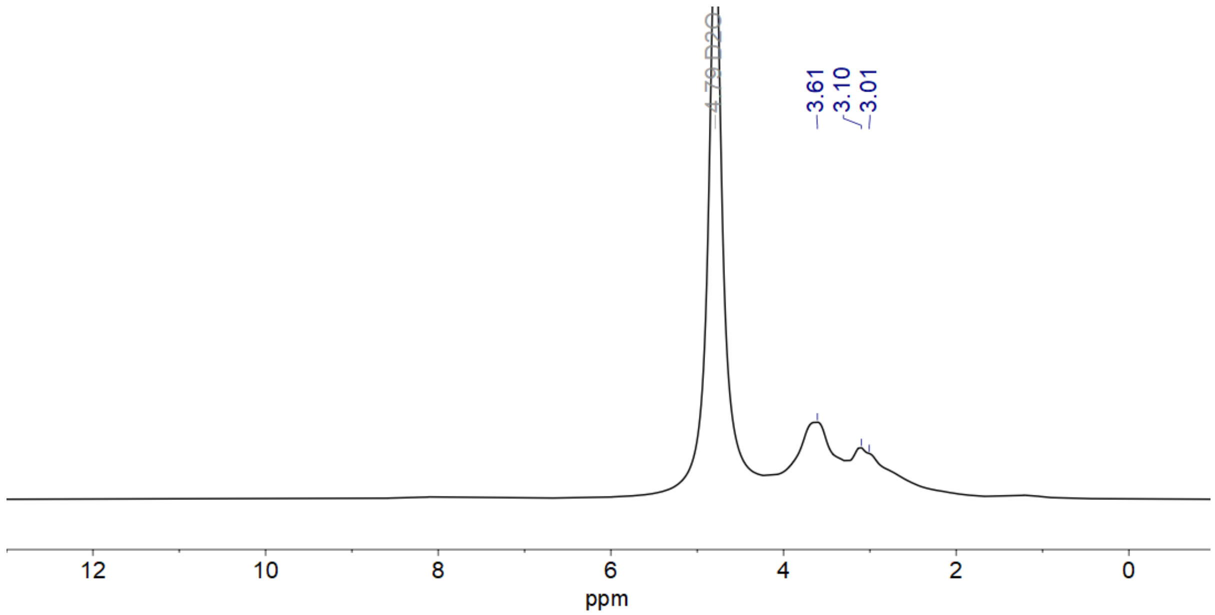

Supplementary Figure 31 ^{1}H NMR (500 MHz, D_2O) spectrum corresponding to product of Supplementary Table 1; Entry 4.

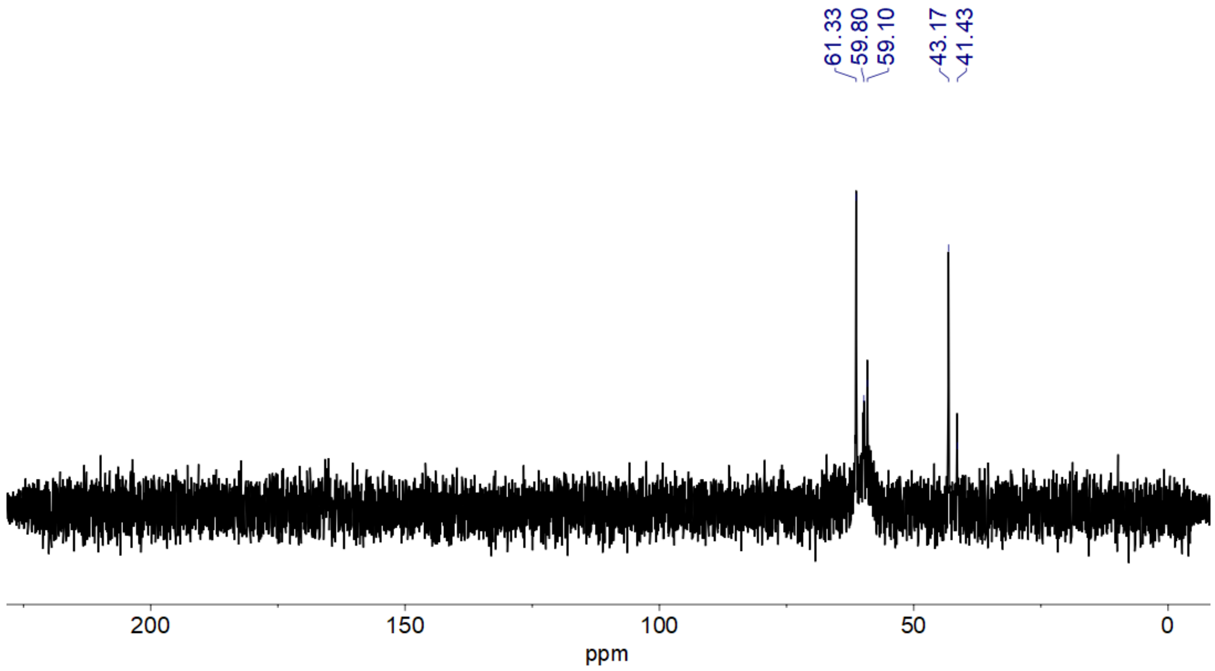

Supplementary Figure 32 $^{13}C\{^{1}H\}$ NMR (126 MHz, D_2O) spectrum corresponding to product of Supplementary Table 1; Entry 4.

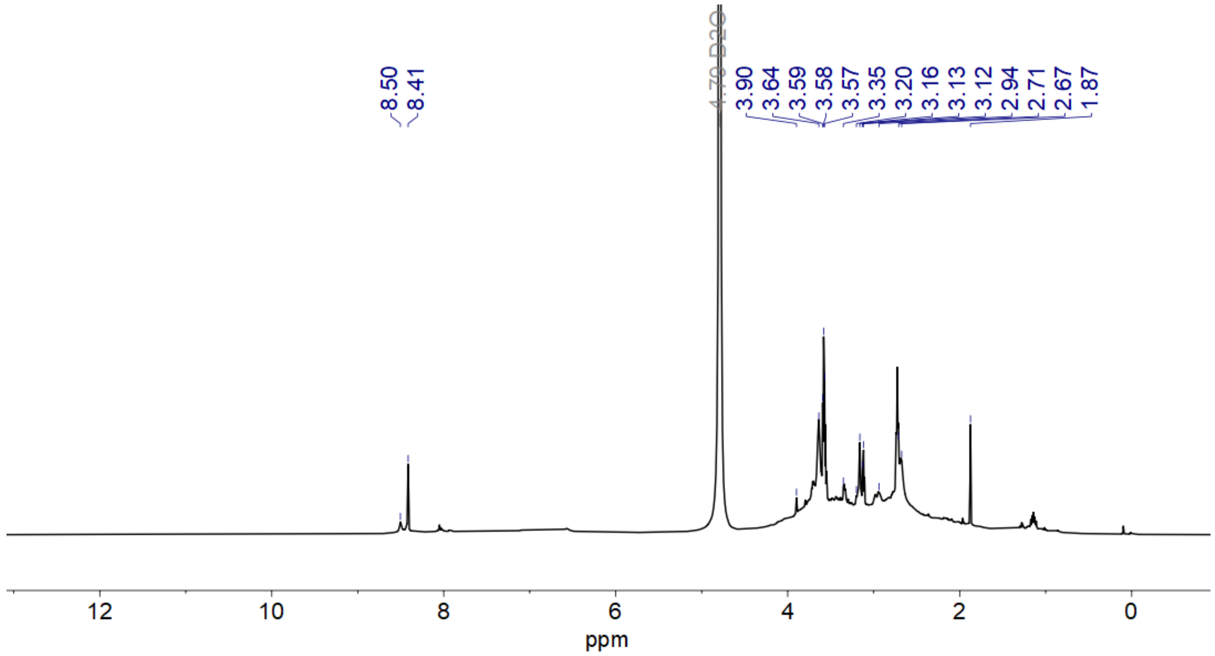

Supplementary Figure 33 ^{1}H NMR (500 MHz, D_2O) spectrum corresponding to product of Supplementary Table 1; Entry 5.

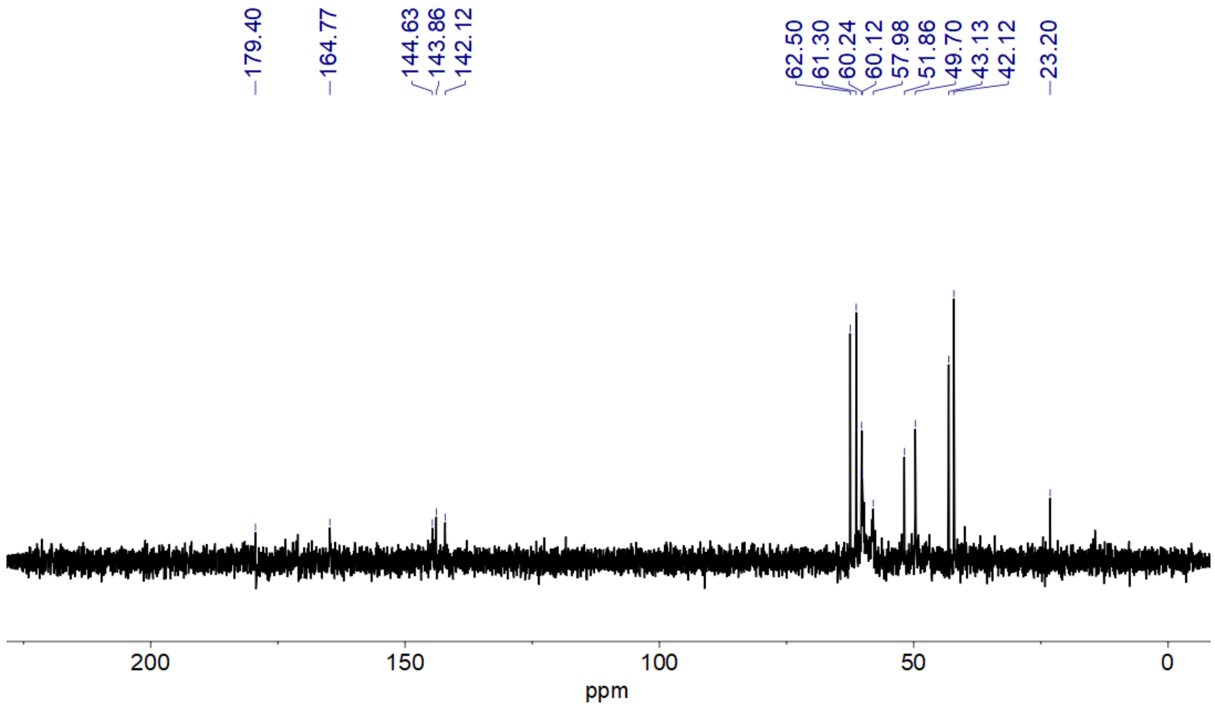

Supplementary Figure 34 $^{13}C\{^{1}H\}$ NMR (126 MHz, D_2O) spectrum corresponding to product of Supplementary Table 1; Entry 5.

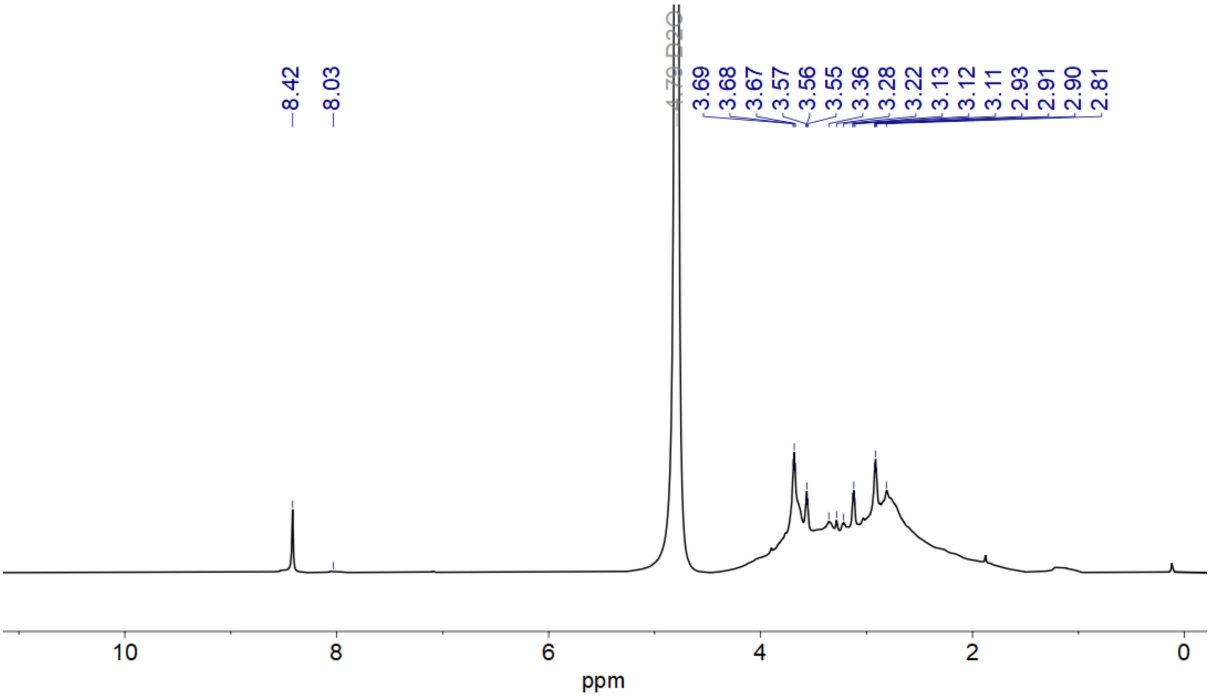

Supplementary Figure 35 ^{1}H NMR (500 MHz, D_2O) spectrum corresponding to product of Supplementary Table 1; Entry 6.

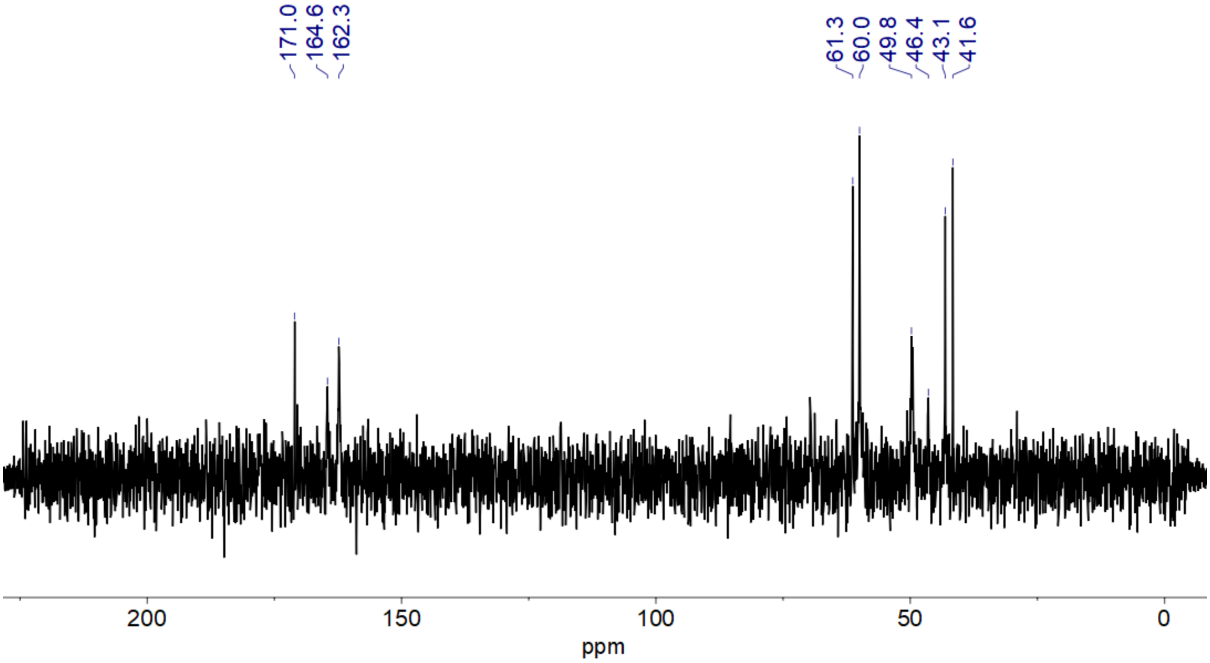

Supplementary Figure 36 $^{13}C\{^1H\}$ NMR (126 MHz, D_2O) spectrum corresponding to product of Supplementary Table 1; Entry 6.

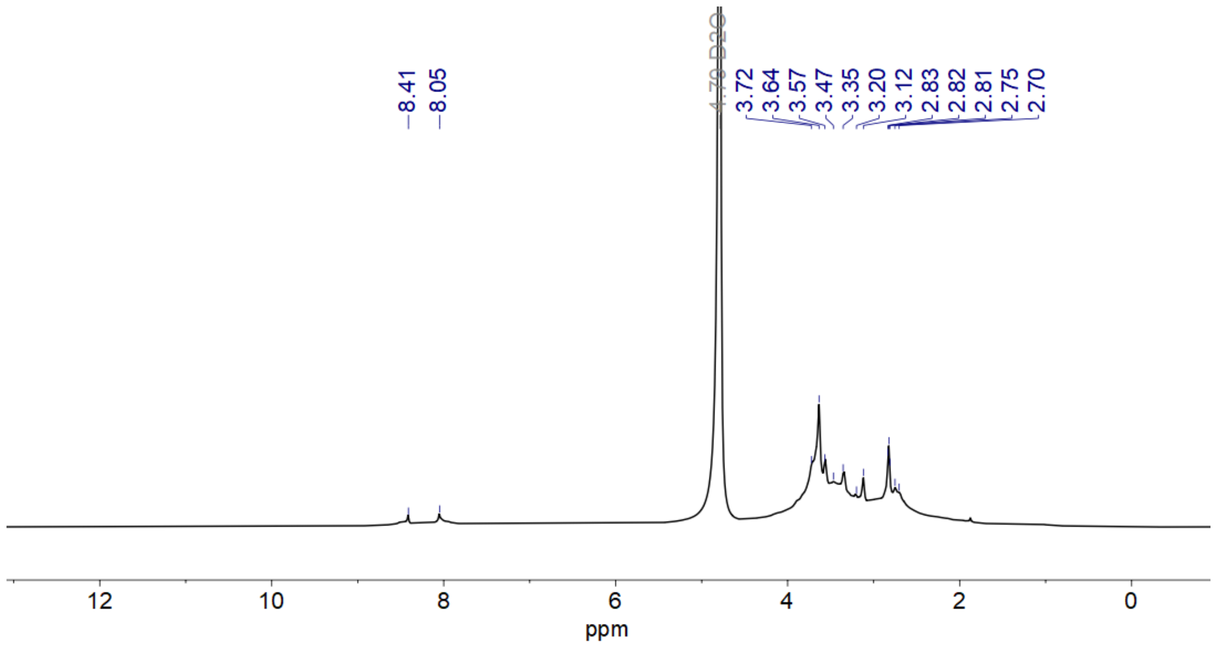

Supplementary Figure 37 ^{1}H NMR (500 MHz, D_2O) spectrum corresponding to product of Supplementary Table 1; Entry 7.

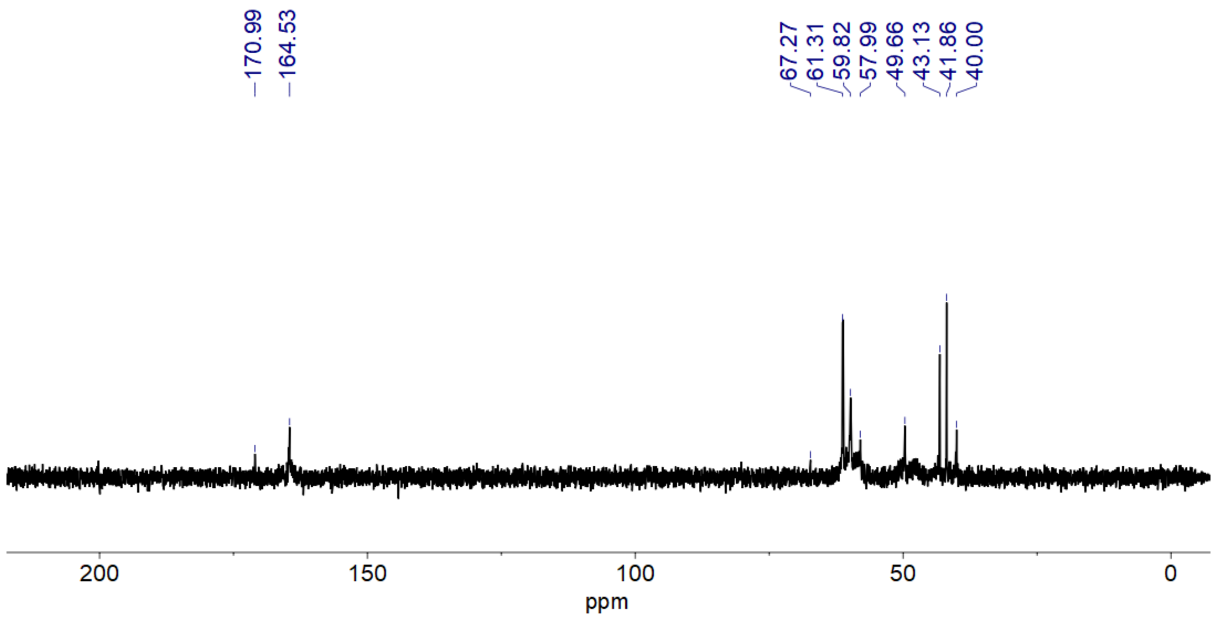

Supplementary Figure 38 $^{13}C\{^{1}H\}$ NMR (126 MHz, D_2O) spectrum corresponding to product of Supplementary Table 1; Entry 7.

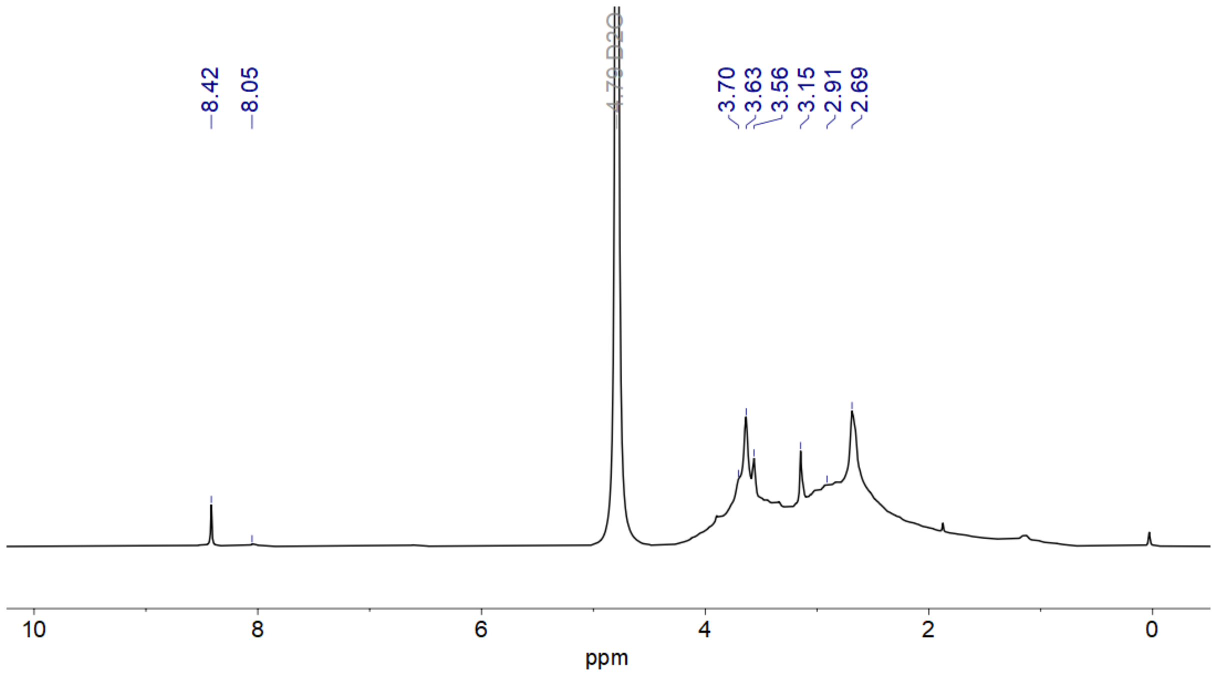

Supplementary Figure 39 ^{1}H NMR (500 MHz, D_2O) spectrum corresponding to product of Supplementary Table 1; Entry 8.

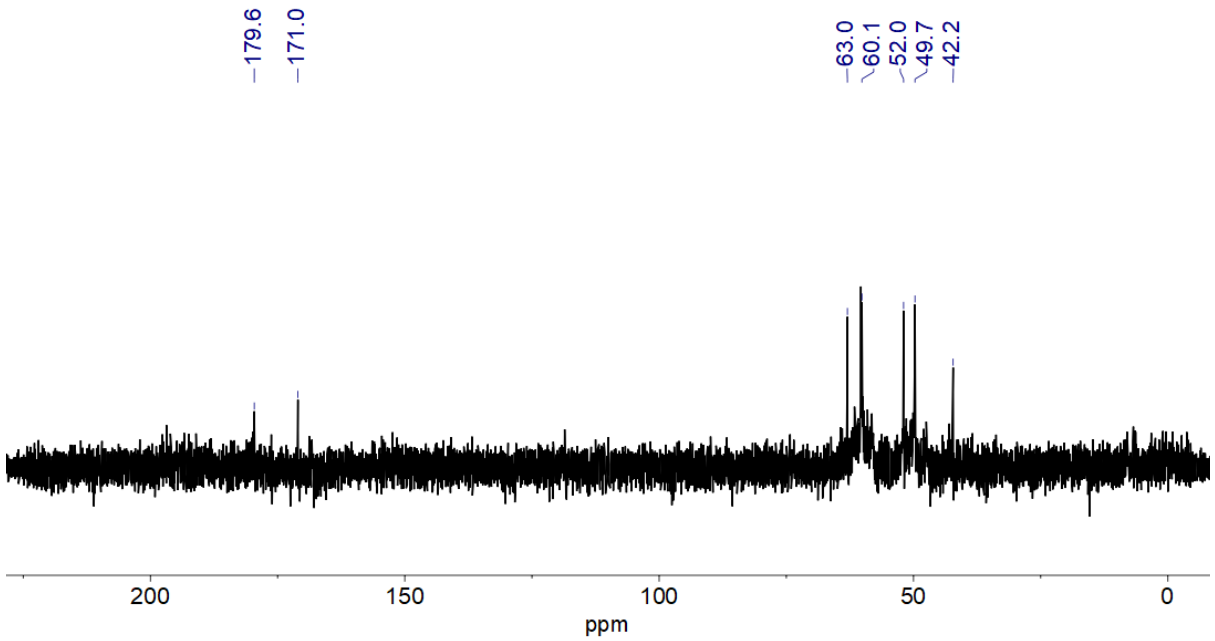

Supplementary Figure 40 $^{13}C\{^{1}H\}$ NMR (126 MHz, D_2O) spectrum corresponding to product of Supplementary Table 1; Entry 8.

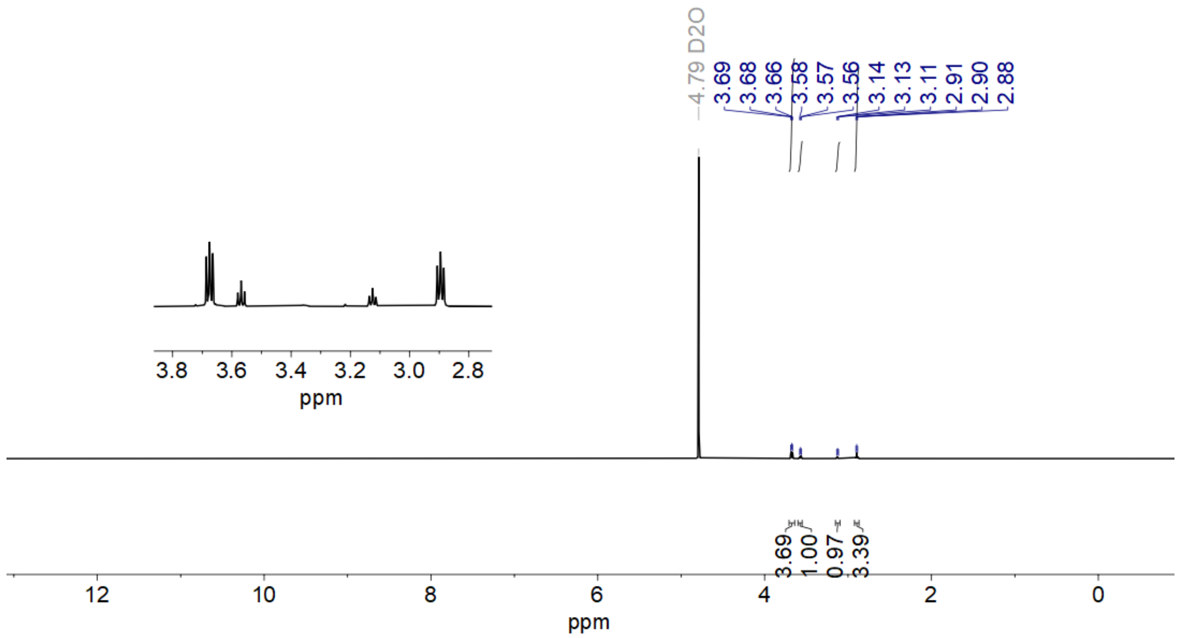

Supplementary Figure 41 ^{1}H NMR (500 MHz, D_2O) spectrum corresponding to product of Supplementary Table 1; Entry 9.

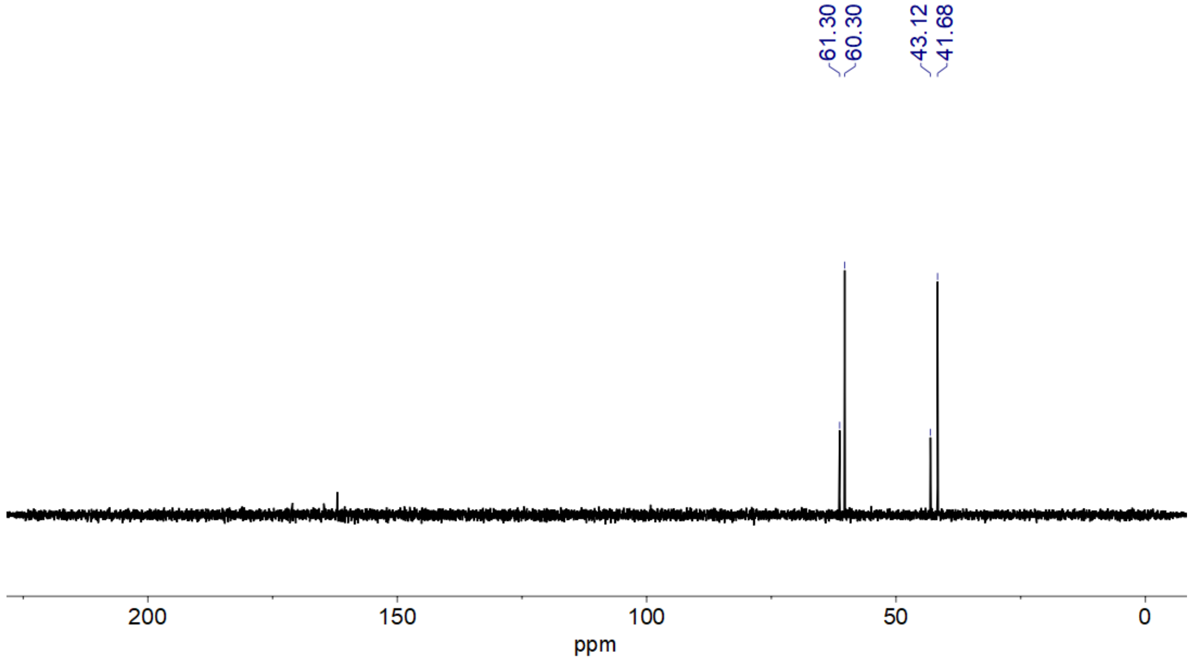

Supplementary Figure 42 $^{13}C\{^{1}H\}$ NMR (126 MHz, D_2O) spectrum corresponding to product of Supplementary Table 1; Entry 9.

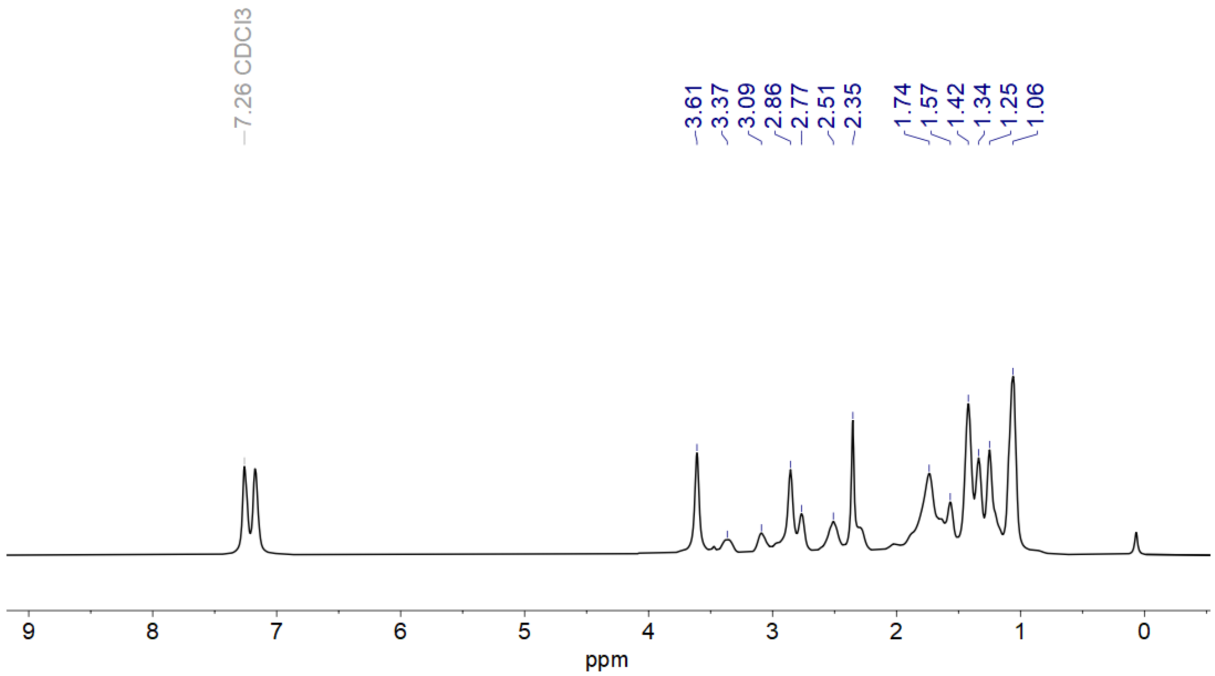

Supplementary Figure 43 ^{1}H NMR (500 MHz, $CDCl_3$) spectrum corresponding to product of Supplementary Table 1; Entry 10.

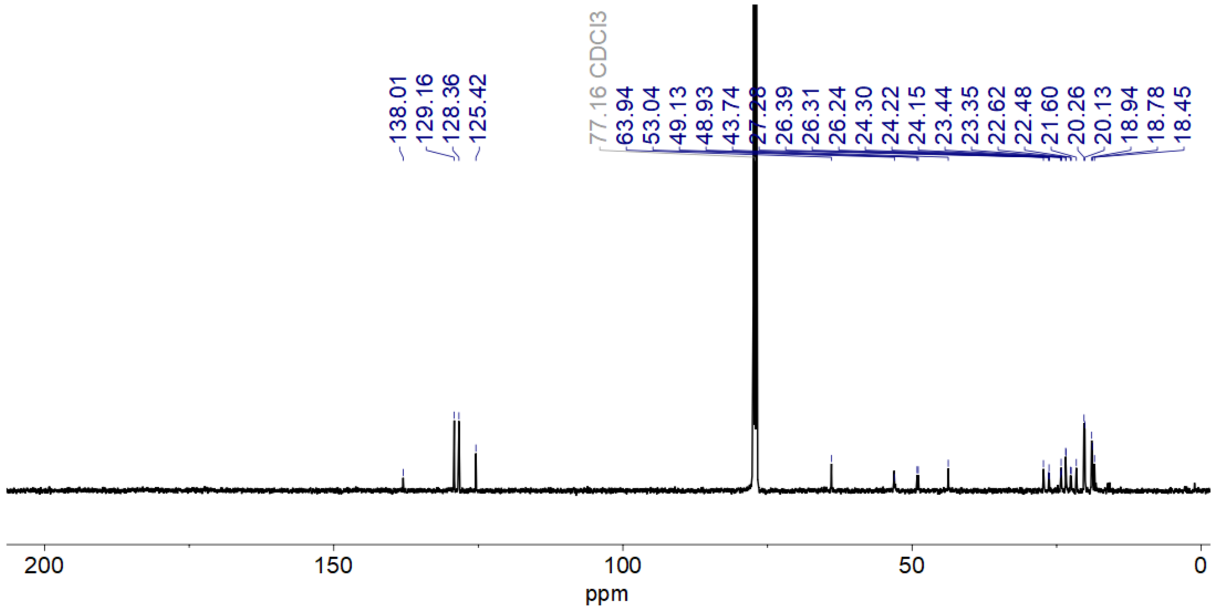

Supplementary Figure 44 $^{13}C\{^{1}H\}$ NMR (126 MHz, $CDCl_3$) spectrum corresponding to product of Supplementary Table 1; Entry 10.

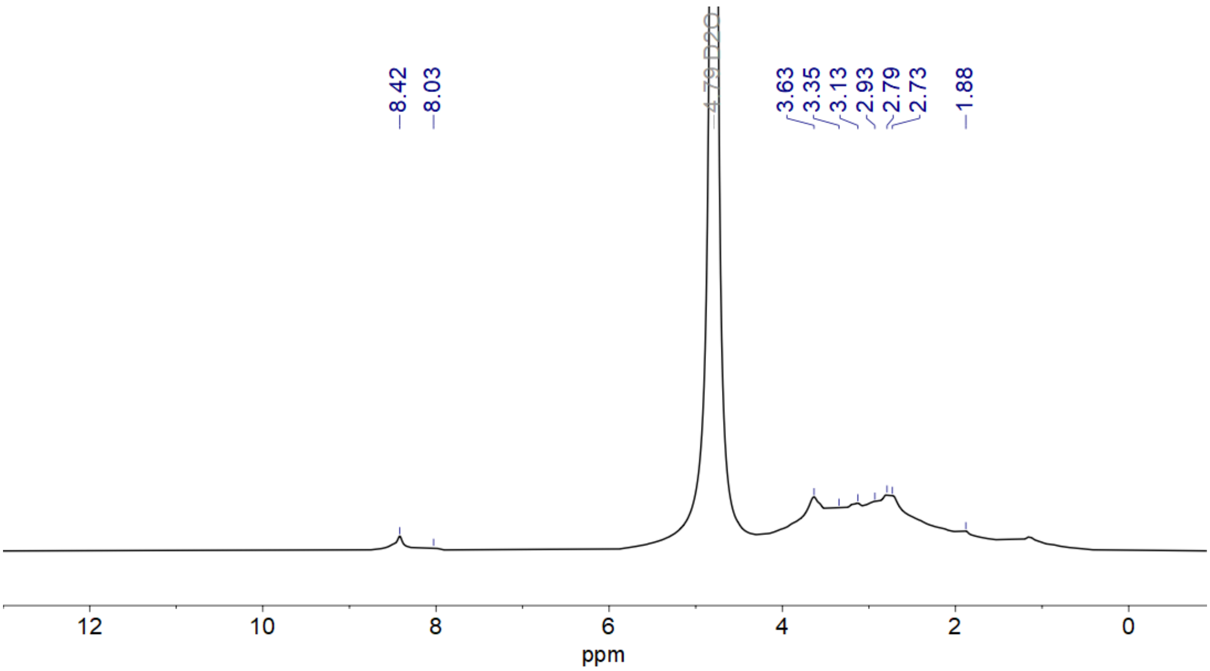

Supplementary Figure 45 ^{1}H NMR (500 MHz, D_2O) spectrum of the product corresponding to Supplementary Table 1; Entry 11.

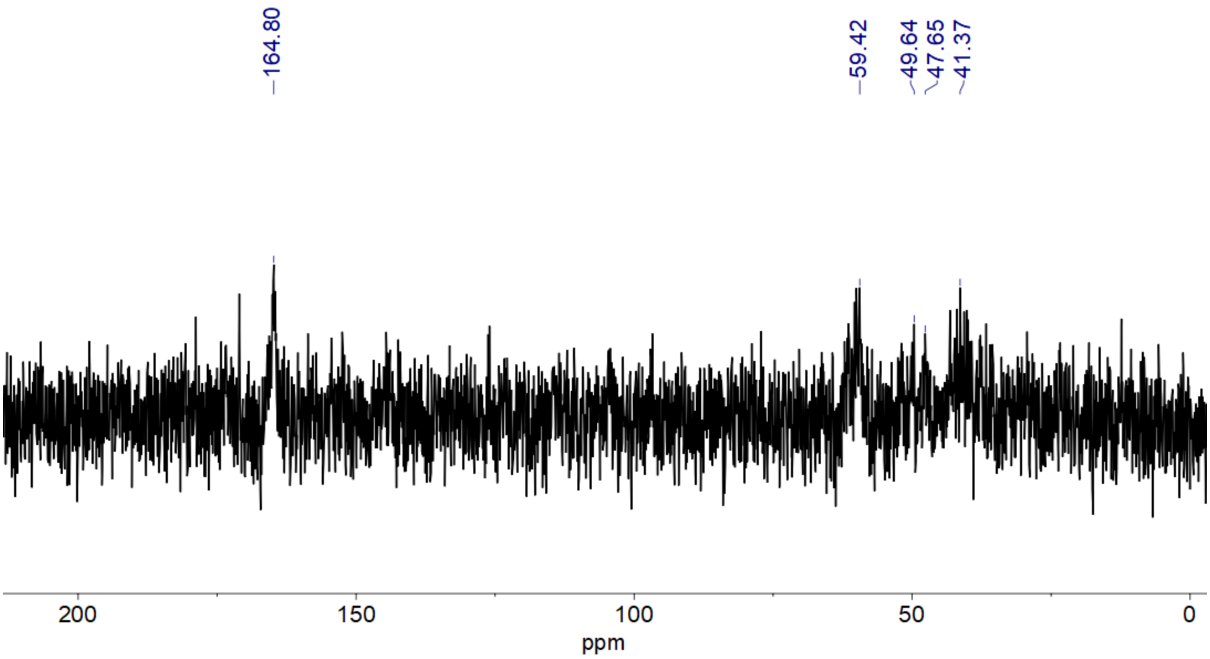

Supplementary Figure 46 $^{13}C\{^{1}H\}$ NMR (126 MHz, D_2O, l.b. 5 Hz) spectrum corresponding to product of Supplementary Table 1; Entry 11.

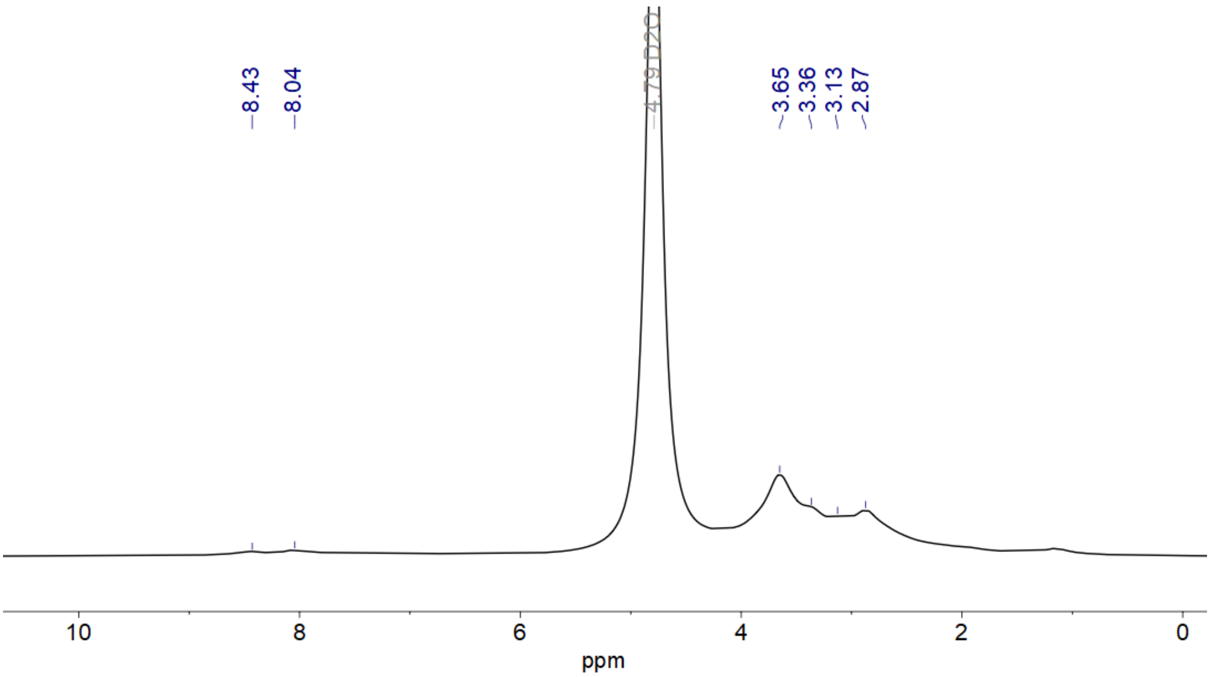

Supplementary Figure 47 ^{1}H NMR (500 MHz, D_2O) spectrum of the product corresponding to Supplementary Table 1; Entry 12.

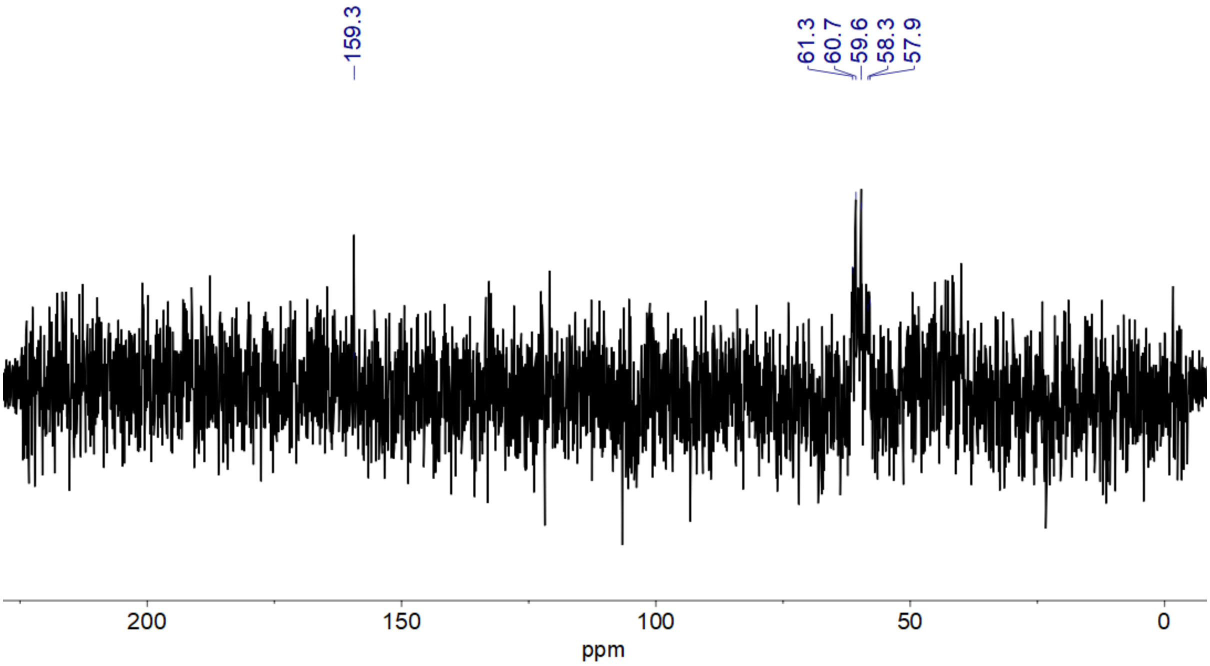

Supplementary Figure 48 $^{13}C\{^{1}H\}$ NMR (126 MHz, D_2O, l.b. 5 Hz) spectrum corresponding to product of Supplementary Table 1; Entry 12.

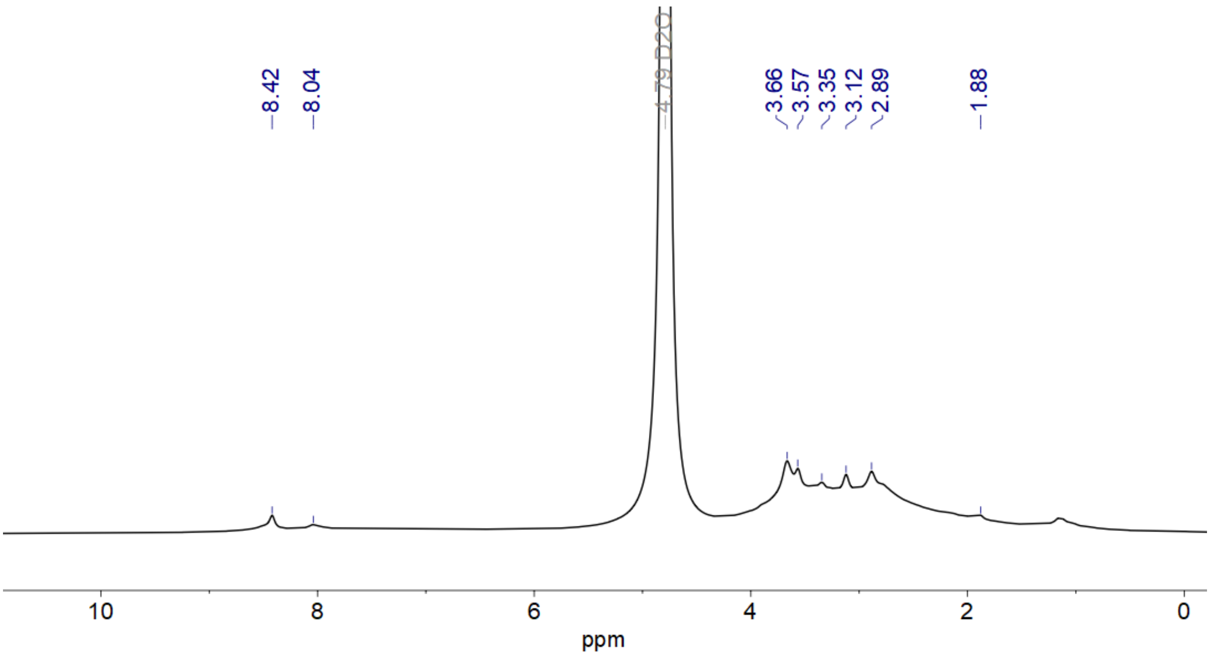

Supplementary Figure 49 ^{1}H NMR (500 MHz, D_2O) spectrum of the product corresponding to Supplementary Table 1; Entry 13

.

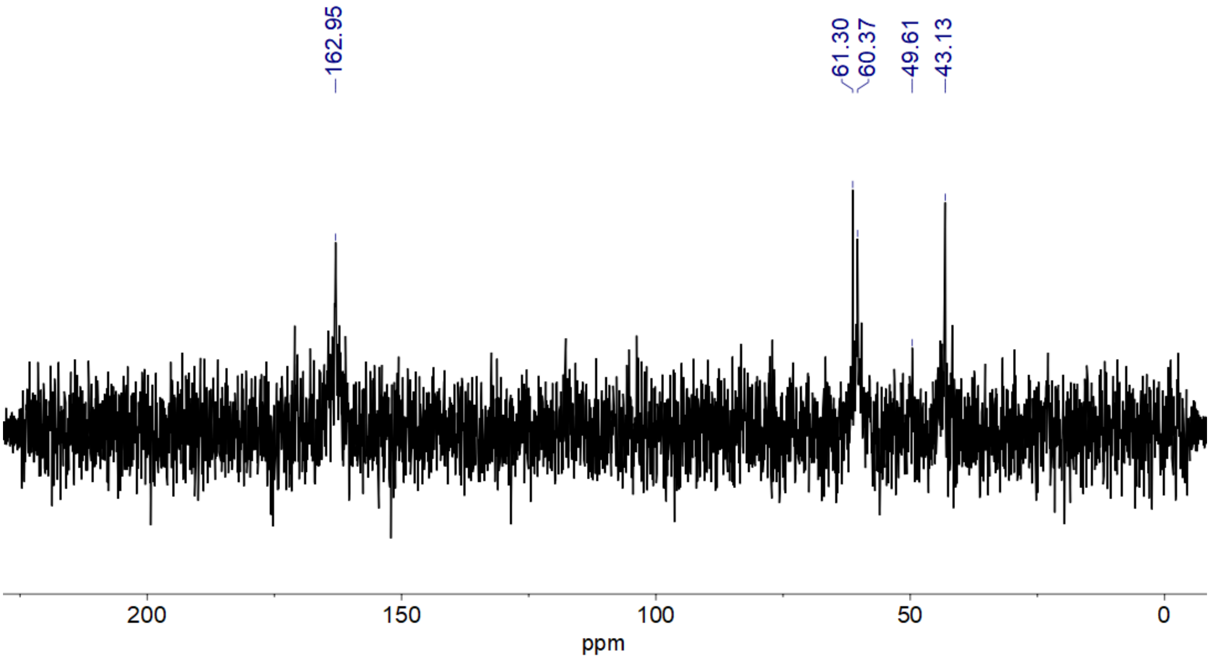

Supplementary Figure 50 $^{13}C\{^{1}H\}$ NMR (126 MHz, D_2O, l.b. 5 Hz) spectrum corresponding to product of Supplementary Table 1; Entry 13.

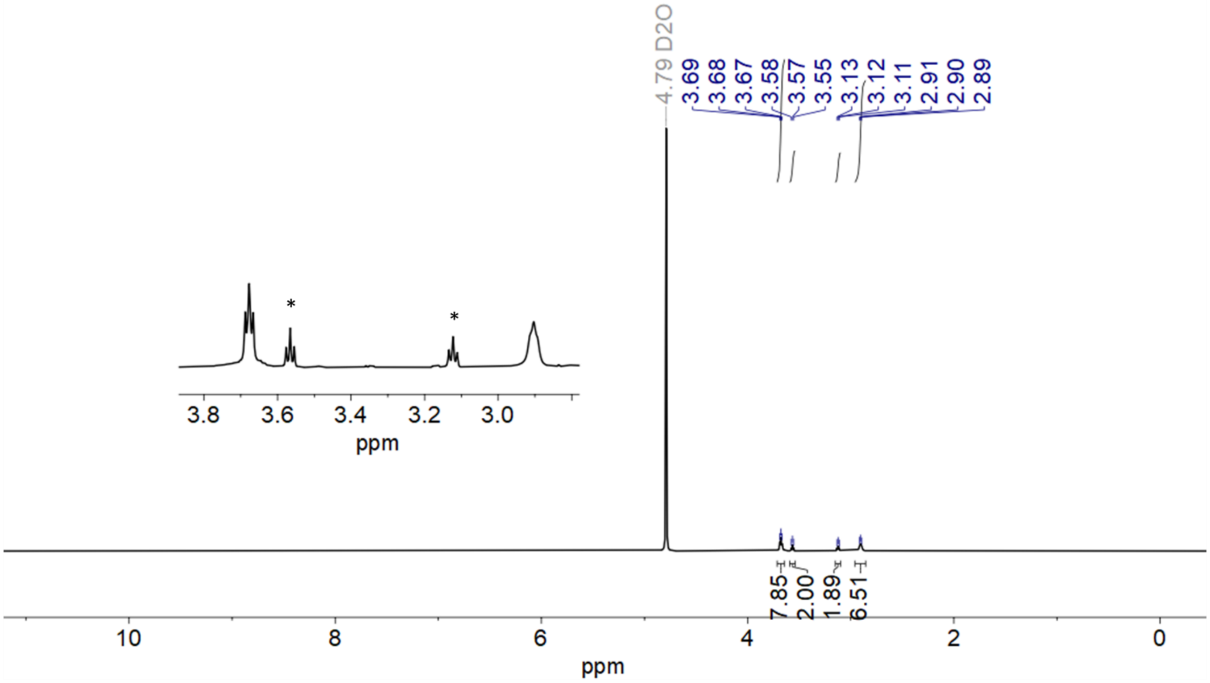

Supplementary Figure 51 ^{1}H NMR (500 MHz, D_2O) spectrum of the product corresponding to Supplementary Table 1; Entry 15. *denotes residual ethanolamine.

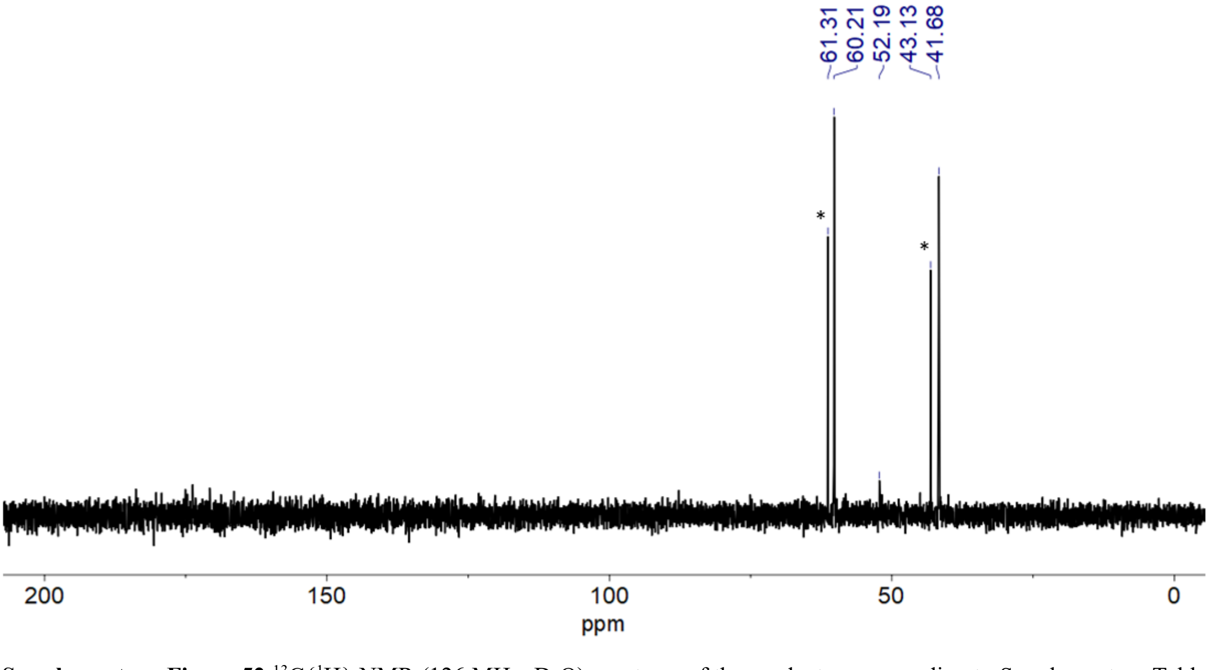

Supplementary Figure 52 $^{13}C\{^{1}H\}$ NMR (126 MHz, D_2O) spectrum of the product corresponding to Supplementary Table 1; Entry 15. *denotes residual ethanolamine.

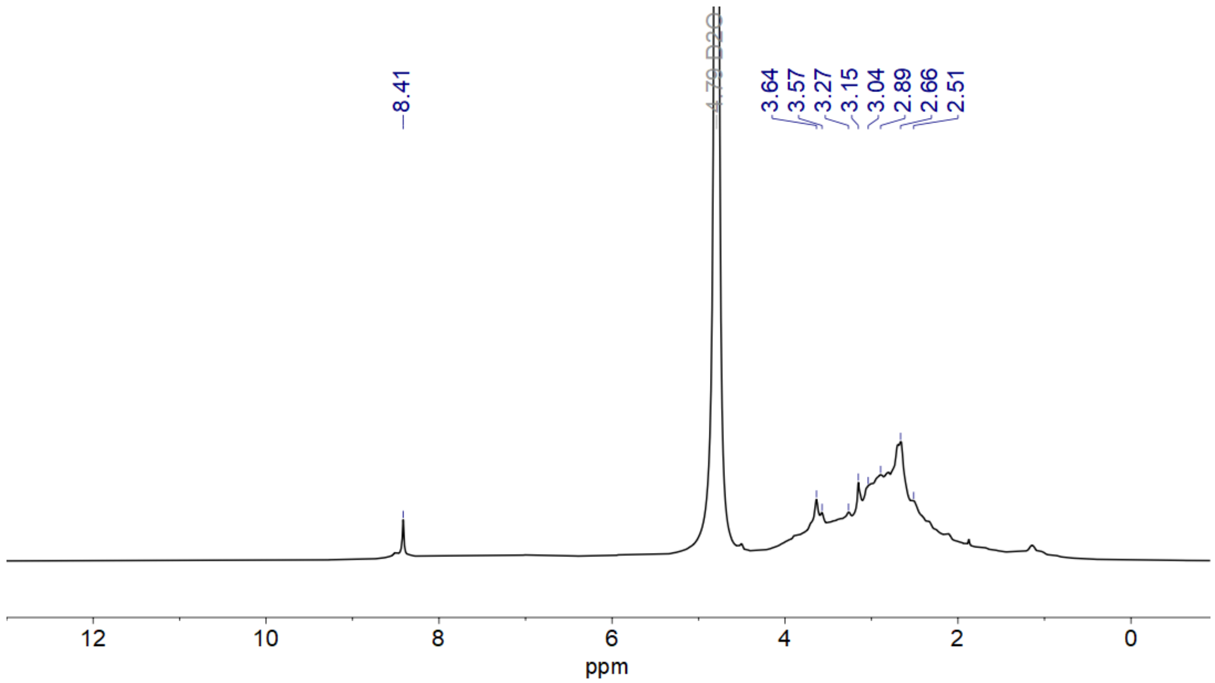

Supplementary Figure 53 ^{1}H NMR (500 MHz, D_2O) spectrum of the product corresponding to Supplementary Table 1; Entry 16.

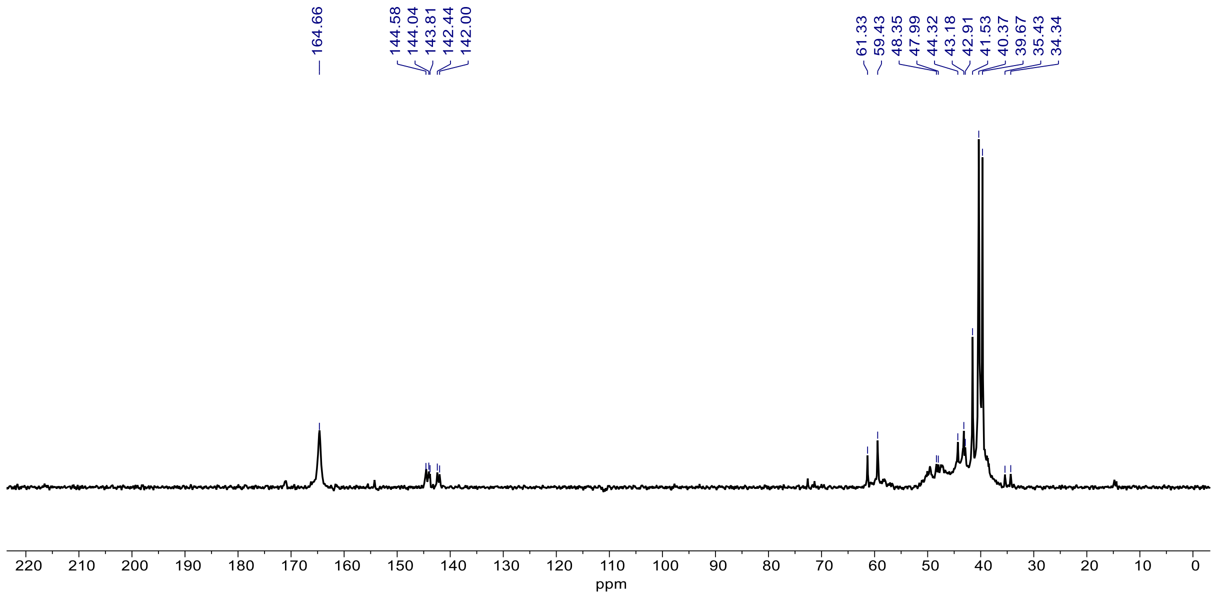

Supplementary Figure 54 $^{13}C\{^{1}H\}$ NMR (126 MHz, D_2O, l.b. 5 Hz) spectrum corresponding to product of Supplementary Table 1; Entry 16.

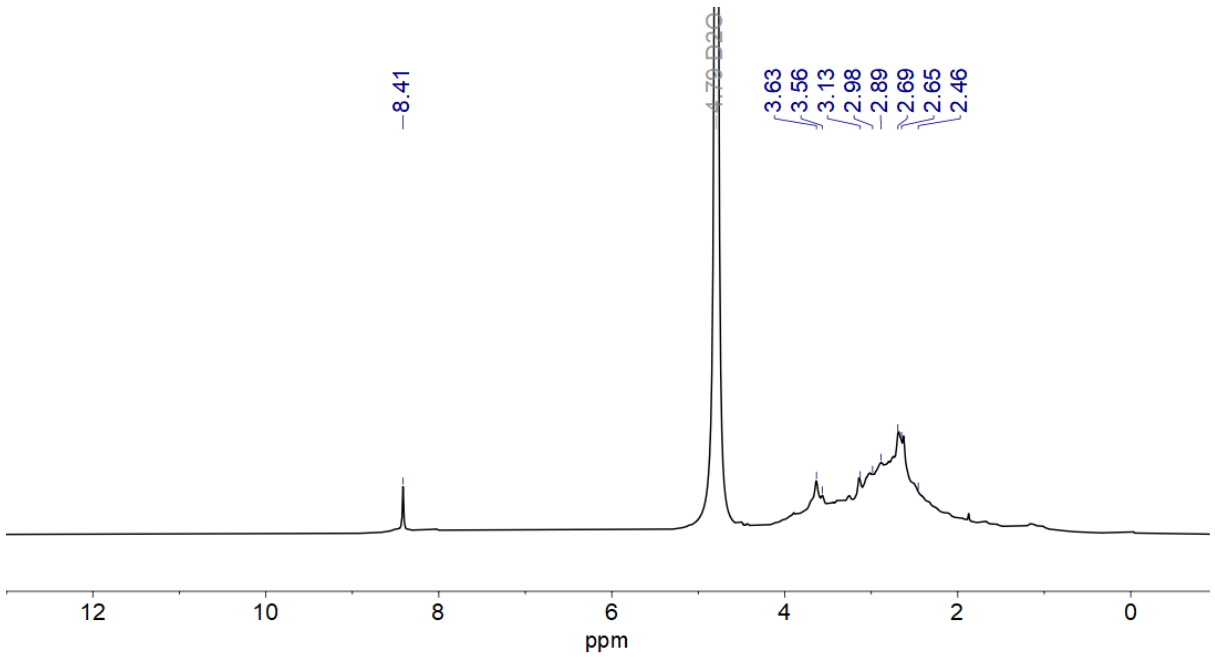

Supplementary Figure 55 ^{1}H NMR (500 MHz, D_2O) spectrum of the product corresponding to Supplementary Table 1; Entry 17.

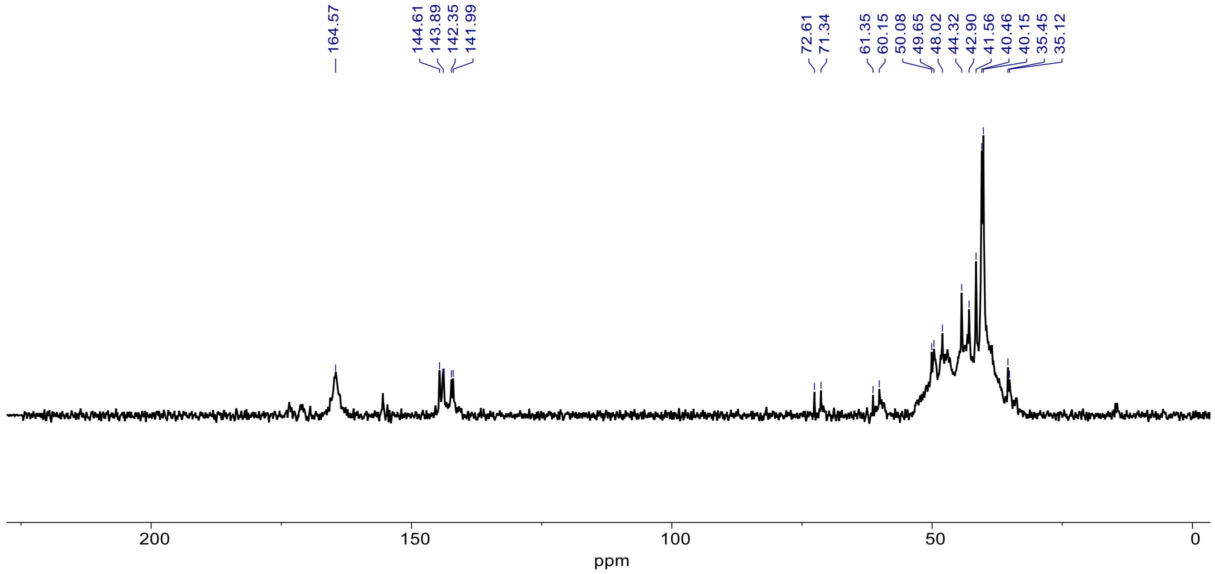

Supplementary Figure 56 $^{13}C\{^{1}H\}$ NMR (126 MHz, D_2O, l.b. 8 Hz) spectrum corresponding to product of Supplementary Table 1; Entry 17.

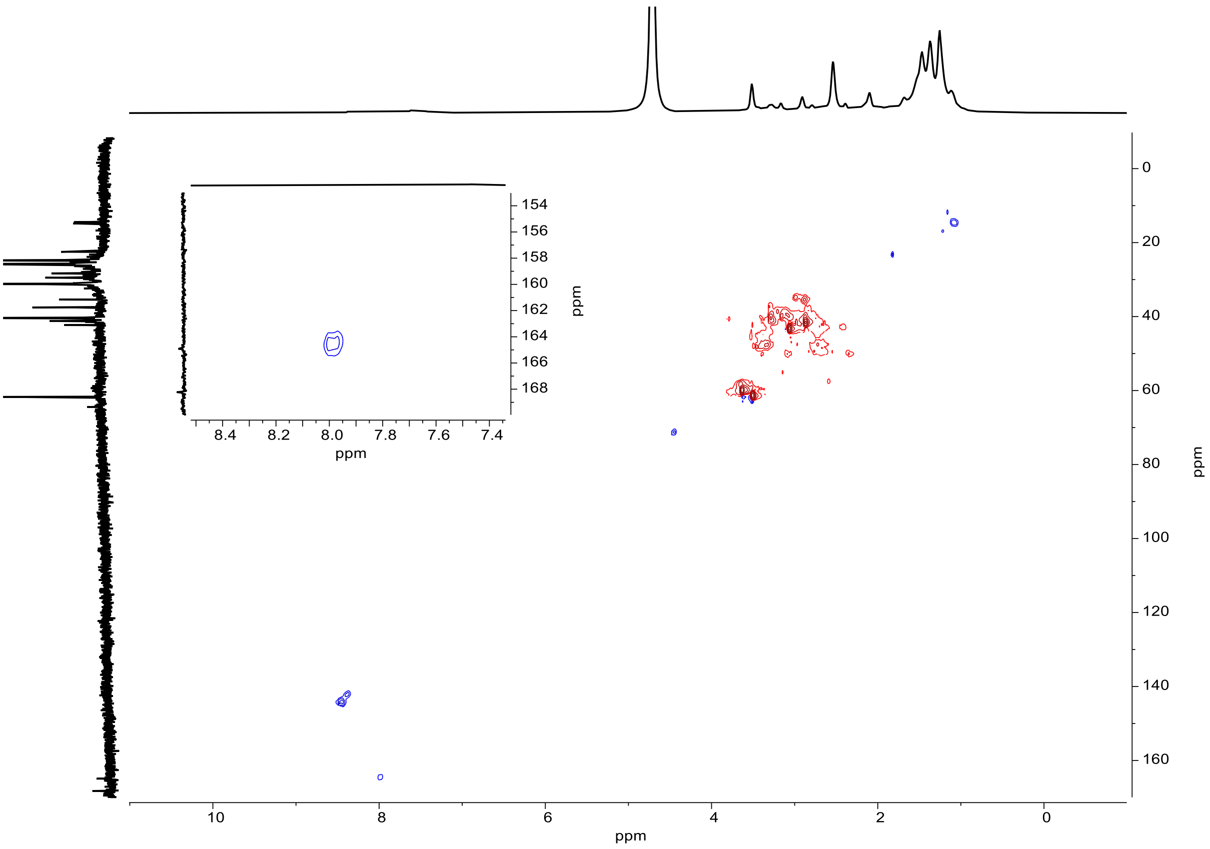

Supplementary Figure 57 Example of an HSQC cross correlation signal showing ^1J coupling between signals with δ_C 164 ppm and δ_H 7.9 ppm corresponding to C(H)=N.

1.3.4 TGA data

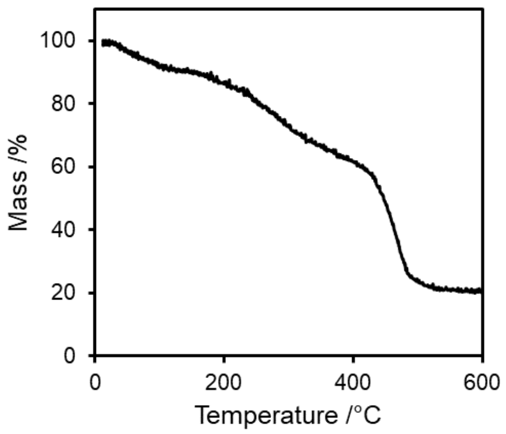

Supplementary Figure 58 TGA curve obtained for product corresponding to Supplementary Table 1; Entry 1.

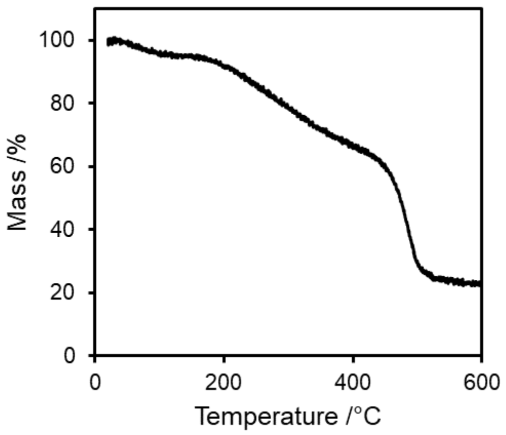

Supplementary Figure 59 TGA curve obtained for product corresponding to Supplementary Table 1; Entry 2.

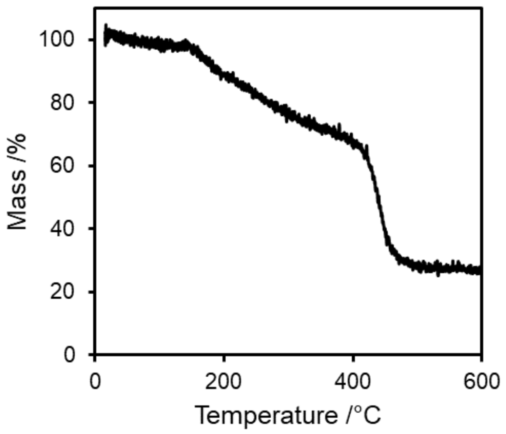

Supplementary Figure 60 TGA curve obtained for product corresponding to Supplementary Table 1; Entry 3.

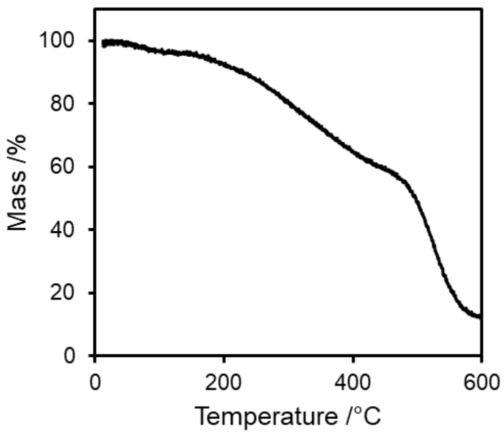

Supplementary Figure 61 TGA curve obtained for product corresponding to Supplementary Table 1; Entry 5.

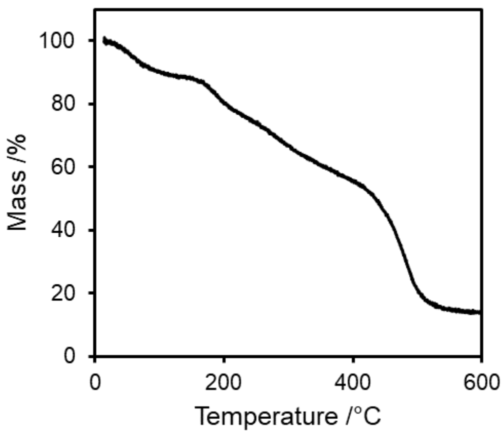

Supplementary Figure 62 TGA curve obtained for product corresponding to Supplementary Table 1; Entry 6.

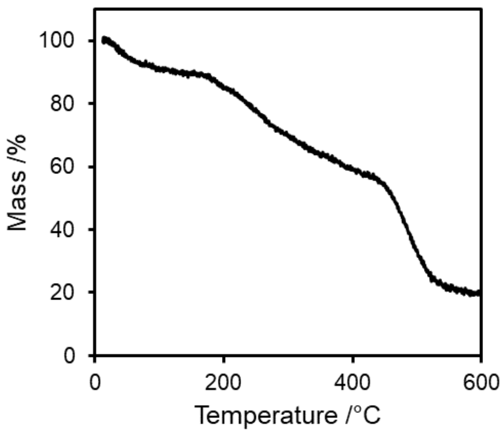

Supplementary Figure 63 TGA curve obtained for product corresponding to Supplementary Table 1; Entry 8.

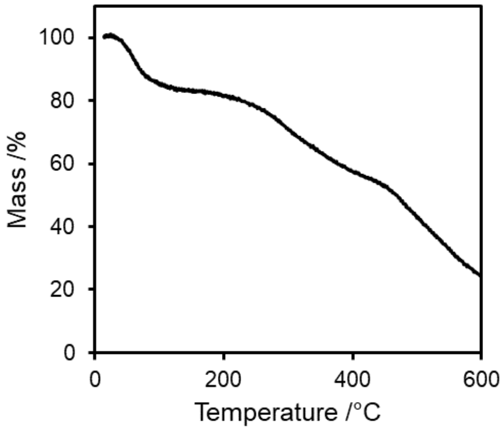

Supplementary Figure 64 TGA curve obtained for product corresponding to Supplementary Table 1; Entry 11.

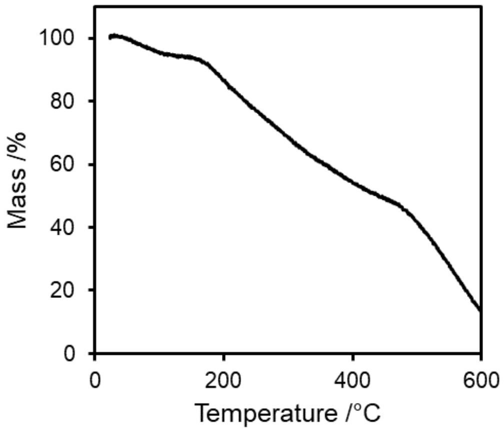

Supplementary Figure 65 TGA curve obtained for product corresponding to Supplementary Table 1; Entry 12.

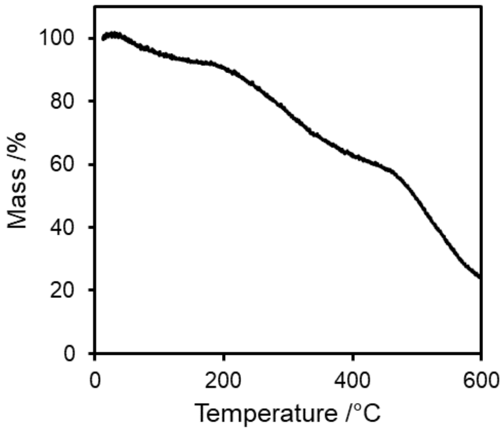

Supplementary Figure 66 TGA curve obtained for product corresponding to Supplementary Table 1; Entry 13.

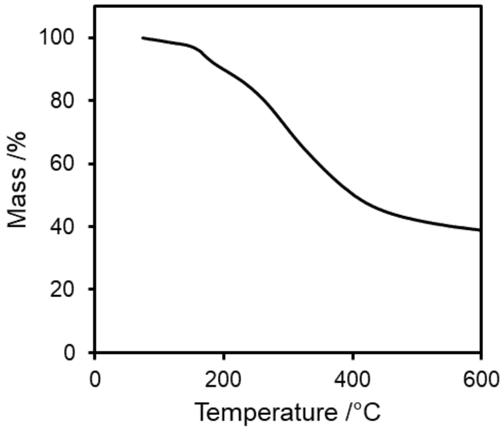

Supplementary Figure 67 TGA curve obtained for product corresponding to Supplementary Table 1; Entry 16.

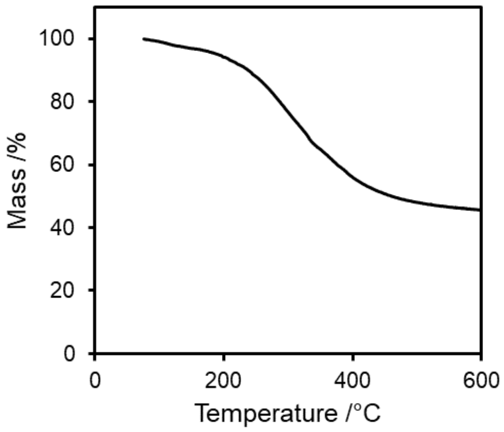

Supplementary Figure 68 TGA curve obtained for product corresponding to Supplementary Table 1; Entry 17.

To probe the mass loss at low temperatures (<200 °C), Supplementary Table 1, Entry 11 was subjected to TGA-MS analysis. Whilst this shows a similar loss in mass from 50 °C, with minor peaks in the DSC, an insufficient ion count was detected for the loss in material (Supplementary Figure 68). Comparison of this with a pure ethanolamine sample (Supplementary Figure 69) however showed a comparable loss in the TGA at low temperatures. The MS at this region, even with a neat sample, only shows as a baseline mass peak of 30 gmol^{-1} detected (the major fragmentation peak for ethanolamine) (Supplementary Figure 70). This occurs significantly below the boiling point for ethanolamine (*c.f.* 170 °C), allowing us to propose that the initial drop off visible in our TGA traces is due to the loss of starting material, however due to the poor detection of ethanolamine by MS, it is not possible to definitively show by TGA-MS.

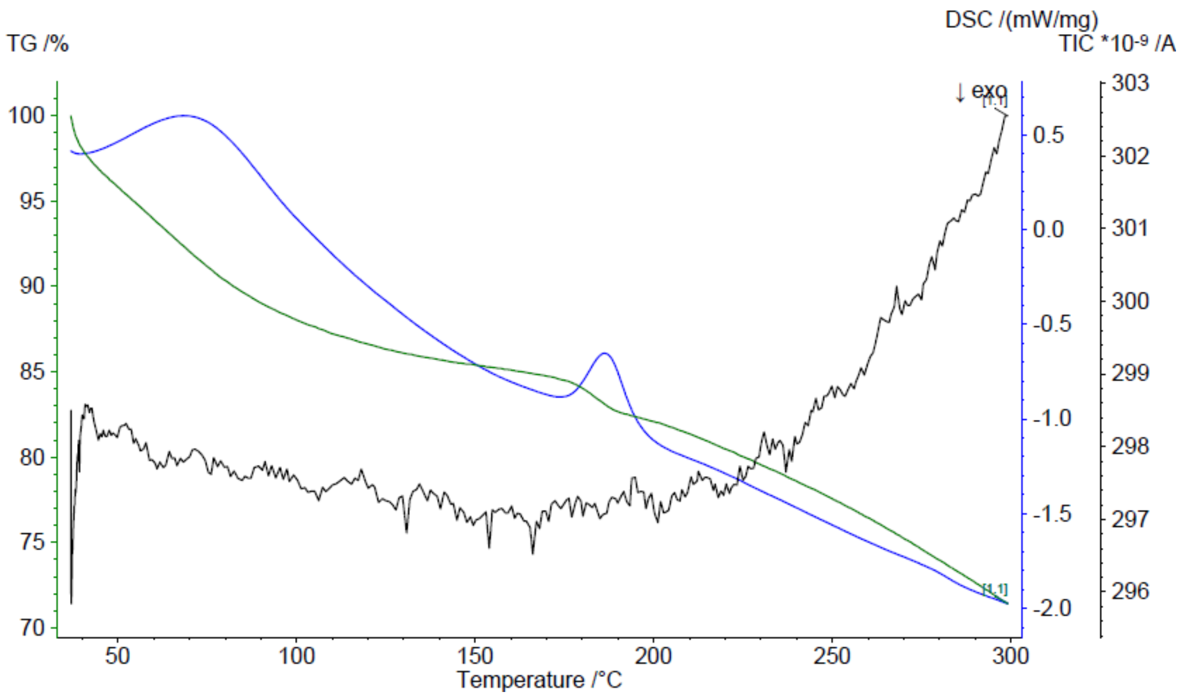

Supplementary Figure 69 TGA-MS trace obtained from Table 1, Entry 11 polymer with TGA (green), DSC (blue) and mass spectrometer ion count (black).

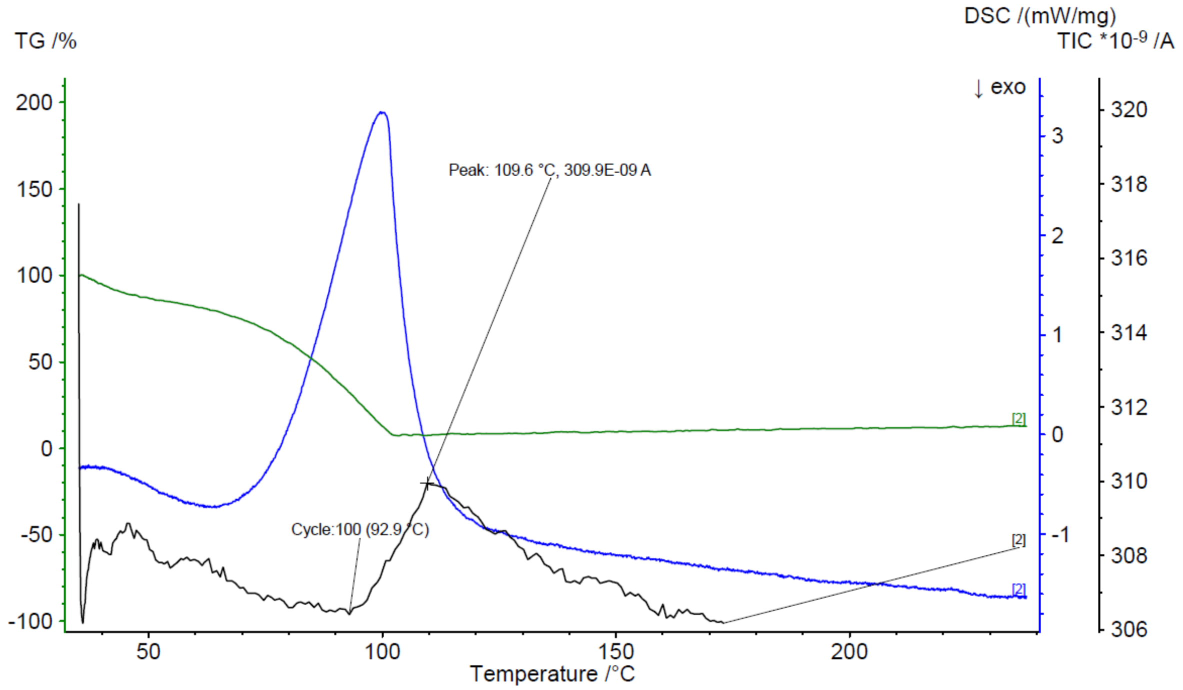

Supplementary Figure 70 TGA-MS trace obtained from ethanolamine with TGA (green), DSC (blue) and mass spectrometer ion count (black).

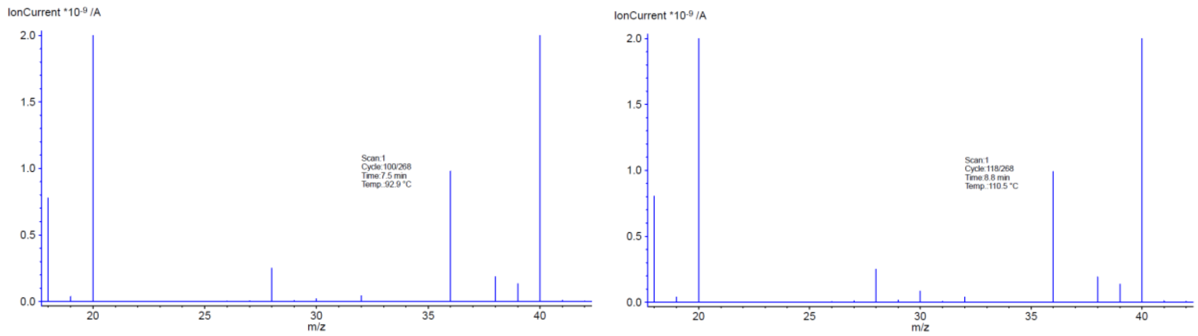

Supplementary Figure 71 Mass spectra obtained through TGA-MS of ethanolamine, showing the baseline (left) and maximum ion peak (right), with the 30 m/z signal increase corresponding to ethanolamine. No peak at 61 m/z observed for the M^+ peak.

1.3.5 DSC Data

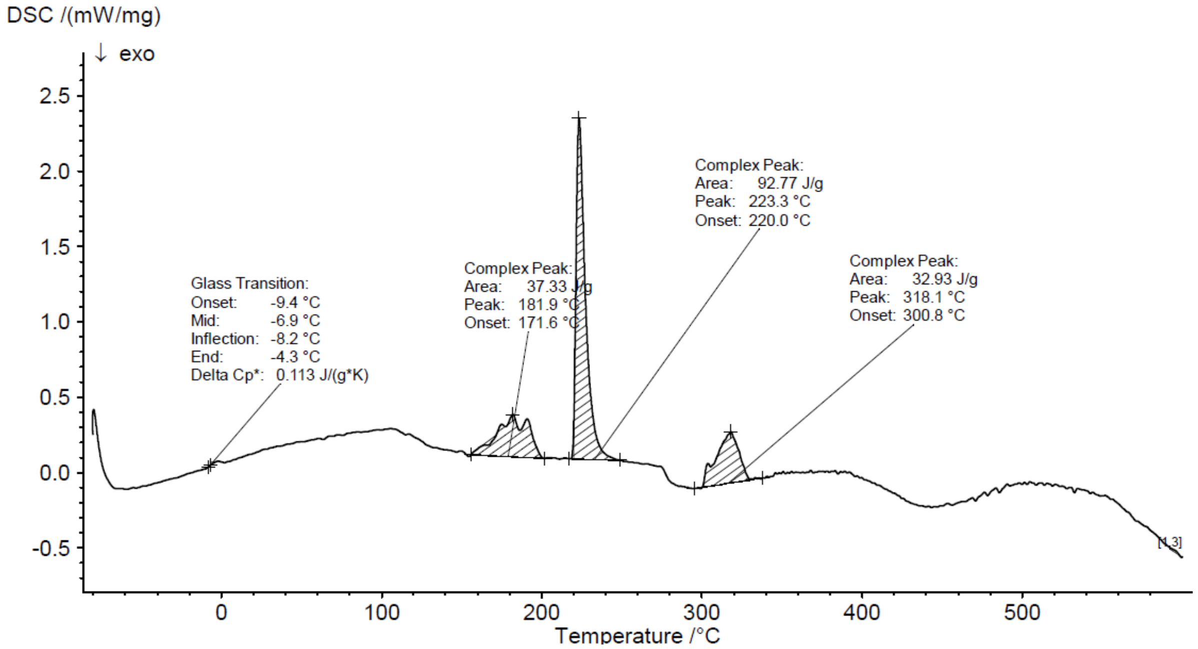

Supplementary Figure 72 DSC heatflow *vs* temperature corresponding to product of Supplementary Table 1; Entry 1. Peak at 181.9 °C is likely caused by residual ethanolamine.

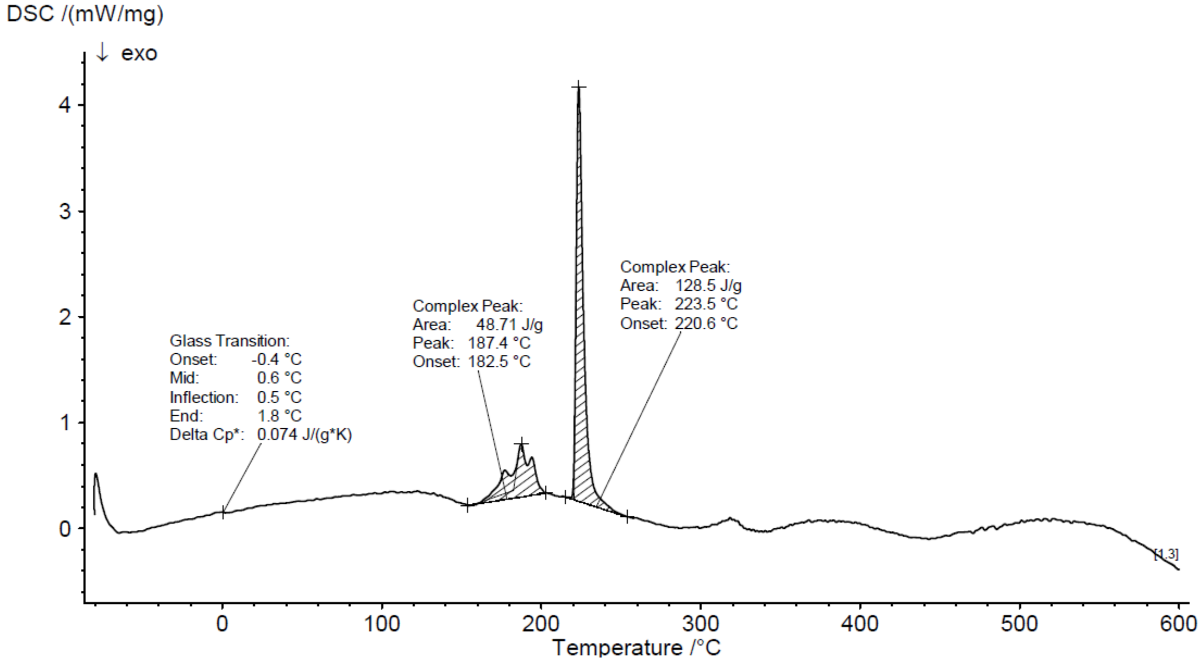

Supplementary Figure 73 DSC heatflow *vs* temperature corresponding to product of Supplementary Table 1; Entry 2. Peak at 187.4 °C is likely caused by residual ethanolamine.

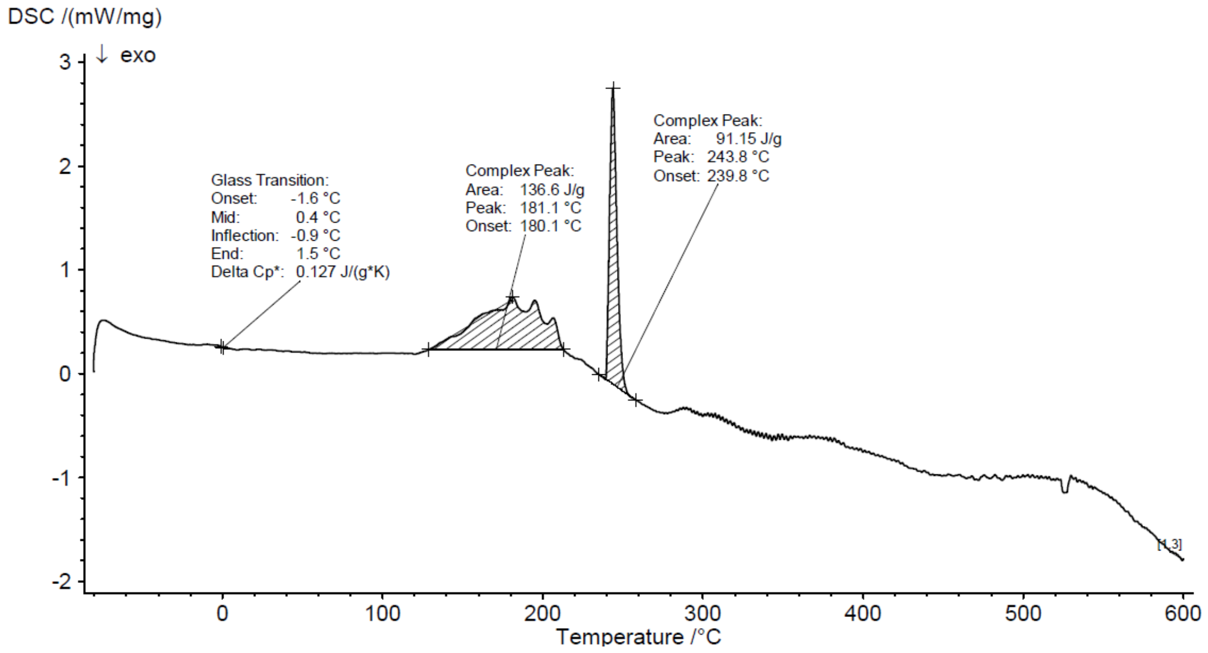

Supplementary Figure 74 DSC heatflow *vs* temperature corresponding to product of Supplementary Table 1; Entry 3. Peak at 181.1 °C is likely caused by residual ethanolamine.

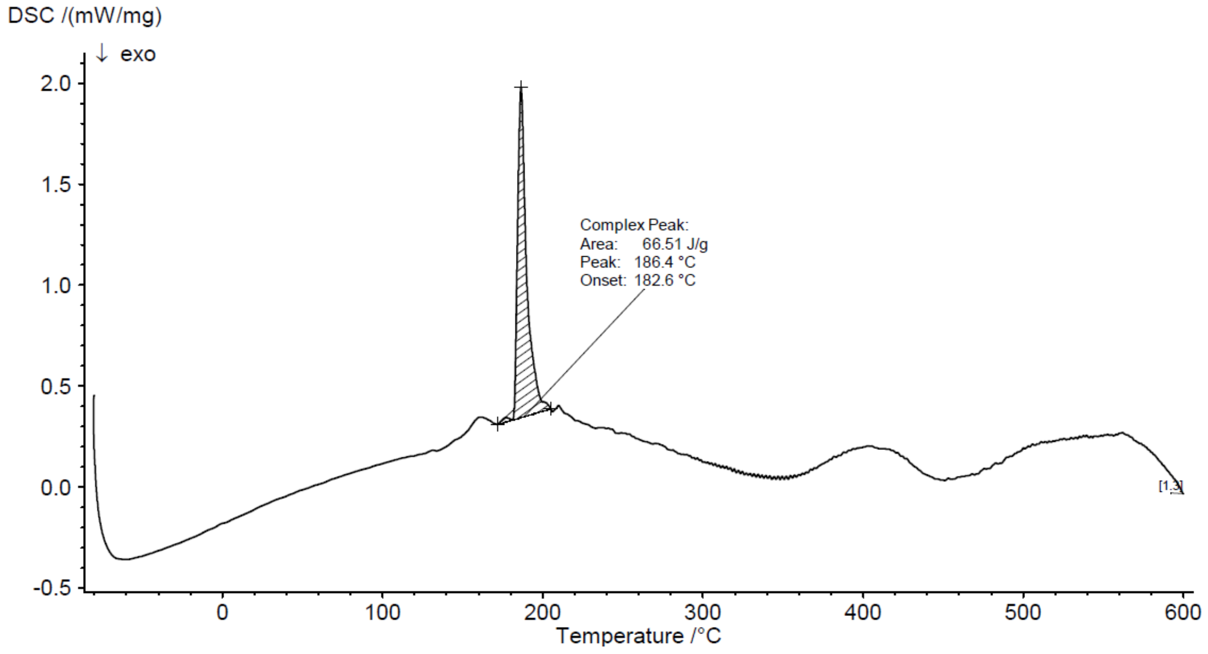

Supplementary Figure 75 DSC heatflow *vs* temperature corresponding to product of Supplementary Table 1; Entry 5.

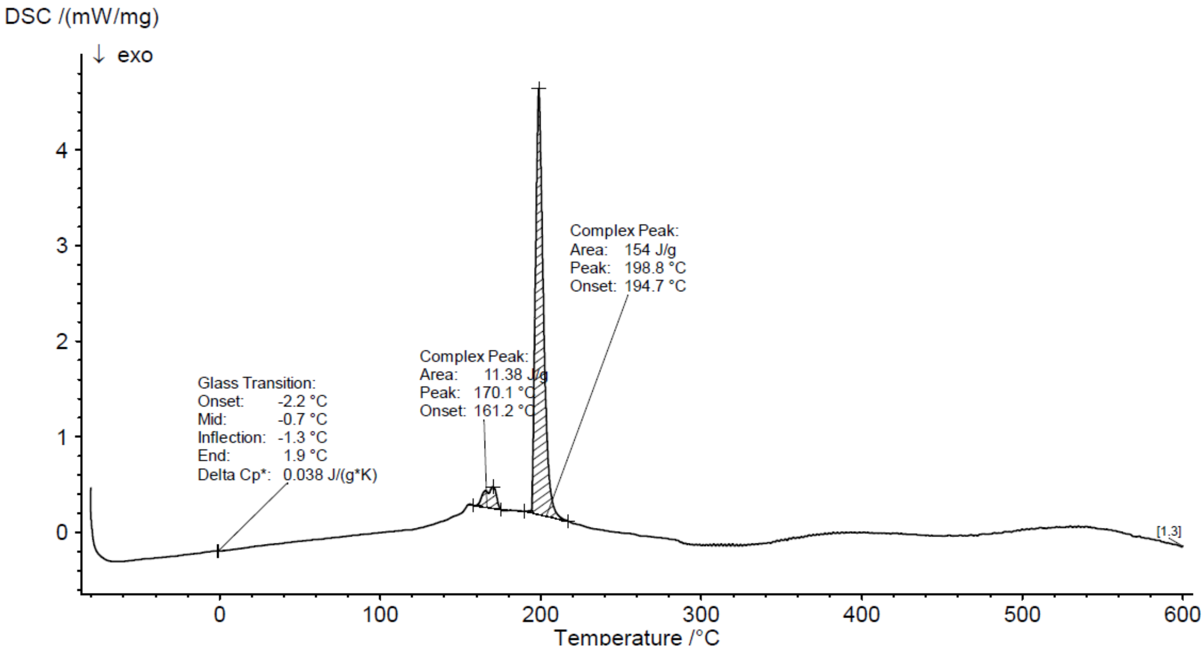

Supplementary Figure 76 DSC heatflow *vs* temperature corresponding to product of Supplementary Table 1; Entry 6. Peak at 170.1 °C is likely caused by residual ethanolamine.

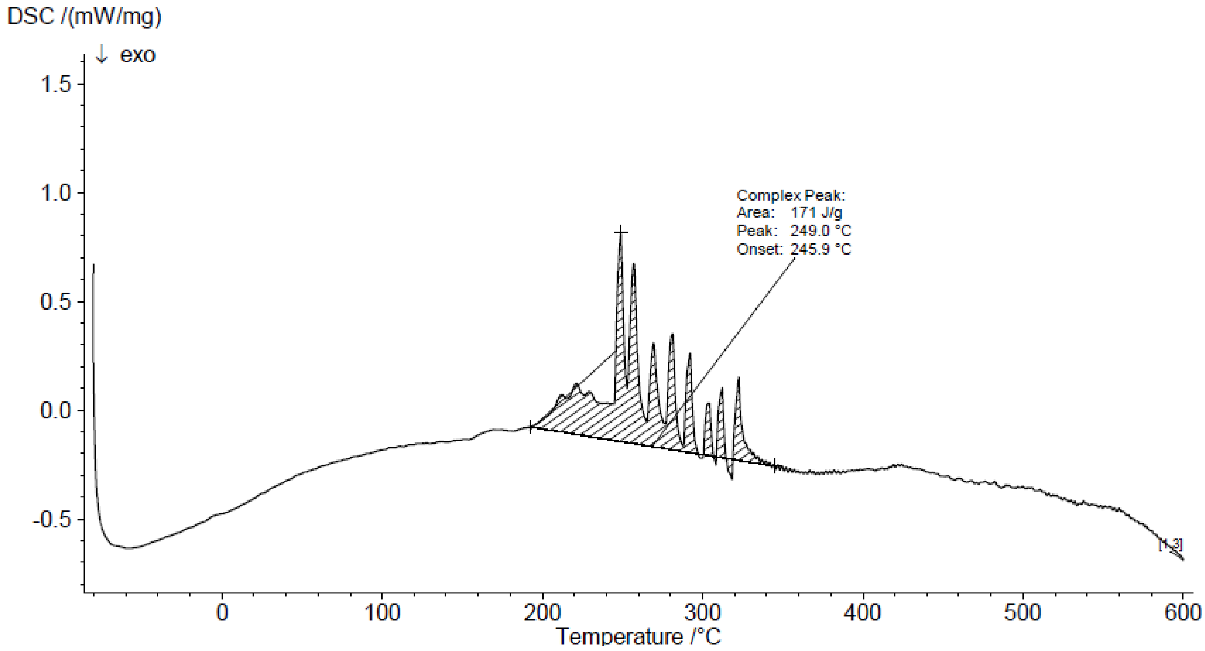

Supplementary Figure 77 DSC heatflow *vs* temperature corresponding to product of Supplementary Table 1; Entry 7.

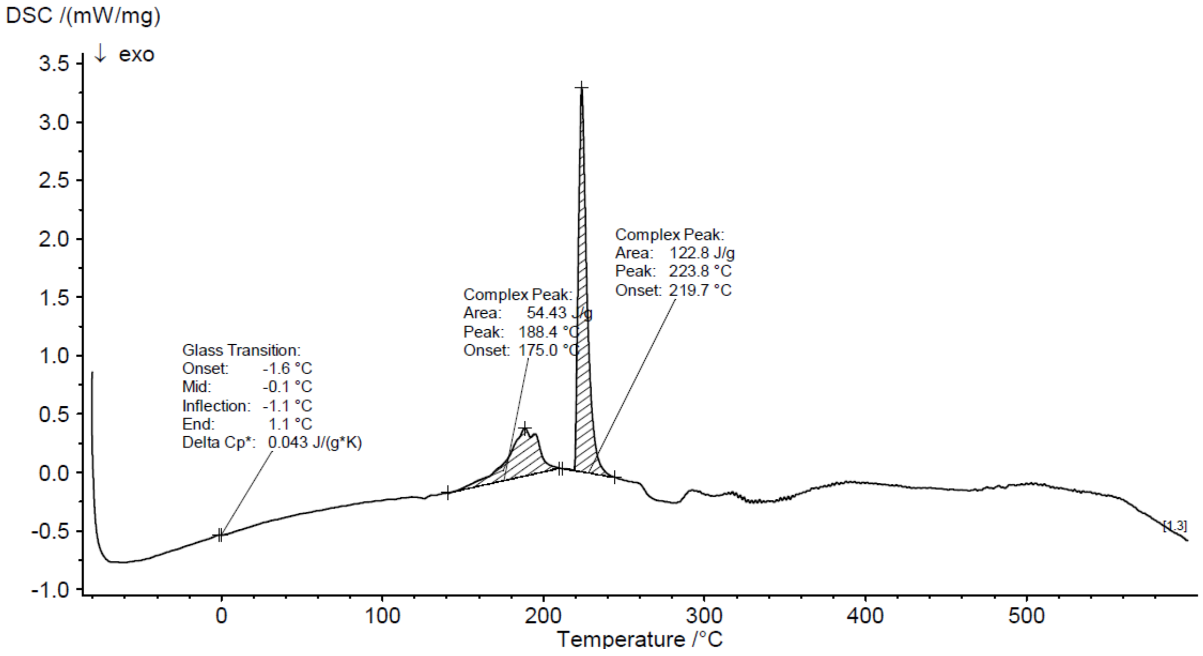

Supplementary Figure 78 DSC heatflow *vs* temperature corresponding to product of Supplementary Table 1; Entry 8. Peak at 188.4 °C is likely caused by residual ethanolamine.

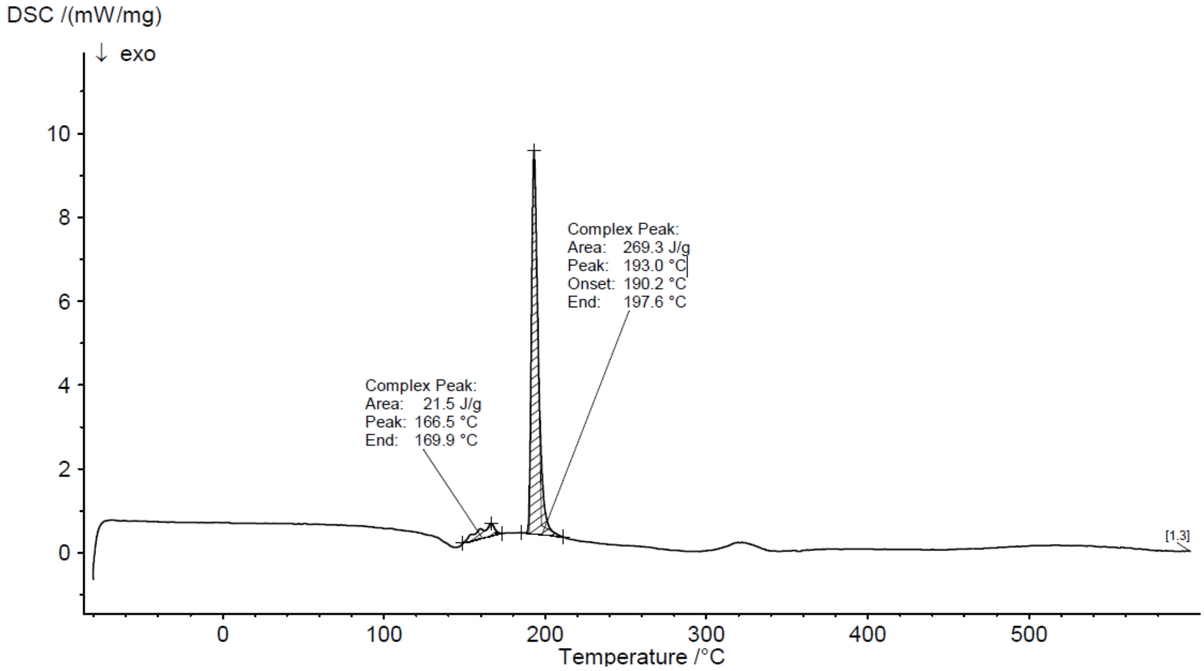

Supplementary Figure 79 DSC heatflow *vs* temperature corresponding to product of Supplementary Table 1; Entry 11.

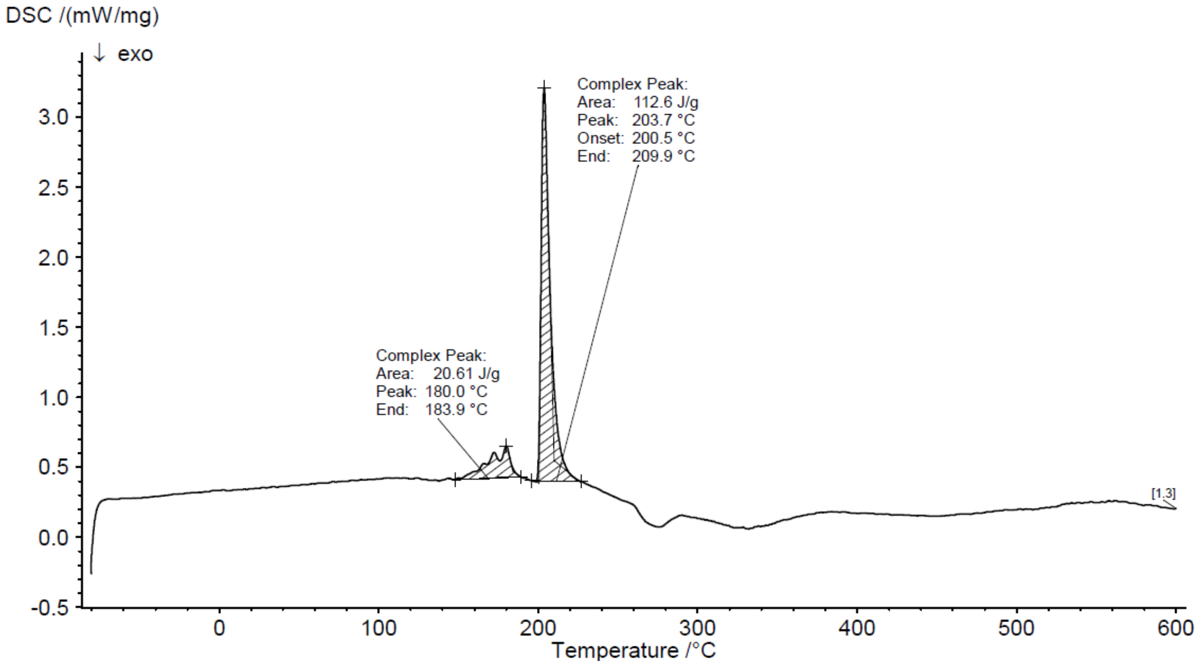

Supplementary Figure 80 DSC heatflow *vs* temperature corresponding to product of Supplementary Table 1; Entry 13. Peak at 180.0 °C is likely caused by residual ethanolamine.

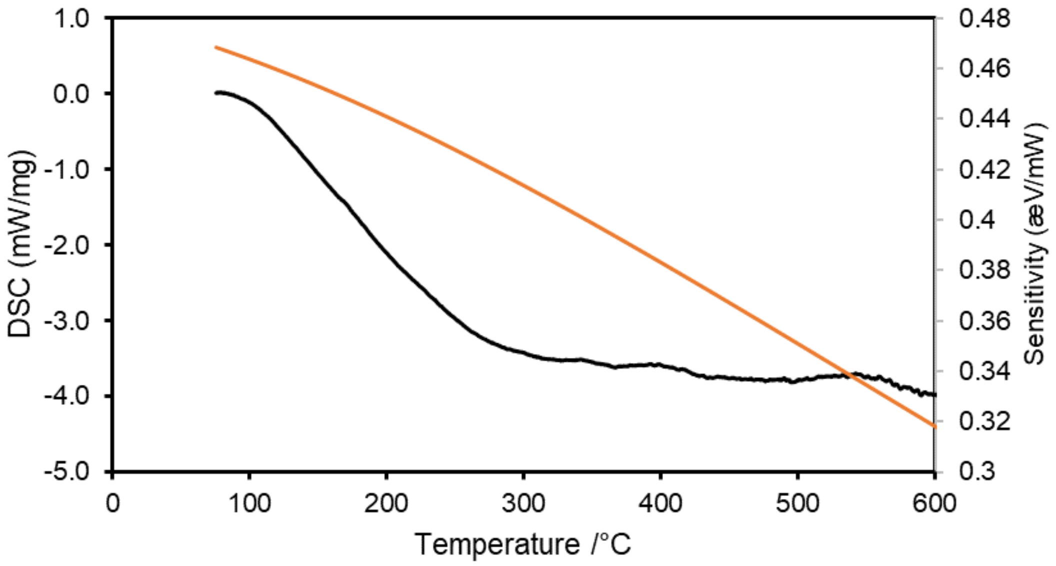

Supplementary Figure 81 DSC heatflow *vs* temperature corresponding to product of Supplementary Table 1; Entry 16.

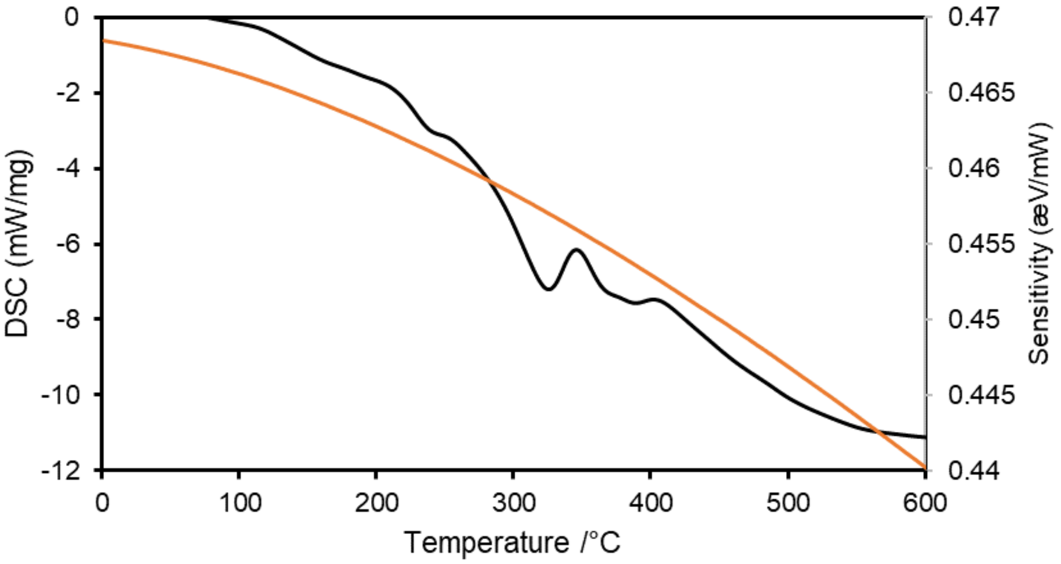

Supplementary Figure 82 DSC heatflow *vs* temperature corresponding to product of Supplementary Table 1; Entry 17.

1.3.6 HRMS (EI) spectra

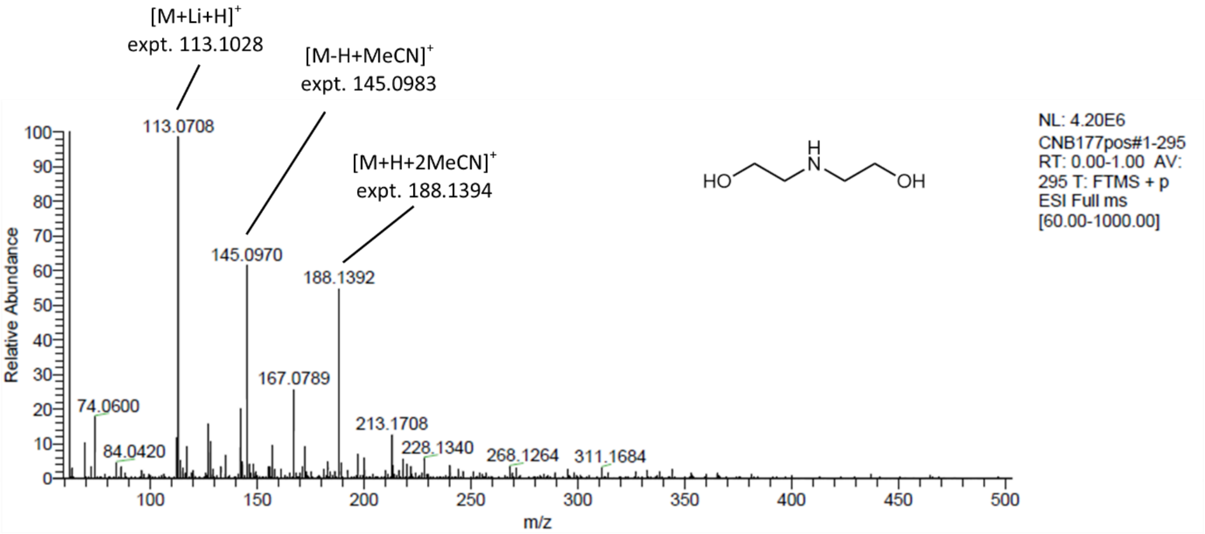

Supplementary Figure 83 HRMS (EI, MeCN) spectrum for diethanolamine produced, here corresponding to Supplementary Table 1; Entry 9.

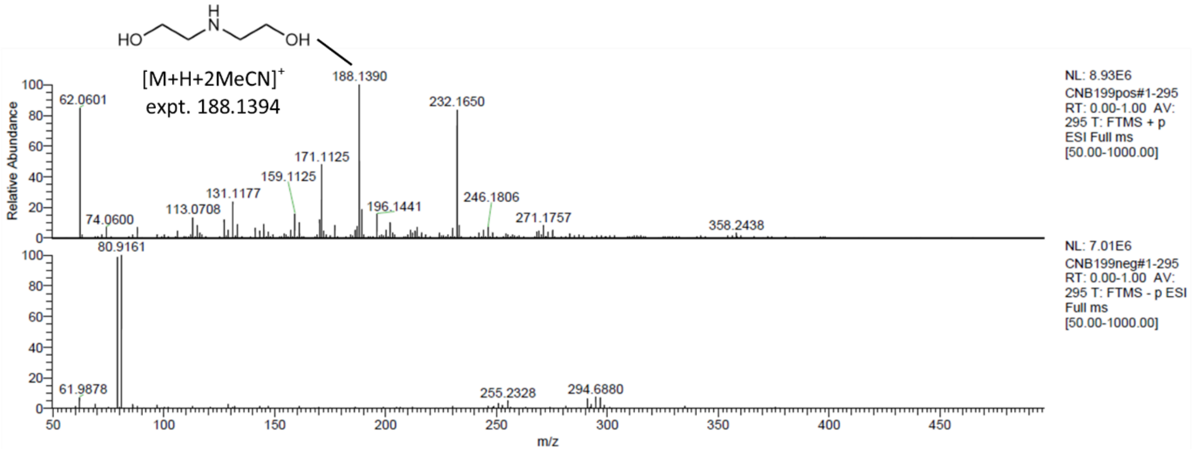

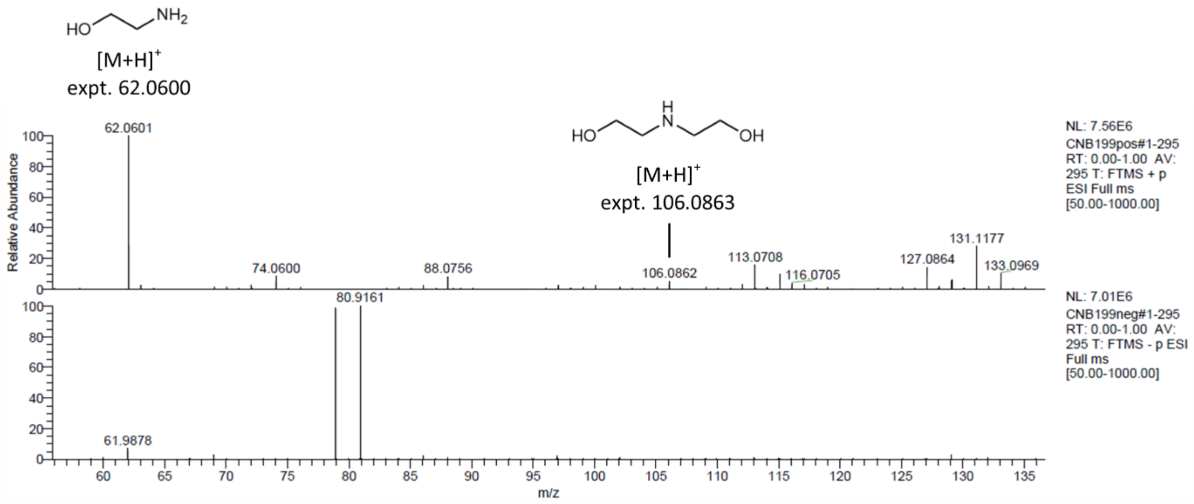

Supplementary Figure 84 HRMS (EI, MeCN) spectrum for diethanolamine produced, here corresponding to Supplementary Table 1; Entry 15.

1.3.7 Powder XRD

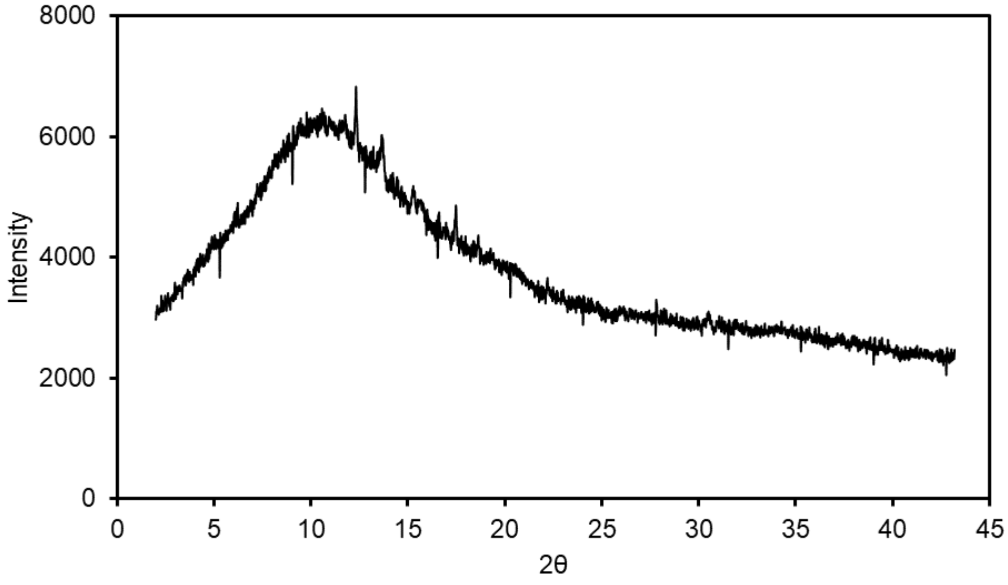

Supplementary Figure 85 p-XRD pattern obtained for a typical sample of **PEI-1**, here corresponding to Supplementary Table 1; Entry 11.

1.4 Ethanolamine Polymerisation Reaction Progression Monitoring

Supplementary Table 2 Reaction progression monitoring of ethanolamine polymerisation catalysed by **Mn-1**.[a]

Entry	Time /h	Yield[b] /%	M_n[c] /g mol^{-1}	Đ[c]
1	0.5	36	28,900	1.44
2	1	46	26,000	1.31
3	2	54	33,600	1.25
4	4	63	31,000	1.28
5	8	69	32,900	1.20
6	14	65	31,600	1.20
7	18	83	40,000	1.17
8	24	81	33,100	1.25

[a] Average of duplicate runs, Reaction conditions: 2 mmol ethanolamine, [0.5 M] in toluene (4 mL), 1 mol% **Mn-1**, 150 °C in a sealed 100 cm^3 system. [b] isolated yield. [c] Determined by GPC analysis relative to PEG/PEO standards.

Entry 1:

^{1}H NMR (500 MHz, D_2O): δ_H 8.42 (s, CH=N), 3.63 (br s), 3.57 (br s), 3.11 (br s), 2.71 (br s).

^{13}C{^{1}H} NMR (126 MHz, D_2O): δ_C 161.9 (C=N), 62.7, 61.3, 60.1, 49.7, 43.1, 42.2.

IR (ATR-FTIR, cm^{-1}): ν 3285s (O-H/N-H), 2932m (C-H), 2842m (C-H), 1641w (C=N), 1578s (N-H), 1437s (C-H), 1350s (O-H), 1059s (C-O).

Entry 2:

^{1}H NMR (500 MHz, D_2O): δ_H 8.42 (s, CH=N), 3.64 (s), 3.60 (br s), 3.59 (br s), 3.34 (br s), 3.12 (br s), 2.75 (s), 2.70 (br s).

^{13}C{^{1}H} NMR (126 MHz, D_2O): δ_C 171.0 (C=N), 166.2 (C=N), 164.8 (C=N), 62.1, 61.3, 60.1, 49.7, 43.1, 42.1.

IR (ATR-FTIR, cm^{-1}): ν 3269m (O-H/N-H), 2940w (C-H), 2843w (C-H), 1649w (C=N), 1578s (N-H), 1348s (O-H), 1057m (C-O).

Entry 3:

^{1}H NMR (500 MHz, D_2O): δ_H 8.42 (s, CH=N), 3.64 (br s), 3.60 (s), 3.57 (br s), 3.12 (s), 2.75 (br s), 2.71 (br s).

^{13}C{^{1}H} NMR (126 MHz, D_2O): δ_C 171.0 (C=N), 166.1 (C=N), 62.2, 61.3, 60.1, 49.7, 46.5, 43.1, 42.1.

IR (ATR-FTIR, cm^{-1}): ν 3260m (O-H/N-H), 2936w (C-H), 2843w (C-H), 1579s (N-H), 1449s (C-H), 1371s (O-H), 1059m (C-O).

Entry 4:

^{1}H NMR (500 MHz, D_2O): δ_H 8.42 (s, CH=N), 3.64 (s), 3.56 (br s), 3.15 (s), 2.70 (br s), 2.69 (s), 2.61 (br s).

^{13}C{^{1}H} NMR (126 MHz, D_2O): δ_C 171.0 (C=N), 63.1, 60.2, 47.7, 47.4, 42.2.

IR (ATR-FTIR, cm^{-1}): ν 3225m (O-H/N-H), 2936w (C-H), 2855w (C-H), 1585s (N-H), 1449s (C-H), 1367s (O-H), 1059m (C-O).

Entry 5:

^{1}H NMR (500 MHz, D_2O): δ_H 8.41 (s, CH=N), 3.63 *s), 3.55 (br s), 3.11 (br s), 2.67 (br s).

^{13}C{^{1}H} NMR (126 MHz, D_2O): δ_C 171.0 (C=N), 168.3 (C=N), 63.1, 61.3, 60.2, 52.0, 49.9, 47.4, 43.1, 42.2.

IR (ATR-FTIR, cm^{-1}): ν 3248m (O-H/N-H), 2930m (C-H), 2857m (C-H), 1578s (N-H), 1443s (C-H), 1350s (O-H), 1061s (C-O).

Entry 6:

^{1}H NMR (500 MHz, D_2O): δ_H 8.41 (s, CH=N), 3.63 (br s), 3.56 9br s), 3.12 (s), 2.68 (br s), 2.60 (br s).

^{13}C{^{1}H} NMR (126 MHz, D_2O): δ_C 171.0 (C=N), 168.3 (C=N), 63.1, 61.3, 60.2, 58.3, 49.7, 47.4, 42.2.

IR (ATR-FTIR, cm^{-1}): ν 2301m (O-H/N-H), 2947w (C-H), 2853w (C-H), 1580s (N-H), 1449s (C-H), 1369s (O-H), 1061m (C-O).

Entry 7:

^{1}H NMR (500 MHz, D_2O): δ_H 8.42 (s, CH=N), 3.64 (s), 3.60 (br s), 3.12 (br s), 2.73 (br s).

^{13}C{^{1}H} NMR (126 MHz, D_2O): δ_C 63.1, 62.5, 62.3, 61.3, 60.0, 49.8, 43.1, 42.1.

IR (ATR-FTIR, cm^{-1}): ν 3227m (O-H/N-H), 2936w (C-H), 2835w (C-H), 1582s (N-H), 1449s (C-H), 1368s (O-H), 1059m (C-O).

Entry 8:

^{1}H NMR (500 MHz, D_2O): δ_H 8.52 (s, CH=N), 8.42 (s, CH=N), 3.62 (br s), 3.13 (br s), 2.79 (br s).

^{13}C{^{1}H} NMR (126 MHz, D_2O): δ_C 61.8, 61.3, 60.0, 49.9, 49.6, 43.1, 42.0

IR (ATR-FTIR, cm^{-1}): ν 3248m (O-H/N-H), 2936w (C-H), 2843w (C-H), 1578s (N-H), 1458s (C-H), 1340s (O-H), 1057m (C-O).

1.4.1 IR Spectra

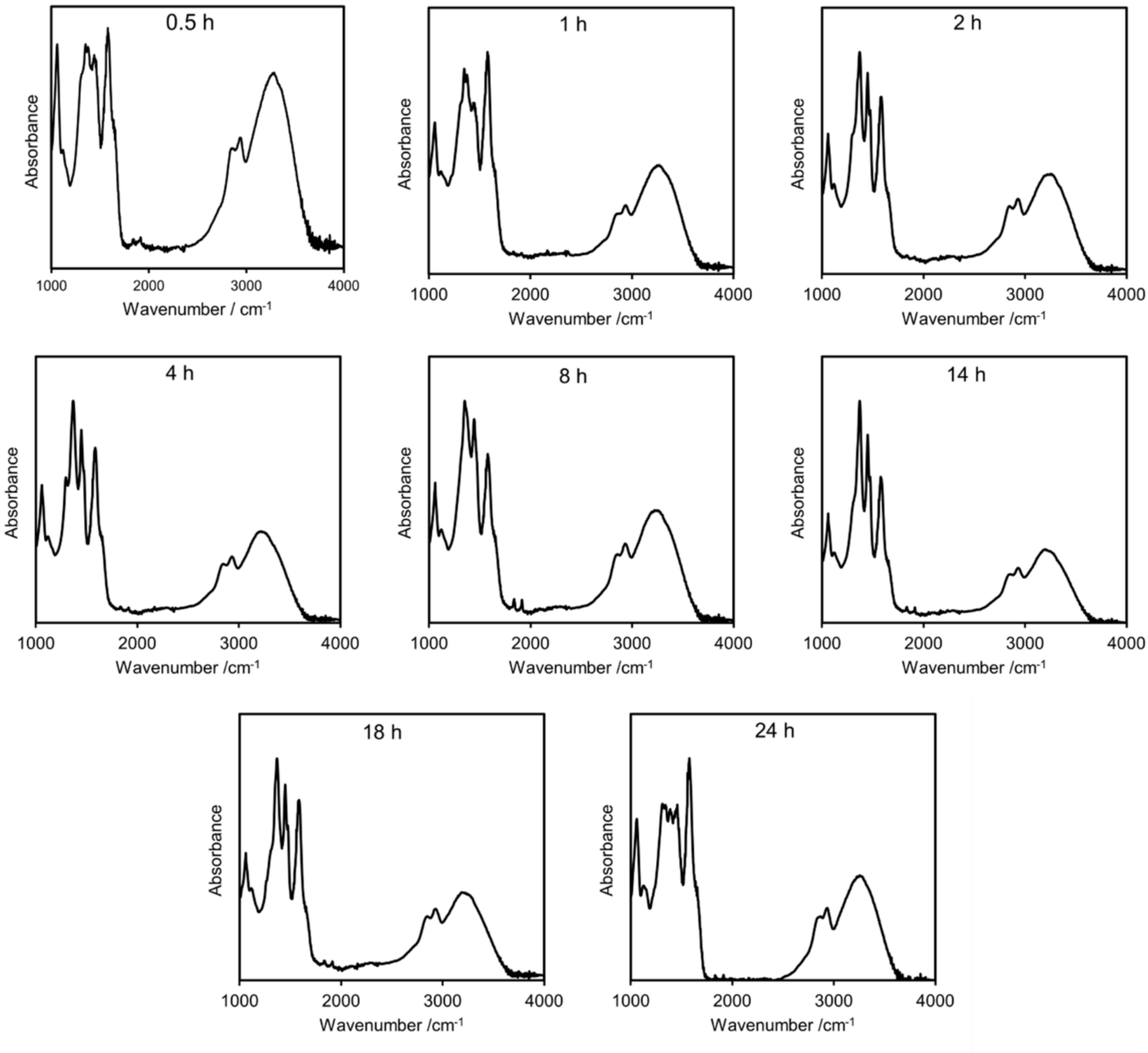

Supplementary Figure 86 IR (ATR-FTIR) spectra obtained of the products of ethanolamine polymerisation with reaction time varied.

1.4.2 GPC Data

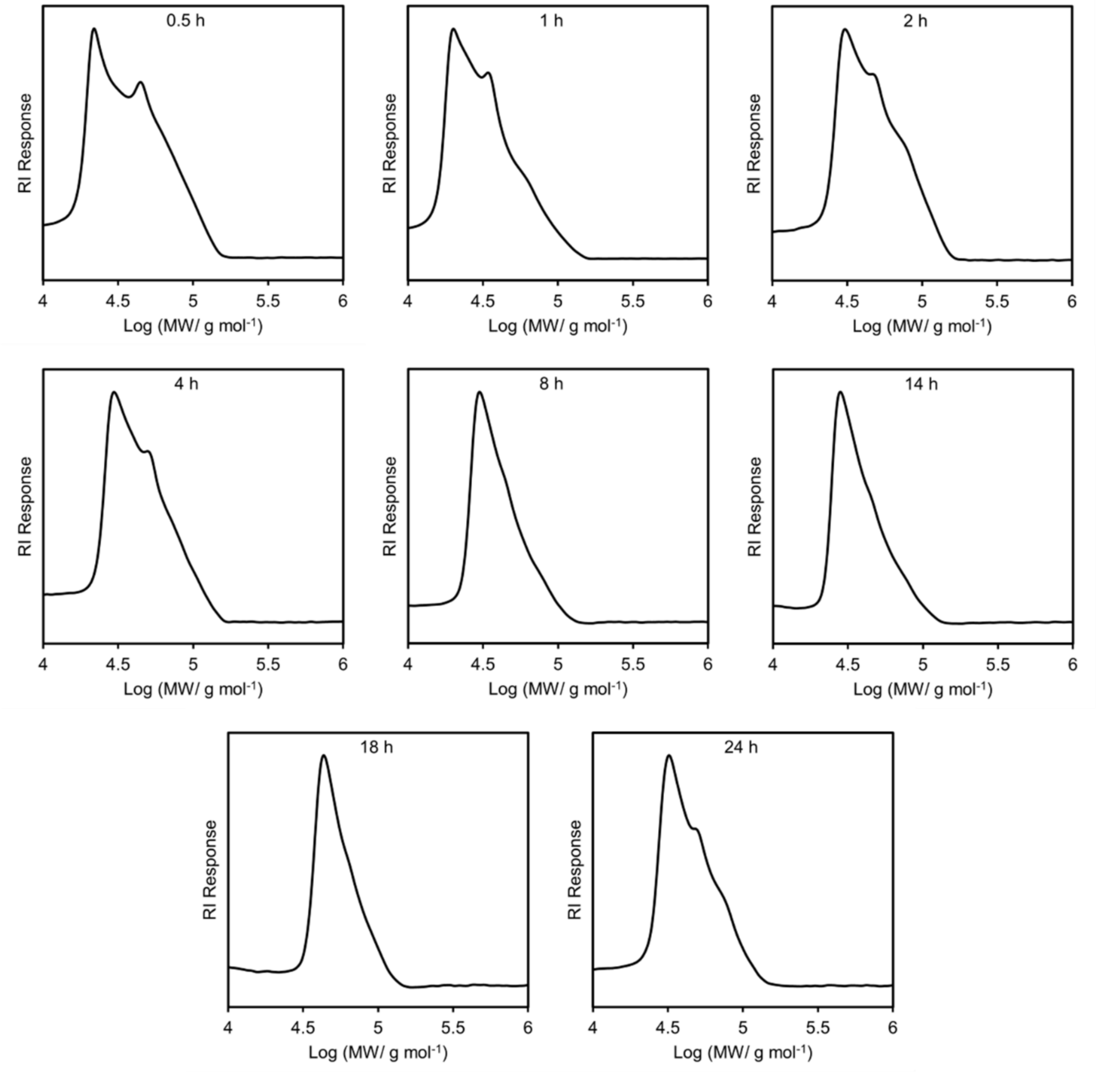

Supplementary Figure 87 GPC chromatographs of isolated product from varying degrees of substrate conversion from the polymerisation of ethanolamine.

1.4.3 NMR Spectra

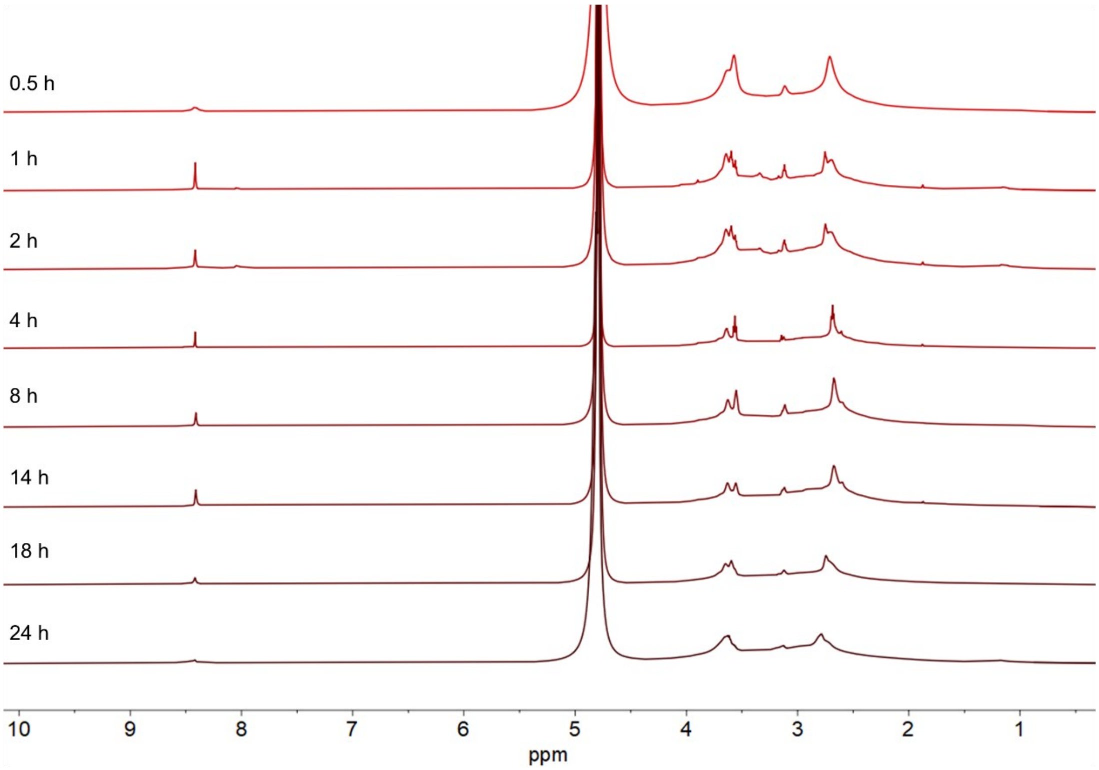

Supplementary Figure 88 ^{1}H NMR (500 MHz, D_2O) spectra of products of ethanolamine polymerisation with varying reaction times.

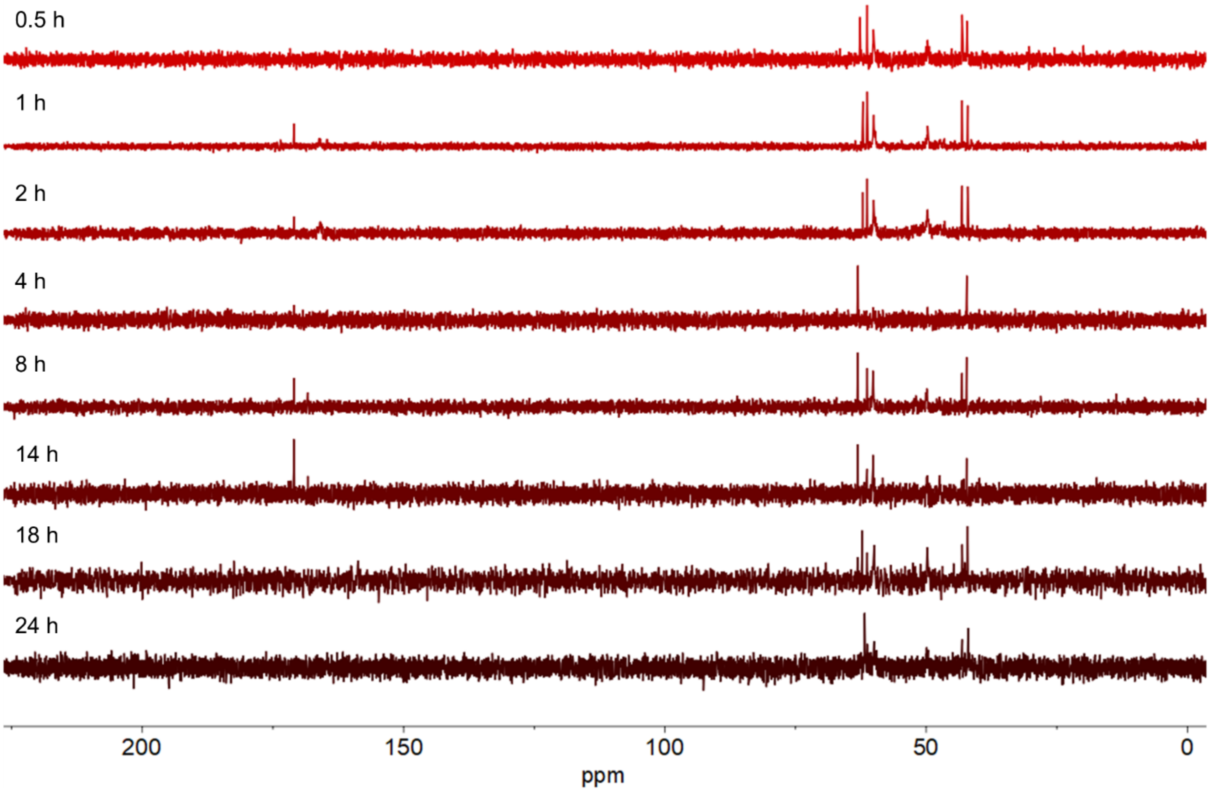

Supplementary Figure 89 $^{31}C\{^{1}H\}$ NMR (126 MHz, D_2O) spectra of products of ethanolamine polymerisation with varying reaction times.

1.5 Catalyst Recyclability

1.5.1 Catalyst recyclability without additional base

To determine the recyclability of the catalyst, a polymerisation was carried out under standard conditions in accordance with Supplementary Table 1, Entry 1. At the end of the reaction, the water soluble polymer (that is solubilised and phase-separates into the liberated water) is isolated *via* decantion and the toluene portion of the reaction mixture along with any toluene-soluble organics was transferred into an ampoule containing fresh ethanolamine (2 mmol, 0.12 mL). This ethanolamine containing reaction vessel was sealed under argon and heated to 150 °C for 24 hours. After this time, the reaction was allowed to cool to room temperature and the resulting product was extracted into distilled water (5 mL). Water and other volatile components were removed under reduced pressure at 110 °C to yield **PEI-1** (55 mg, 64 %).

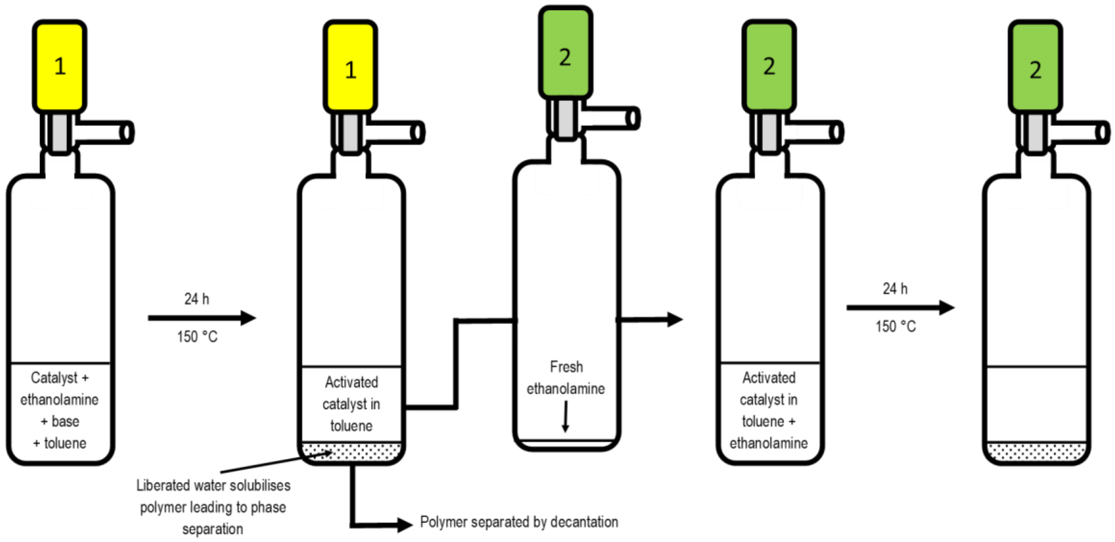

Supplementary Figure 90 Simple schematic showing how the catalyst recyclability test was performed.

^{1}H NMR (500 MHz, D_2O): δ_H 3.65 (br s), 3.59 (s), 2093 (br s), 2.72 (br s).

^{13}C{^{1}H} NMR (126 MHz, D_2O): δ_C 62.6, 60.0, 58.1, 49.6, 42.2.

IR (ATR-FTIR, cm^{-1}): ν 3285m, (O-H/N-H), 2928m (C-H), 2862m (C-H), 1638m (C=N), 1570m (N-H), 1449m (C-H), 1339m (O-H), 1053 (vs, C-O).

GPC (H_2O, 30 °C, g mol^{-1}): Mn >60,000 g mol^{-1}. Accurate M_n and dispersity could not be determined due to the size exclusion limitations of our GPC column set up.

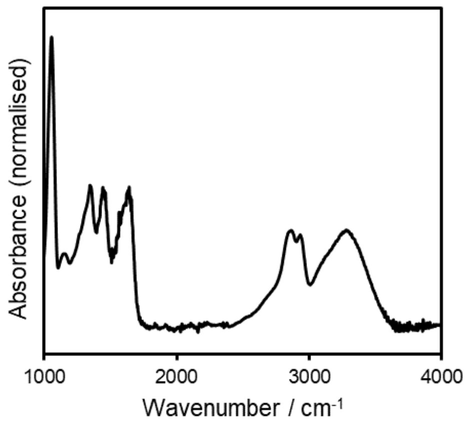

Supplementary Figure 91 (ATR-FTIR) spectrum of product of ethanolamine polymerisation using recycled catalyst.

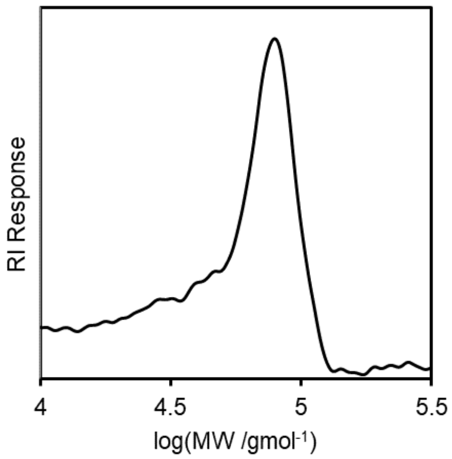

Supplementary Figure 92 Measured GPC data for the product of the polymerisation of ethanolamine using recycled catalyst.

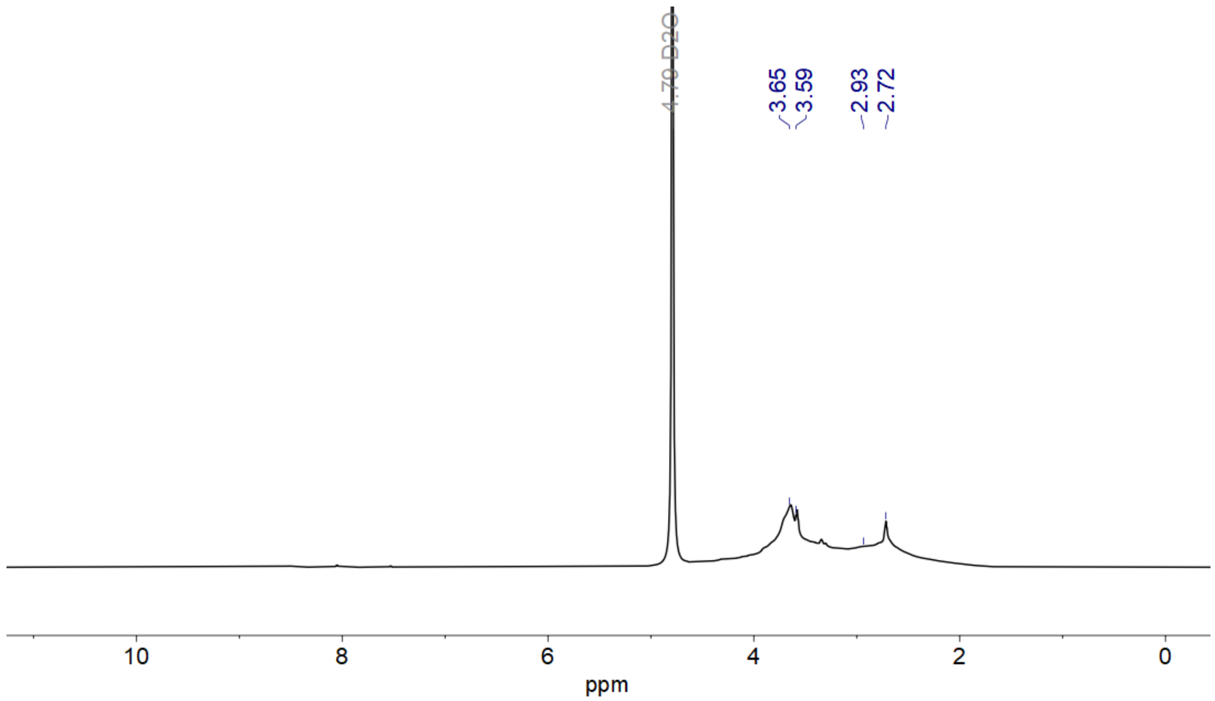

Supplementary Figure 93 ^{1}H NMR (500 MHz, D_2O) spectrum of product of the polymerisation of ethanolamine using recycled catalyst.

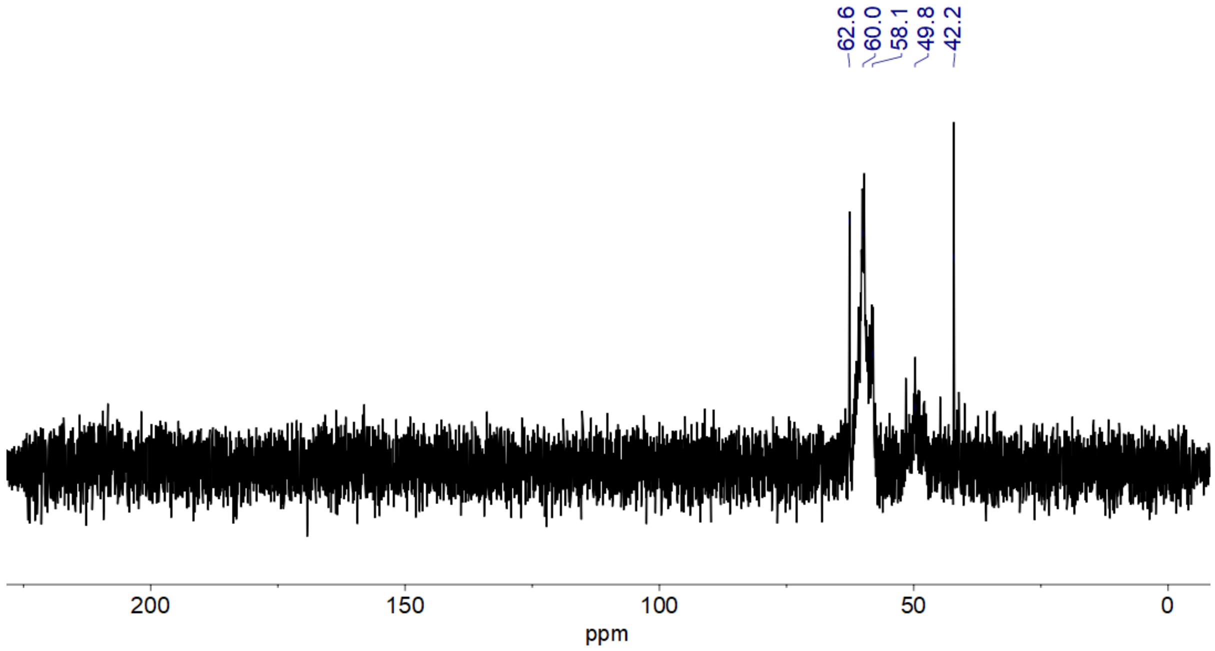

Supplementary Figure 94 $^{13}C\{^{1}H\}$ NMR (126 MHz, D_2O) spectrum of product of the polymerisation of ethanolamine using recycled catalyst.

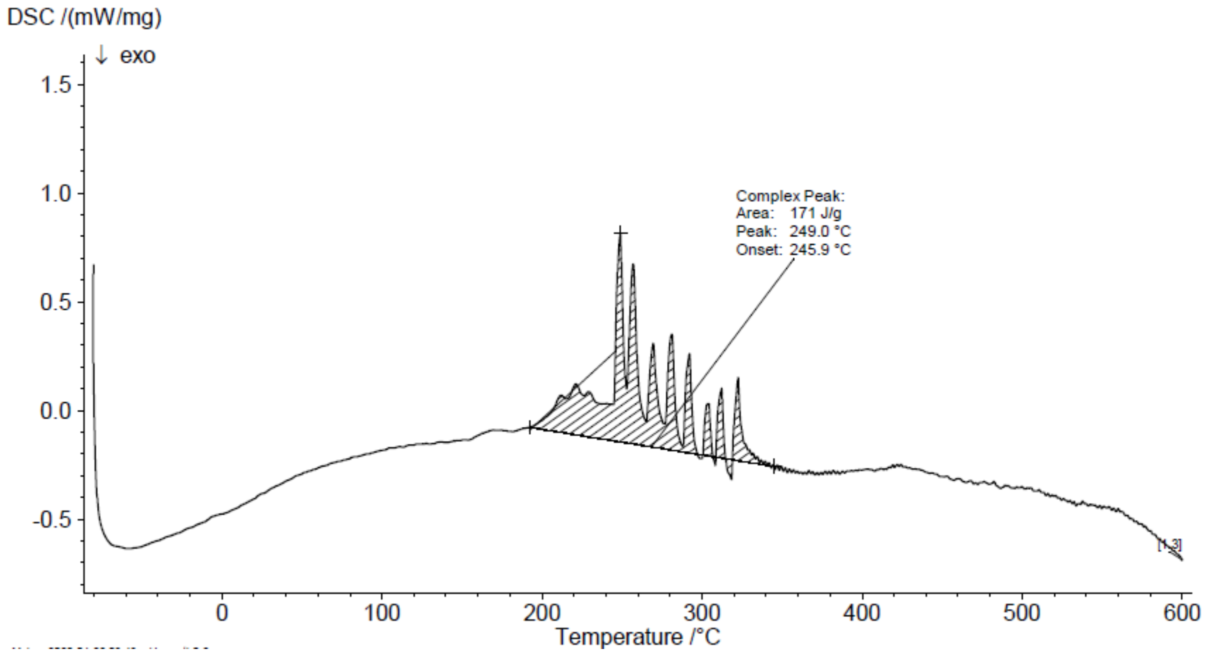

Supplementary Figure 95 DSC trace corresponding to polymer obtained using recycled catalyst.

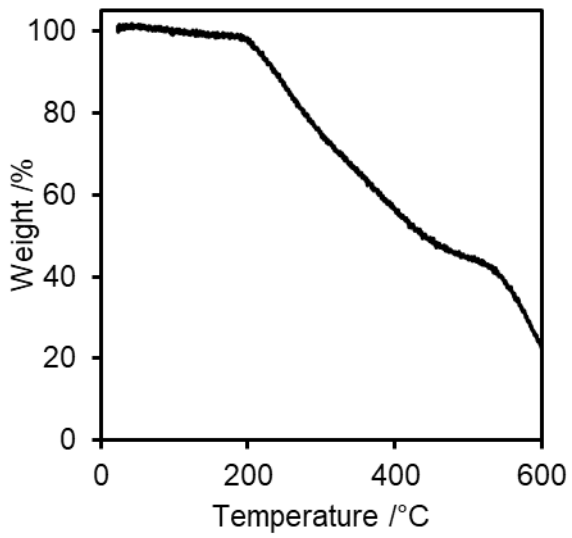

Supplementary Figure 96 TGA trace corresponding to polymer obtained using recycled catalyst.

1.5.2 Catalyst recyclability with additional base

To determine whether this increased degree of ethoxylation was the result of decreased base loading in the recycled catalyst mixture compared to initial run, a second experiment was conducted in accordance with the above, but the new batch of substrate (0.12 mL, 2 mmol) was combined with the toluene soluble portion of the post reaction mixture as well as additional KO^{t}Bu (10 mol% *c.f.* initial [Mn], 22.4 mg). This ethanolamine containing reaction vessel was sealed under argon and heated to 150 °C for 24 hours. After this time, the reaction is allowed to cool to room temperature and the resulting product extracted into distilled water (5 mL). Water and other volatile components are removed under reduced pressure at 110 °C to yield a polymeric product with mixed amine, imine and amine functionalities (72.5 mg).

^{1}H NMR (500 MHz, D_2O): δ_H 8.41 (CH=N), 3.65 (br s), 3.55 (br s), 3.12 (br s), 3.00 (br s), 2.79 (br s), 2.66 (br s).

^{13}C{^{1}H} NMR (126 MHz, D_2O): δ_C 179.6 (C=O), 171.0 (C=N), 81.0, 63.0, 60.1, 59.6, 58.0, 52.5, 52.0, 50.7, 49.9, 47.6, 47.4, 46.5, 42.7, 46.5, 42.7, 42.2, 39.8.

IR (ATR-FTIR, cm^{-1}): ν 3283s (O-H/N-H), 2945m (C-H), 2835m (C-H), 1655m (C=N), 1584s (N-H), 1437s (C-H), 1389s (O-H), 1055s (C-O).

GPC (H_2O, 30 °C, g mol^{-1}): M_n 43,900 g mol^{-1} (Đ 1.14).

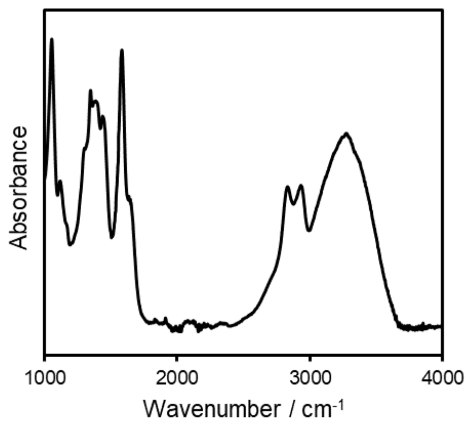

Supplementary Figure 97 IR (ATR-FTIR) spectrum for the product of the polymerisation of ethanolamine using recycled catalyst with additional base.

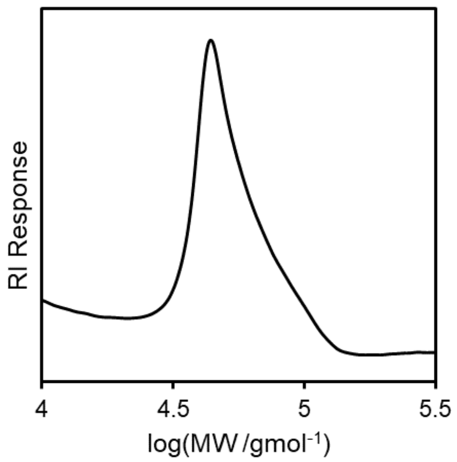

Supplementary Figure 98 Measured GPC data for the product of the polymerisation of ethanolamine using recycled catalyst with additional base.

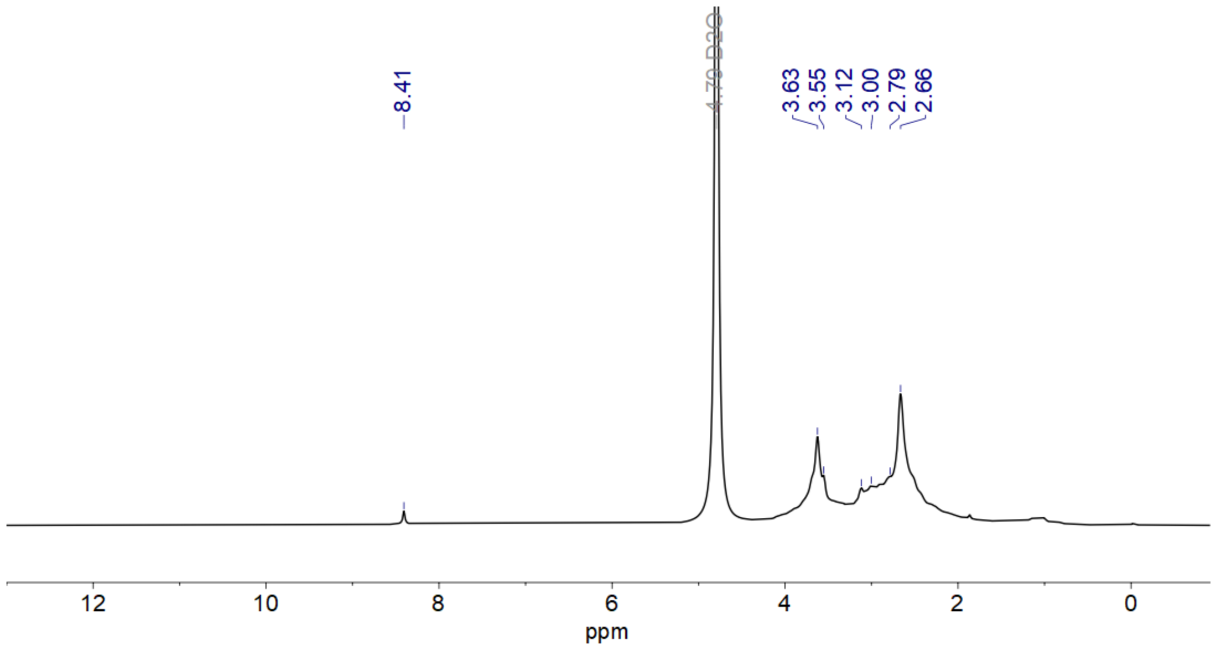

Supplementary Figure 99 ^{1}H NMR (500 MHz, D_2O) spectrum for the product of the polymerisation of ethanolamine using recycled catalyst with additional base.

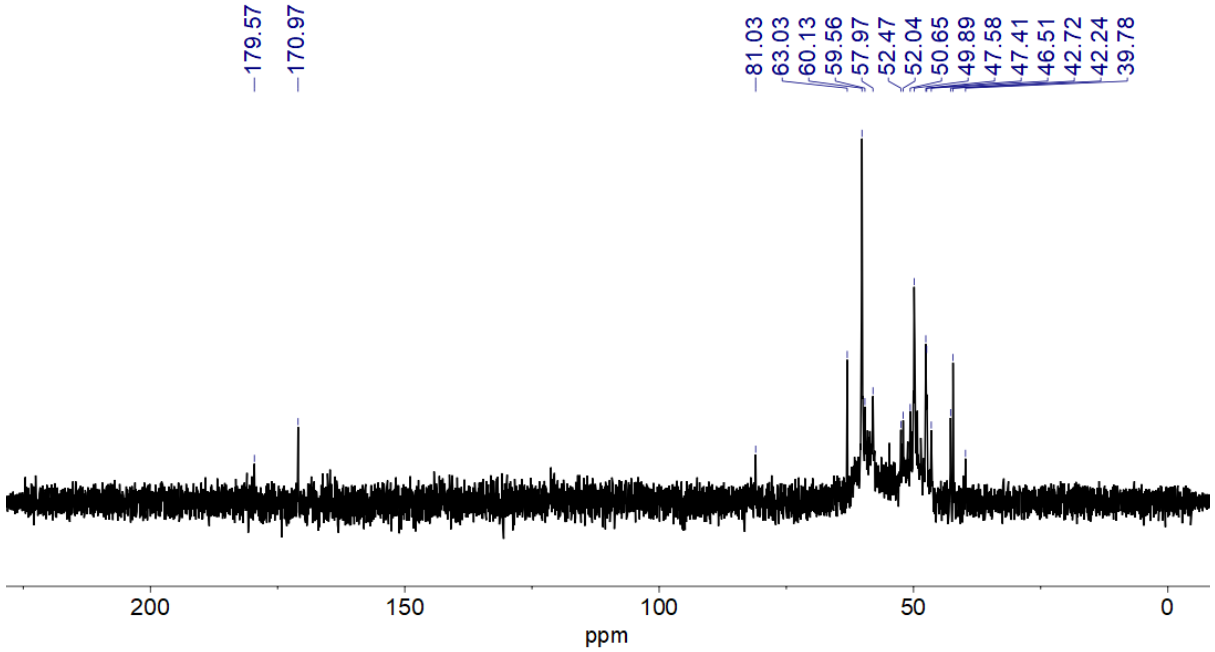

Supplementary Figure 100 $^{13}C\{^1H\}$ NMR (162 MHz, D_2O) spectrum for the product of the polymerisation of ethanolamine using recycled catalyst with additional base.

1.6 Mechanistic Studies

1.6.1 Stochiometric experiments

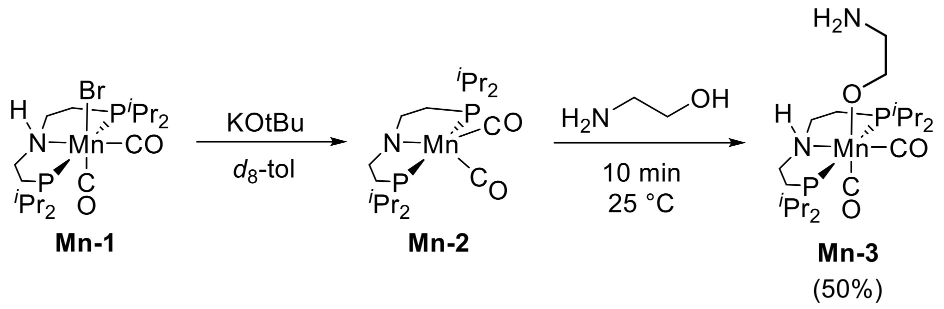

To an NMR tube equipped with a J-Young's NMR valve was added **Mn-1** (7.0 mg, 14 µmol), KO^{t}Bu (1.3 mg, 14 µmol) and toluene-d_8 (0.5 mL). The tube was heated to 110 °C for 10 minutes to generate **Mn-2** quantitatively. After cooling to room temperature, a drop of ethanolamine (*ca*. 0.05 mL) was added, and the NMR tube shaken vigorously at room temperature for 2 minutes. The reaction was probed *in situ* by NMR spectroscopy (^{1}H, ^{31}P{^{1}H}, ^{13}C{^{1}H}).

^{31}P{^{1}H} NMR (203 MHz, toluene-d_8): δ_P **Mn-3** = 81.3

1.6.1.1 NMR Spectra

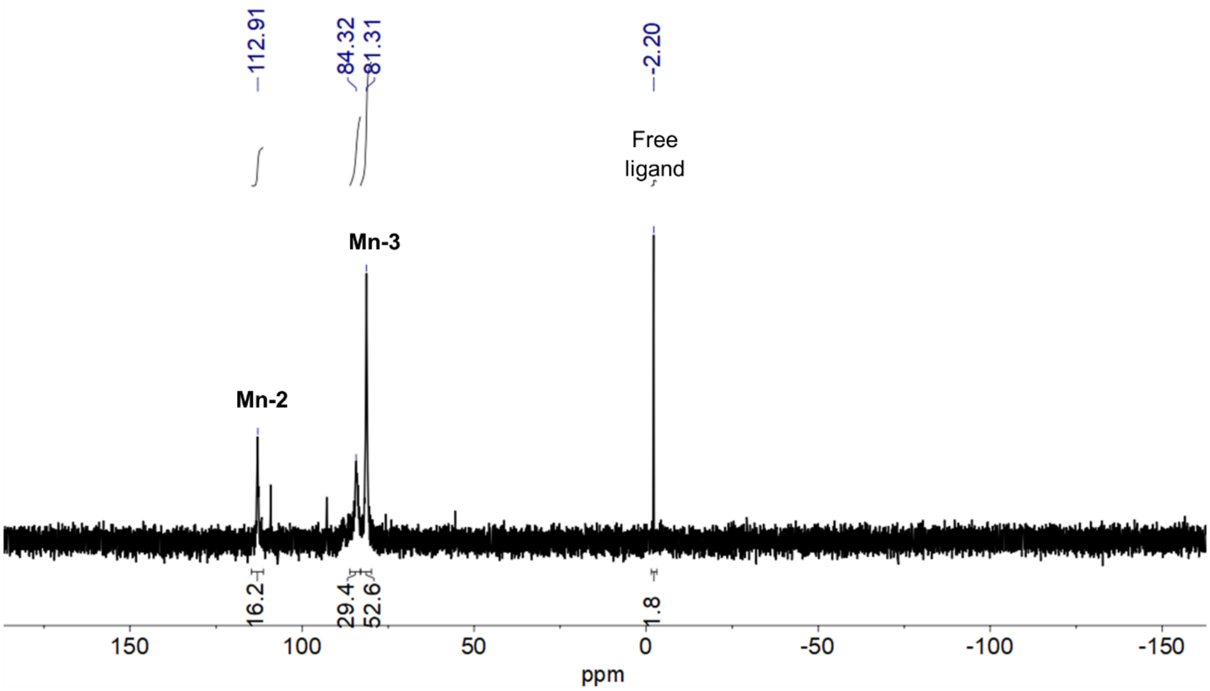

Supplementary Figure 101 ^{31}P{^{1}H} NMR (203 MHz, toluene-d$_8$) spectrum obtained for the reaction of **Mn-2** with ethanolamine.

1.6.2 In situ NMR spectra

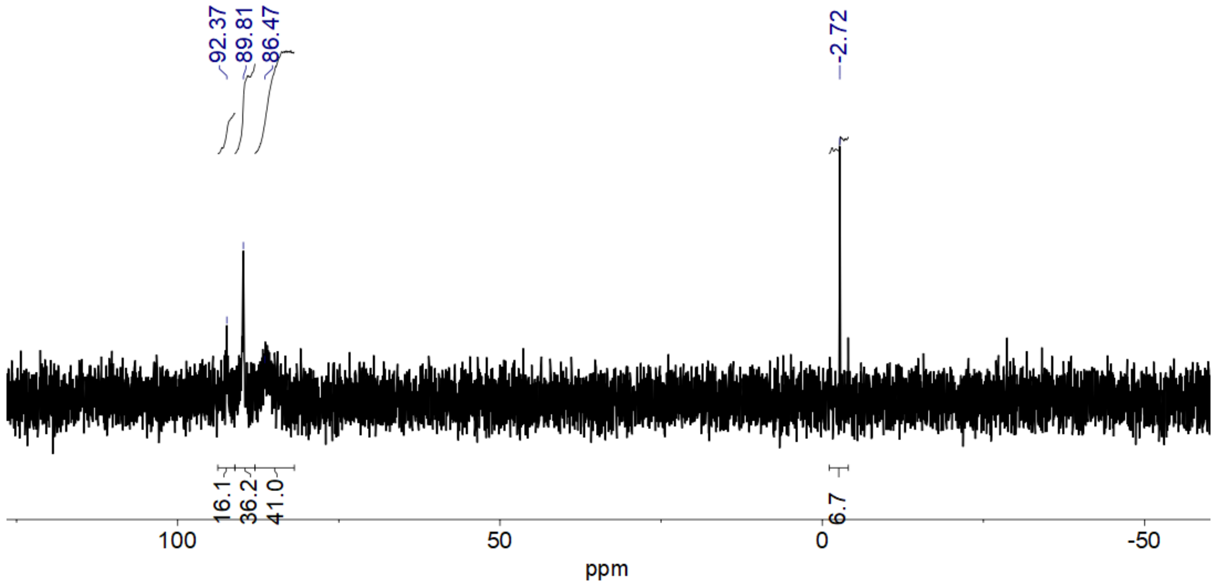

Supplementary Figure 102 *In situ* $^{31}P\{^{1}H\}$ NMR (202 MHz, H_8-toluene) spectrum after catalysis allowed to proceed for 1 hour at 150 °C.

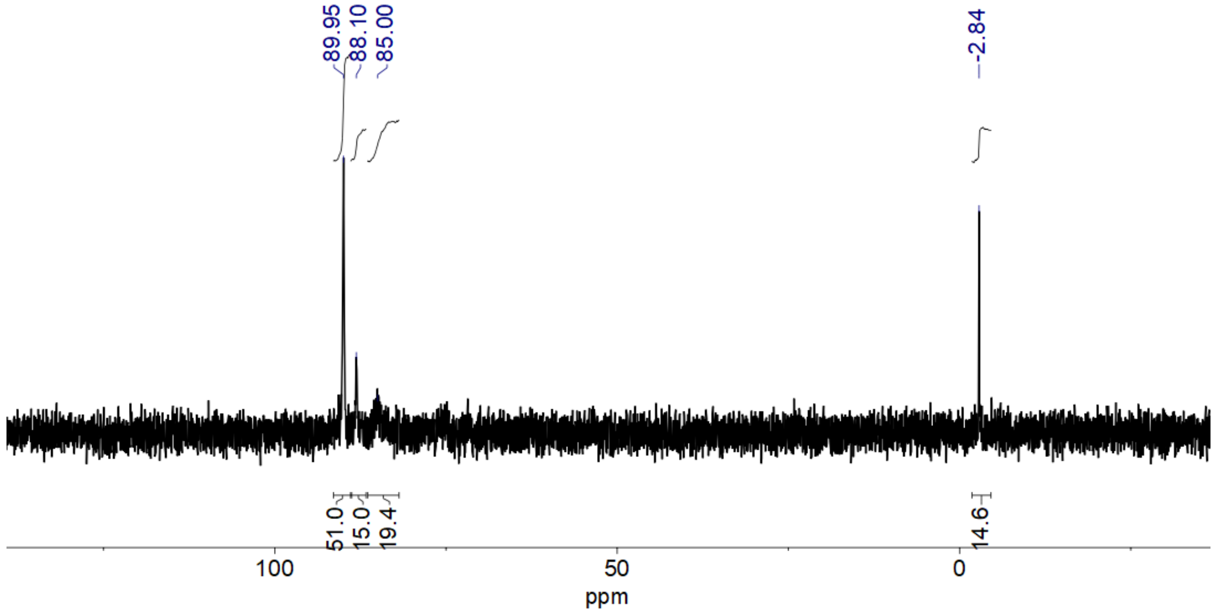

Supplementary Figure 103 *In situ* $^{31}P\{^{1}H\}$ NMR (202 MHz, H_8-toluene) spectrum after catalysis allowed to proceed for 24 hours at 150 °C.

1.6.3 Polymerisation of N-(2-Hydroxyethyl)ethylenediamine

To probe the role of *N*-(2-hydroxyethyl)ethylenediamine as a potential intermediate within the reaction, this compound was applied as a substrate for the production of **PEI-1**.

Procedure: A 100 mL ampoule equipped with a J-Young's valve was charged with pre-catalyst (**Mn-1**; 10 mg, 0.02 mmol, 1 mol%) and KO^{t}Bu (22.4 mg, 0.20 mmol, 10 mol%). Toluene (4 mL) and *N*-(2-hydroxyethyl)ethylenediamine (0.20 mL, 2.0 mmol) were added, and the flask was sealed under an argon atmosphere before heating to 150 °C for 24 hours with stirring (400 rpm). After this period, the reaction vessel was allowed to cool to room temperature. The product was extracted into distilled water (5 mL) before volatile components are removed under reduced pressure at 110 °C to yield **PEI-1** (96.6 mg, 56%).

^{1}H NMR (500 MHz, D_2O): δ_H 3.89 (br s), 3.73 (br s), 3.29 (br s), 2.98 (br s), 2.81 (br s), 2.68 (br s), 2.42 (br s).

^{13}C{^{1}H} NMR (126 MHz, D_2O): δ_C 53.1, 49.3, 47.2, 46.5, 44.5, 44.0, 43.6, 40.3, 39.4, 38.3.

IR (ATR-FTIR, cm^{-1}): ν 3272m (O-H/N-H), 2936w (C-H), 2849w (C-H), 1562s (N-H), 1458s (C-H), 1332s (C-N), 1121m (C-N).

GPC (H_2O, 30 °C, g mol^{-1}): M_n 46,100 g mol^{-1} (Đ 1.22).

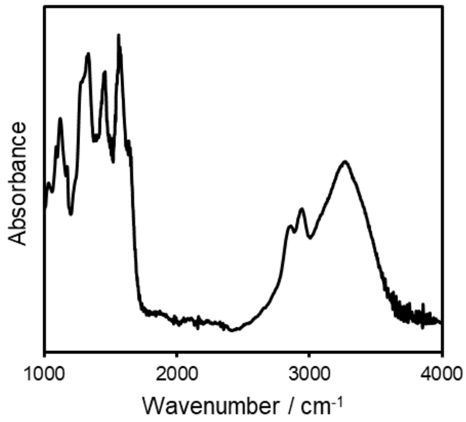

Supplementary Figure 104 IR (ATR-FTIR) spectrum corresponding to product of *N*-(2-hydroxyethyl)ethylenediamine polymerisation.

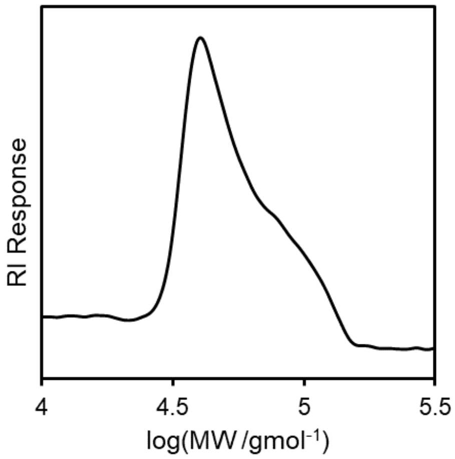

Supplementary Figure 105 GPC chromatograph corresponding to product of *N*-(2-hydroxyethyl)ethylenediamine polymerisation.

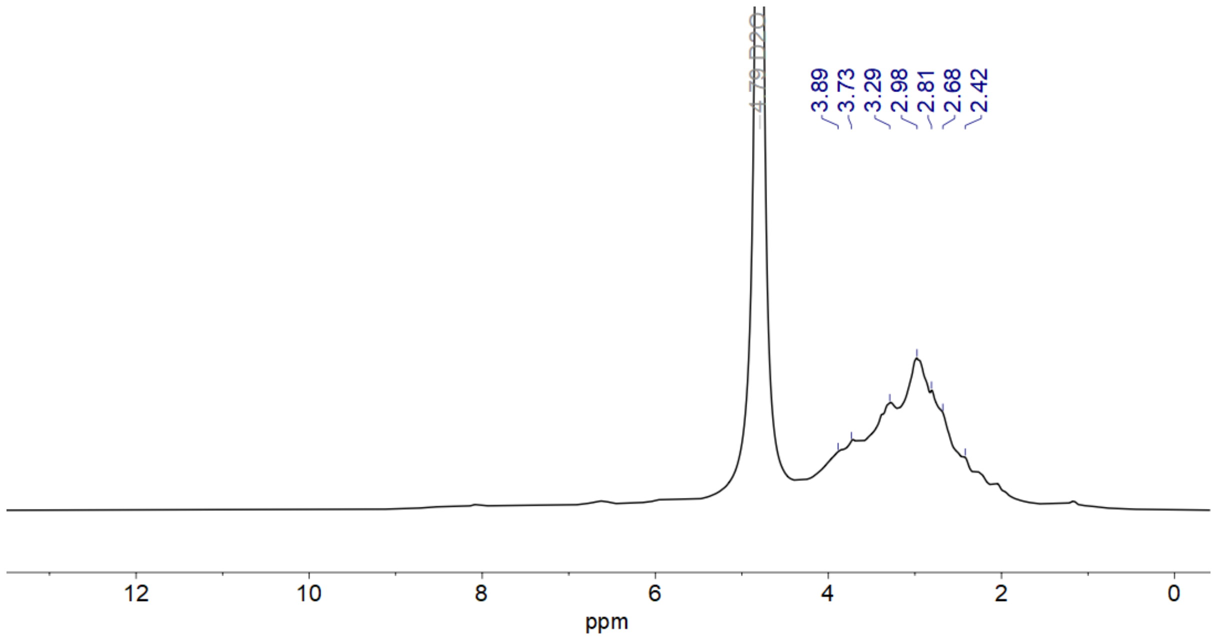

Supplementary Figure 106 ^{1}H NMR (500 MHz, D_2O) spectrum corresponding to product of *N*-(2-hydroxyethyl)ethylenediamine polymerisation.

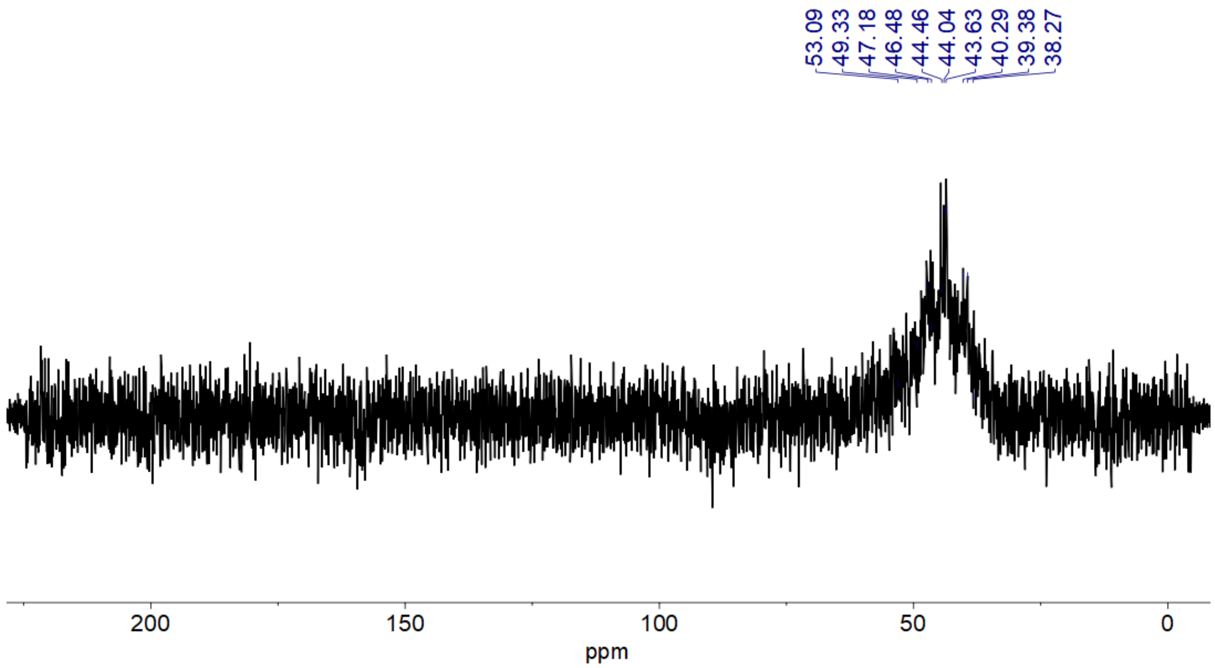

Supplementary Figure 107 $^{13}C\{^{1}H\}$ NMR (126 MHz, D_2O, l.b. 5 Hz) spectrum corresponding to product of *N*-(2-hydroxyethyl)ethylenediamine polymerisation.

1.6.4 Hydrogenation of N-benzyl-1-phenylmethanimine

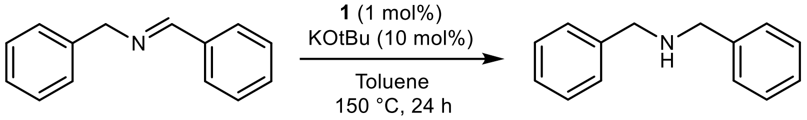

A microwave vial is charged with pre-catalyst (**Mn-1**; 5 mg, 0.01 mmol, 1 mol%) and base (KO^{t}Bu, 11.2 mg, 0.10 mmol, 10 mol%). Toluene (2 mL) and *N*-benzyl-1-phenylmethanimine (0.20 mL, 1 mmol) are added and the vial is pierced with a needle and placed in a stainless-steel autoclave containing aluminium beads for effective heat transfer. The argon atmosphere is replaced with H_2 (1 bar) and the vessel sealed before heating to 150 °C for 24 hours with stirring (400 rpm). After this period, the reaction vessel is allowed to cool to room temperature and the H_2 atmosphere is vented. Volatile components are removed *in vacuo*. The product is analysed by GC-MS as a solution in DCM and revealed partial imine hydrogenation to *N*,*N*'-dibenzylamine (15 %).

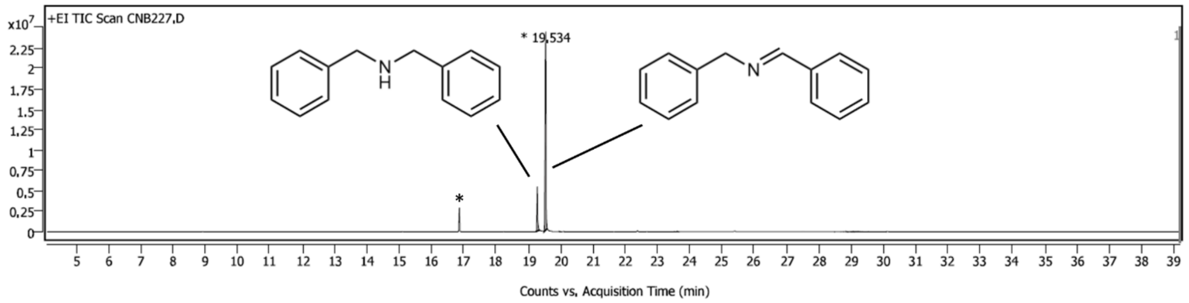

Chromatogram Peaks

Peak	Start	RT	End	Height	Area	Area %	SNR
1	19.234	19.279	19.342	5446908	9159550	17.08	
2	19.485	19.534	19.588	22681168	53627906	100.00	

Supplementary Figure 108 GC chromatograph of the products of hydrogenation of *N*-benzyl-1-phenylmethanimine. *indicates internal standard, 1,1-diphenyletheylene.

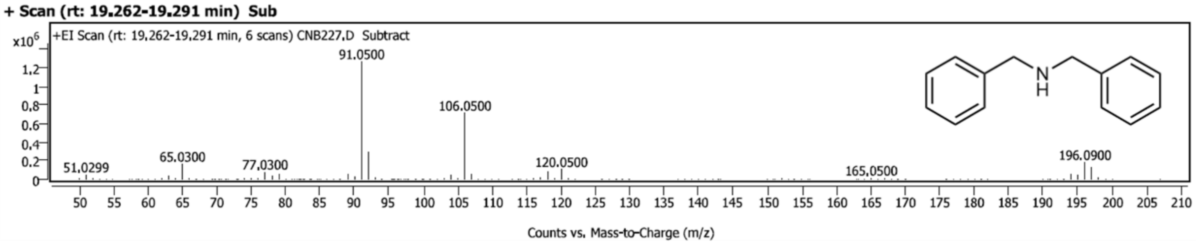

Supplementary Figure 109 Mass spectrum (EI) of *N*,*N*'-dibenzylamine obtained from the GC-MS analysis of the products produced from hydrogenation of *N*-benzyl-1-phenylmethanimine.

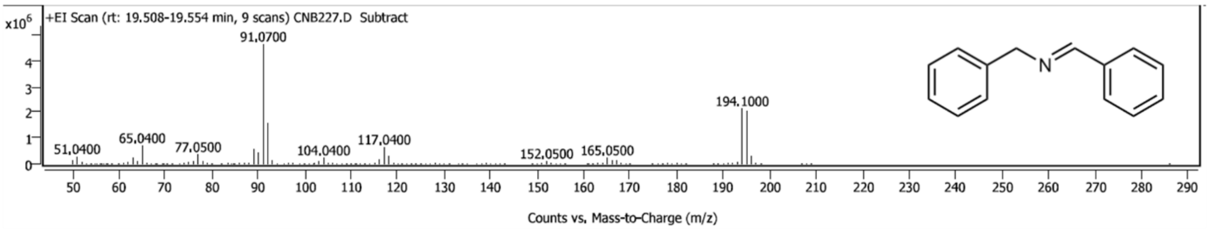

Supplementary Figure 110 Mass spectrum (EI) of *N*-benzyl-1-phenylmethanimine obtained from the GC-MS analysis of the products produced from hydrogenation of *N*-benzyl-1-phenylmethanimine.

1.6.5 Hydrogenation of 1,5-cyclooctadiene

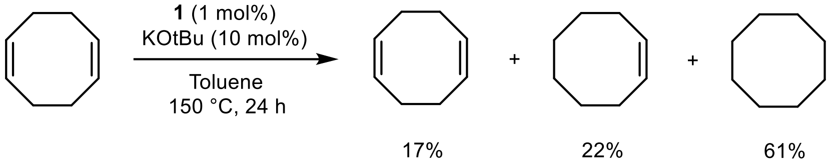

A microwave vial is charged with pre-catalyst (**Mn-1**; 5 mg, 0.01 mmol, 1 mol%) and base (KO^{t}Bu, 11.2 mg, 0.10 mmol, 10 mol%). Toluene (2 mL) and 1,5-cyclococtadiene (0.12 mL, 1 mmol) are added and the vial is pierced with a needle and placed in a stainless-steel autoclave containing aluminium beads for effective heat transfer. The argon atmosphere is replaced with H_2 (1.2 bar) and the vessel sealed before heating to 150 °C for 24 hours with stirring (400 rpm). After this period, the reaction vessel is allowed to cool to room temperature and the H_2 atmosphere is vented. Volatile components are removed *in vacuo*. The product is analysed by GC-MS as a solution in DCM and revealed 83% conversion, with 75% selectivity to cyclooctane and 25% selectivity to cyclooctene.

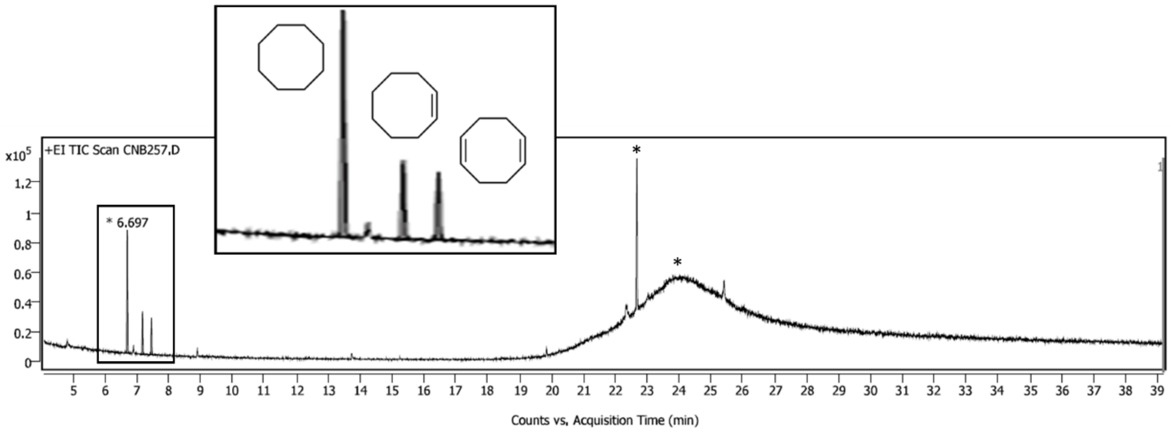

Chromatogram Peaks

Peak	Start	RT	End	Height	Area	Area %	SNR
1	6.656	6.697	6.748	82664	147221	100.00	
2	7.126	7.177	7.229	28997	51276	34.83	
3	7.423	7.458	7.503	24749	42089	28.59	

Supplementary Figure 111 GC chromatograph of the products of hydrogenation of 1,5-cyclooctadiene. *indicates column bleed (silicas).

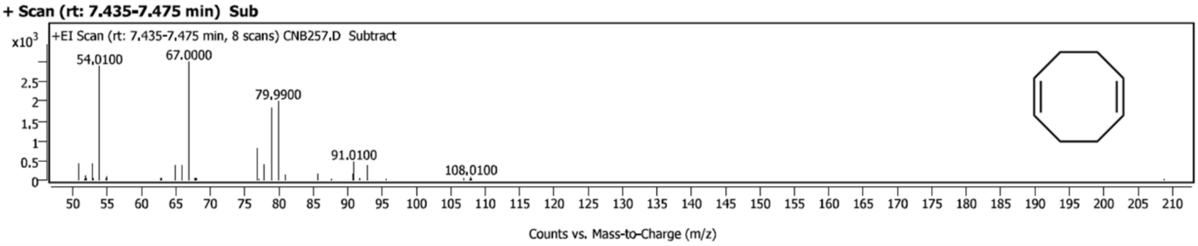

Supplementary Figure 112 Mass spectrum (EI) of 1,5-cyclooctadiene obtained from the GC-MS analysis of the products produced from hydrogenation of 1,5-cyclooctadiene.

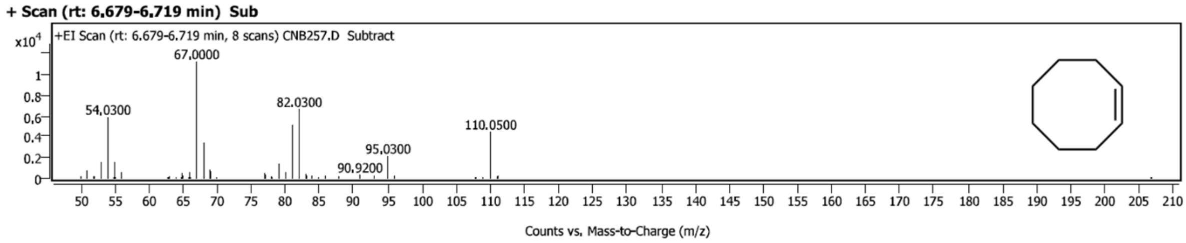

Supplementary Figure 113 Mass spectrum (EI) of cyclooctene obtained from the GC-MS analysis of the products produced from hydrogenation of 1,5-cyclooctadiene.

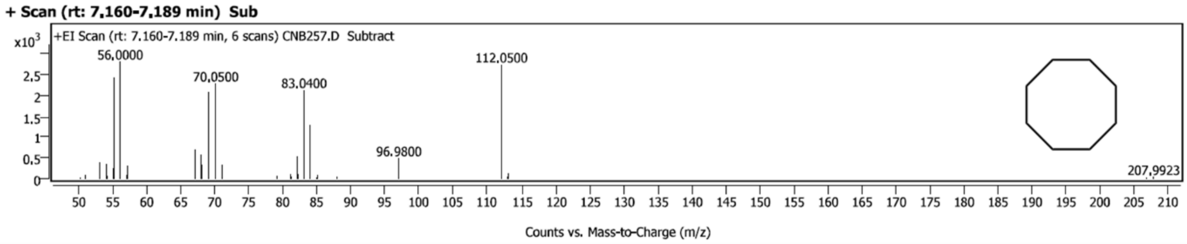

Supplementary Figure 114 Mass spectrum (EI) of cyclooctane obtained from the GC-MS analysis of the products produced from hydrogenation of 1,5-cyclooctadiene.

1.6.6 Hydrogenation of 1-(1-butenyl)pyrrolidine

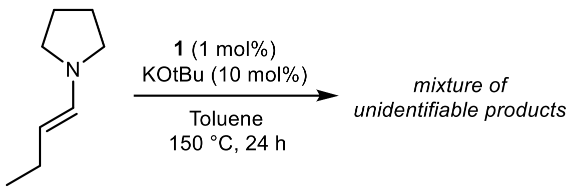

A microwave vial is charged with pre-catalyst (**Mn-1**; 5 mg, 0.01 mmol, 1 mol%), base (KO^{t}Bu, 11.2 mg, 0.10 mmol, 10 mol%) and 1-(1-butenyl)pyrrolidine (125 mg, 1.00 mmol). Toluene (2 mL) was added and the vial is pierced with a needle and placed in a stainless-steel autoclave containing aluminium beads for effective heat transfer. The argon atmosphere is replaced with H_2 (1.2 bar) and the vessel sealed before heating to 150 °C for 24 hours with stirring (400 rpm). After this period, the reaction vessel is allowed to cool to room temperature and the H_2 atmosphere is vented. Volatile components are removed *in vacuo*. The product is analysed by GC-MS as a solution in DCM, which revealed a complex mixture of unidentifiable products, and by ^{1}H NMR to monitor the enamine region.

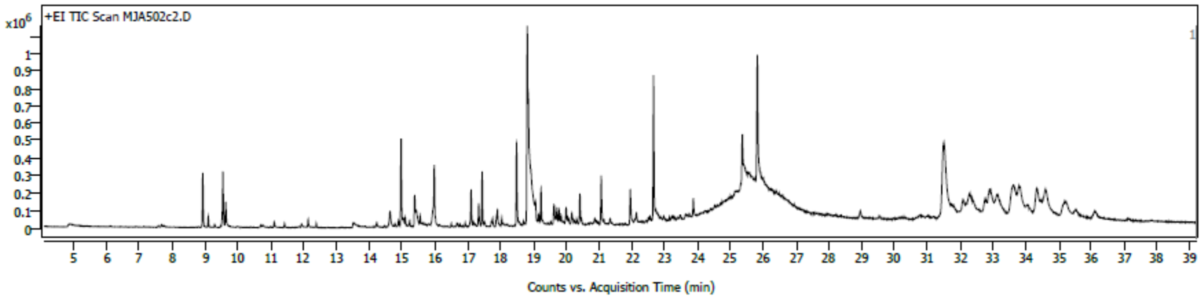

Supplementary Figure 115 GC chromatograph of the products of hydrogenation of *N*-benzyl-1-phenylmethanimine.

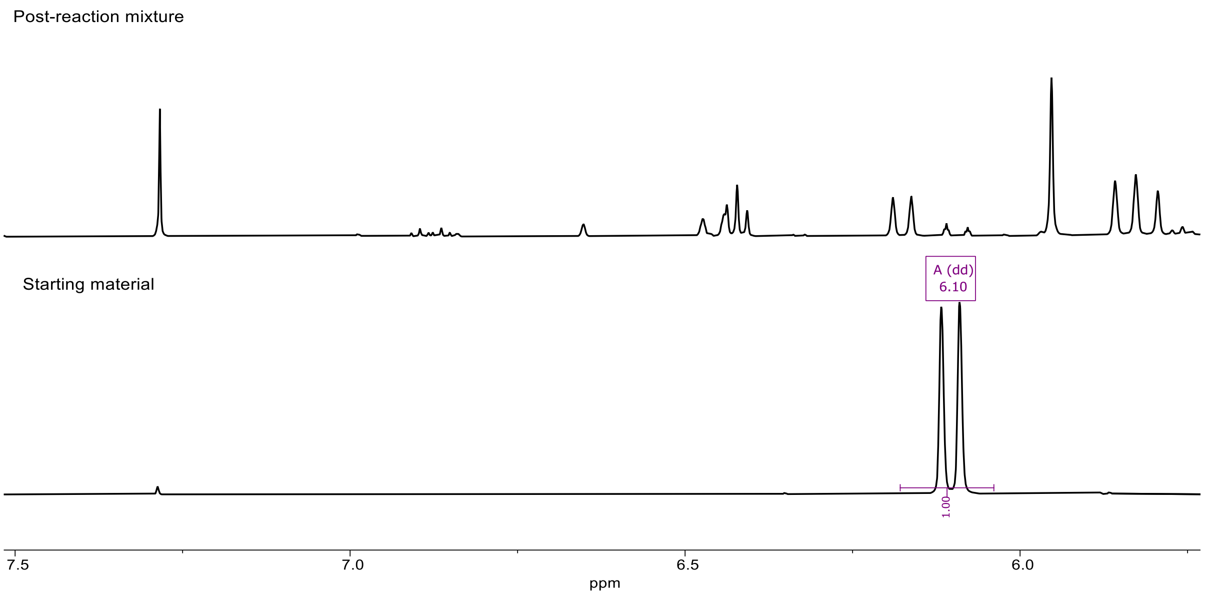

Supplementary Figure 116 Stacked ^{1}H NMR spectra between 5.5 and 7.5 ppm (enamine region) of 1-(1-butenyl)pyrrolidine with the reaction mixture obtained from the hydrogenation of 1-(1-butenyl)pyrrolidine, showing the initial peak corresponding to the enamine proton.

1.6.7 Headspace gas analysis of the dehydrogenative coupling of ethanolamine

A 100 mL ampoule equipped with a J-Young's valve was charged with pre-catalyst (**Mn-1**; 10 mg, 0.02 mmol, 1 mol%) and base (*e.g.* KO^{t}Bu, 22.4 mg, 0.20 mmol, 10 mol%). Toluene (4 mL) and ethanolamine (0.12 mL, 2.0 mmol) were added and the flask was sealed under an argon atmosphere before heating to 150 °C for 5 hours with stirring (400 rpm). After this period, the reaction vessel was allowed to cool to room temperature. A sample of the headspace was extracted using a gas tight syringe and analysed using GC-TCD. Comparison with a standard sample of H_2 confirms the release of H_2 gas during the reaction progression.

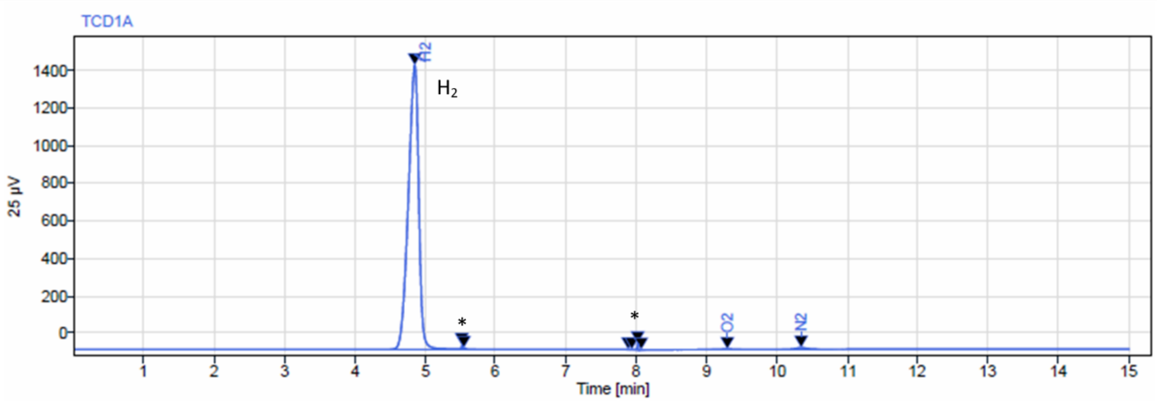

Supplementary Figure 117 GC-TCD chromatograph of the reaction headspace from the dehydrogenative coupling of ethanoamine mediated by Mn-1, showing H_2 release. *indicates positions of valve change events.

1.6.8 Polymerisation under D_2 gas (1.2 bar)

A 100 mL ampoule equipped with a J-Young's valve was charged with pre-catalyst (**Mn-1**; 10 mg, 0.02 mmol, 1 mol%) and base (*e.g.* KO^{t}Bu, 22.4 mg, 0.20 mmol, 10 mol%). Toluene (4 mL) and

ethanolamine (0.12 mL, 2.0 mmol) were added and the flask was sealed under an argon atmosphere, before degassing and replaced with D_2 (1.2 bar). The ampoule was then heated to 150 °C for 24 hours with stirring (400 rpm). After this period, the reaction vessel was allowed to cool to room temperature. The product was then extracted into distilled water (5 mL) and any volatile components were removed under reduced pressure at 110 °C, to yield the polymer.

^{1}H NMR (500 MHz, D_2O): δ_H 8.36 (s, CH=N), 3.50 (br s), 3.07 (br s), 2.86 (br s), 2.63 (br s).

^{2}H NMR (500 MHz, H_2O): δ_D 4.95 (br s), 3.71 (br s), 2.84 (br s)

^{13}C{^{1}H} NMR (126 MHz, D_2O): δ_C 171.0, 164.8, 155.6, 144.7, 143.9, 142.1, 63.1, 61.4, 50.1, 50.0, 43.2, 42.3, 42.2.

1.6.8.1 NMR Spectra

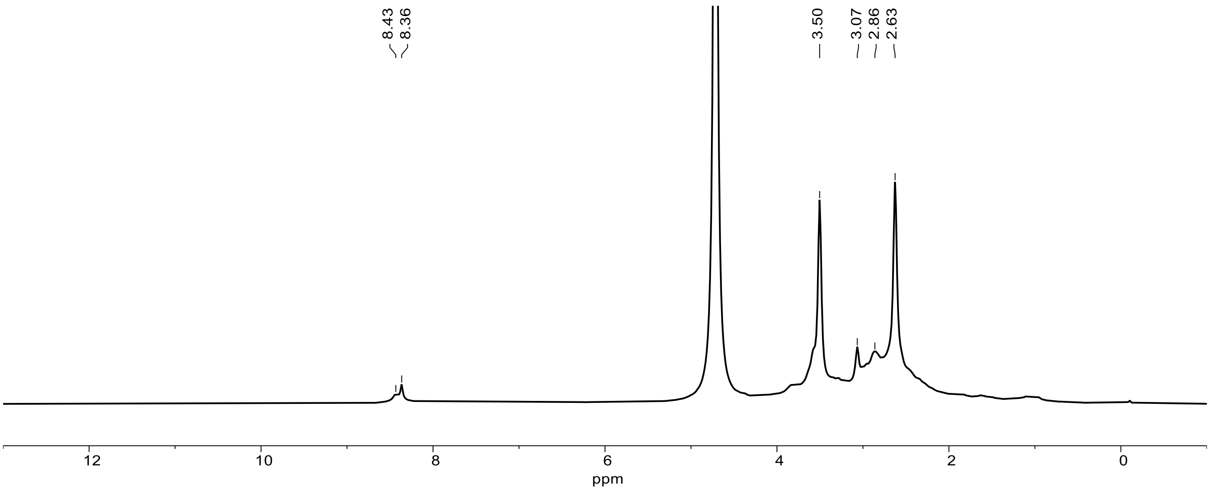

Supplementary Figure 118 ^{1}H NMR (500 MHz, D_2O) spectrum obtained for **PEI-1** formed under D_2.

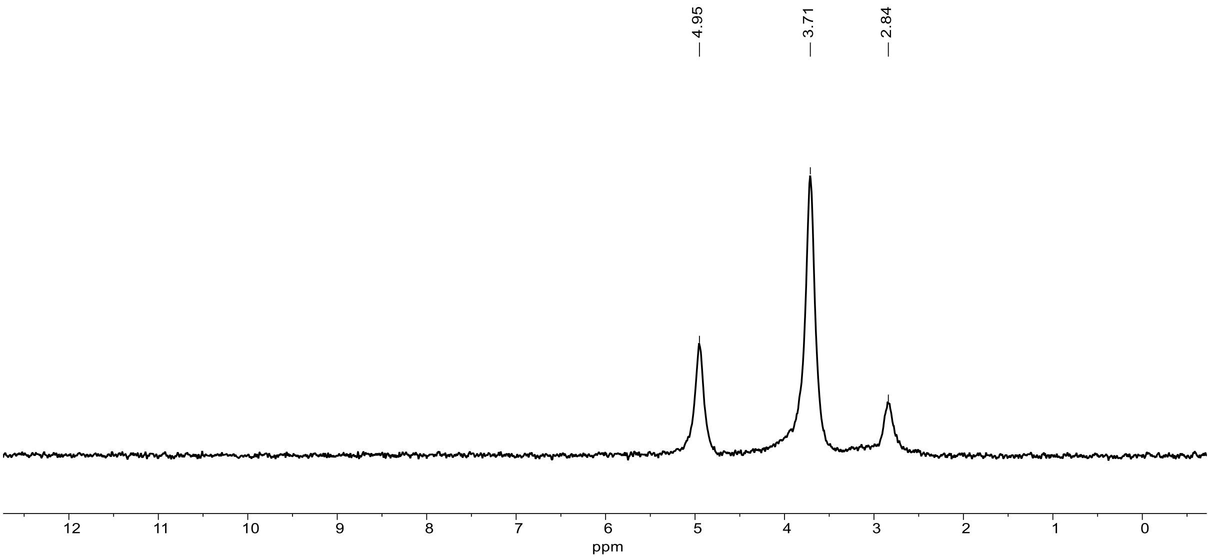

Supplementary Figure 119 ^{2}H NMR (77 MHz, H_2O, d_1 = 1, ns = 128) spectrum obtained for **PEI-1** formed under D_2.

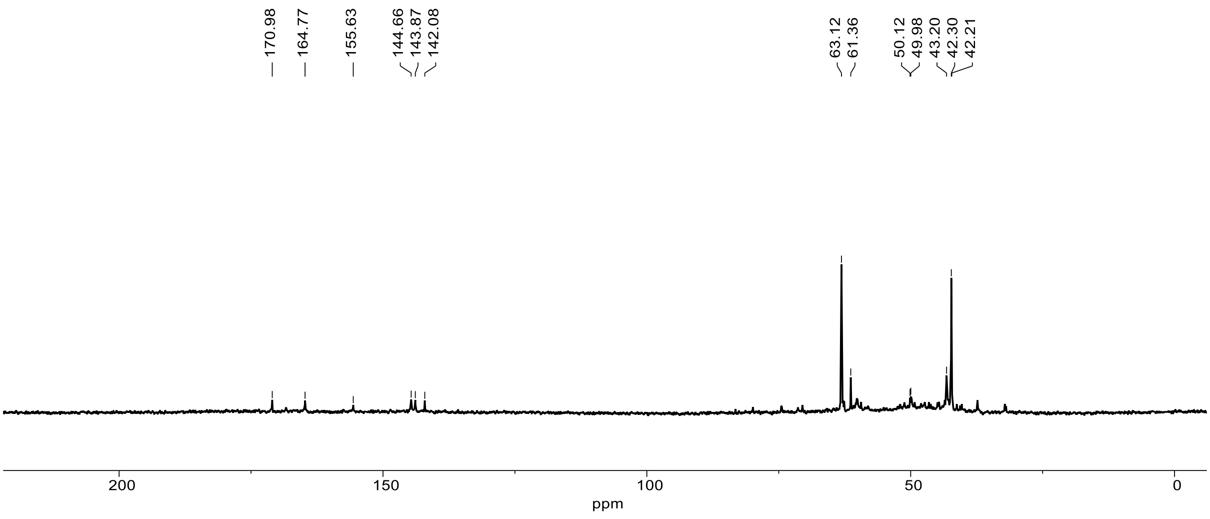

Supplementary Figure 120 $^{13}C\{^{1}H\}$NMR (126 MHz, D_2O) spectrum obtained for **PEI-1** formed under D_2.

1.6.9 Polymerisation using pre-formed catalyst

Preparation of active complex **Mn-2**: A 100 mL ampoule equipped with a J-Young's valve was charged with **Mn-1** (30 mg, 0.06 mmol) and KO^{t}Bu (7.5 mg, 0.07 mmol, 1.1 equiv.). Toluene (4 mL) was added and the flask sealed under an argon atmosphere before stirring (400 rpm) at room temperature for 18 hours. Solvent was then removed under reduced pressure before hexane (5 mL) was added. The flask was then chilled to -30 °C and the solution was filtered and kept at −30 °C over 3 days, resulting in the formation of red crystals (**Mn-2**; 12.2 mg, 49%) Data is in agreement with literature.[S2]

^{31}P{^{1}H} NMR (202 MHz, toluene-d8): 113.0 (s)

Polymerisation was then carried out as follows.

A 100 mL ampoule equipped with a J-Young's valve was charged with **Mn-2** (8.3 mg, 0.02 mmol, 1 mol%), toluene (4 mL) and ethanolamine (0.12 mL, 2.0 mmol) and the flask was sealed under an argon atmosphere before heating to 150 °C for 24 hours with stirring (400 rpm). After this period, the reaction vessel was allowed to cool to room temperature. The product was extracted into distilled water (5 mL) and any volatile components were removed under reduced pressure at 110 °C, to yield the polymer (66.1 mg, 77%).

^{1}H NMR (500 MHz, D_2O): δ_H 3.67 (br s), 3.60 (br s), 3.35 (br s), 2.97 (br s), 2.75 (br s).

^{13}C{^{1}H} NMR (126 MHz, D_2O): δ_C 62.6, 59.9, 56.0, 49.8, 44.2.

IR (ATR-FTIR, cm^{-1}): ν 3246m (O-H/N-H), 2924m (C-H), 2859m (C-H), 1630m (C=N/C=O), 1570m (N-H), 1449m (C-H), 1340m (O-H), 1053s (C-O).

GPC (H_2O, 30 °C, g mol^{-1}): M_n 33,200 g mol^{-1} (Đ 1.29).

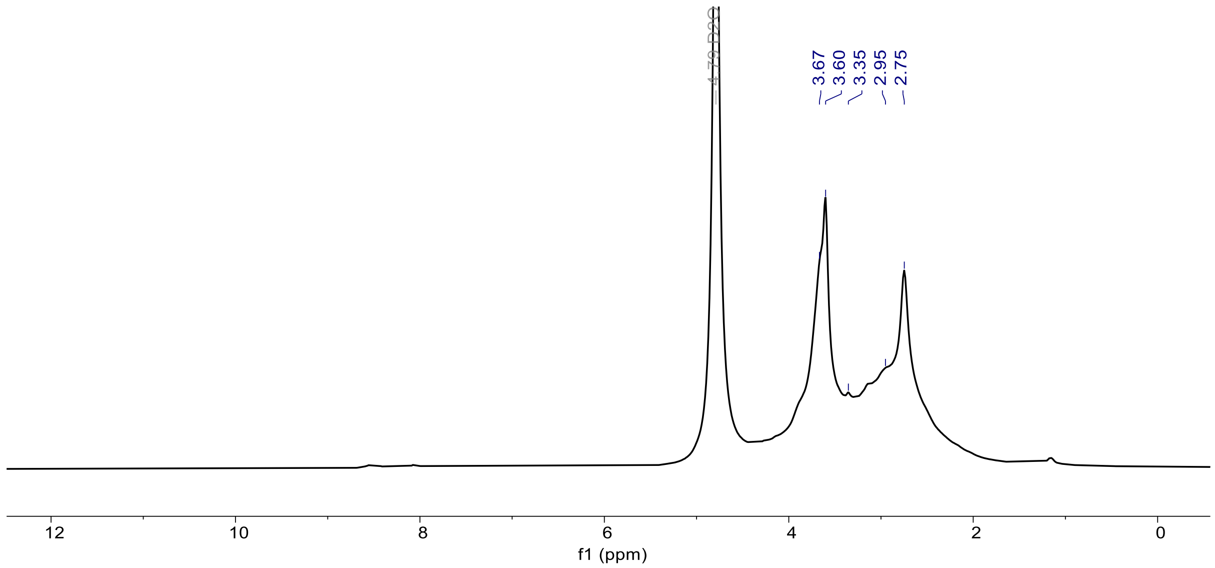

Supplementary Figure 121 ^{1}H NMR (500 MHz, D_2O) spectrum corresponding to polymer produced using **Mn-2** catalyst.

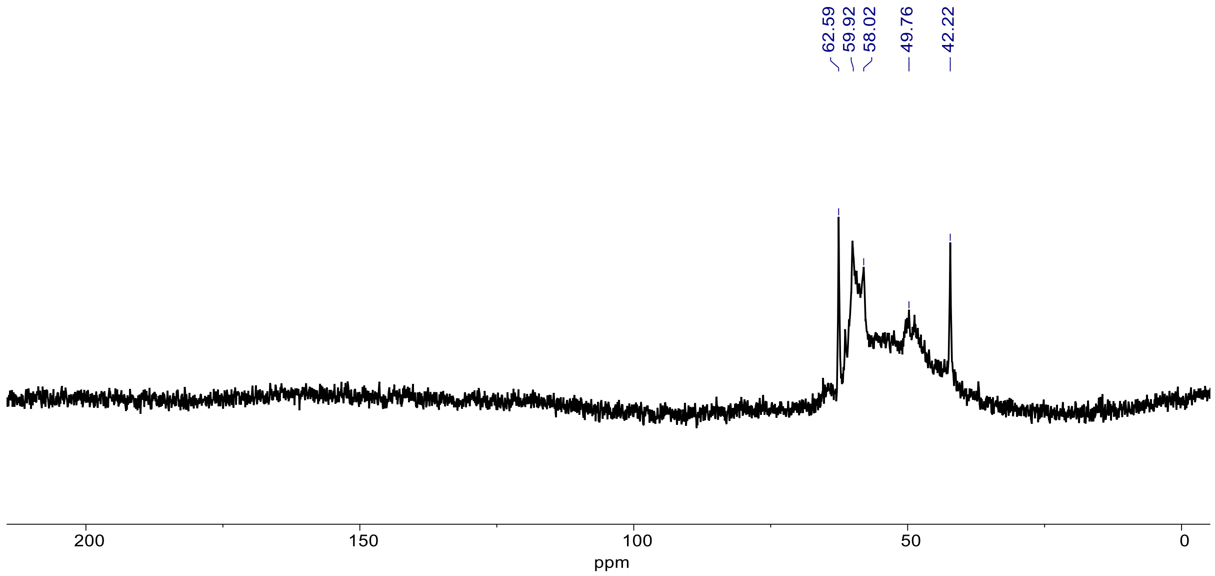

Supplementary Figure 122 $^{13}C\{^{1}H\}$ NMR (126 MHz, D_2O, l.b. 5 Hz) spectrum corresponding to polymer produced using **Mn-2** catalyst.

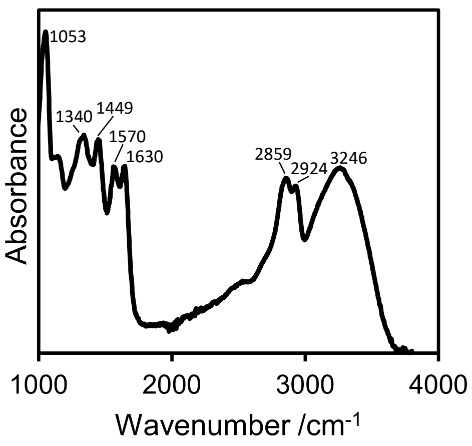

Supplementary Figure 123 IR (ATR-FTIR) spectrum corresponding to polymer produced using **Mn-2** catalyst.

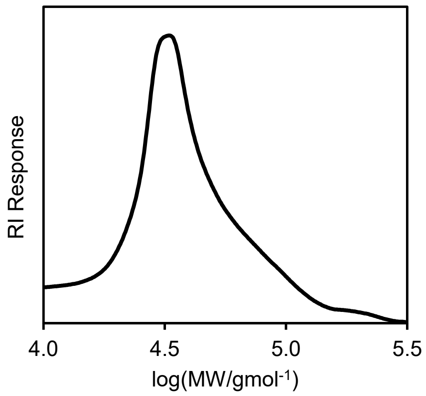

Supplementary Figure 124 GPC chromatograph corresponding to polymer produced using **Mn-2** catalyst.

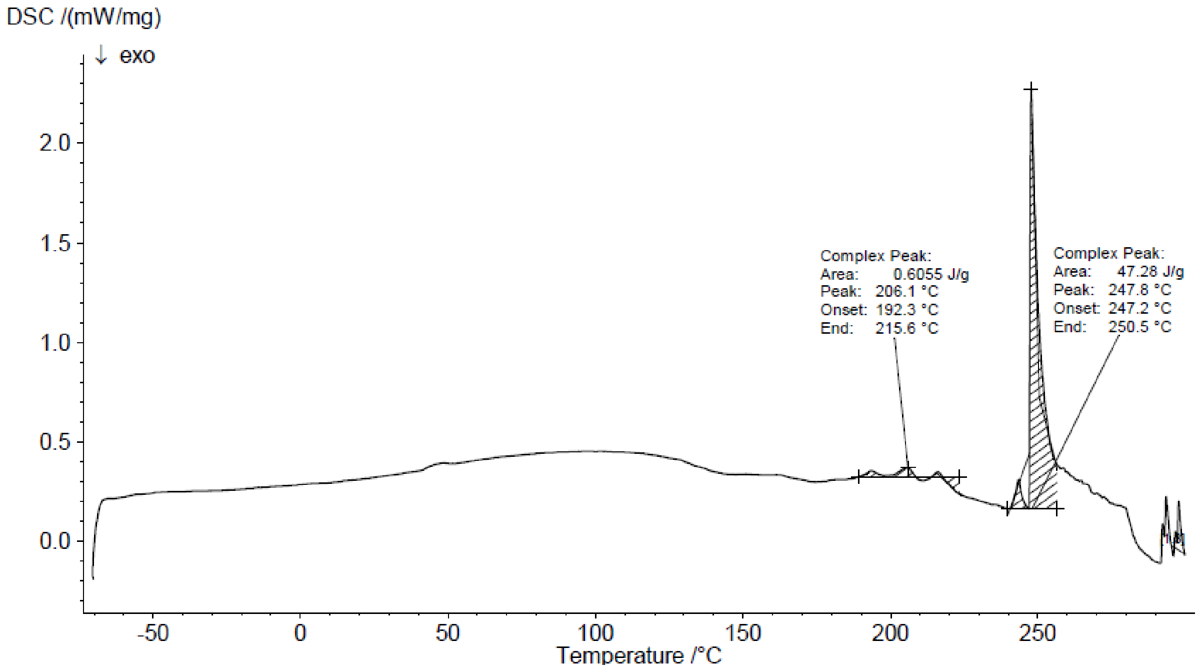

Supplementary Figure 125 DSC heatflow *vs* temperature corresponding to polymer produced by **Mn-2**. Peak at 192.3 °C is likely caused by residual ethanolamine.

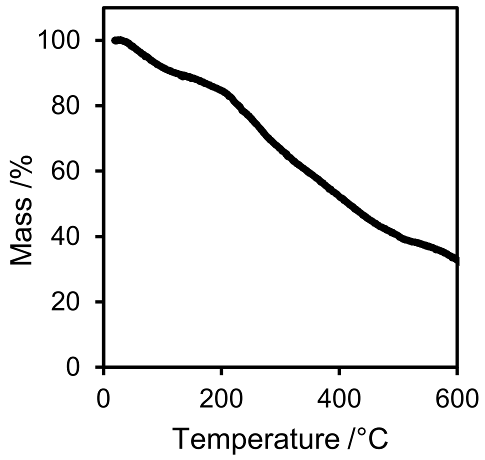

Supplementary Figure 126 TGA curve obtained for product corresponding to Supplementary Table 1; Entry 1.

1.7 Polymer Degradation

To explore the solubility of polymer in different solvents such as trifluoroacetic acid, polymer produced by the scale-up procedure (see SI Section 1.9) was dissolved in trifluoroacetic acid. The samples were then kept for a set period of time, before the trifluoroacetic acid was removed under reduced pressure. The resultant polymer was then dissolved in H_2O and analysed through GPC analysis. This showed a dramatic loss in M_n, and broadening of PDI over a very short timeframe suggestive of polymer degradation.

Time	M_n	PDI
0 minutes	43,371	1.28
2 minutes	579	3.63
120 minutes	339	3.71

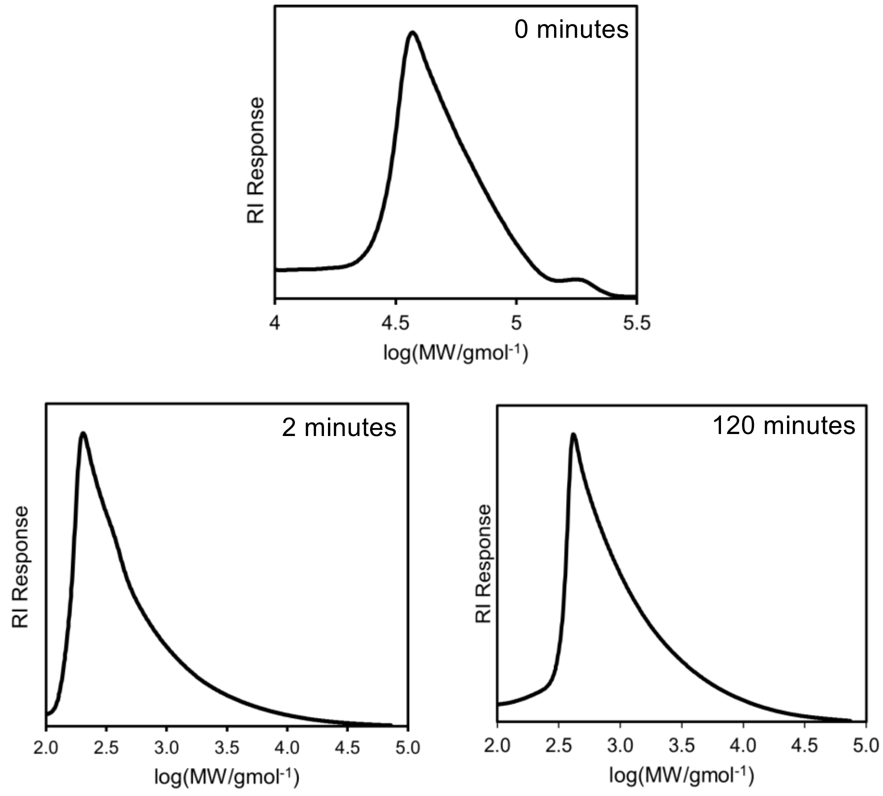

Supplementary Figure 127 GPC traces of PEI-1 showing a reducing molecular weight with time exposed to trifluoroacetic acid.

1.8 CHN/O Analyses

Supplementary Table 3 CHN elemental analysis results for selected **PEI-1** samples.

**Substrate**	**C %**	**H %**	**N %**	**Residual %**
C_2H_7NO	52.36	7.55	18.41	21.69
C_2H_7NO + 0.25 equiv. $C_2H_8N_2$	55.52	7.25	20.66	16.57
C_2H_7NO + 0.50 equiv. $C_2H_8N_2$	55.10	7.05	22.05	15.81
		Theoretical		
PEI $(C_2H_5N)_n$	55.78	11.70	32.52	-
PEIE $(C_4H_9NO)_n$	55.15	10.41	16.08	18.36

C_2H_7NO denotes ethanolamine and $C_2H_8N_2$ denotes ethylene dimaine.

1.9 Scale Up Procedure

Safety note: The reaction is carried out in a 150 mL stainless steel autoclave. This is due to the quantity of solvent (20 mL toluene) being used above its boiling point and the possibility of dihydrogen build up. At this 20 mmol scale the maximum theoretical pressure possible is 49 bar in the event of volatilisation of all solvent present and dehydrogenation with no hydrogenation. The reaction is carried out under inert conditions using standard Schlenk techniques.

Scale up procedure: To a 150 mL stainless steel autoclave is added precatalyst **Mn-1** (49.5 mg, 0.1 mmol, 0.5 mol%), KO^{t}Bu (224 mg, 2 mmol, 10 mol%) and toluene (20 mL). Ethanolamine (1.20 mL, 20 mmol) is added, and the reaction vessel sealed under argon before heating at 150 °C with magnetic stirring (400 rpm) for 24 hours. After this time, the vessel is allowed to cool to room temperature and opened carefully to release any residual pressure. The product is extracted into distilled water (20 mL) and any volatile components are removed under reduced pressure at 110 °C to yield **PEI-1** (725 mg, 84%).

^{1}H NMR (500 MHz, D_2O): δ_H 8.39 (CH=N), 8.04 (CH=N), 3.67 (br s), 3.34 (br s), 3.11 (br s), 2.91 (br s), 2.55 (br s).

^{13}C{^{1}H} NMR (126 MHz, D_2O): δ_C 164.7 (C=N), 61.4, 60.1, 59.6, 58.6, 58.1, 43.3, 43.2, 41.7.

IR (ATR-FTIR, cm^{-1}): ν 3250m (O-H/N-H), 2920w (C-H), 2849w (C-H), 1578s (N-H), 1389s (O-H), 1057s (C-O).

DSC (N_2, °C): No T_m or T_g observed between 30 °C and 600 °C.

GPC (H_2O, 30 °C, g mol^{-1}): M_n 40,100 g mol^{-1} (Đ 1.23).

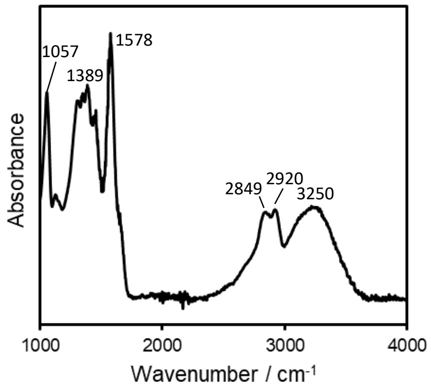

Supplementary Figure 128 IR (ATR-FTIR) spectrum of product obtained from scaled-up reaction.

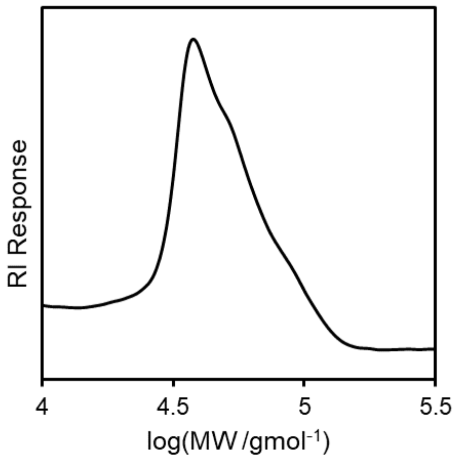

Supplementary Figure 129 GPC chromatograph corresponding to product of polymerisation in scaled-up reaction.

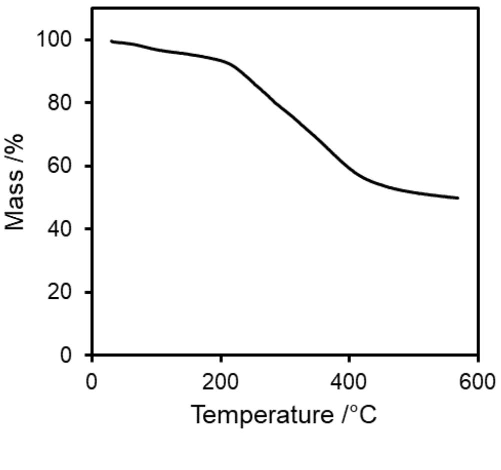

Supplementary Figure 130 TGA curve corresponding to product of polymerisation of scaled-up reaction.

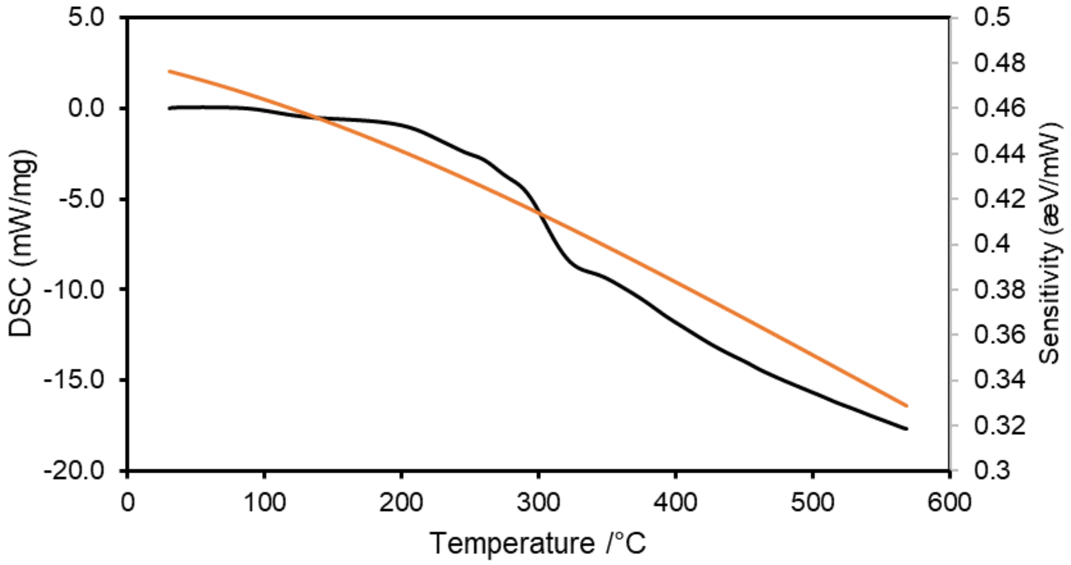

Supplementary Figure 131 DSC trace corresponding to product of polymerisation of scaled-up reaction.

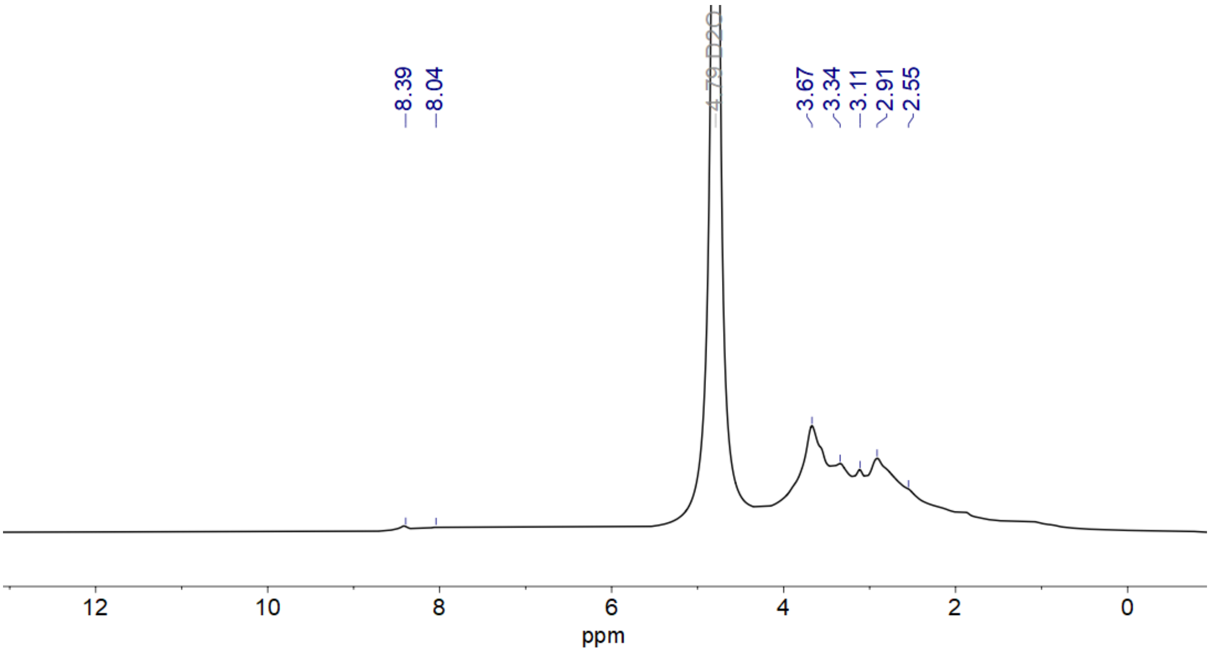

Supplementary Figure 132 ^{1}H NMR (500 MHz, D_2O) spectrum of product obtained from scaled-up reaction.

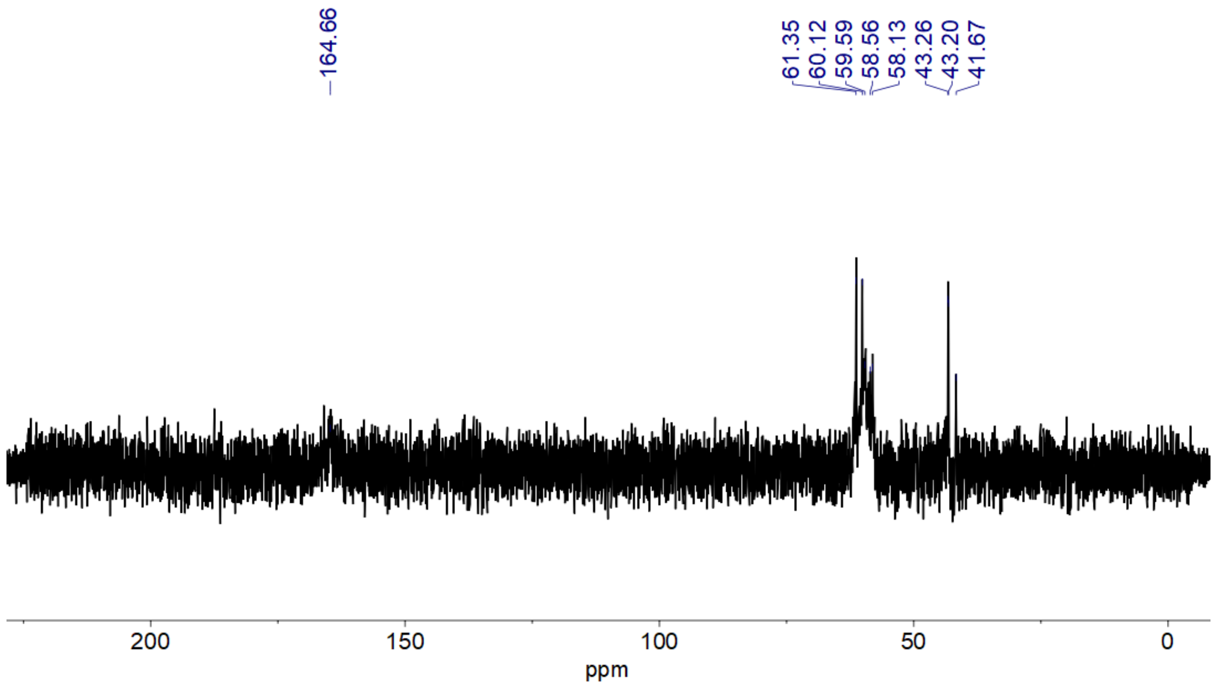

Supplementary Figure 133 $^{13}C\{^1H\}$ NMR (126 MHz, D_2O) spectrum of product obtained from scaled-up reaction.

1.10 Quantatifying Primary Amines (quantitative $^{13}C\{^1H\}$ NMR and UV-Vis experiments)

Quantification of the end groups present within PEI-1 presents a challenge. We have used a combination of quantitative $^{13}C\{^1H\}$ NMR (d_1 = 30s, ns = 3,200) spectroscopy and well documented reaction between amines (primary and secondary) with ninhydrin (Supplementary Supplementary Figure ***134***).

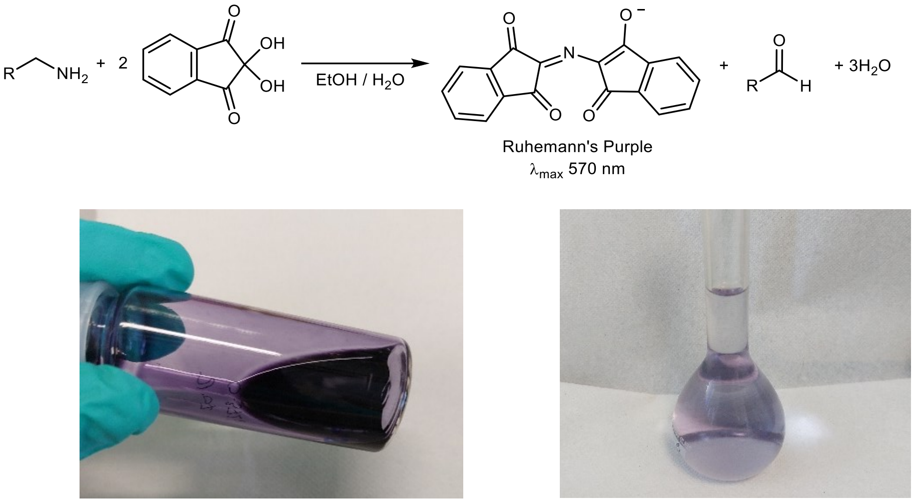

Supplementary Figure 134. Formation of Ruhemann's purples from amine and ninhydrin and images of examples of the colour observed within this study using u-**PEI-1** (43.02 µM, LHS and 0.258 µM RHS).

The resolution obtained in these quantitative $^{13}C\{^1H\}$ NMR spectra is improved on those obtained using standard cross-polarisation $^{13}C\{^1H\}$ NMR parameters (*e.g.* d_1 = 2 s, ns = 800). Indeed, we can now resolve the (previously poorly resolved) signals associated with C̲-OH and C̲-3°N along with quantification of the degree of branching present within the polymer (for instance, see inset in **Supplementary Supplementary Figure *138***).

Procedure for the reaction between amines and ninhydrin:

An aquesous solution of the amine of interest was prepared in distilled water. To this solution was added a solution of ninhydrin (0.5170 M in EtOH) such that the resulting H_2O:EtOH = 1:9.3. [For instance, *N*-(2-hydroxyethyl)ethylenediamine (21.7 mg, 0.2084 mmol) is dissolved in 0.3723 mL H_2O and ninydrin solution (3.440 mL, 0.5170 M in EtOH, 1.778 mmol) added.]

**Sample**	**Weight /mg**	**H_2O / g**	**Ninhydrin (0.5170 M in EtOH) / mL**
PEI-1	7.2	0.3080	2.860
u-PEI-1	7.7	0.4332	4.030

The resulting solution is heated to 60 °C for 90 minutes during which time a deep purple colour is observed. This solution is then diluted with a 1:9.3 H_2O:EtOH solution and the resulting solution of known polymer concentration interrogated by UV-Vis spectroscopic analysis. The results used in conjunction with the below calibration to determine the concentration of primary amines present within the sample.

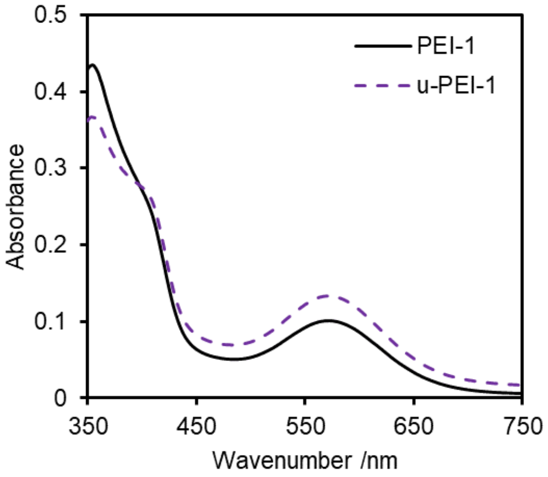

Supplementary Figure 135 UV-vis spectra obtained of Ruhemann's purple containing solutions after reaction of PEI-1 and u-PEI-1 with ninhydrin.

	UV-Vis						*^{13}C NMR*
**Sample**	**MW[a] / g mol^{-1}**	**pol. conc. / µM**	**λ_{570nm} /A**	**[1° amine] /mM**	**~number 1° amine**	**~1° amine /wt%**	**1° amine[b] / %**
PEI-1[c]	38,700	0.470	0.101	0.098	141	16	38
u-PEI-1[d]	40,100	0.258	0.133	0.130	17	2	21
PEI-2[e]							5

[a] Measured by GPC. [b] Determined by quantitative $^{13}C\{^1H\}$ NMR spectroscopy (d_1 = 30 s, ns = 3,200). [c] From optimised conditions. [d] From scaled-up procedure. [e] From **Mn-2** catalyst. The average value of primary amine concentrations using quantitative $^{13}C\{^1H\}$ NMR (38%) and UV-vis (16%) techniques have been used for discussion in the paper.

1.10.1 Calibration using N-(2-hydroxyethyl)ethylenediamine:

N-(2-hydroxyethyl)ethylenediamine is used as a surrogate amine to allow a calibration of [Ruhemann's purple] *vs* absorbance to be generated. The results of this calibration are shown in **Supplementary Supplementary Figure** ***136***.

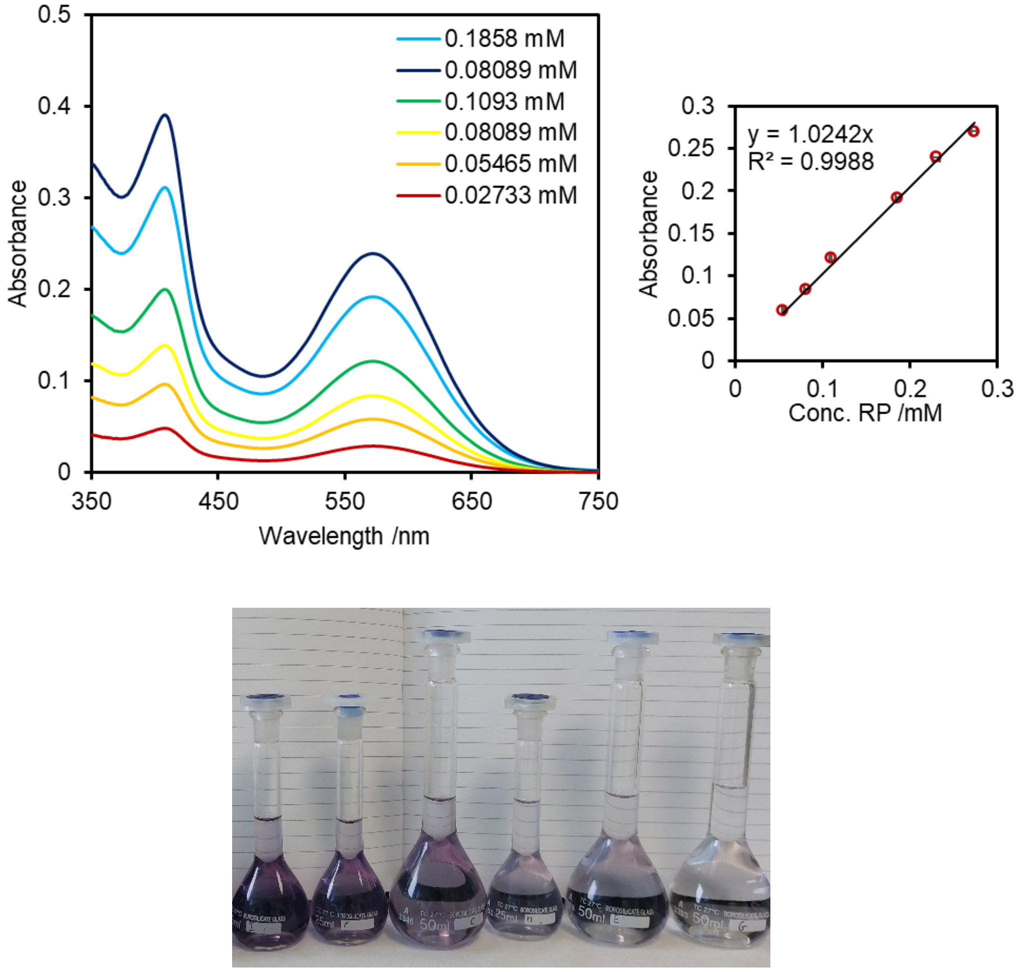

Supplementary Figure 136 UV-Vis spectra of *N*-(2-hydroxyethyl)ethylenediamine solutions and resulting relationship between [ruhemann's purple, RP] and absorption obtained from the reaction of ninhydrin with *N*-(2-hydroxyethyl)ethylenediamine.

1.10.2 Quantitative $^{13}C\{^{1}H\}$ NMR spectra

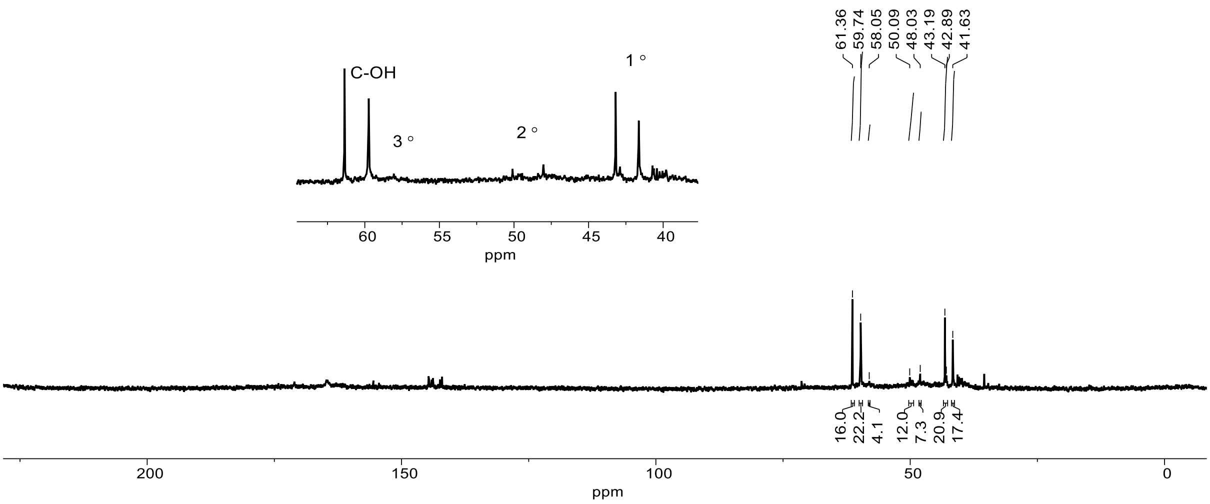

Supplementary Figure 137 Quantitative $^{13}C\{^{1}H\}$ NMR spectrum obtained of **PEI-1** generated using our optimised procedure.

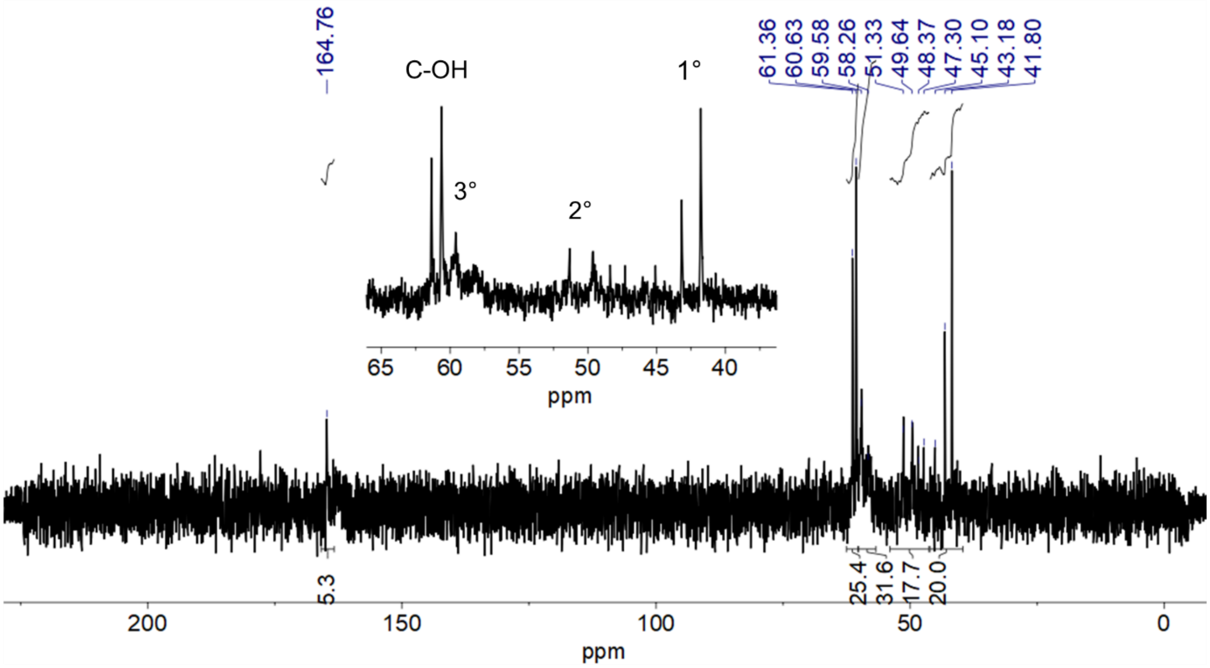

Supplementary Figure 138 Quantitative $^{13}C\{^{1}H\}$ NMR spectrum obtained of the **u-PEI-1** generated using our scaled up procedure.

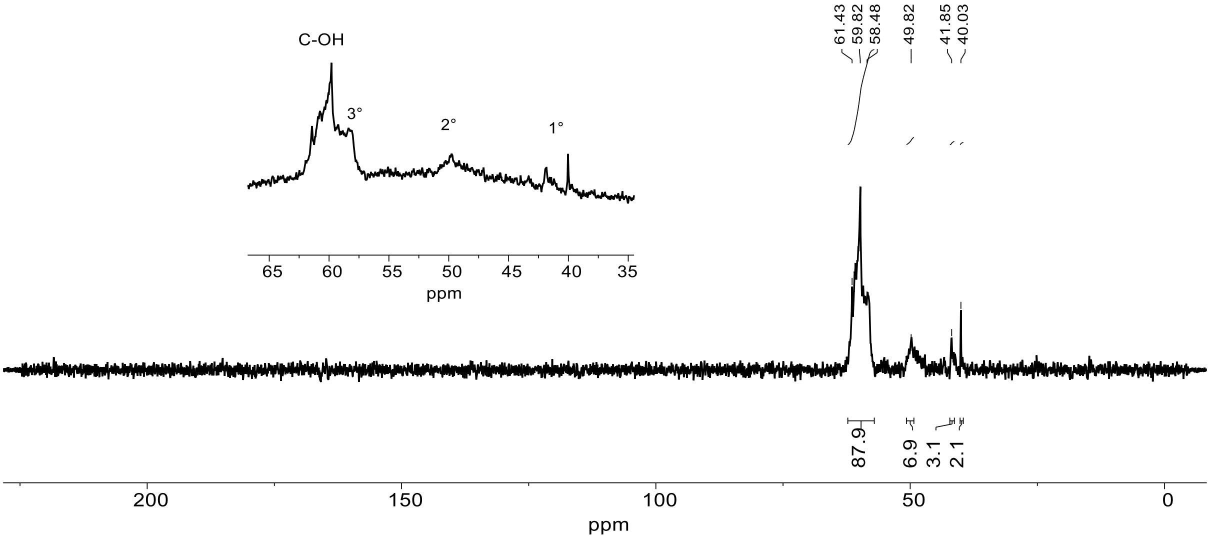

Supplementary Figure 139 Quantitative $^{13}C\{^{1}H\}$ NMR spectrum obtained of the polymer generated using **Mn-2** catalyst.

1.11 Substrate Scope

Supplementary Table 4 Amino alcohol dehydrogenation facilitated by **Mn-1**.[a]

Entry	Amino alcohol	Product(s)	Conv.[b] % (yield)	Yield[c] /%
1	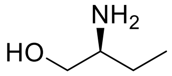	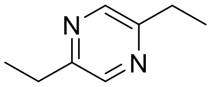	100	12
2	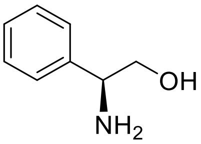	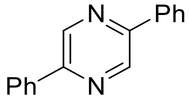	100	36
3	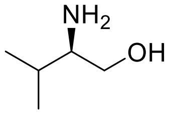	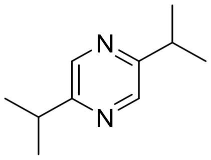	100	10 °
4	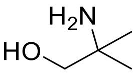	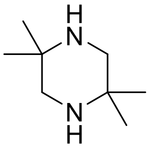 **4a** 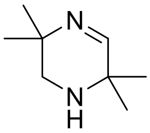 **4b** 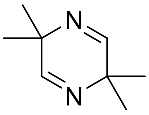 **4c**	100	**4a** 38<br>**4b** 12<br>**4c** 45
5	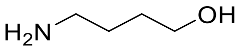	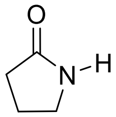 **5a** 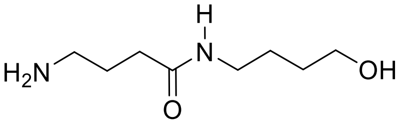 **5b**	100	**5a** 56<br>**5b** 10
6	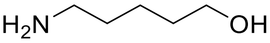	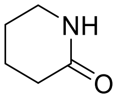 **6a** 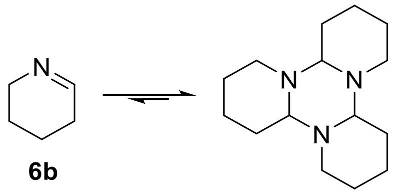 **6b** **6c**	100	**6a** 18[e]<br>**6b**/**6c** 82[e]

[a] Experimental conditions: amino alcohol (2 mmol), **Mn-1** (1 mol%), KO^{t}Bu (10 mol%), toluene (4 mL), 150 °C, 24 h; [b] Determined by GC-MS; [c] NMR yield ($CDCl_3$) relative to 1,1-diphenylethylene internal standard; [d]NMR yield determination aided by peak deconvolution (see Supplementary Supplementary Figure *167*); [e] Determined by GCMS due to overlapping signals obfuscating ^{1}H NMR spectrum integration.

1.11.1 GC-MS Spectra

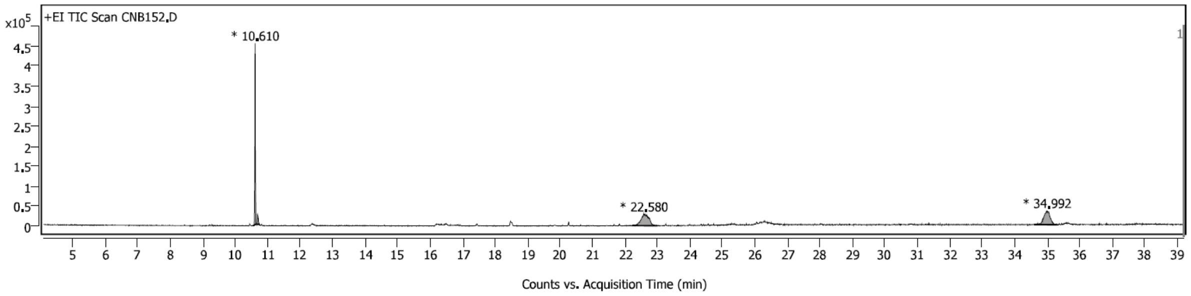

Chromatogram Peaks

Peak	Start	RT	End	Height	Area	Area %	SNR
1	10.570	10.610	10.650	453767	646715	100.00	
2	10.673	10.690	10.747	23205	41228	6.37	
3	22.220	22.580	22.998	28799	490429	75.83	
4	34.585	34.992	35.301	34670	464410	71.81	

Supplementary Figure 140 GC spectra of products obtained from the dehydrogenation of 2-amino-1-butanol.

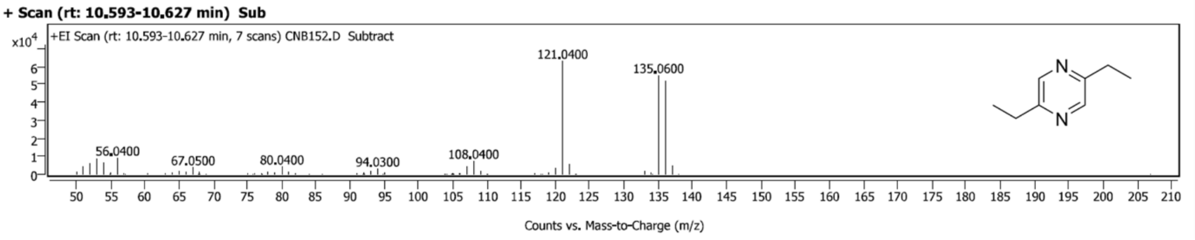

Supplementary Figure 141 MS spectra of 2,5-diethylpyrazine obtained from the dehydrogenation/dehydration of 2-amino-1-butanol.

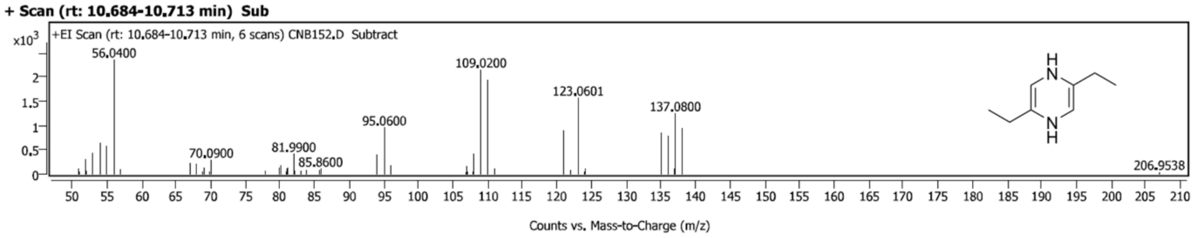

Supplementary Figure 142 MS spectra of 2,5-diethyl-1,4-dihydropyrazine obtained from the dehydrogenation/dehydration of 2-amino-1-butanol.

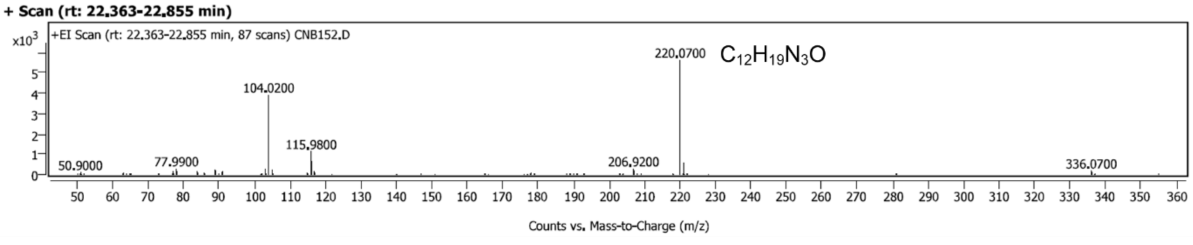

Supplementary Figure 143 MS spectra of trimeric product formed from the dehydrogenation/dehydration of 2-amino-1-butanol.

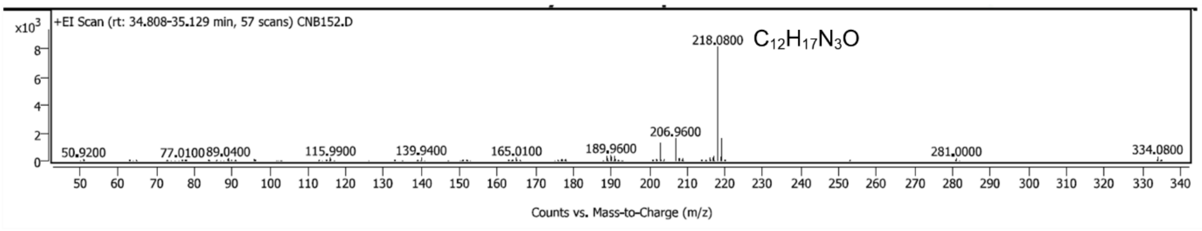

Supplementary Figure 144 MS spectra of trimeric product formed from the dehydrogenation/dehydration of 2-amino-1-butanol.

Sample Chromatograms

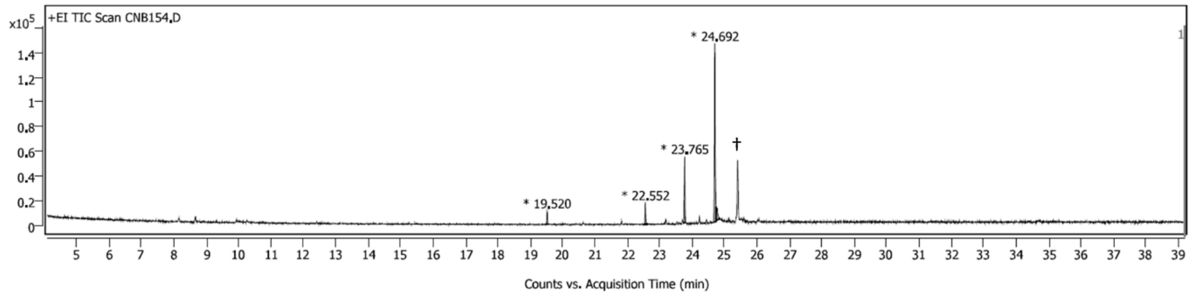

Chromatogram Peaks

Peak	Start	RT	End	Height	Area	Area %	SNR
1	19.497	19.520	19.554	10825	15941	5.72	
2	22.524	22.552	22.610	17311	28099	10.09	
3	23.731	23.765	23.800	53381	78579	28.21	
4	24.658	24.692	24.738	143722	278565	100.00	

Supplementary Figure 145 GC obtained of product mixture from the dehydrogenation/dehydration of (R)-2-phenylglycinol. †denotes impurity present within the starting material.

+ Scan (rt: 19.514-19.531 min) Sub

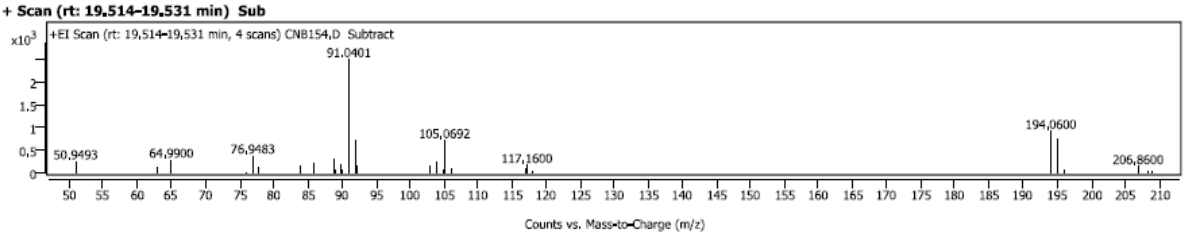

Supplementary Figure 146 MS spectra of unidentified product obtained from the dehydrogenation/dehydration of (R)-2-phenylglycinol.

+ Scan (rt: 22.535-22.564 min) Sub

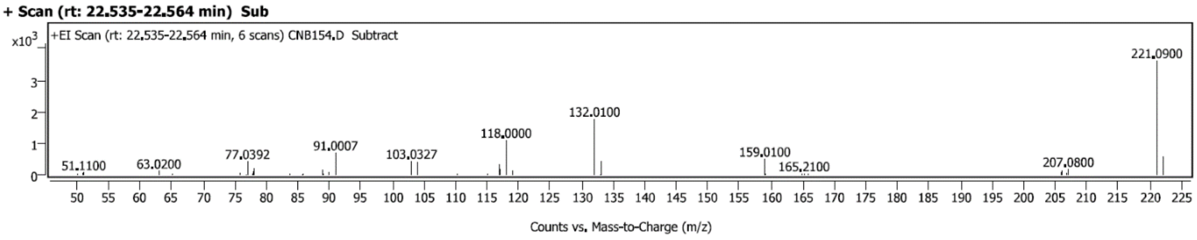

Supplementary Figure 147 MS spectra of unidentified product obtained from the dehydrogenation/dehydration of (R)-2-phenylglycinol.

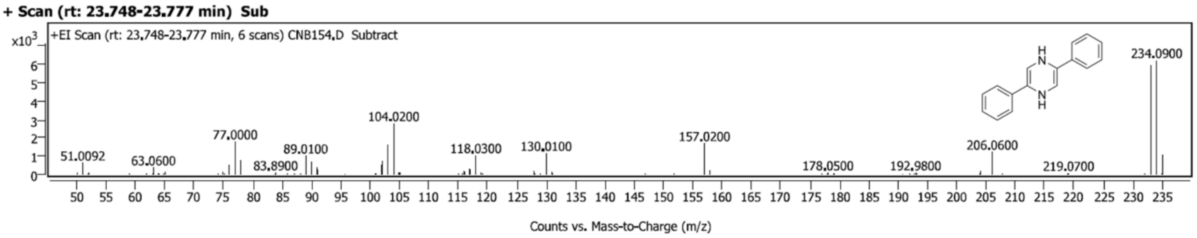

Supplementary Figure 148 MS spectra of 2,5-diphenyl-1,4-dihydropyrazine obtained from the dehydrogenation/dehydration of (R)-2-phenylglycinol.

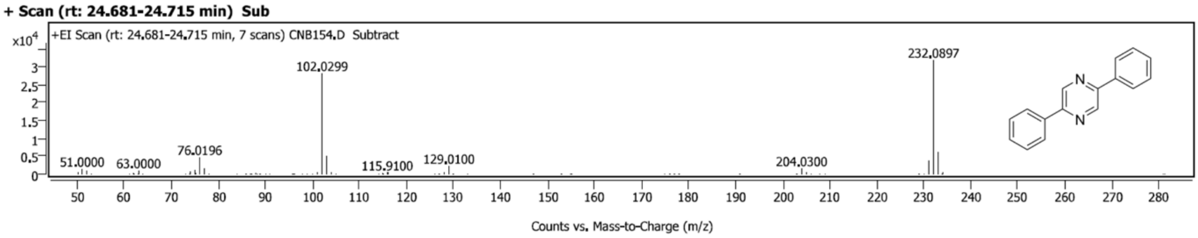

Supplementary Figure 149 MS spectra of 2,5-diphenylpyrazine obtained from the dehydrogenation/dehydration of (R)-2-phenylglycinol.

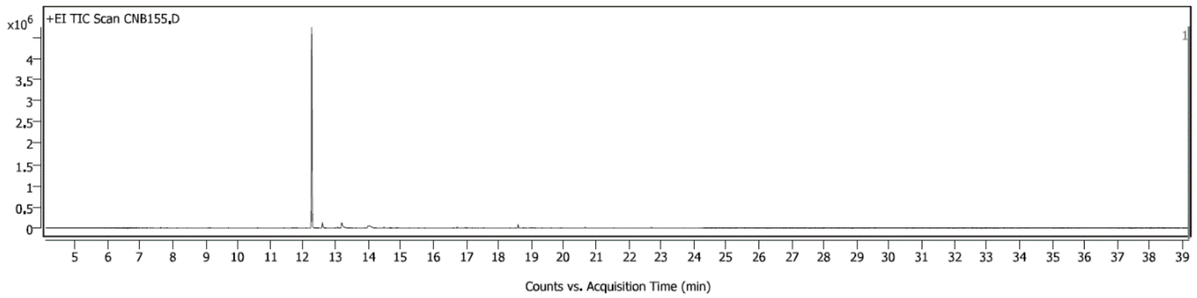

Supplementary Figure 150 GC spectra obtained from the dehydrogenation/dehydration of (S)-(+)-2-amino-3-methyl-1-butanol.

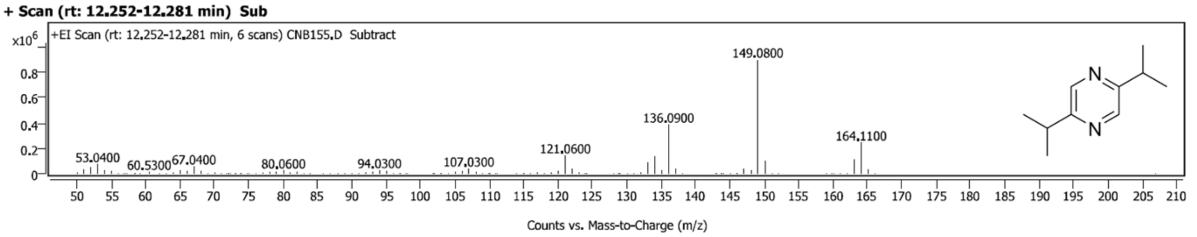

Supplementary Figure 151 MS spectra of 2,5-diisopropylpyrazine obtained from the dehydrogenation/dehydration of (S)-(+)-2-amino-3-methyl-1-butanol.

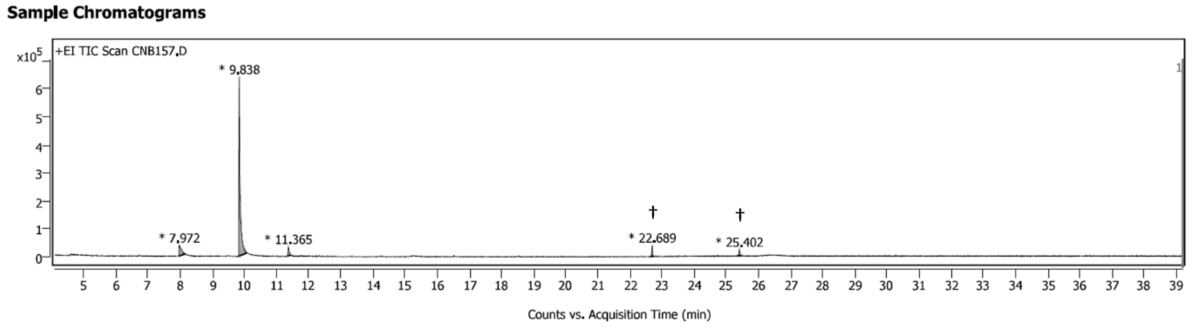

Chromatogram Peaks

Peak	Start	RT	End	Height	Area	Area %	SNR
1	7.955	7.972	8.155	37981	201445	11.27	
2	9.815	9.838	10.078	639345	1786689	100.00	
3	11.343	11.365	11.451	32620	89442	5.01	
4	22.655	22.689	22.747	40635	59799	3.35	
5	25.333	25.402	25.482	22688	49587	2.78	

Supplementary Figure 152 GC spectra of products obtained from the dehydrogenation/dehydration of 2-amino-2-methyl-1-propanol. †denotes signals corresponding to column bleed.

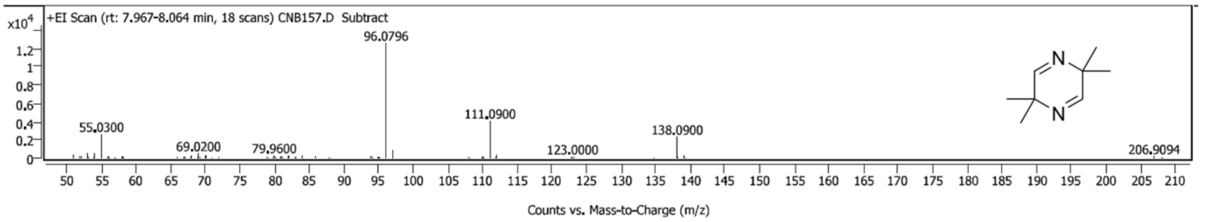

Supplementary Figure 153 MS spectra corresponding to 2,2,5,5-tetramethylpyrazine obtained from the dehydrogenation/dehydration of 2-amino-2-methyl-1-propanol.

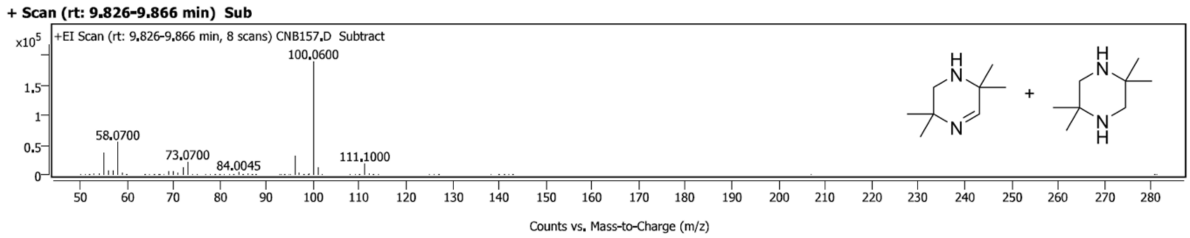

Supplementary Figure 154 MS spectra corresponding to 2,2,5,5-tetramethyl-1,4-dihydropyrazine and 2,2,5,5-tetramethyl-1-hydropyrazine obtained from the dehydrogenation/dehydration of 2-amino-2-methyl-1-propanol.

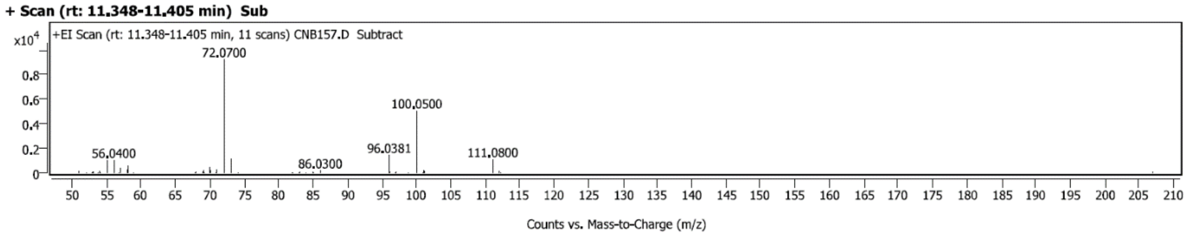

Supplementary Figure 155 MS spectra corresponding to unidentified product obtained from the dehydrogenation/dehydration of 2-amino-2-methyl-1-propanol.

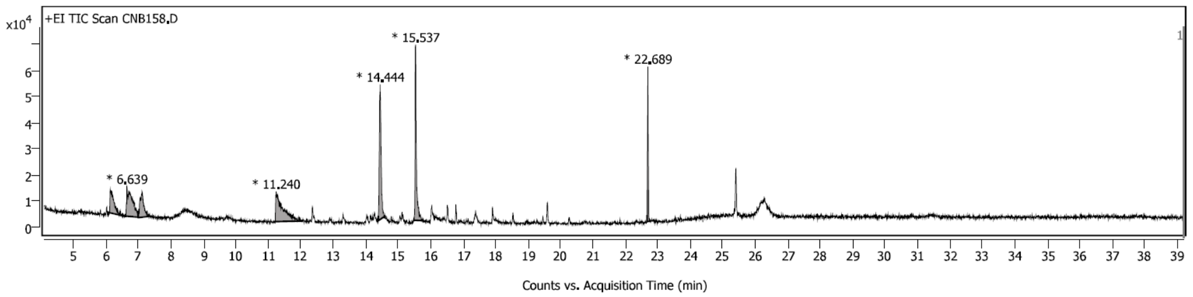

Chromatogram Peaks

Peak	Start	RT	End	Height	Area	Area %	SNR
1	6.044	6.130	6.548	9017	74497	37.02	
2	6.610	6.639	6.982	11419	130096	64.65	
3	7.000	7.108	7.412	10022	79075	39.30	
4	11.182	11.240	12.069	11533	173966	86.45	
5	14.387	14.444	14.610	51822	201228	100.00	
6	15.457	15.537	15.771	67371	181058	89.98	
7	22.667	22.689	22.781	59522	91350	45.40	

Supplementary Figure 156 GC spectra corresponding to product mixture obtained from the dehydrogenation/dehydration of 3-amino-1-propanol.

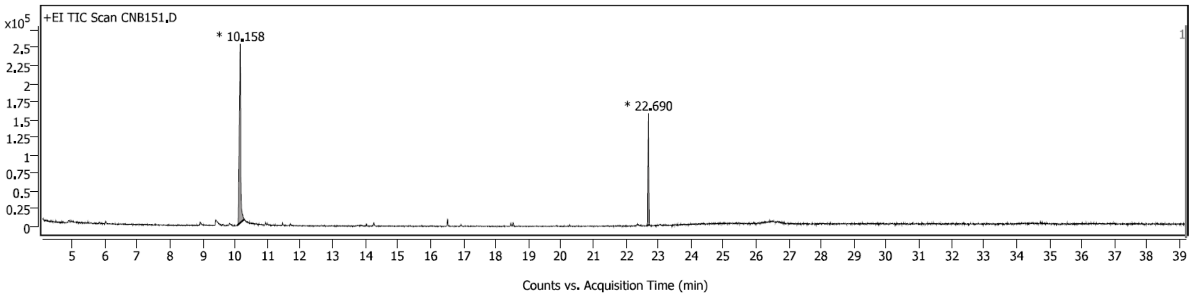

Chromatogram Peaks

Peak	Start	RT	End	Height	Area	Area %	SNR
1	10.084	10.158	10.284	249928	723085	100.00	
2	22.655	22.690	22.730	156342	239185	33.08	

Supplementary Figure 157 GC spectra of products from the dehydrogenation of 4-amino-1-butanol.

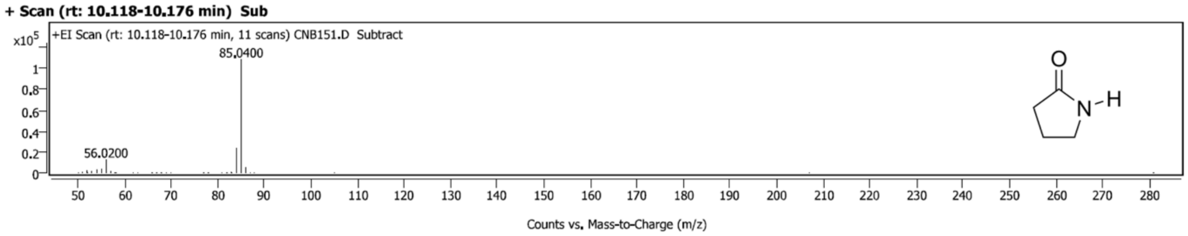

Supplementary Figure 158 MS (EI) spectrum of β-lactam obtained from the dehydrogenation/dehydration of 4-amino-1-butanol.

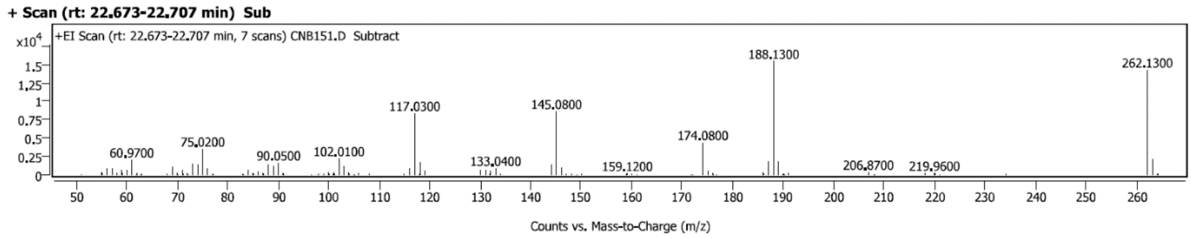

Supplementary Figure 159 MS (EI) spectrum of dimer product obtained from the dehydrogenation/dehydration of 4-amino-1-butanol.

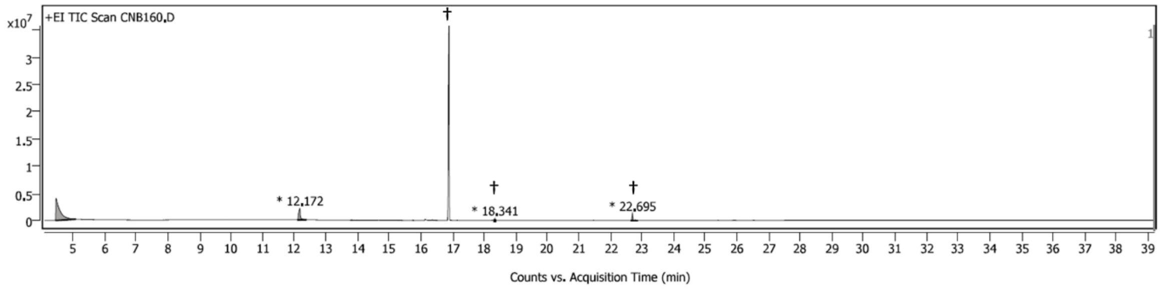

Chromatogram Peaks

Peak	Start	RT	End	Height	Area	Area %	SNR
1	4.442	4.459	5.088	4083016	37782929	100.00	
2	12.092	12.172	12.384	2114113	8454585	22.38	
3	18.306	18.341	18.381	395558	537785	1.42	
4	22.655	22.695	22.855	1234524	2254783	5.97	

Supplementary Figure 160 GC spectrum obtained of products from dehydrogenation/dehydration of 5-amino-1-pentanol. †denotes signals arising from internal standard, 1,1'-diphenylethylene and impurities resulting from this compound.

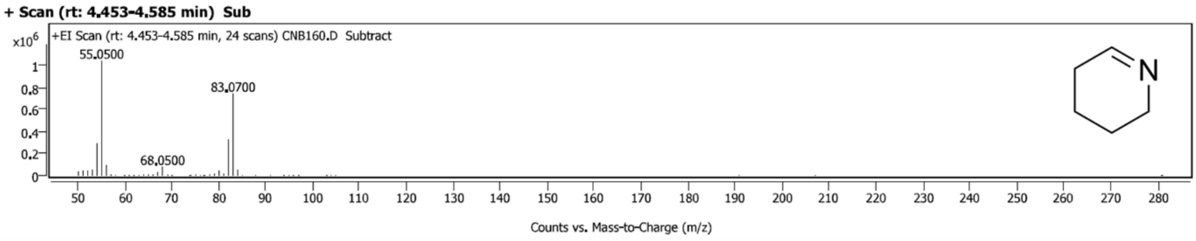

Supplementary Figure 161 MS spectrum of 2,3,4,5-tetrahydropyridine obtained from the dehydrogenation/dehydration of 5-amino-1-pentanol.

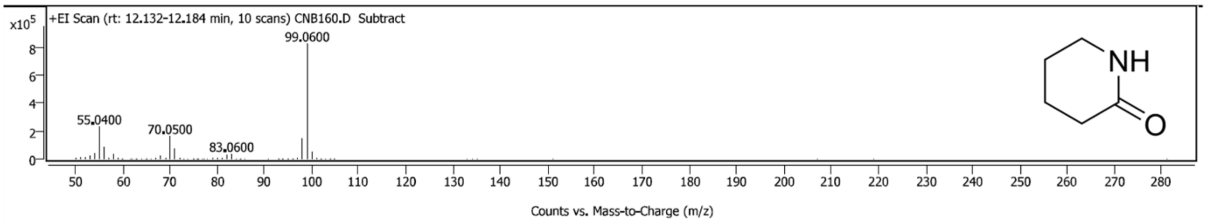

Supplementary Figure 162 MS spectrum of δ-lactam obtained from the dehydrogenation/dehydration of 5-amino-1-pentanol.

1.11.2 NMR Spectra

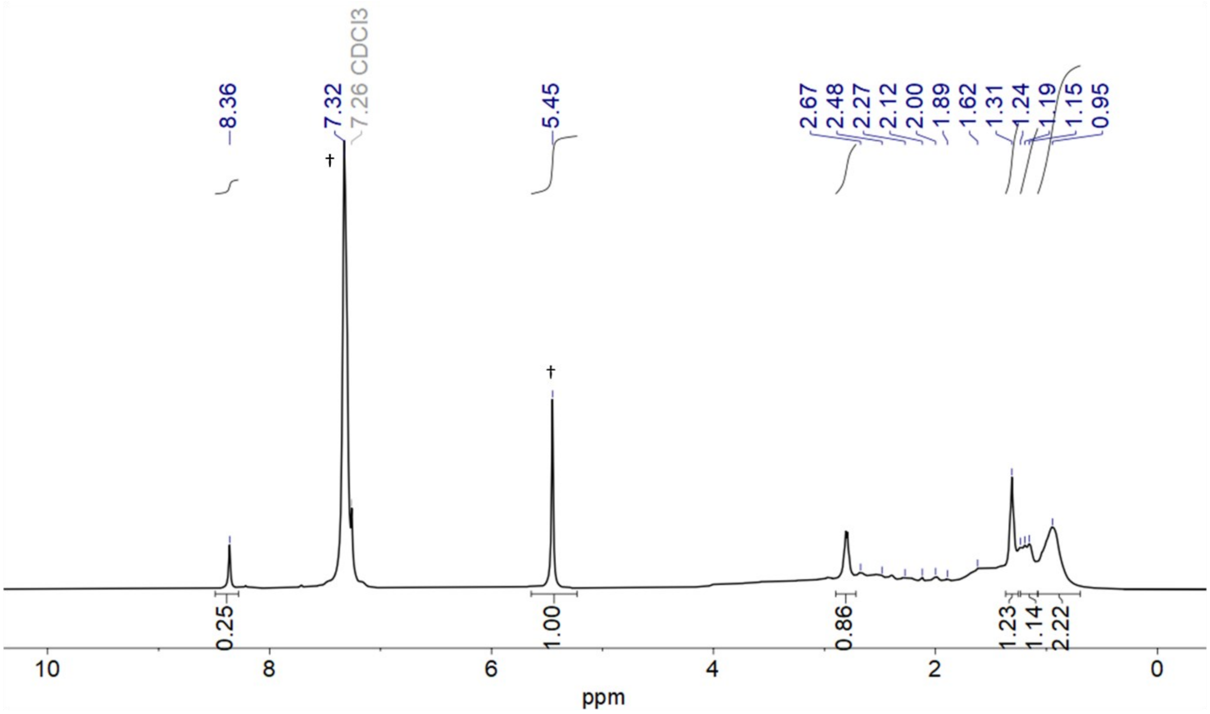

Supplementary Figure 163 ^{1}H NMR (500 MHz, $CDCl_3$) spectrum obtained from the dehydrogenation of 2-amino-1-butanol. † denotes 1,1'-diphenylethylene internal standard.

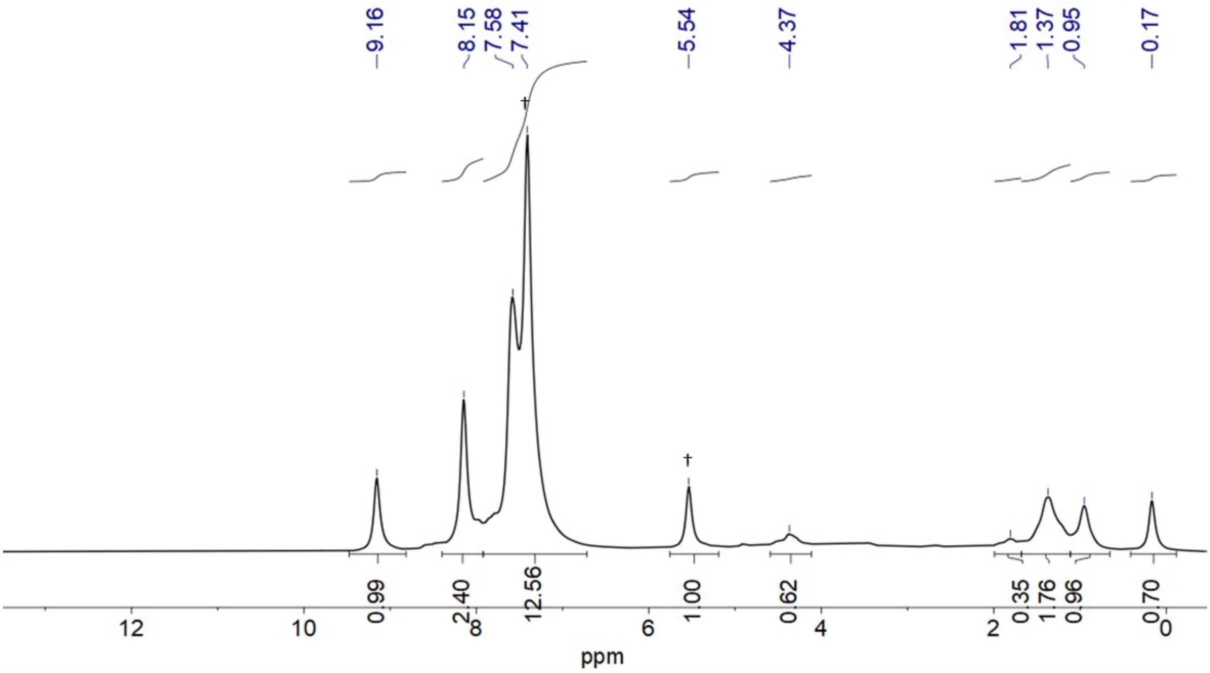

Supplementary Figure 164 ^{1}H NMR (500 MHz, $CDCl_3$) spectrum obtained from the dehydrogenation of (R)-2-phenylglycinol. † denotes 1,1'-diphenylethylene internal standard.

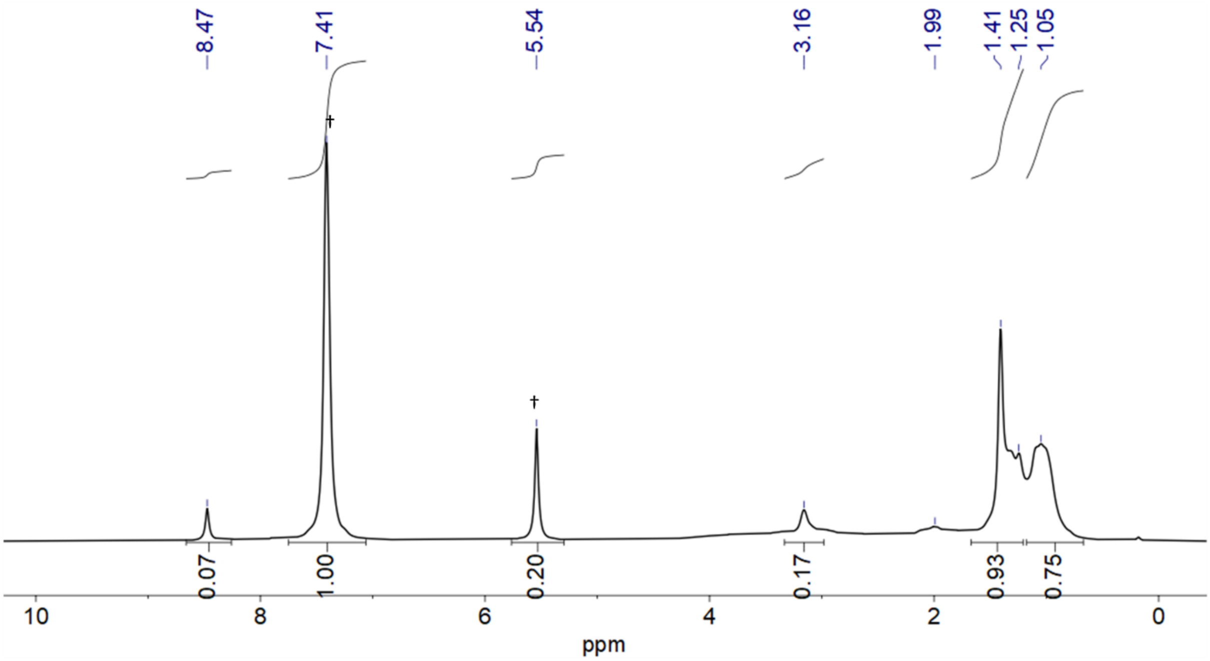

Supplementary Figure 165 ^{1}H NMR (500 MHz, $CDCl_3$) spectrum obtained from the dehydrogenation of (S)-(+)-2-amino-3-methyl-1-butanol. † denotes 1,1'-diphenylethylene internal standard.

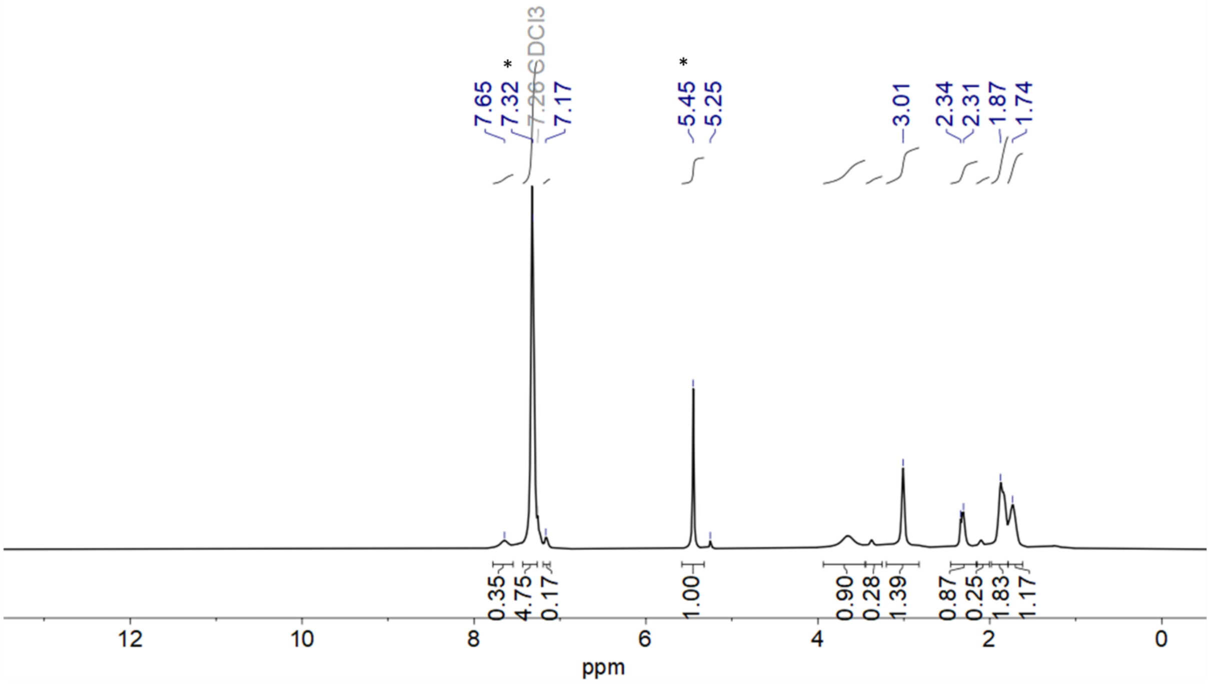

Supplementary Figure 166 ^{1}H NMR (500 MHz, $CDCl_3$) spectrum obtained from the dehydrogenation of 4-amino-1-butanol. * indicates 1,1'-diphenylethylene internal standard.

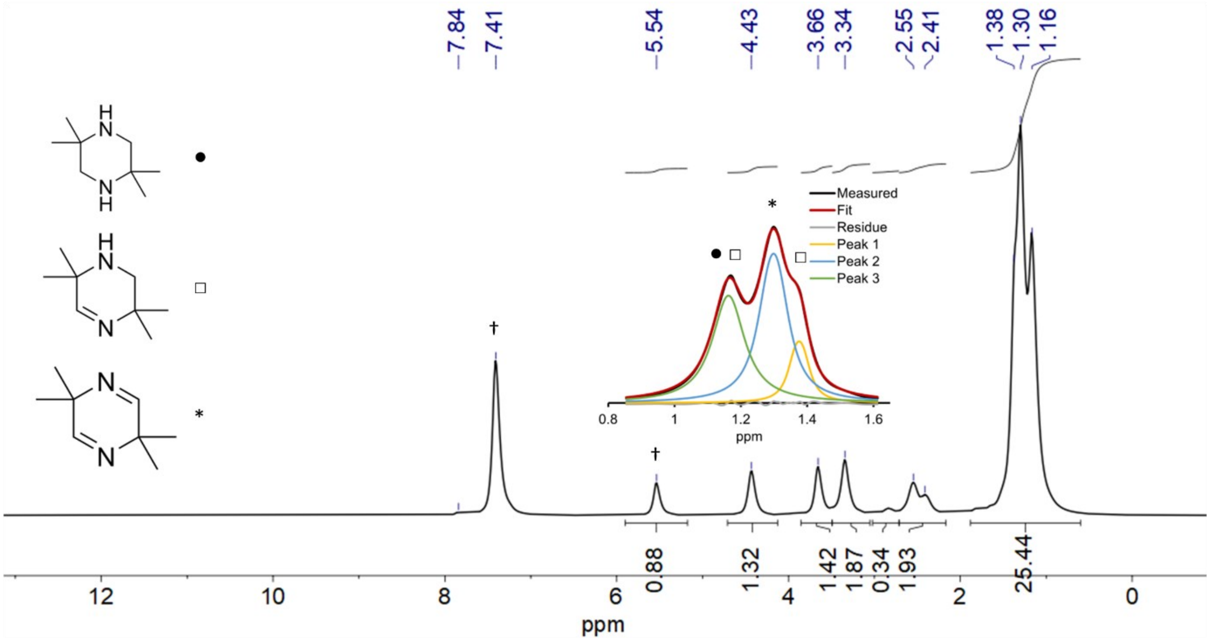

Supplementary Figure 167 ^{1}H NMR (500 MHz, $CDCl_3$) spectrum obtained from the dehydrogenation of 4-amino-butan-1-ol. † denotes 1,1'-diphenylethylene internal standard.

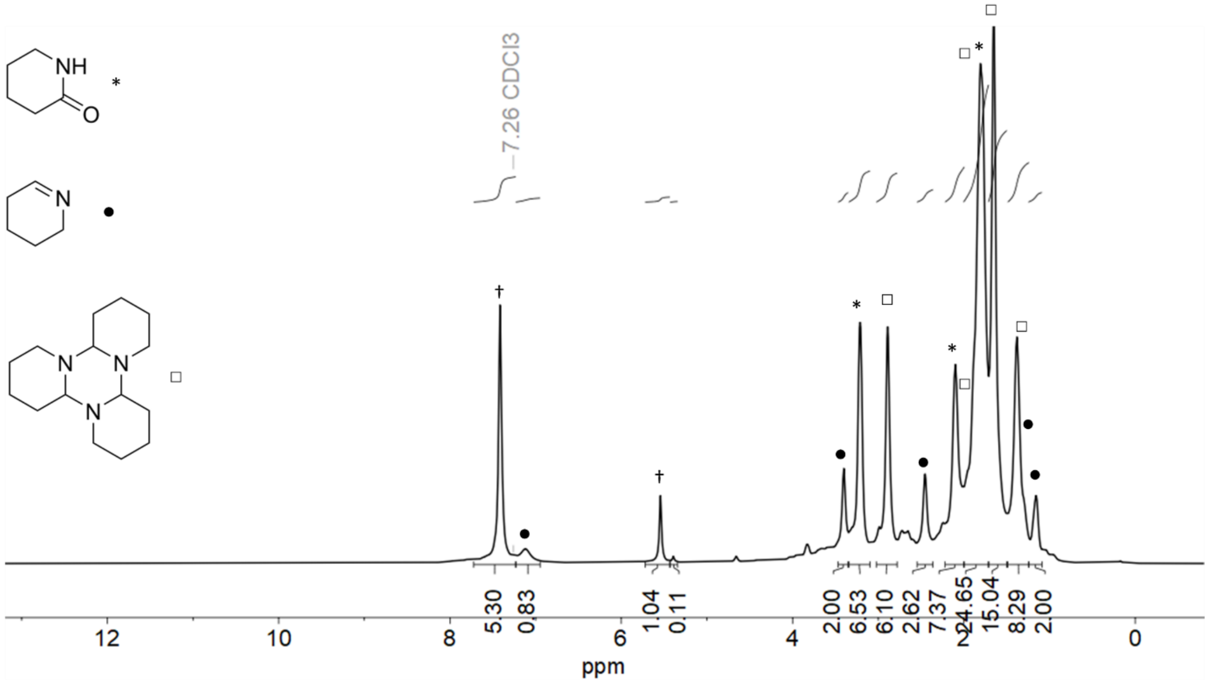

Supplementary Figure 168 ^{1}H NMR (500 MHz, $CDCl_3$) spectrum obtained from the dehydrogenation of 5-amino-1-pentanol. †denotes 1,1'-diphenylethylene internal standard.

1.12 Expanded Substrate Scope

An expanded substrate scope was carried out following the general procedure using either 1-amino-2-hydroxypropane or 6-aminohexanol. The product was extracted into distilled water (5 mL) and any volatile components were removed under reduced pressure at 110 °C. Both substrates yielded a complex mixture of unidentifiable products (as observed in GC-MS) along with a polymeric material

(containing imine, amine and amide groups) in low yield (1-amino-2-hydroxypropane; 38% yield, 6-aminohexanol; 21% yield)

<u>Polymerisation of 1-amino-2-hydroxypropane.</u>

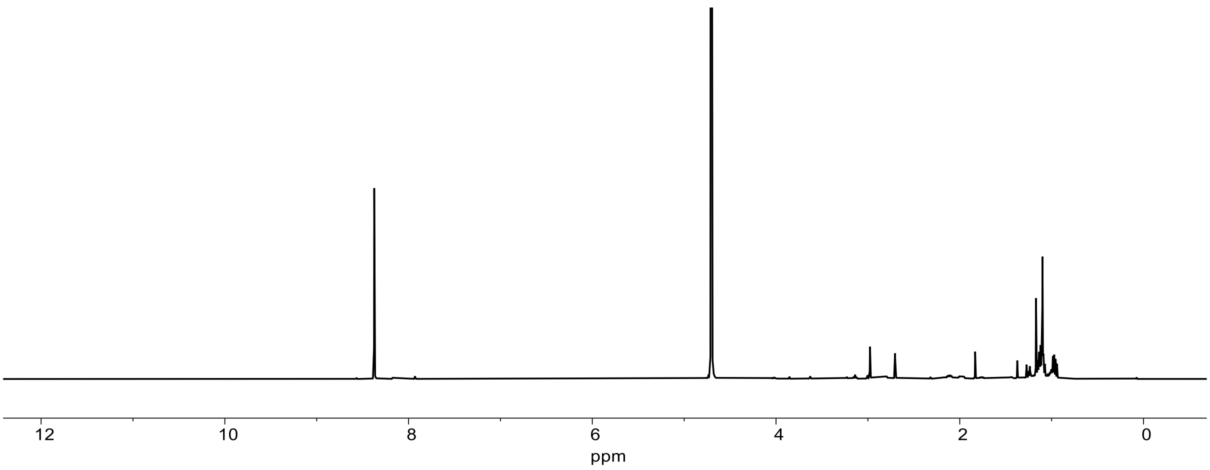

Supplementary Figure 169 ^{1}H NMR (500 MHz, D_2O) spectrum of products of 1-amino-2-hydroxypropane polymerisation.

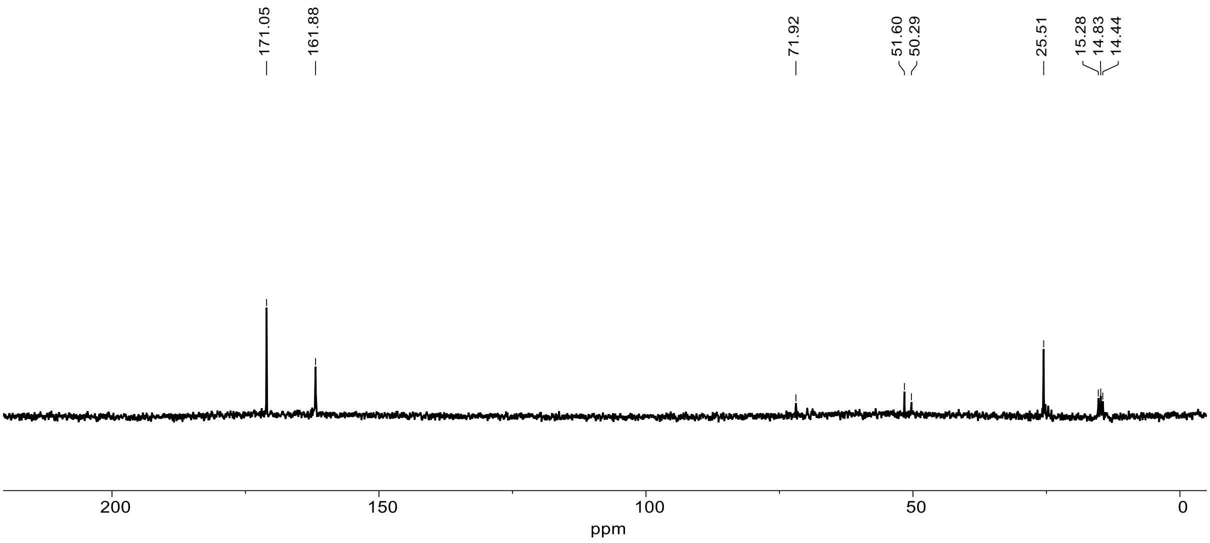

Supplementary Figure 170 $^{31}C\{^{1}H\}$ NMR (126 MHz, D_2O) spectrum of products of 1-amino-2-hydroxypropane polymerisation.

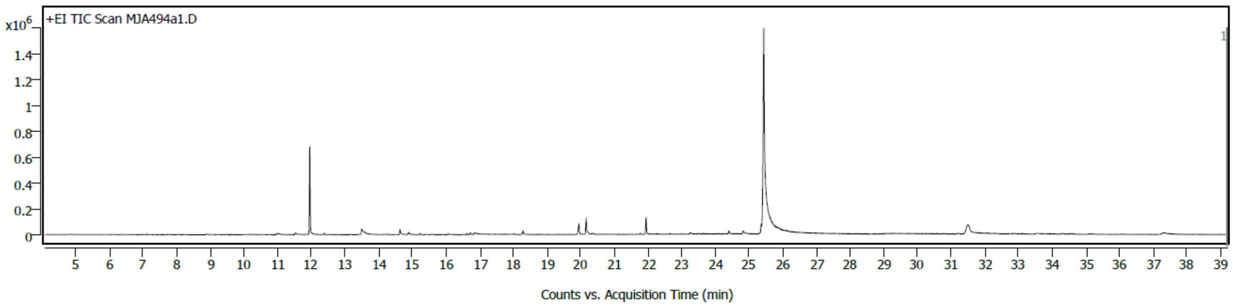

Supplementary Figure 171 GC data of products of 1-amino-2-hydroxypropane polymerisation.

GPC (H_2O, 30 °C, g mol^{-1}): 12,300 (Đ 1.16).

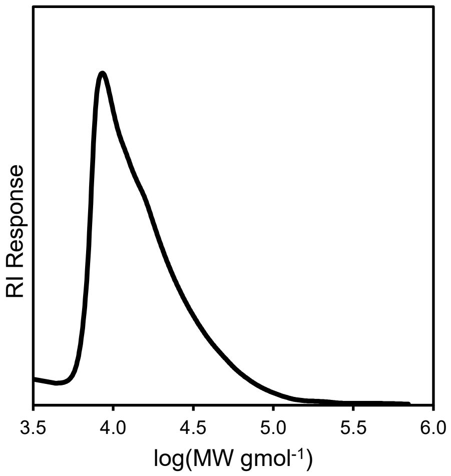

Supplementary Figure 172 GPC chromatograph of product corresponding to polymerisation of 1-amino-2-hydroxypropane.

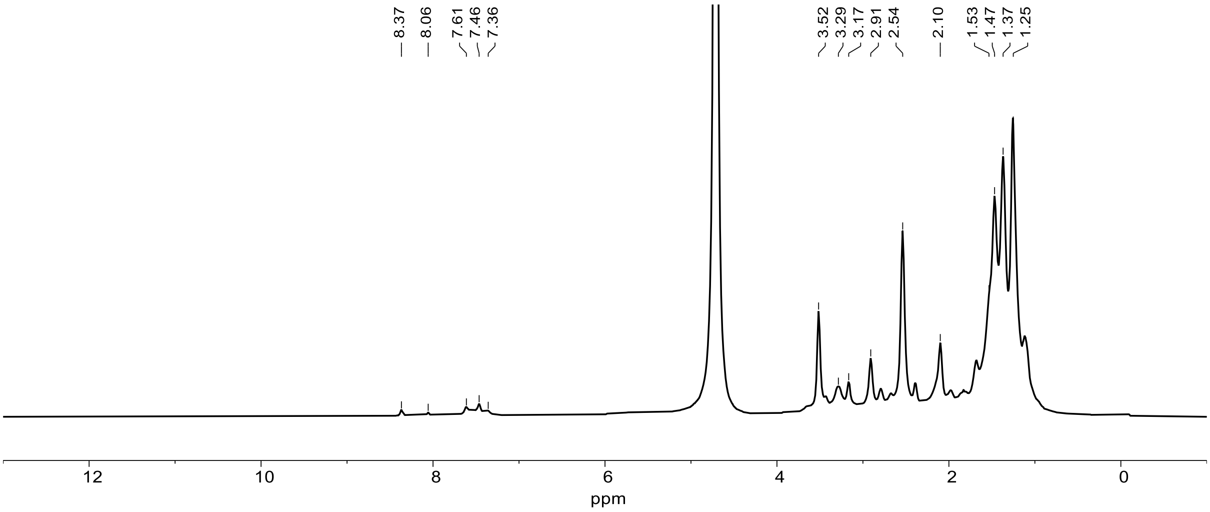

Supplementary Figure 173 ^{1}H NMR (500 MHz, D_2O) spectrum of products of 6-aminohexanol polymerisation.

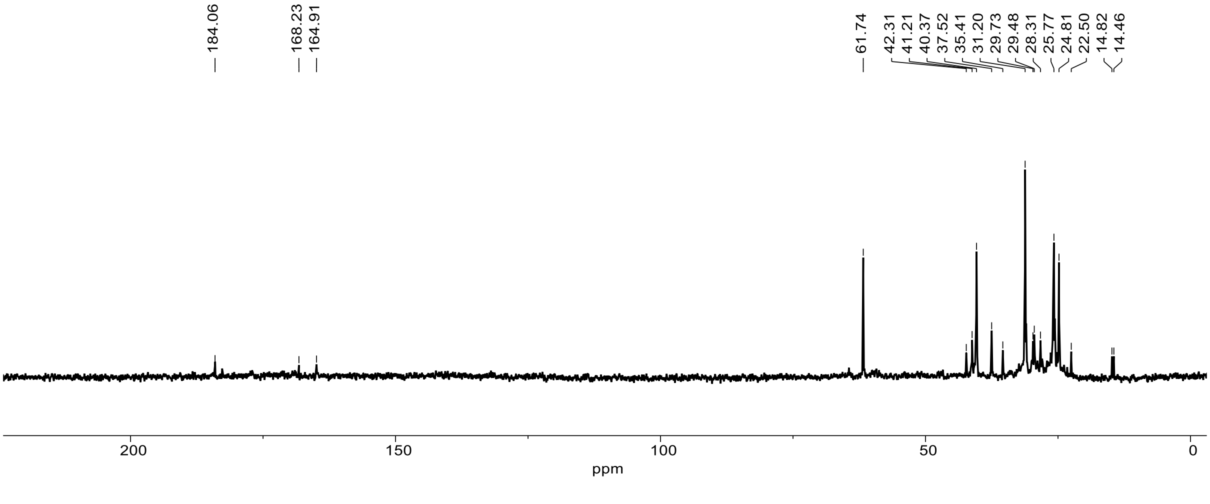

Supplementary Figure 174 $^{31}C\{^{1}H\}$ NMR (126 MHz, D_2O) spectrum of products of 6-aminohexanol polymerisation.

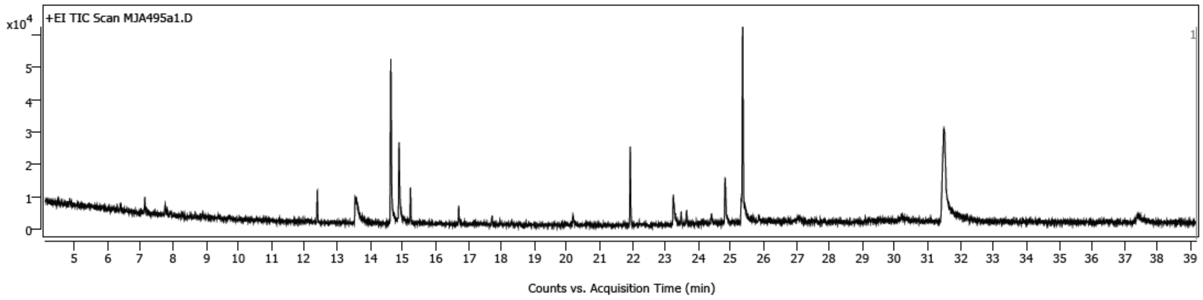

Supplementary Figure 175 GC data of products of 6-aminohexanol polymerisation.

GPC (H_2O, 30 °C, g mol^{-1}): 43,000 (Đ 1.74)

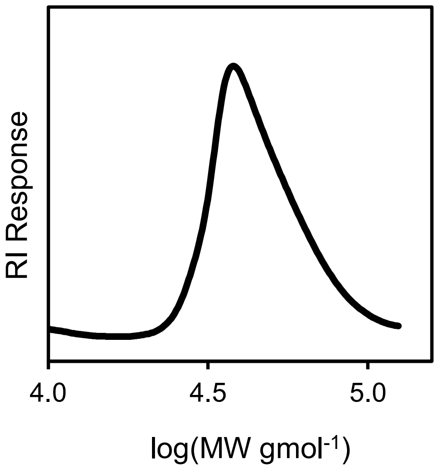

Supplementary Figure 176 GPC chromatograph of product corresponding to polymerisation of 6-aminohexanol.

2. Computational Details

2.1 General Considerations

DFT calculations were performed using the PBE0-D3(BJ)$_{PCM(THF)}$/def2-TZVP//RI-BP86$_{PCM(THF)}$/def2-SVP level of theory with Gaussian16, C.01.[S3] This is the same level as used in previous work from the group,[S4–S8] benchmarked against a series of experimental 3d transition metal hydride bond strengths[S9] and including Martin, Hay and Pratt empirical entropy corrections[S10] (*i.e.* 5.09 kcal mol^{-1} per particle[S4]). We note that the choice of solvent between THF (consistent with previous work) and toluene (used experimentally) has little impact upon the overall thermodynamics (**Supplementary Supplementary Figure *179***) and for continuity we retain THF.

As the chain length increases during condensation of monomers, the intermediates have an ever increasing conformational flexibility. This problem was addressed by using Stefan Grimme's CREST program,[S11] to search for all possible conformers at the GFN2-xTB level.[S12] Following a meta-dynamics run with an 8 kcal mol^{-1} cutoff, full DFT calculations were performed on each conformer to identify the lowest in free energy. With such exhaustive calculations we are confident that the free energy of the lowest DFT conformer is representative and that the profile in **Supplementary Supplementary Figure *177*** is reliable and free of bias toward arbitrary conformers.

Starting structures for Mn complexes were generated from related structures from previous studies (notably with ethylene glycol and ethylene diamine as substrates.[S4] For key transition states, conformational searches were performed with CREST, where the [Mn(MACHO)(CO)$_2$] fragment and transition state atoms were frozen using a set of constraints, to ensure that the same conformation of the pincer ligand was maintained and the transition state was not lost. In these cases, to reduce the dependence on non-equilibrium GFN2-xTB energies (e.g. containing fixed transition state bond lengths), an RMSD matrix was constructed between each conformer from the CREST ensemble and the number of conformers were reduced, with a similarity cutoff, to a tractable number of calculations (~25, following a protocol by Iribarren and Trujillo[S13]). These were subject to a constrained optimisation (with frozen transition state atoms) and the lowest energy conformers (~3-5) were relaxed to obtain formal transition states. Conformational flexibility of Mn complex intermediates (*i.e.* alkoxides) were constructed in the same way and optimised to minima to try to capture the lowest lying structure without the expense of thousands of DFT calculations from the CREST ensemble with a moderate size system.

2.2 Driving forces

The following reaction profiles have been calculated for off-metal thermodynamics, though we note that with a hydrogen borrowing mechanism (*i.e.* reversible H_2 uptake), the formation of the Mn-hydrogenated species is exergonic by $\Delta G = -3.6$ kcal mol^{-1} (**Supplementary Supplementary Figure *181***).

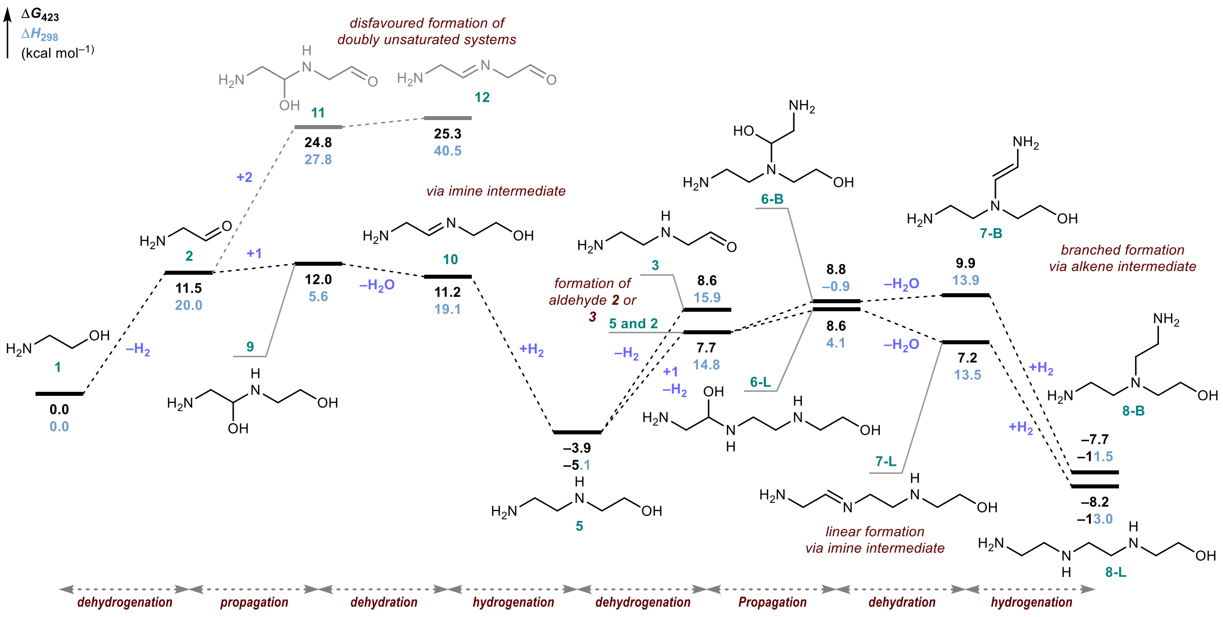

Supplementary Figure 177 Off metal thermodynamic driving forces for the formation of trimer **8-L** from monomer units **1** *via* monomer activation (**1** to **2**).

Formation of trimer **8-L** from dimer **5** may proceed through condensation of the primary amine with monomeric aldehyde **2** (**Supplementary Supplementary Figure *177***), or through dehydrogenation of the alcohol affording the dimer aldehyde **3**, which may react with monomeric **1** (**Supplementary Supplementary Figure *178***).

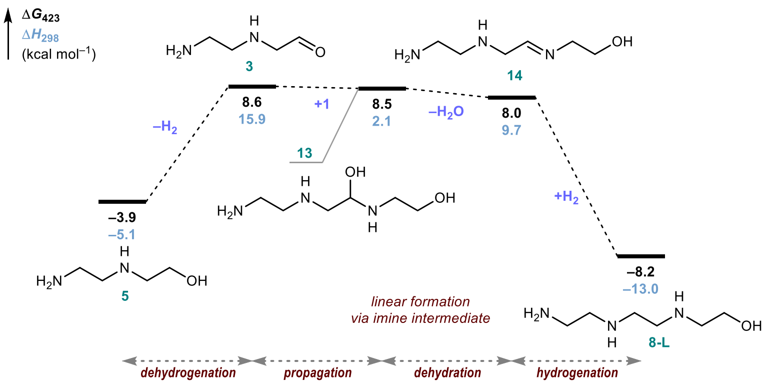

Supplementary Figure 178 Off metal thermodynamic driving force for the formation of trimer **8-L** *via* dimer dehydrogenation (formation of **3**).

The driving forces in the above profile (**Supplementary Supplementary Figure** ***177***) have been recomputed using toluene as an implicit solvent and corresponding Martin, Hay, Pratt correction (**Supplementary Supplementary Figure** ***179***, THF; $\varepsilon = 7.4257$ and toluene; $\varepsilon = 2.3741$; $S_{\mathrm{MHP}} = 4.86$ kcal mol^{-1} per particle). THF has been used to ensure consistency with previous work, allowing for a direct comparison of energetics by using the same methodology. Based on a comparison of the driving forces for trimer formation, we note that these are broadly consistent across the two solvents and that maintaining the THF solvent model is suitable in this case.

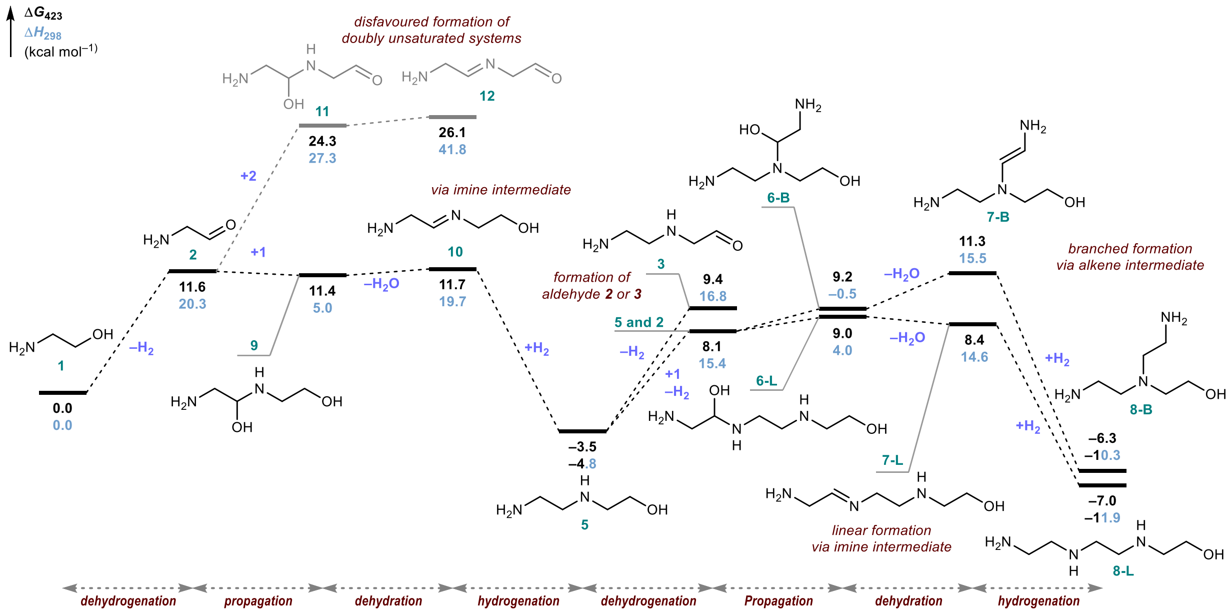

Supplementary Figure 179 Off metal thermodynamic driving forces for the formation of trimer **8-L** from monomer units **1** *via* monomer activation (**1** to **2**) using toluene as a solvent model. DFT at the PBE0-D3(BJ)$_{\mathrm{PCM(toluene)}}$/def2-TZVP//RI-BP86$_{\mathrm{PCM(toluene)}}$/def2-SVP level of theory.

2.3 Barriers for (de)hydrogenation steps

Consistent with previous work, active catalyst **Mn-2** is predicted to OH activate ethanolamine (**1**) in a mildly exergonic reaction. This we predict to be a slightly stabilised off-cycle intermediate ($\Delta G = -1.2$ kcal mol^{-1}) which is in accord with the experimental observation of **Mn-3** in stochiometric reactions at room temperature. This will revert to **Mn-2** for (de)hydrogenations. Similarly, we identify OH activated dimer and trimer species which we calculate to be stabilised by −1.9 and −0.8 kcal mol^{-1}, respectively, **Supplementary Supplementary Figure *180***.

On a technical note, dehydrogenation is often found to be a stepwise process with a zwitterionic intermediate in a shallow minimum; however, this disappears upon adding higher-level and thermodynamic corrections. This is a concerted but highly asynchronous process, and we present only the key transition state for this transformation.

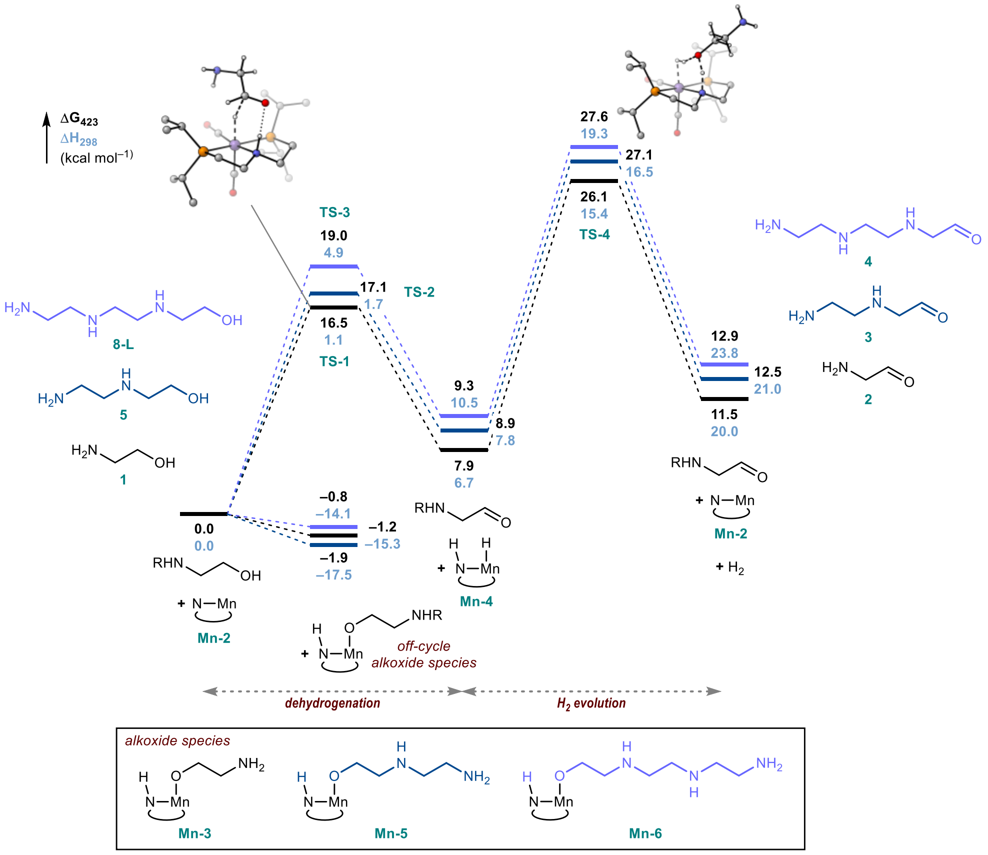

Supplementary Figure 180 Computed barriers for the oxidation of alcohol oligomers and hydrogen evolution including alkoxide off-cycle species.

We find that the production of the monomer aldehyde **2** is favoured over dimer or trimer dehydrogenation based on the driving forces and kinetics of the oligomer reduction (**Supplementary Supplementary Figure *180***). Additionally, with a greater concentration of **1** at the start of the reaction this is likely to be favoured; however, there are likely to be a variety of alcohols oxidised to aldehydes throughout the course of the reaction. Similar to previous work,[S4–S7,] we find that H_2 evolution can be

assisted by an outer sphere protic source with a barrier of 27.3 kcal mol^{-1} (relative to off-cycle alkoxide intermediate, **Supplementary Supplementary Figure *180***). The inclusion of a monomer alcohol unit reduces the barrier by 4.9 kcal mol^{-1} (**Supplementary Supplementary Figure *181***).

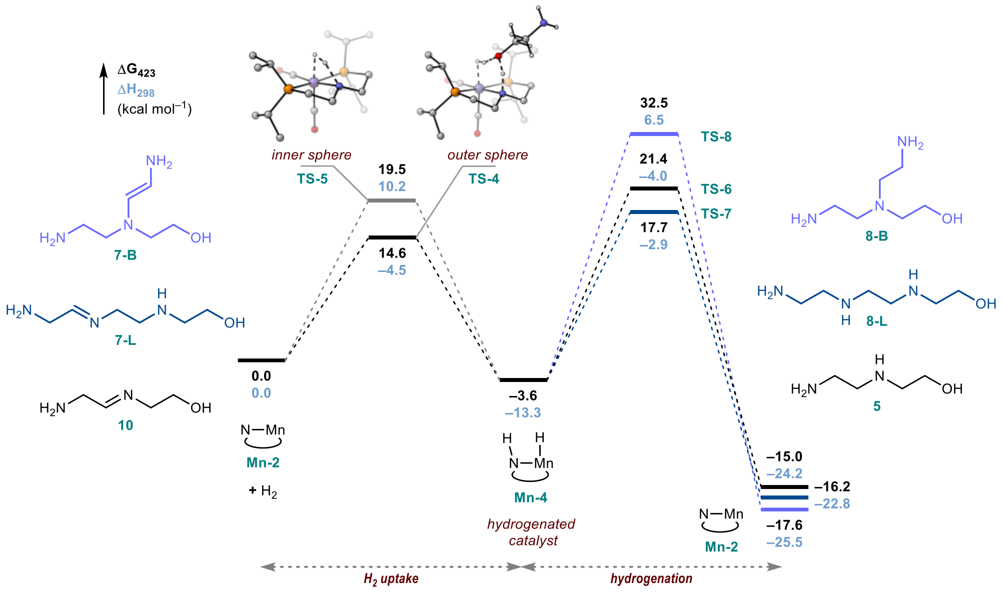

Supplementary Figure 181 Barriers for hydrogenation of the catalyst, and of imine and enamine substrates.

From the hydrogenated catalyst, imines or enamines formed in the reaction must be reduced and we calculate higher barriers for the enamine hydrogenation ($\Delta^{\ddagger}G$ = 36.1 kcal mol^{-1}, relative to the hydrogenated catalyst) than for imine hydrogenation ($\Delta^{\ddagger}G$ = 21.3 and 26.0 kcal mol^{-1}, **Supplementary Supplementary Figure *181***).

We also have considered the true transfer hydrogenation pathway, where H_2 remains on-metal, rather than reversible dihydrogen loss, for the dimer formation (**Supplementary Supplementary Figure *182***). We find that imine hydrogenation remains higher than reversible dihydrogen uptake/release by the catalyst ($\Delta^{\ddagger}G$ = 27.3 kcal mol^{-1}, **Supplementary Supplementary Figure *180***, compared to $\Delta^{\ddagger}G$ = 33.8 kcal mol^{-1}, **Supplementary Supplementary Figure *182***).

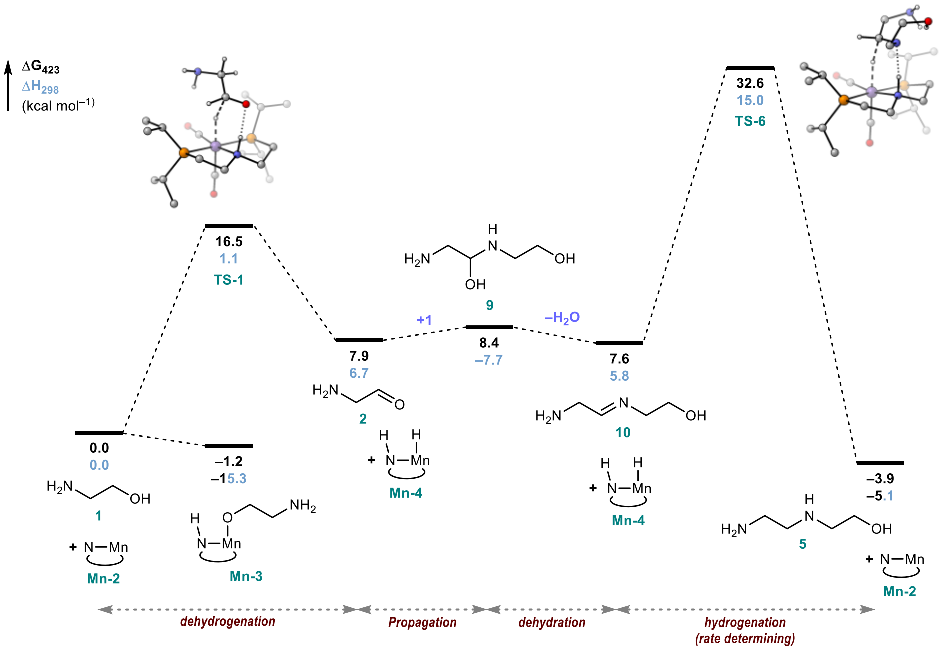

Supplementary Figure 182 Hydrogen borrowing pathway for the formation of dimer **5** from **1**.

2.4 Computational Raw Data

1
Frequencies, energies and thermodynamic properties:
Lowest Vibrational Mode (1/cm) = 195.5787
2nd Lowest Vibrational Mode (1/cm) = 295.8606
E(RB-P86) (a.u.) = -210.236793102
Thermal correction to Enthalpy (a.u.) = 0.101235
Thermal correction to Gibbs Free Energy (a.u.) = 0.068353
Total Entropy (cal/Kmol) = 69.207
E(RPBE1PBE) (a.u.) = -210.242890948
Optimised cartesian coordinates (Angstrom):
O -1.339469 -0.695588 -0.159419
C -0.828947 0.564312 0.236193
C 0.629419 0.690252 -0.240285
N 1.316905 -0.562312 0.114813
H -0.515660 -1.252313 -0.117921
H -0.864095 0.697047 1.351342
H -1.453438 1.374759 -0.201304
H 1.100003 1.616923 0.168243
H 0.638395 0.776343 -1.348064
H 2.162362 -0.697636 -0.452672
H 1.627023 -0.541614 1.096589

10
Frequencies, energies and thermodynamic properties:
Lowest Vibrational Mode (1/cm) = 42.0074
2nd Lowest Vibrational Mode (1/cm) = 72.2459
E(RB-P86) (a.u.) = -342.890605332
Thermal correction to Enthalpy (a.u.) = 0.152607
Thermal correction to Gibbs Free Energy (a.u.) = 0.108600
Total Entropy (cal/Kmol) = 92.620
E(RPBE1PBE) (a.u.) = -342.891712822
Optimised cartesian coordinates (Angstrom):
N 2.679977 -0.649446 -0.711494
C 2.290724 -0.009184 0.540386
C 0.884189 0.547661 0.622821
N 0.070355 0.484515 -0.364247
C -1.271749 1.019958 -0.197529
C -2.309042 -0.092236 -0.419366
O -2.204648 -1.130174 0.544949
H 2.498850 0.020134 -1.475353
H 2.004739 -1.405712 -0.902227
H 2.995202 0.824586 0.773963
H 2.423689 -0.722479 1.388010
H 0.601592 1.032216 1.594967
H -1.439656 1.809406 -0.965395
H -1.429426 1.480752 0.810784
H -2.204219 -0.481083 -1.462416
H -3.329233 0.336187 -0.324755
H -1.301408 -1.495290 0.455139

11
Frequencies, energies and thermodynamic properties:
Lowest Vibrational Mode (1/cm) = 41.8562
2nd Lowest Vibrational Mode (1/cm) = 78.5863
E(RB-P86) (a.u.) = -418.076085980
Thermal correction to Enthalpy (a.u.) = 0.158027
Thermal correction to Gibbs Free Energy (a.u.) = 0.112950
Total Entropy (cal/Kmol) = 94.873
E(RPBE1PBE) (a.u.) = -418.086942665
Optimised cartesian coordinates (Angstrom):
N 3.189884 -0.233715 -0.155493
C 1.915680 -0.825764 0.273808
C 0.847209 0.290900 0.332440
O 1.036162 1.141913 -0.796460
N -0.470176 -0.287881 0.391659
C -1.561177 0.654087 0.537904
C -2.871800 0.103673 0.032918
O -2.975084 -0.932813 -0.604828
H 3.701169 0.160942 0.646244
H 3.807405 -0.940586 -0.572650
H 1.947622 -1.371938 1.244620
H 1.597771 -1.555031 -0.503096
H 0.985307 0.882581 1.276513
H 2.007130 1.001232 -0.983463
H -0.650554 -0.870076 -0.440771
H -1.422827 1.614198 -0.031692
H -1.706619 0.973405 1.598482
H -3.772469 0.746274 0.260524

12
Frequencies, energies and thermodynamic properties:
Lowest Vibrational Mode (1/cm) = 33.3984
2nd Lowest Vibrational Mode (1/cm) = 83.1132
E(RB-P86) (a.u.) = -341.681912869
Thermal correction to Enthalpy (a.u.) = 0.128375
Thermal correction to Gibbs Free Energy (a.u.) = 0.085225
Total Entropy (cal/Kmol) = 90.817
E(RPBE1PBE) (a.u.) = -341.678157243
Optimised cartesian coordinates (Angstrom):
N -2.588321 -0.840221 -0.500798
C -2.233412 0.153293 0.505888

C -0.827710 0.712790 0.451989
N 0.010711 0.342951 -0.441604
C 1.342458 0.896921 -0.404943
C 2.407577 -0.116586 -0.010613
O 2.192639 -1.239990 0.403004
H -1.892314 -1.600167 -0.449651
H -2.410971 -0.425131 -1.428444
H -2.385703 -0.269370 1.527483
H -2.942538 1.013770 0.458800
H -0.567948 1.470916 1.237998
H 1.472979 1.763594 0.300568
H 1.633364 1.285183 -1.410090
H 3.461814 0.283504 -0.117807

13
Frequencies, energies and thermodynamic properties:
Lowest Vibrational Mode (1/cm) = 25.1737
2nd Lowest Vibrational Mode (1/cm) = 51.2503
E(RB-P86) (a.u.) = -553.155111861
Thermal correction to Enthalpy (a.u.) = 0.257701
Thermal correction to Gibbs Free Energy (a.u.) = 0.202655
Total Entropy (cal/Kmol) = 115.854
E(RPBE1PBE) (a.u.) = -553.165562029
Optimised cartesian coordinates (Angstrom):
N 3.495357 -0.122776 -0.964085
C 3.142392 0.308879 0.396002
C 2.015929 -0.577196 0.942096
N 0.864943 -0.500717 0.035473
C -0.180183 -1.501137 0.256033
C -1.537222 -1.072810 -0.341559
O -2.318351 -2.277938 -0.366276
N -2.230409 -0.038958 0.385857
C -2.384585 1.327299 -0.103073
C -1.240974 2.307601 0.223378
O -0.040803 2.042287 -0.462925
H 4.102777 -0.953715 -0.923170
H 4.048499 0.603205 -1.437198
H 2.761697 1.351479 0.337648
H 3.991229 0.309611 1.123111
H 1.718596 -0.234697 1.957674
H 2.390137 -1.628227 1.054916
H 1.263911 -0.597077 -0.915439
H -0.326528 -1.643492 1.349982
H 0.090768 -2.502743 -0.156201
H -1.374476 -0.688459 -1.380651
H -3.242621 -1.958451 -0.400875
H -2.089348 -0.094371 1.401168
H -3.332029 1.749675 0.309298
H -2.512607 1.285900 -1.207630
H -1.106820 2.322253 1.340272
H -1.576450 3.334345 -0.055629
H 0.265114 1.111325 -0.201646

14
Frequencies, energies and thermodynamic properties:
Lowest Vibrational Mode (1/cm) = 66.5603
2nd Lowest Vibrational Mode (1/cm) = 84.1044
E(RB-P86) (a.u.) = -476.771240975
Thermal correction to Enthalpy (a.u.) = 0.228446
Thermal correction to Gibbs Free Energy (a.u.) = 0.179969
Total Entropy (cal/Kmol) = 102.029
E(RPBE1PBE) (a.u.) = -476.765319997
Optimised cartesian coordinates (Angstrom):
N -0.165319 1.754218 -0.440364
C -1.261859 1.523805 0.512639
C -2.472362 0.743932 -0.053338
N -2.224579 -0.599784 -0.586934
C -1.645130 -1.566056 0.333839
C -0.131185 -1.616370 0.447890
N 0.644407 -1.069156 -0.410730
C 2.084587 -1.141994 -0.212176
C 2.734024 0.259217 -0.281780
O 2.268728 1.154623 0.701336
H -0.433644 2.453758 -1.146812
H 0.007829 0.858293 -0.937962
H -1.611701 2.501525 0.909533
H -0.842471 0.973563 1.384949
H -2.938788 1.342078 -0.868500
H -3.241118 0.663575 0.749979
H -1.652480 -0.555899 -1.440871
H -1.966419 -2.602673 0.056812
H -2.070719 -1.411128 1.350697
H 0.272928 -2.206519 1.310779
H 2.524254 -1.758326 -1.030859
H 2.346305 -1.631598 0.759275
H 2.587129 0.659977 -1.317181
H 3.832203 0.121944 -0.150179
H 1.406855 1.542285 0.343415

2
Frequencies, energies and thermodynamic properties:
Lowest Vibrational Mode (1/cm) = 120.7696
2nd Lowest Vibrational Mode (1/cm) = 251.0502
E(RB-P86) (a.u.) = -209.028423858
Thermal correction to Enthalpy (a.u.) = 0.076927

Thermal correction to Gibbs Free Energy (a.u.) = 0.043688
Total Entropy (cal/Kmol) = 69.958
E(RPBE1PBE) (a.u.) = -209.031694710
Optimised cartesian coordinates (Angstrom):
O 1.401952 -0.658325 -0.000015
C 0.886647 0.447186 0.000127
C -0.615031 0.686615 -0.000097
N -1.465577 -0.492732 -0.000103
H 1.518517 1.389291 0.000205
H -0.837375 1.338166 -0.879523
H -0.837754 1.338414 0.879024
H -1.214115 -1.076979 -0.811423
H -1.215542 -1.075974 0.812376

3
Frequencies, energies and thermodynamic properties:
Lowest Vibrational Mode (1/cm) = 46.3716
2nd Lowest Vibrational Mode (1/cm) = 76.8071
E(RB-P86) (a.u.) = -342.897516442
Thermal correction to Enthalpy (a.u.) = 0.152389
Thermal correction to Gibbs Free Energy (a.u.) = 0.109017
Total Entropy (cal/Kmol) = 91.285
E(RPBE1PBE) (a.u.) = -342.896485479
Optimised cartesian coordinates (Angstrom):
N -2.653871 -0.909548 0.573397
C -2.305125 -0.007473 -0.529527
C -1.077242 0.889167 -0.285588
N 0.102824 0.072078 -0.013904
C 1.282964 0.797536 0.390432
C 2.551184 -0.002168 0.205525
O 2.604995 -1.060677 -0.400096
H -2.940649 -0.337894 1.383109
H -1.772512 -1.352137 0.881071
H -3.180046 0.634131 -0.771908
H -2.116275 -0.620521 -1.442482
H -1.267872 1.530598 0.605868
H -0.940205 1.592701 -1.151674
H 0.341400 -0.516710 -0.825463
H 1.231329 1.126682 1.456859
H 1.474031 1.754885 -0.180006
H 3.477493 0.473600 0.643896

4
Frequencies, energies and thermodynamic properties:
Lowest Vibrational Mode (1/cm) = 26.4869
2nd Lowest Vibrational Mode (1/cm) = 48.6585
E(RB-P86) (a.u.) = -476.768966696
Thermal correction to Enthalpy (a.u.) = 0.228142
Thermal correction to Gibbs Free Energy (a.u.) = 0.174857
Total Entropy (cal/Kmol) = 112.149
E(RPBE1PBE) (a.u.) = -476.763367771
Optimised cartesian coordinates (Angstrom):
N 3.846791 -0.751478 1.144695
C 3.381277 -1.077881 -0.209027
C 2.526978 0.010182 -0.885540
N 1.350079 0.309923 -0.074891
C 0.550167 1.437094 -0.539678
C -0.767880 1.509596 0.235398
N -1.547664 0.291256 0.024336
C -2.735365 0.165168 0.835662
C -3.719973 -0.832743 0.272401
O -3.624358 -1.327117 -0.839563
H 4.505397 0.040186 1.076629
H 3.031158 -0.350474 1.637321
H 2.779672 -2.016432 -0.159860
H 4.256136 -1.312484 -0.853828
H 2.277658 -0.299598 -1.937772
H 3.132828 0.942237 -0.976448
H 0.722643 -0.509194 -0.042235
H 0.317057 1.385447 -1.640439
H 1.115151 2.385452 -0.394001
H -1.318674 2.444704 -0.048130
H -0.538671 1.590122 1.321316
H -1.813718 0.187867 -0.966116
H -3.337037 1.115097 0.950085
H -2.498298 -0.132241 1.885781
H -4.592105 -1.070151 0.949933

5
Frequencies, energies and thermodynamic properties:
Lowest Vibrational Mode (1/cm) = 64.9783
2nd Lowest Vibrational Mode (1/cm) = 78.7652
E(RB-P86) (a.u.) = -344.107981286
Thermal correction to Enthalpy (a.u.) = 0.176962
Thermal correction to Gibbs Free Energy (a.u.) = 0.134089
Total Entropy (cal/Kmol) = 90.233
E(RPBE1PBE) (a.u.) = -344.109674911
Optimised cartesian coordinates (Angstrom):
N -2.790139 0.330332 -0.565530
C -2.139500 -0.682860 0.277480
C -0.798910 -0.147345 0.796892
N 0.033882 0.261924 -0.334329
C 1.220725 1.056364 -0.011137
C 2.368742 0.094107 0.358473
O 2.397866 -0.963915 -0.582306

H -3.221555 1.053951 0.027609
H -3.564470 -0.091054 -1.094736
H -1.942407 -1.576885 -0.353040
H -2.750342 -1.029849 1.147240
H -0.291224 -0.937445 1.393065
H -0.990289 0.705127 1.503391
H -0.592332 0.751893 -0.991458
H 1.050151 1.801613 0.807255
H 1.506401 1.621424 -0.924108
H 2.222113 -0.283644 1.405122
H 3.341936 0.634288 0.350008
H 1.436540 -1.005485 -0.843136

6-B
Frequencies, energies and thermodynamic properties:
Lowest Vibrational Mode (1/cm) = 61.1337
2nd Lowest Vibrational Mode (1/cm) = 80.6611
E(RB-P86) (a.u.) = -553.162973736
Thermal correction to Enthalpy (a.u.) = 0.258285
Thermal correction to Gibbs Free Energy (a.u.) = 0.206933
Total Entropy (cal/Kmol) = 108.079
E(RPBE1PBE) (a.u.) = -553.170891004
Optimised cartesian coordinates (Angstrom):
O 0.030114 -0.202366 1.655726
C 0.685533 -0.735263 0.481607
N 0.112506 -0.304960 -0.778745
C -1.105076 -0.997799 -1.211827
C -2.386476 -0.817000 -0.364075
O -2.288874 -1.349039 0.940847
C 0.311883 1.065783 -1.247696
C -0.457827 2.215094 -0.553165
N -0.060429 2.381335 0.858227
C 2.210484 -0.490808 0.567720
N 3.009991 -1.121796 -0.485317
H -0.002495 0.811730 1.507880
H 0.516766 -1.834783 0.533307
H -0.884983 -2.090734 -1.240634
H -1.323792 -0.686567 -2.258570
H -3.209915 -1.357015 -0.886284
H -2.683543 0.264462 -0.359498
H -1.513808 -0.882332 1.373240
H 0.042480 1.096046 -2.326918
H 1.398051 1.307884 -1.207452
H -1.546870 2.004736 -0.579845
H -0.298885 3.142200 -1.154367
H 0.864943 2.831503 0.919596
H -0.710081 3.013844 1.345343
H 2.542917 -0.863392 1.559005
H 2.412639 0.604211 0.580417
H 2.546614 -0.927255 -1.387290
H 2.934444 -2.145401 -0.385059

6-L
Frequencies, energies and thermodynamic properties:
Lowest Vibrational Mode (1/cm) = 14.3200
2nd Lowest Vibrational Mode (1/cm) = 32.7206
E(RB-P86) (a.u.) = -553.149629942
Thermal correction to Enthalpy (a.u.) = 0.258049
Thermal correction to Gibbs Free Energy (a.u.) = 0.200796
Total Entropy (cal/Kmol) = 120.499
E(RPBE1PBE) (a.u.) = -553.162710869
Optimised cartesian coordinates (Angstrom):
N -1.765828 2.310236 0.024344
C -2.836238 1.314163 -0.027314
C -2.287662 -0.092663 0.239019
O -3.423965 -0.970827 0.256685
N -1.284238 -0.426490 -0.756943
C -0.503408 -1.651469 -0.533377
C 0.627471 -1.433287 0.480811
N 1.504145 -0.353433 0.034184
C 2.528091 0.072660 0.991512
C 3.567902 0.903625 0.223655
O 3.951463 0.205043 -0.948730
H -1.124262 2.129994 -0.761354
H -2.160423 3.244998 -0.148116
H -3.403446 1.268804 -0.995882
H -3.586956 1.536380 0.760068
H -1.782781 -0.094146 1.233326
H -3.156036 -1.794925 0.705178
H -1.727739 -0.451449 -1.684871
H -0.053452 -1.941747 -1.505747
H -1.128376 -2.518844 -0.201824
H 1.212633 -2.374785 0.577028
H 0.191395 -1.236376 1.495836
H 0.894918 0.440477 -0.221356
H 3.020052 -0.842026 1.390902
H 2.126715 0.639669 1.869285
H 4.464671 1.086675 0.855964
H 3.131036 1.910124 -0.015438
H 3.106582 -0.266916 -1.173572

7-B
Frequencies, energies and thermodynamic properties:
Lowest Vibrational Mode (1/cm) = 39.4778
2nd Lowest Vibrational Mode (1/cm) = 72.2566

E(RB-P86) (a.u.) = -476.765925282
Thermal correction to Enthalpy (a.u.) = 0.228732
Thermal correction to Gibbs Free Energy (a.u.) = 0.177701
Total Entropy (cal/Kmol) = 107.405
E(RPBE1PBE) (a.u.) = -476.758926385
Optimised cartesian coordinates (Angstrom):
N -3.695296 0.252948 -0.754676
C -2.480030 -0.149206 -0.134701
C -1.428787 0.683806 0.123876
N -0.176597 0.356252 0.657387
C 0.805863 1.421403 0.825291
C 1.674270 1.744959 -0.408170
O 2.674752 0.796040 -0.685955
C 0.078420 -0.997075 1.107990
C 0.405486 -2.025004 0.002268
N 1.734516 -1.795806 -0.587752
H -4.516176 -0.146365 -0.280788
H -3.809624 1.274828 -0.754737
H -2.412843 -1.234966 0.042597
H -1.541188 1.759969 -0.101453
H 1.484294 1.146253 1.663440
H 0.275098 2.353879 1.133126
H 2.178810 2.722060 -0.219221
H 0.981991 1.917192 -1.277028
H 2.244382 -0.116999 -0.740310
H -0.816763 -1.370380 1.664450
H 0.915134 -0.973174 1.840931
H -0.349917 -1.934628 -0.805506
H 0.292688 -3.050103 0.431790
H 1.839248 -2.333288 -1.459093
H 2.467149 -2.149662 0.045404

7-L
Frequencies, energies and thermodynamic properties:
Lowest Vibrational Mode (1/cm) = 30.0570
2nd Lowest Vibrational Mode (1/cm) = 35.2539
E(RB-P86) (a.u.) = -476.763523109
Thermal correction to Enthalpy (a.u.) = 0.228459
Thermal correction to Gibbs Free Energy (a.u.) = 0.174826
Total Entropy (cal/Kmol) = 112.880
E(RPBE1PBE) (a.u.) = -476.759352130
Optimised cartesian coordinates (Angstrom):
N 3.663719 -1.025232 -0.971844
C 3.162516 -1.077512 0.397452
C 2.091425 -0.075055 0.777620
N 1.643754 0.788562 -0.054501
C 0.607412 1.711719 0.379826
C -0.641316 1.587475 -0.522095
N -1.320034 0.291831 -0.499730
C -1.979606 -0.094156 0.754437
C -3.023365 -1.172991 0.417921
O -3.799952 -0.748837 -0.687442
H 3.988347 -0.062068 -1.149402
H 2.855190 -1.116328 -1.606213
H 4.008701 -0.953988 1.114648
H 2.766564 -2.097440 0.617164
H 1.721851 -0.127146 1.836745
H 0.996213 2.750178 0.273979
H 0.328877 1.571128 1.455751
H -0.336849 1.799426 -1.570255
H -1.372161 2.373049 -0.230655
H -0.663350 -0.444900 -0.796595
H -2.502197 0.806649 1.145414
H -1.291179 -0.450837 1.559628
H -3.688285 -1.352493 1.291521
H -2.496345 -2.142910 0.210439
H -3.130233 -0.224639 -1.201080

8-B
Frequencies, energies and thermodynamic properties:
Lowest Vibrational Mode (1/cm) = 38.9035
2nd Lowest Vibrational Mode (1/cm) = 52.1029
E(RB-P86) (a.u.) = -477.977821084
Thermal correction to Enthalpy (a.u.) = 0.252049
Thermal correction to Gibbs Free Energy (a.u.) = 0.200713
Total Entropy (cal/Kmol) = 108.047
E(RPBE1PBE) (a.u.) = -477.977909706
Optimised cartesian coordinates (Angstrom):
N 2.109191 0.284478 1.509628
C 2.540154 -0.267026 0.222458
C 1.701829 0.349103 -0.907872
N 0.269243 0.096640 -0.748019
C -0.624994 1.206357 -1.096907
C -1.122495 1.955622 0.149328
O -1.957757 1.173442 0.977159
C -0.172700 -1.237704 -1.128057
C -1.416513 -1.724991 -0.365226
N -1.271172 -1.527872 1.086341
H 2.529427 -0.249016 2.282771
H 1.090582 0.126877 1.575076
H 2.481580 -1.385944 0.153847
H 3.608958 -0.012494 0.040962
H 2.091649 -0.006270 -1.899087
H 1.855550 1.448167 -0.878470
H -1.508020 0.816770 -1.652804

H -0.119123 1.925691 -1.786258
H -1.704401 2.851207 -0.172411
H -0.222159 2.340498 0.698544
H -1.526119 0.275565 1.123075
H 0.661712 -1.941454 -0.913177
H -0.379145 -1.335764 -2.229350
H -1.599782 -2.786403 -0.661318
H -2.311227 -1.149204 -0.688038
H -0.416234 -1.991641 1.427375
H -2.053719 -1.975010 1.584001

8-L
Frequencies, energies and thermodynamic properties:
Lowest Vibrational Mode (1/cm) = 45.6063
2nd Lowest Vibrational Mode (1/cm) = 72.6278
E(RB-P86) (a.u.) = -477.982280040
Thermal correction to Enthalpy (a.u.) = 0.252675
Thermal correction to Gibbs Free Energy (a.u.) = 0.202464
Total Entropy (cal/Kmol) = 105.679
E(RPBE1PBE) (a.u.) = -477.980908871
Optimised cartesian coordinates (Angstrom):
C 2.051406 1.114133 0.644447
C 2.505867 -0.144393 -0.123341
H 1.418949 0.815941 1.518314
H 2.955189 1.611131 1.074152
H 3.159440 -0.769222 0.527488
H 3.142397 0.197661 -0.972526
O 1.385328 2.036242 -0.199312
H 0.389426 1.911877 -0.136758
N 1.420302 -0.976304 -0.650600
H 0.697131 -0.353888 -1.046599
C 0.755047 -1.818436 0.340540
H 0.665664 -1.334313 1.350146
H 1.343599 -2.749490 0.514109
C -0.647834 -2.215184 -0.144060
H -0.547958 -2.808787 -1.078028
H -1.115790 -2.884825 0.623734
N -1.463998 -1.036796 -0.460612
H -2.266831 -1.316386 -1.038306
C -1.923842 -0.264023 0.693961
H -1.060772 -0.123241 1.378952
H -2.719174 -0.781121 1.291559
C -2.425589 1.119759 0.269891
H -3.379442 1.009916 -0.304351
H -2.673252 1.706734 1.179669
N -1.387242 1.831215 -0.494850
H -1.249511 1.326853 -1.383401
H -1.715451 2.773289 -0.749856

9
Frequencies, energies and thermodynamic properties:
Lowest Vibrational Mode (1/cm) = 68.3392
2nd Lowest Vibrational Mode (1/cm) = 104.8583
E(RB-P86) (a.u.) = -419.290344540
Thermal correction to Enthalpy (a.u.) = 0.182497
Thermal correction to Gibbs Free Energy (a.u.) = 0.138769
Total Entropy (cal/Kmol) = 92.034
E(RPBE1PBE) (a.u.) = -419.301819864
Optimised cartesian coordinates (Angstrom):
N -2.977899 -0.525751 0.099457
C -2.110705 0.654666 -0.032574
C -0.653213 0.227993 0.253216
O -0.475438 -1.054048 -0.395956
N 0.278294 1.221317 -0.182048
C 1.674497 1.105893 0.253942
C 2.481583 -0.108468 -0.252954
O 2.085336 -1.344974 0.307917
H -3.842093 -0.419628 -0.445469
H -3.265890 -0.665027 1.077985
H -2.382711 1.516642 0.617999
H -2.162569 1.006651 -1.085611
H -0.508129 0.100513 1.355599
H -1.408475 -1.424043 -0.365279
H 0.215757 1.346967 -1.201911
H 1.686741 1.088228 1.367469
H 2.203277 2.035483 -0.055995
H 3.549226 0.052163 0.022275
H 2.444189 -0.117511 -1.375185
H 1.135761 -1.477736 0.040797

H2
Frequencies, energies and thermodynamic properties:
Lowest Vibrational Mode (1/cm) = 4273.6262
2nd Lowest Vibrational Mode (1/cm) =
E(RB-P86) (a.u.) = -1.17253810941
Thermal correction to Enthalpy (a.u.) = 0.013041
Thermal correction to Gibbs Free Energy (a.u.) = -0.001813
Total Entropy (cal/Kmol) = 31.262
E(RPBE1PBE) (a.u.) = -1.16812439147
Optimised cartesian coordinates (Angstrom):
H 0.000000 0.000000 0.383762
H 0.000000 0.000000 -0.383762

H2O
Frequencies, energies and thermodynamic properties:

Lowest Vibrational Mode (1/cm) = 1608.3449
2nd Lowest Vibrational Mode (1/cm) = 3675.6577
E(RB-P86) (a.u.) = -76.3650012389
Thermal correction to Enthalpy (a.u.) = 0.024388
Thermal correction to Gibbs Free Energy (a.u.) = 0.002912
Total Entropy (cal/Kmol) = 45.200
E(RPBE1PBE) (a.u.) = -76.3831415862
Optimised cartesian coordinates (Angstrom):
O 0.000000 0.000000 0.123211
H 0.000000 0.757670 -0.492844
H 0.000000 -0.757670 -0.492844

Mn-2
Frequencies, energies and thermodynamic properties:
Lowest Vibrational Mode (1/cm) = 26.1911
2nd Lowest Vibrational Mode (1/cm) = 33.6855
E(RB-P86) (a.u.) = -2745.85675854
Thermal correction to Enthalpy (a.u.) = 0.534071
Thermal correction to Gibbs Free Energy (a.u.) = 0.438785
Total Entropy (cal/Kmol) = 200.546
E(RPBE1PBE) (a.u.) = -2745.47148486
Optimised cartesian coordinates (Angstrom):
P 2.254297 -0.122813 -0.230038
C 2.468120 -1.210391 -1.729666
C 1.194338 -2.046528 -1.888736
H 3.381155 -1.840820 -1.686339
H 2.577004 -0.519139 -2.592545
H 1.045272 -2.291178 -2.969661
H 1.313539 -3.038405 -1.385008
N -0.000475 -1.358746 -1.364265
C -1.195394 -2.042851 -1.893198
C -2.468602 -1.206003 -1.732758
H -1.316578 -3.036672 -1.393752
H -1.044762 -2.283349 -2.974849
H -2.577061 -0.512914 -2.594196
H -3.382167 -1.835765 -1.690739
P -2.254333 -0.121982 -0.230656
Mn -0.000125 0.129481 -0.180706
C -0.000221 0.734267 1.481511
O -0.000159 1.126506 2.602958
C -0.000155 1.739172 -0.855422
O -0.000711 2.789161 -1.410284
C 3.378067 1.346157 -0.628010
C 4.861146 0.973879 -0.794044
C 3.184884 2.550365 0.310274
H 2.975900 1.639745 -1.624964
H 5.012676 0.131852 -1.500334
H 5.428486 1.843701 -1.190043
H 5.326999 0.696406 0.174834
H 2.116038 2.812961 0.431414
H 3.609060 2.367310 1.318207
H 3.706220 3.437670 -0.108806
C 3.128682 -1.098551 1.140539
C 2.402399 -2.433285 1.385120
C 3.282883 -0.306996 2.450555
H 4.142123 -1.318515 0.734623
H 2.404702 -3.088109 0.490872
H 2.901572 -2.992567 2.205446
H 1.345789 -2.264837 1.681453
H 3.920924 0.590826 2.331516
H 2.302544 0.021788 2.850844
H 3.762434 -0.950040 3.219956
C -3.377894 1.348229 -0.624479
C -3.183574 2.550109 0.316578
C -4.861208 0.976909 -0.790403
H -2.976275 1.644097 -1.620972
H -2.114592 2.812572 0.436779
H -3.705628 3.438375 -0.099586
H -3.606270 2.364502 1.324664
H -5.013540 0.136470 -1.498422
H -5.326569 0.697498 0.178151
H -5.428465 1.847811 -1.184149
C -3.128347 -1.101083 1.137837
C -3.281432 -0.312851 2.449953
C -2.402343 -2.436714 1.378364
H -4.142110 -1.319541 0.731897
H -3.918811 0.585783 2.333508
H -3.761137 -0.957498 3.217894
H -2.300675 0.014228 2.850632
H -2.407024 -3.089765 0.482848
H -1.344934 -2.269405 1.672529
H -2.899984 -2.997289 2.198740

Mn-3
Frequencies, energies and thermodynamic properties:
Lowest Vibrational Mode (1/cm) = 28.7966
2nd Lowest Vibrational Mode (1/cm) = 33.4971
E(RB-P86) (a.u.) = -2956.11510448
Thermal correction to Enthalpy (a.u.) = 0.638347
Thermal correction to Gibbs Free Energy (a.u.) = 0.531751
Total Entropy (cal/Kmol) = 224.350
E(RPBE1PBE) (a.u.) = -2955.74186801
Optimised cartesian coordinates (Angstrom):
P -2.296957 -0.238483 0.169649
C -2.461277 -0.299843 2.040675

C -1.257949 -1.036084 2.629731
H -3.418053 -0.745641 2.380162
H -2.448695 0.757471 2.381997
H -1.235691 -0.915760 3.739043
H -1.311645 -2.126006 2.423016
N -0.013723 -0.511928 2.026154
C 1.215457 -1.073124 2.627104
C 2.438883 -0.370799 2.038149
H 1.237376 -2.163603 2.417613
H 1.198109 -0.954816 3.736711
H 2.456907 0.685910 2.381101
H 3.382713 -0.844372 2.376222
P 2.274945 -0.301841 0.167434
Mn -0.012593 -0.329145 -0.114111
C -0.036659 -2.048319 -0.445693
O -0.054295 -3.213678 -0.691515
C -0.007056 0.043533 -1.831656
O -0.002436 0.283853 -2.992401
C -3.216888 1.367213 -0.225085
C -4.594778 1.502285 0.445270
C -3.301818 1.656377 -1.733520
H -2.520319 2.104015 0.231830
H -4.554731 1.382409 1.546525
H -5.013177 2.511766 0.240720
H -5.324810 0.765465 0.046775
H -2.327289 1.525980 -2.244140
H -4.044915 1.000635 -2.234999
H -3.633181 2.704368 -1.897846
C -3.445570 -1.601520 -0.478929
C -3.404377 -2.891078 0.360274
C -3.159166 -1.907139 -1.961765
H -4.468924 -1.169985 -0.396394
H -3.699310 -2.726836 1.416221
H -4.116501 -3.631517 -0.064288
H -2.398050 -3.356383 0.342323
H -3.165599 -1.000324 -2.597278
H -2.171983 -2.396438 -2.081800
H -3.930735 -2.601602 -2.359047
C 3.245496 1.274878 -0.226023
C 3.341724 1.560924 -1.734523
C 4.626638 1.366489 0.445283
H 2.574198 2.034721 0.230817
H 2.365580 1.456128 -2.248090
H 3.701528 2.599490 -1.898749
H 4.068375 0.885191 -2.233520
H 4.582020 1.249488 1.546664
H 5.333014 0.606246 0.048227
H 5.077185 2.361836 0.239721
C 3.382066 -1.697914 -0.483722
C 3.086265 -1.993304 -1.966708
C 3.303425 -2.986723 0.354011
H 4.417780 -1.297019 -0.401156
H 3.121460 -1.086800 -2.601677
H 3.835571 -2.711523 -2.364351
H 2.084169 -2.451084 -2.087139
H 3.602980 -2.832266 1.410116
H 2.284152 -3.422952 0.335542
H 3.994066 -3.746748 -0.071427
C 0.041352 2.780151 -0.122346
H 0.001730 0.531849 2.100684
H -0.872114 2.931433 -0.767816
O 0.009096 1.599672 0.605010
H 0.904533 2.834094 -0.845763
C 0.147288 4.013000 0.804743
H 1.063575 3.910280 1.430698
H -0.709276 3.996209 1.517109
N 0.177861 5.317041 0.129998
H 0.960972 5.325522 -0.541744
H -0.664193 5.406093 -0.459536

Mn-4
Frequencies, energies and thermodynamic properties:
Lowest Vibrational Mode (1/cm) = 31.9909
2nd Lowest Vibrational Mode (1/cm) = 37.9241
E(RB-P86) (a.u.) = -2747.05386562
Thermal correction to Enthalpy (a.u.) = 0.555150
Thermal correction to Gibbs Free Energy (a.u.) = 0.461619
Total Entropy (cal/Kmol) = 196.852
E(RPBE1PBE) (a.u.) = -2746.66880885
Optimised cartesian coordinates (Angstrom):
P 2.226138 0.065304 -0.311693
C 2.447407 -1.089129 -1.788246
C 1.242325 -2.027633 -1.850612
H 3.400692 -1.654776 -1.761880
H 2.474273 -0.453853 -2.700906
H 1.236281 -2.602934 -2.806818
H 1.264692 -2.762318 -1.018765
N 0.000062 -1.230229 -1.711034
C -1.242687 -2.026662 -1.851741
C -2.447165 -1.087381 -1.789374
H -1.265947 -2.761898 -1.020410
H -1.236576 -2.601326 -2.808331
H -2.472927 -0.451256 -2.701468
H -3.400869 -1.652370 -1.764117
P -2.226078 0.065708 -0.311797

Mn 0.000053 0.035640 0.050513
C -0.000006 -1.288432 1.237158
O -0.000149 -2.116812 2.092924
C 0.000248 1.290356 1.270100
O 0.000664 2.164773 2.074802
C 3.041257 1.645298 -0.981785
C 4.468646 1.461983 -1.523954
C 2.962457 2.830880 -0.005727
H 2.358979 1.866082 -1.834315
H 4.547942 0.642003 -2.266557
H 4.804704 2.394801 -2.026791
H 5.196438 1.256765 -0.710223
H 1.947808 2.955798 0.420165
H 3.676868 2.714389 0.836461
H 3.233186 3.772434 -0.530576
C 3.459702 -0.574476 0.983726
C 3.459170 -2.108380 1.105474
C 3.187403 0.073650 2.353710
H 4.463743 -0.252996 0.626062
H 3.741001 -2.616951 0.161290
H 4.198458 -2.420539 1.874822
H 2.468255 -2.488399 1.427237
H 3.178470 1.180542 2.312281
H 2.206223 -0.253593 2.754235
H 3.970831 -0.230526 3.081332
C -3.041162 1.646294 -0.980445
C -2.962778 2.830853 -0.003116
C -4.468422 1.463318 -1.523067
H -2.358731 1.868053 -1.832596
H -1.948198 2.955688 0.422958
H -3.233784 3.772868 -0.526998
H -3.677186 2.713258 0.838920
H -4.547457 0.644186 -2.266633
H -5.196296 1.257035 -0.709676
H -4.804568 2.396657 -2.024882
C -3.459839 -0.575281 0.982878
C -3.187713 0.071425 2.353569
C -3.459504 -2.109331 1.103027
H -4.463828 -0.253376 0.625446
H -3.178633 1.178359 2.313264
H -3.971337 -0.233388 3.080713
H -2.206681 -0.256376 2.753988
H -3.741489 -2.616879 0.158341
H -2.468654 -2.489848 1.424390
H -4.198790 -2.422183 1.872097
H -0.000396 1.240492 -1.031860
H 0.000679 -0.525982 -2.464713

Mn-5
Frequencies, energies and thermodynamic properties:
Lowest Vibrational Mode (1/cm) = 17.6156
2nd Lowest Vibrational Mode (1/cm) = 23.5098
E(RB-P86) (a.u.) = -3089.98839579
Thermal correction to Enthalpy (a.u.) = 0.714505
Thermal correction to Gibbs Free Energy (a.u.) = 0.599586
Total Entropy (cal/Kmol) = 241.867
E(RPBE1PBE) (a.u.) = -3089.61253649
Optimised cartesian coordinates (Angstrom):
P -1.141722 1.820829 -0.046583
C -1.719292 1.485119 -1.803726
C -0.525097 1.060661 -2.658256
H -2.257478 2.342822 -2.255406
H -2.443654 0.645188 -1.735108
H -0.872473 0.671321 -3.644726
H 0.145300 1.920930 -2.867409
N 0.259377 0.031109 -1.941358
C 1.367678 -0.530083 -2.745393
C 2.031892 -1.670719 -1.974364
H 2.089389 0.287031 -2.957090
H 0.990564 -0.895092 -3.730210
H 1.325163 -2.524810 -1.898411
H 2.939884 -2.038879 -2.493281
P 2.364588 -1.114946 -0.210101
Mn 0.721550 0.468039 0.108548
C 1.813987 1.791100 -0.239989
O 2.563866 2.689714 -0.460332
C 0.970731 0.670168 1.837024
O 1.147646 0.816797 2.999596
C -2.705105 1.411508 0.939288
C -3.990542 2.062651 0.399740
C -2.544016 1.670707 2.447397
H -2.776956 0.312061 0.782113
H -4.181949 1.825059 -0.665924
H -4.865801 1.693738 0.977403
H -3.977668 3.168459 0.507808
H -1.598734 1.255310 2.849966
H -2.570680 2.755872 2.682597
H -3.383805 1.198678 3.001337
C -1.008351 3.709907 0.080536
C -0.429136 4.375133 -1.180812
C -0.211399 4.127646 1.331424
H -2.059815 4.056827 0.199042
H -1.034151 4.177166 -2.088483
H -0.407779 5.477557 -1.039930
H 0.613261 4.048917 -1.371664

H -0.590823 3.659280 2.260318
H 0.858566 3.857366 1.229721
H -0.269547 5.229801 1.463139
C 2.259207 -2.759183 0.720217
C 2.575557 -2.623156 2.219655
C 3.059587 -3.910008 0.086898
H 1.173363 -2.972734 0.609156
H 2.051601 -1.766487 2.687983
H 2.263079 -3.545266 2.755390
H 3.664809 -2.499829 2.398373
H 2.791942 -4.091974 -0.973207
H 4.155185 -3.732020 0.137901
H 2.861186 -4.852885 0.641445
C 4.209120 -0.674809 -0.164317
C 4.556806 0.123504 1.107076
C 4.695713 0.067023 -1.422107
H 4.731573 -1.657449 -0.120465
H 4.212705 -0.372945 2.035076
H 5.658527 0.249710 1.182457
H 4.103832 1.134619 1.077192
H 4.550147 -0.519780 -2.351337
H 4.188425 1.046143 -1.538416
H 5.785092 0.269462 -1.333552
C -1.076117 -1.720688 1.340352
H -1.617718 -0.992348 2.002825
O -0.578829 -1.125642 0.183860
C -2.056478 -2.849332 0.983074
H -2.477500 -3.286868 1.918243
H -1.486578 -3.676507 0.474799
N -3.141287 -2.338452 0.148649
H -2.690480 -1.788278 -0.598993
H -0.372646 -0.741452 -1.633965
H -0.274806 -2.167313 1.990056
C -3.997503 -3.364806 -0.439403
H -4.398471 -3.998866 0.386011
C -5.176551 -2.729263 -1.197624
H -4.766923 -2.112809 -2.033121
H -5.775942 -3.531474 -1.681035
N -6.063714 -1.885381 -0.387092
H -5.448535 -1.288978 0.190281
H -6.539391 -2.487727 0.302745
H -3.441287 -4.066127 -1.121588

Mn-6
Frequencies, energies and thermodynamic properties:
Lowest Vibrational Mode (1/cm) = 14.5507
2nd Lowest Vibrational Mode (1/cm) = 15.6289
E(RB-P86) (a.u.) = -3223.85787458
Thermal correction to Enthalpy (a.u.) = 0.790244
Thermal correction to Gibbs Free Energy (a.u.) = 0.665389
Total Entropy (cal/Kmol) = 262.780
E(RPBE1PBE) (a.u.) = -3223.47834718
Optimised cartesian coordinates (Angstrom):
P 2.832751 -1.394299 0.168290
C 2.509739 -1.858883 1.960527
C 2.055181 -0.618216 2.729326
H 3.378433 -2.345139 2.448680
H 1.683899 -2.601930 1.939176
H 1.676681 -0.904479 3.739396
H 2.896897 0.088986 2.886529
N 1.002695 0.082320 1.960762
C 0.410571 1.229242 2.683922
C -0.749370 1.805439 1.872264
H 1.207182 1.987273 2.839041
H 0.058892 0.914914 3.695293
H -1.588334 1.076807 1.855591
H -1.137692 2.739573 2.326168
P -0.211214 2.022066 0.084027
Mn 1.424042 0.407780 -0.117739
C 2.714659 1.560442 0.148813
O 3.598281 2.341835 0.313076
C 1.618380 0.541710 -1.860213
O 1.761548 0.643457 -3.032122
C 2.439762 -3.024028 -0.709125
C 3.095464 -4.266877 -0.083647
C 2.693971 -2.965228 -2.225137
H 1.341677 -3.073182 -0.539053
H 2.860933 -4.384537 0.993307
H 2.731524 -5.181620 -0.600114
H 4.201015 -4.254135 -0.194108
H 2.274328 -2.050783 -2.689352
H 3.778918 -3.003703 -2.459687
H 2.225013 -3.842634 -2.720303
C 4.717427 -1.234175 0.023316
C 5.373168 -0.553392 1.238119
C 5.114229 -0.517271 -1.281786
H 5.085531 -2.284327 -0.022617
H 5.188804 -1.095792 2.187233
H 6.474641 -0.519514 1.092558
H 5.025536 0.493072 1.354972
H 4.649710 -0.968429 -2.180037
H 4.822720 0.551674 -1.251691
H 6.216754 -0.562417 -1.415234
C -1.862015 1.829133 -0.822032
C -1.736605 2.026144 -2.342885

C -3.010792 2.675616 -0.246587
H -2.086515 0.756245 -0.629781
H -0.885351 1.462354 -2.773593
H -2.663601 1.674094 -2.844514
H -1.610950 3.096925 -2.609936
H -3.180980 2.497684 0.834271
H -2.843102 3.764254 -0.392514
H -3.957933 2.423879 -0.771343
C 0.191593 3.867675 -0.094277
C 0.977224 4.140696 -1.391518
C 0.924986 4.461322 1.121822
H -0.802089 4.364227 -0.171026
H 0.487865 3.714457 -2.288758
H 1.072738 5.236745 -1.549606
H 2.000603 3.719197 -1.332925
H 0.343441 4.371555 2.061466
H 1.915996 3.987178 1.271940
H 1.101775 5.545681 0.952761
C -0.727039 -1.518900 -1.211933
H -0.023489 -2.080083 -1.885047
O -0.100223 -0.977128 -0.092671
C -1.833487 -2.497240 -0.787198
H -1.359093 -3.380173 -0.275069
H -2.344652 -2.896607 -1.693933
N -2.809879 -1.826002 0.068031
H -2.265485 -1.326705 0.788578
H 0.251497 -0.595353 1.701337
H -1.197031 -0.735929 -1.867058
C -3.780946 -2.711737 0.703989
H -3.312634 -3.455994 1.404992
C -4.843233 -1.894650 1.464599
H -5.542832 -2.586908 1.982811
H -4.330028 -1.314378 2.267655
N -5.620455 -0.962321 0.645658
H -4.944716 -0.433433 0.072134
H -4.273265 -3.317197 -0.090904
C -6.610634 -1.574655 -0.244772
H -6.170277 -2.189785 -1.073863
H -7.232763 -2.271567 0.363815
C -7.520732 -0.486695 -0.847638
H -6.895835 0.176946 -1.491205
H -8.258903 -0.961679 -1.530263
N -8.227212 0.345793 0.133821
H -8.905009 -0.251725 0.632725
H -7.526468 0.578724 0.857970

TS-1
Frequencies, energies and thermodynamic properties:
Lowest Vibrational Mode (1/cm) = -127.4399
2nd Lowest Vibrational Mode (1/cm) = 35.9426
E(RB-P86) (a.u.) = -2956.08351552
Thermal correction to Enthalpy (a.u.) = 0.632803
Thermal correction to Gibbs Free Energy (a.u.) = 0.527613
Total Entropy (cal/Kmol) = 221.392
E(RPBE1PBE) (a.u.) = -2955.71004236
Optimised cartesian coordinates (Angstrom):
P -2.267164 -0.163283 0.157505
C -2.416096 -0.012324 2.026361
C -1.225323 -0.727123 2.665786
H -3.379131 -0.396250 2.420864
H -2.378030 1.072335 2.268484
H -1.191931 -0.527439 3.762928
H -1.310595 -1.826306 2.536608
N 0.038783 -0.289564 2.022066
C 1.227005 -0.983326 2.583087
C 2.490207 -0.382264 1.968028
H 1.138473 -2.065788 2.351949
H 1.247478 -0.880692 3.694036
H 2.606722 0.663772 2.323850
H 3.398364 -0.942898 2.267183
P 2.255011 -0.277539 0.104983
Mn -0.015390 -0.325315 -0.128539
C -0.052212 -2.083155 -0.301912
O -0.084991 -3.258859 -0.480764
C -0.032324 -0.036133 -1.857446
O -0.047307 0.194268 -3.021864
C -3.214264 1.371224 -0.439804
C -4.561277 1.611776 0.261478
C -3.363434 1.429837 -1.970520
H -2.510353 2.182348 -0.147438
H -4.468993 1.675070 1.364220
H -5.000064 2.571684 -0.087557
H -5.299979 0.816404 0.024927
H -2.412398 1.218317 -2.498589
H -4.127020 0.711411 -2.335302
H -3.703713 2.442758 -2.275959
C -3.440280 -1.583543 -0.312820
C -3.402541 -2.762919 0.675130
C -3.180721 -2.074984 -1.750192
H -4.457822 -1.132966 -0.270139
H -3.688686 -2.472172 1.705971
H -4.123402 -3.542639 0.346575
H -2.400325 -3.236509 0.705932
H -3.182490 -1.255951 -2.494841
H -2.205063 -2.595013 -1.821911

H -3.970564 -2.798922 -2.046207
C 3.227046 1.303445 -0.276860
C 3.150401 1.720828 -1.754300
C 4.678050 1.311433 0.234171
H 2.645742 2.035920 0.326952
H 2.115155 1.690924 -2.147305
H 3.532925 2.757006 -1.878314
H 3.777754 1.066839 -2.396228
H 4.758158 1.049812 1.308783
H 5.324697 0.612795 -0.338068
H 5.113562 2.326586 0.109796
C 3.339198 -1.654463 -0.627180
C 2.970745 -1.916621 -2.099389
C 3.314233 -2.960464 0.186293
H 4.374505 -1.248043 -0.589071
H 2.966720 -0.993076 -2.711318
H 3.702478 -2.619224 -2.553639
H 1.965117 -2.376290 -2.181307
H 3.665165 -2.824255 1.229073
H 2.300646 -3.407963 0.211707
H 3.991108 -3.703497 -0.288670
H 0.009009 1.346141 -0.007704
C 0.003430 2.838557 1.159082
H 0.174027 0.726957 2.235715
O 0.693485 2.522006 2.145649
H -1.117202 2.694321 1.176028
C 0.457887 3.836385 0.088382
H 0.408908 4.834360 0.586543
H 1.534464 3.669391 -0.128889
N -0.312178 3.886595 -1.146580
H -1.282054 4.173339 -0.955773
H -0.368441 2.937092 -1.544567

TS-2
Frequencies, energies and thermodynamic properties:
Lowest Vibrational Mode (1/cm) = -156.8437
2nd Lowest Vibrational Mode (1/cm) = 14.9072
E(RB-P86) (a.u.) = -3089.95344574
Thermal correction to Enthalpy (a.u.) = 0.708617
Thermal correction to Gibbs Free Energy (a.u.) = 0.593510
Total Entropy (cal/Kmol) = 242.264
E(RPBE1PBE) (a.u.) = -3089.57608773
Optimised cartesian coordinates (Angstrom):
P 2.589425 -0.863414 0.161718
C 2.341970 -1.529013 1.901865
C 1.426174 -0.568979 2.660876
H 3.290495 -1.706392 2.447787
H 1.824307 -2.504915 1.784943
H 1.098568 -1.025098 3.625214
H 1.947228 0.380659 2.905476
N 0.245972 -0.244084 1.817877
C -0.746341 0.590179 2.543091
C -1.932862 0.904327 1.631891
H -0.234670 1.521390 2.865393
H -1.088460 0.067236 3.467552
H -2.492820 -0.030117 1.404592
H -2.635059 1.615452 2.114228
P -1.272094 1.510615 -0.018602
Mn 0.753445 0.472400 -0.132965
C 1.633122 1.921678 0.352971
O 2.226430 2.922840 0.603300
C 1.042097 0.768322 -1.838117
O 1.240738 0.948377 -2.993609
C 2.822636 -2.485334 -0.801746
C 3.946661 -3.395380 -0.277028
C 2.923778 -2.280243 -2.322201
H 1.856881 -2.993859 -0.588297
H 3.876414 -3.578938 0.814432
H 3.894508 -4.384566 -0.781521
H 4.953813 -2.979390 -0.491027
H 2.129143 -1.612209 -2.709369
H 3.903833 -1.845746 -2.611402
H 2.836548 -3.257212 -2.844833
C 4.336817 -0.115454 0.154655
C 4.684071 0.654900 1.440816
C 4.561574 0.760300 -1.092312
H 5.016194 -0.994192 0.093419
H 4.629797 0.020044 2.348120
H 5.727072 1.033720 1.372694
H 4.024211 1.533290 1.584927
H 4.303862 0.238422 -2.035003
H 3.953923 1.685905 -1.044594
H 5.629950 1.060731 -1.154954
C -2.694941 1.013690 -1.171192
C -2.326675 1.034296 -2.663092
C -4.018826 1.753257 -0.911551
H -2.837900 -0.045072 -0.857416
H -1.391787 0.478459 -2.868892
H -3.141571 0.568590 -3.259059
H -2.196564 2.068116 -3.047647
H -4.297446 1.779757 0.162394
H -3.993409 2.800465 -1.280608
H -4.844249 1.243461 -1.454389
C -1.414662 3.402006 0.075521
C -0.720032 4.066333 -1.127212

C -0.922241 3.995975 1.406895
H -2.505731 3.603653 0.004720
H -1.092226 3.681765 -2.098090
H -0.900540 5.163130 -1.112165
H 0.376386 3.905203 -1.096310
H -1.482292 3.603774 2.280092
H 0.158384 3.813661 1.568532
H -1.072804 5.097544 1.395661
H -0.168570 -0.840032 -0.653754
C -0.788429 -2.594747 -0.329664
H -0.210124 -1.158859 1.593899
O -0.663084 -2.775486 0.901952
H 0.040103 -2.836202 -1.041484
C -2.166076 -2.588840 -0.976430
H -2.183581 -1.916860 -1.862536
H -2.260890 -3.632693 -1.396379
H -2.907806 -2.656709 0.888016
N -3.209289 -2.270270 -0.021116
C -4.535983 -2.772115 -0.377024
H -4.539179 -3.882093 -0.554922
H -4.842938 -2.306434 -1.342330
C -5.578474 -2.434810 0.703435
H -6.539592 -2.920078 0.425417
H -5.263357 -2.917680 1.658440
N -5.824538 -1.013813 0.964047
H -4.916042 -0.555172 1.127848
H -6.185952 -0.572683 0.105100

TS-3
Frequencies, energies and thermodynamic properties:
Lowest Vibrational Mode (1/cm) = -153.8080
2nd Lowest Vibrational Mode (1/cm) = 14.1793
E(RB-P86) (a.u.) = -3223.82332164
Thermal correction to Enthalpy (a.u.) = 0.784381
Thermal correction to Gibbs Free Energy (a.u.) = 0.660470
Total Entropy (cal/Kmol) = 260.794
E(RPBE1PBE) (a.u.) = -3223.44220498
Optimised cartesian coordinates (Angstrom):
P -1.042064 2.301583 0.234817
C -0.851630 2.395392 2.103302
C -1.693313 1.285035 2.733335
H -1.114082 3.389319 2.520177
H 0.223931 2.225792 2.328157
H -1.483768 1.207964 3.826492
H -2.776416 1.502094 2.624014
N -1.418270 -0.010291 2.062748
C -2.249900 -1.114749 2.606769
C -1.799683 -2.432270 1.976622
H -3.313835 -0.896582 2.375557
H -2.153793 -1.161959 3.717573
H -0.781586 -2.683360 2.342801
H -2.472667 -3.268862 2.251369
P -1.640835 -2.182139 0.118769
Mn -1.453877 0.084757 -0.084713
C -3.198112 0.310201 -0.250412
O -4.365081 0.470335 -0.416976
C -1.166063 0.100984 -1.813950
O -0.933168 0.112362 -2.978580
C 0.573154 3.082890 -0.383182
C 0.998446 4.354853 0.369450
C 0.586382 3.308762 -1.905637
H 1.311629 2.275543 -0.177415
H 1.111419 4.194183 1.460407
H 1.981881 4.704542 -0.013272
H 0.279747 5.189113 0.219194
H 0.241933 2.418617 -2.468604
H -0.045124 4.172695 -2.201531
H 1.622288 3.536276 -2.236989
C -2.338255 3.627636 -0.188056
C -3.497346 3.697124 0.821882
C -2.882607 3.449551 -1.618826
H -1.779986 4.590305 -0.137609
H -3.160443 3.931637 1.851643
H -4.202307 4.501948 0.520386
H -4.073678 2.749883 0.844890
H -2.083030 3.384395 -2.381098
H -3.501074 2.533727 -1.697842
H -3.528009 4.315185 -1.883230
C -0.158146 -3.301923 -0.251209
C 0.294319 -3.242287 -1.718718
C -0.309711 -4.755008 0.230435
H 0.617644 -2.808128 0.376901
H 0.389751 -2.201267 -2.085358
H 1.283197 -3.737102 -1.832581
H -0.412361 -3.776657 -2.388416
H -0.605641 -4.826841 1.296743
H -1.055088 -5.317853 -0.370849
H 0.659254 -5.289041 0.122147
C -3.108334 -3.110067 -0.651925
C -3.309486 -2.689410 -2.119420
C -4.417331 -2.967003 0.144234
H -2.809021 -4.181661 -0.629072
H -2.382323 -2.772293 -2.720279
H -4.079720 -3.333321 -2.596473
H -3.657741 -1.638928 -2.185821

H -4.333732 -3.349826 1.181281
H -4.758926 -1.913447 0.184877
H -5.218327 -3.554904 -0.354395
H 0.208508 -0.121139 0.050222
C 1.636889 -0.270080 1.147424
H -0.422506 -0.276041 2.264347
O 1.279018 -0.975663 2.116735
H 1.610829 0.853324 1.219500
C 2.597723 -0.757401 0.076076
H 3.616403 -0.701406 0.531090
H 2.414200 -1.843160 -0.133578
N 2.602997 0.102492 -1.097754
H 1.677198 0.059551 -1.544509
C 3.651348 -0.168306 -2.082202
H 3.350306 0.319956 -3.032630
H 3.752961 -1.263522 -2.317505
C 5.034247 0.380171 -1.702100
H 4.917244 1.441003 -1.386627
H 5.671855 0.395627 -2.627874
N 5.668480 -0.355240 -0.605246
H 5.777115 -1.341618 -0.886160
C 6.974035 0.172744 -0.211381
H 6.839239 1.241264 0.076452
H 7.718392 0.173138 -1.054651
C 7.560468 -0.601166 0.983694
H 8.572945 -0.196007 1.202175
H 7.721297 -1.661615 0.677104
N 6.767620 -0.592470 2.216851
H 6.661375 0.383573 2.533679
H 5.808546 -0.886452 1.976371

TS-4
Frequencies, energies and thermodynamic properties:
Lowest Vibrational Mode (1/cm) = -592.1101
2nd Lowest Vibrational Mode (1/cm) = 17.7598
E(RB-P86) (a.u.) = -2957.26977943
Thermal correction to Enthalpy (a.u.) = 0.649857
Thermal correction to Gibbs Free Energy (a.u.) = 0.539779
Total Entropy (cal/Kmol) = 231.679
E(RPBE1PBE) (a.u.) = -2956.89121891
Optimised cartesian coordinates (Angstrom):
P 2.302407 -0.188629 -0.015360
C 2.412889 1.012805 -1.447905
C 1.147987 0.851520 -2.295958
H 3.341072 0.895900 -2.042147
H 2.408543 2.024746 -0.992657
H 1.079790 1.701123 -3.021062
H 1.200848 -0.078954 -2.912909
N -0.037109 0.840488 -1.421083
C -1.270785 0.805717 -2.219272
C -2.499336 0.794774 -1.307189
H -1.284765 -0.090396 -2.887594
H -1.317440 1.694399 -2.897628
H -2.577258 1.767475 -0.774334
H -3.442264 0.650241 -1.872783
P -2.226473 -0.505625 0.012404
Mn 0.056323 -0.650745 0.104420
C 0.143681 -1.958158 -1.068023
O 0.202830 -2.861519 -1.829111
C 0.146022 -1.819527 1.430616
O 0.202597 -2.622908 2.296407
C 3.013089 0.848495 1.401621
C 4.429418 1.385220 1.135935
C 2.915542 0.168738 2.777019
H 2.298921 1.704288 1.376672
H 4.512945 1.906745 0.160805
H 4.707551 2.116544 1.925318
H 5.191783 0.577638 1.161472
H 1.904550 -0.240573 2.973834
H 3.645470 -0.661685 2.880665
H 3.144696 0.903863 3.578143
C 3.583374 -1.540019 -0.351755
C 3.631385 -1.976997 -1.826827
C 3.372496 -2.752456 0.572662
H 4.558512 -1.065507 -0.100529
H 3.867775 -1.141354 -2.515303
H 4.426362 -2.742538 -1.957546
H 2.675185 -2.434965 -2.150486
H 3.313185 -2.473104 1.642915
H 2.437628 -3.291047 0.315587
H 4.215434 -3.467086 0.456348
C -3.110971 0.269145 1.501950
C -2.975464 -0.553504 2.794322
C -4.574169 0.659460 1.232889
H -2.518503 1.204049 1.629357
H -1.928527 -0.856114 2.994882
H -3.327472 0.045872 3.660880
H -3.596997 -1.472783 2.762186
H -4.691160 1.293047 0.330611
H -5.224303 -0.232861 1.113321
H -4.973070 1.235578 2.095375
C -3.298363 -1.989401 -0.468564
C -2.897532 -3.245698 0.326390
C -3.297766 -2.273773 -1.981232
H -4.330861 -1.695817 -0.174192

H -2.870766 -3.073851 1.420159
H -3.625291 -4.062270 0.131030
H -1.895584 -3.608622 0.019842
H -3.668113 -1.417685 -2.579691
H -2.287351 -2.545870 -2.347179
H -3.968384 -3.134313 -2.192695
H -0.099884 0.380685 1.485649
H 0.041714 0.998565 0.938102
H 0.069566 1.937202 -0.655112
O 0.343305 2.738020 0.184455
C -0.555924 3.799748 0.327223
H -0.699548 4.057757 1.411813
H -1.587980 3.551677 -0.052522
C -0.092268 5.084152 -0.397466
H 0.015625 4.860611 -1.483537
H 0.929712 5.336827 -0.033946
N -0.958451 6.258894 -0.239718
H -1.909451 6.020086 -0.560544
H -1.069727 6.459060 0.766249

TS-5
Frequencies, energies and thermodynamic properties:
Lowest Vibrational Mode (1/cm) = -469.8819
2nd Lowest Vibrational Mode (1/cm) = 32.3217
E(RB-P86) (a.u.) = -2747.01460198
Thermal correction to Enthalpy (a.u.) = 0.549315
Thermal correction to Gibbs Free Energy (a.u.) = 0.455371
Total Entropy (cal/Kmol) = 197.723
E(RPBE1PBE) (a.u.) = -2746.62561705
Optimised cartesian coordinates (Angstrom):
P 2.264754 0.034074 -0.318748
C 2.450063 -1.126113 -1.778883
C 1.204990 -2.018569 -1.820900
H 3.392737 -1.709988 -1.752939
H 2.476937 -0.484425 -2.686119
H 1.197680 -2.584739 -2.789533
H 1.284039 -2.804437 -1.017062
N 0.000000 -1.219985 -1.694110
H 0.000000 0.428956 -1.685303
C -1.204990 -2.018569 -1.820901
C -2.450064 -1.126113 -1.778884
H -1.284040 -2.804437 -1.017063
H -1.197680 -2.584739 -2.789533
H -2.476938 -0.484425 -2.686120
H -3.392737 -1.709988 -1.752938
P -2.264754 0.034075 -0.318749
Mn 0.000000 0.085126 -0.009919
C -0.000001 -1.176593 1.221320
O -0.000001 -2.023899 2.048312
C 0.000001 1.376409 1.199815
O 0.000002 2.224481 2.026718
C 3.092606 1.608568 -0.983599
C 4.518082 1.391085 -1.520009
C 3.037458 2.799870 -0.012544
H 2.425483 1.841219 -1.845415
H 4.578673 0.569375 -2.261758
H 4.876281 2.316198 -2.021162
H 5.236630 1.169512 -0.702563
H 2.023126 2.963457 0.400673
H 3.736733 2.665073 0.839273
H 3.345397 3.728703 -0.539014
C 3.441050 -0.595215 1.023968
C 3.448635 -2.129191 1.149007
C 3.135809 0.063878 2.381825
H 4.451880 -0.270721 0.688952
H 3.749413 -2.635084 0.209890
H 4.177229 -2.431112 1.932180
H 2.456231 -2.519256 1.452299
H 3.105197 1.169644 2.327058
H 2.158995 -0.278953 2.779080
H 3.917213 -0.216073 3.120782
C -3.092606 1.608568 -0.983600
C -3.037451 2.799874 -0.012550
C -4.518084 1.391087 -1.520004
H -2.425486 1.841215 -1.845420
H -2.023117 2.963459 0.400663
H -3.345390 3.728706 -0.539021
H -3.736723 2.665081 0.839271
H -4.578682 0.569371 -2.261746
H -5.236631 1.169522 -0.702553
H -4.876281 2.316197 -2.021162
C -3.441051 -0.595215 1.023966
C -3.135814 0.063880 2.381823
C -3.448634 -2.129191 1.149007
H -4.451881 -0.270723 0.688948
H -3.105205 1.169646 2.327055
H -3.917217 -0.216073 3.120780
H -2.158999 -0.278948 2.779080
H -3.749409 -2.635086 0.209891
H -2.456231 -2.519255 1.452302
H -4.177230 -2.431112 1.932179
H 0.000001 1.248388 -1.308108

TS-6
Frequencies, energies and thermodynamic properties:

Lowest Vibrational Mode (1/cm) = -422.2205
2nd Lowest Vibrational Mode (1/cm) = 25.4904
E(RB-P86) (a.u.) = -3089.92066736
Thermal correction to Enthalpy (a.u.) = 0.707881
Thermal correction to Gibbs Free Energy (a.u.) = 0.593715
Total Entropy (cal/Kmol) = 240.284
E(RPBE1PBE) (a.u.) = -3089.54590512
Optimised cartesian coordinates (Angstrom):

P	2.195349	-0.900796	0.025686
C	2.597764	0.028255	-1.559014
C	1.339495	0.116899	-2.424910
H	3.435087	-0.424812	-2.126849
H	2.928819	1.043237	-1.260258
H	1.481945	0.863659	-3.241468
H	1.130675	-0.859107	-2.911372
N	0.145806	0.469381	-1.605748
C	-1.072661	0.593308	-2.451892
C	-2.288761	0.898715	-1.577448
H	-1.206959	-0.361496	-3.001160
H	-0.929439	1.393959	-3.214789
H	-2.161952	1.903989	-1.118878
H	-3.223873	0.918281	-2.173997
P	-2.320380	-0.330206	-0.159258
Mn	-0.081595	-0.735015	0.124206
C	-0.236697	-2.297440	-0.668406
O	-0.337995	-3.367811	-1.177762
C	-0.234082	-1.376427	1.755501
O	-0.337603	-1.785500	2.863661
C	3.318202	-0.012216	1.274665
C	4.794626	0.095630	0.856570
C	3.175824	-0.559292	2.704898
H	2.885187	1.012314	1.262233
H	4.926944	0.532667	-0.154021
H	5.338574	0.749933	1.571770
H	5.305666	-0.890261	0.872254
H	2.116725	-0.657704	3.014876
H	3.658571	-1.553359	2.812410
H	3.679702	0.123611	3.422262
C	3.045096	-2.595275	-0.139112
C	3.019998	-3.179373	-1.562658
C	2.484763	-3.602378	0.883843
H	4.107192	-2.390794	0.121413
H	3.522099	-2.525646	-2.303838
H	3.561913	-4.149946	-1.569494
H	1.986668	-3.377811	-1.909292
H	2.461035	-3.198918	1.915114
H	1.453855	-3.909771	0.619455
H	3.117111	-4.516366	0.894373
C	-3.366071	0.575107	1.142054
C	-3.660975	-0.274174	2.391492
C	-4.646367	1.232558	0.598380
H	-2.671498	1.377759	1.459655
H	-2.756996	-0.784256	2.779214
H	-4.053401	0.379670	3.199602
H	-4.437046	-1.042143	2.192974
H	-4.455282	1.917384	-0.252571
H	-5.392848	0.479662	0.267300
H	-5.126355	1.832578	1.401161
C	-3.518941	-1.693787	-0.733073
C	-3.442785	-2.950263	0.156719
C	-3.375197	-2.070352	-2.218322
H	-4.523265	-1.232282	-0.599786
H	-3.524148	-2.722230	1.236491
H	-4.276663	-3.638172	-0.102360
H	-2.495131	-3.499385	-0.004574
H	-3.537428	-1.210003	-2.898329
H	-2.382778	-2.513926	-2.435579
H	-4.138844	-2.835354	-2.477646
C	0.471787	2.426845	0.998839
H	0.296715	1.424361	-1.183045
C	-0.655006	2.853804	1.966659
H	1.416154	2.187282	1.546145
H	-1.063873	1.974367	2.506880
H	-0.133724	3.459496	2.745272
N	0.557571	3.029649	-0.181667
C	1.862864	3.412481	-0.707189
H	2.724375	3.052071	-0.091254
H	1.996478	3.031605	-1.746630
H	0.124450	0.848205	0.851304
C	1.871933	4.957890	-0.773552
H	1.998496	5.360233	0.265355
H	2.732911	5.322954	-1.376943
O	0.662681	5.403347	-1.366587
H	0.029160	4.705912	-1.051390
N	-1.737822	3.667757	1.428993
H	-1.318176	4.449070	0.903688
H	-2.255881	3.139010	0.712970

TS-7
Frequencies, energies and thermodynamic properties:
Lowest Vibrational Mode (1/cm) = -432.9661
2nd Lowest Vibrational Mode (1/cm) = 13.0324
E(RB-P86) (a.u.) = -3223.79577031
Thermal correction to Enthalpy (a.u.) = 0.783491
Thermal correction to Gibbs Free Energy (a.u.) = 0.659473

Total Entropy (cal/Kmol) = 261.017
E(RPBE1PBE) (a.u.) = -3223.41747474
Optimised cartesian coordinates (Angstrom):

P	0.310441	2.369372	-0.000659
C	-0.357903	2.372457	-1.758477
C	0.333776	1.269700	-2.562142
H	-0.245984	3.353877	-2.261708
H	-1.445612	2.172493	-1.689152
H	-0.214779	1.083572	-3.516074
H	1.364866	1.577378	-2.836737
N	0.444221	0.008946	-1.775624
C	1.088110	-1.064463	-2.583853
C	1.293449	-2.324473	-1.743574
H	2.058128	-0.670886	-2.952925
H	0.464697	-1.289134	-3.481416
H	0.313188	-2.769671	-1.465350
H	1.871623	-3.087443	-2.305807
P	2.089583	-1.838890	-0.117910
Mn	1.256923	0.297996	0.166225
C	2.822873	0.980906	-0.243954
O	3.891579	1.459332	-0.459704
C	1.544535	0.392759	1.897064
O	1.716335	0.459324	3.069088
C	-1.247632	2.836869	0.984064
C	-1.964408	4.109536	0.501896
C	-1.019093	2.861714	2.504501
H	-1.905785	1.969263	0.757890
H	-2.175132	4.101163	-0.586932
H	-2.939825	4.211265	1.025269
H	-1.381857	5.027110	0.729856
H	-0.490520	1.957771	2.865941
H	-0.429969	3.750027	2.815299
H	-1.995279	2.921612	3.032388
C	1.362212	3.949407	0.141146
C	2.179144	4.280574	-1.120566
C	2.263217	3.913547	1.390618
H	0.612107	4.759763	0.276968
H	1.544343	4.424246	-2.017985
H	2.730026	5.232765	-0.960056
H	2.931699	3.498437	-1.341724
H	1.707945	3.654288	2.313219
H	3.082105	3.176986	1.272051
H	2.727196	4.911491	1.546675
C	1.608631	-3.300054	0.988218
C	1.900286	-3.085281	2.482717
C	2.126365	-4.668080	0.509992
H	0.505549	-3.298116	0.828317
H	1.553302	-2.098508	2.845862
H	1.384600	-3.868000	3.080971
H	2.983518	-3.168002	2.715702
H	1.924944	-4.853050	-0.565461
H	3.216471	-4.796031	0.682383
H	1.616762	-5.475797	1.079882
C	3.947753	-2.132616	-0.407408
C	4.798061	-1.621827	0.770607
C	4.476488	-1.607444	-1.753606
H	4.030442	-3.241223	-0.435180
H	4.461219	-2.028754	1.744590
H	5.857507	-1.929046	0.631262
H	4.776906	-0.516249	0.835881
H	3.952178	-2.058912	-2.620213
H	4.404204	-0.504949	-1.830027
H	5.551439	-1.873964	-1.853598
C	-1.867270	-0.810386	0.402131
H	-0.528896	-0.337904	-1.557258
C	-1.791994	-2.130307	1.171675
H	-2.226497	0.029190	1.045889
H	-1.020442	-2.082118	1.970729
H	-2.779346	-2.194560	1.702264
N	-2.257724	-0.874611	-0.867936
C	-3.118515	0.168934	-1.397066
H	-3.265865	1.017232	-0.679040
H	-2.702322	0.605604	-2.335748
H	-0.337910	-0.331846	0.555207
C	-4.502317	-0.420680	-1.774144
H	-5.126777	0.379365	-2.230275
H	-4.342298	-1.195703	-2.555754
N	-1.550134	-3.249892	0.267789
H	-1.927735	-4.122649	0.656663
H	-2.063989	-3.036088	-0.603838
N	-5.255381	-1.032412	-0.679993
H	-4.677133	-1.773910	-0.256665
C	-5.763517	-0.132191	0.363270
H	-6.173947	0.767775	-0.145736
H	-4.996118	0.223862	1.093939
C	-6.905567	-0.862589	1.090707
H	-6.469231	-1.649854	1.762607
H	-7.460661	-0.156172	1.746802
O	-7.791797	-1.417604	0.135984
H	-7.162380	-1.633834	-0.602425

TS-8
Frequencies, energies and thermodynamic properties:
Lowest Vibrational Mode (1/cm) = -678.2137
2nd Lowest Vibrational Mode (1/cm) = 16.7656

E(RB-P86) (a.u.) = -3223.77794967
Thermal correction to Enthalpy (a.u.) = 0.780805
Thermal correction to Gibbs Free Energy (a.u.) = 0.660196
Total Entropy (cal/Kmol) = 253.844
E(RPBE1PBE) (a.u.) = -3223.39310787
Optimised cartesian coordinates (Angstrom):

P	0.698235	2.334963	0.030273
C	-0.720052	2.277542	-1.192382
C	-0.469269	1.103604	-2.137812
H	-0.872355	3.228494	-1.742984
H	-1.638988	2.082197	-0.596190
H	-1.369559	0.918524	-2.773121
H	0.367029	1.324391	-2.835960
N	-0.139066	-0.114822	-1.354541
C	0.039031	-1.271812	-2.273835
C	0.352422	-2.544937	-1.486646
H	0.857583	-1.038430	-2.989261
H	-0.887148	-1.418123	-2.881012
H	-0.560298	-2.894651	-0.959938
H	0.677586	-3.367536	-2.155527
P	1.609784	-2.128001	-0.157487
Mn	1.336694	0.136574	0.074186
C	2.720781	0.462033	-0.942919
O	3.674959	0.693719	-1.617523
C	2.227440	0.254490	1.591970
O	2.834079	0.333654	2.611024
C	-0.120904	3.155804	1.532692
C	-0.932792	4.422433	1.212816
C	0.841246	3.390415	2.710379
H	-0.828149	2.354030	1.835221
H	-1.695012	4.258962	0.424695
H	-1.469683	4.763540	2.124573
H	-0.281780	5.263031	0.890894
H	1.459346	2.499204	2.936633
H	1.523877	4.244449	2.517874
H	0.261169	3.643253	3.624173
C	1.852862	3.701332	-0.613065
C	2.028358	3.691546	-2.142027
C	3.220982	3.675376	0.095510
H	1.332060	4.645998	-0.337122
H	1.069953	3.815251	-2.685319
H	2.683330	4.537949	-2.442834
H	2.515765	2.759421	-2.491537
H	3.134799	3.664966	1.199198
H	3.810137	2.787715	-0.207317
H	3.804667	4.578525	-0.186239
C	1.109947	-3.350949	1.213171
C	1.957521	-3.221522	2.489996
C	1.001328	-4.816534	0.757101
H	0.082179	-2.999170	1.454509
H	2.070621	-2.169540	2.817951
H	1.481251	-3.788972	3.318379
H	2.973252	-3.647476	2.350076
H	0.355687	-4.944465	-0.135179
H	1.995434	-5.254774	0.526601
H	0.561067	-5.430365	1.572546
C	3.256749	-2.849501	-0.773193
C	4.447209	-2.312733	0.044240
C	3.488875	-2.654281	-2.281979
H	3.165146	-3.942016	-0.583021
H	4.302588	-2.423826	1.136705
H	5.370944	-2.866705	-0.230409
H	4.624043	-1.239346	-0.165211
H	2.696857	-3.121160	-2.901411
H	3.560872	-1.581756	-2.550329
H	4.450114	-3.132287	-2.570758
H	-0.223733	-0.190178	1.082441
C	-1.391519	-0.631135	1.473559
H	-1.122529	-0.376127	-0.733513
C	-2.245186	-0.850623	0.299567
H	-2.295654	-1.917294	-0.027614
H	-1.006707	-1.563634	1.931371
N	-3.487118	-0.116258	0.223497
N	-1.890495	0.249486	2.486589
H	-1.160459	0.612986	3.109688
C	-3.941378	0.117166	-1.158543
H	-3.111570	0.633583	-1.681703
H	-4.115934	-0.841935	-1.712484
H	-2.422137	1.024984	2.068660
C	-4.530389	-0.588209	1.168692
H	-4.094628	-0.530719	2.188986
H	-5.378012	0.132611	1.155029
C	-5.083190	-2.012323	0.977867
H	-4.247987	-2.747382	1.063129
H	-5.752854	-2.213923	1.859289
C	-5.203023	0.991411	-1.296315
H	-5.232186	1.710545	-0.450466
H	-5.104615	1.607114	-2.219981
N	-6.458990	0.205042	-1.299500
H	-7.256480	0.798463	-1.032966
H	-6.664726	-0.111960	-2.257939
O	-5.745605	-2.238538	-0.251831
H	-6.151881	-1.361577	-0.536451

----
toluene/1

Frequencies, energies and thermodynamic properties:
Lowest Vibrational Mode (1/cm) = 195.8852
2nd Lowest Vibrational Mode (1/cm) = 291.4916
E(RB-P86) (a.u.) = -210.234445773
Thermal correction to Enthalpy (a.u.) = 0.101310
Thermal correction to Gibbs Free Energy (a.u.) = 0.068403
Total Entropy (cal/Kmol) = 69.260
E(RPBE1PBE) (a.u.) = -210.240408374
Optimised cartesian coordinates (Angstrom):
O -1.348703 -0.687829 -0.161110
C -0.825201 0.561346 0.240520
C 0.630017 0.685668 -0.244588
N 1.323830 -0.561383 0.115811
H -0.539386 -1.261126 -0.116963
H -0.850330 0.687776 1.357533
H -1.448184 1.379294 -0.185054
H 1.100673 1.618306 0.152524
H 0.631105 0.762222 -1.353184
H 2.169490 -0.698012 -0.450661
H 1.630551 -0.538227 1.098409
----
toluene/10
Frequencies, energies and thermodynamic properties:
Lowest Vibrational Mode (1/cm) = 43.1642
2nd Lowest Vibrational Mode (1/cm) = 72.8550
E(RB-P86) (a.u.) = -342.887752286
Thermal correction to Enthalpy (a.u.) = 0.152661
Thermal correction to Gibbs Free Energy (a.u.) = 0.108721
Total Entropy (cal/Kmol) = 92.481
E(RPBE1PBE) (a.u.) = -342.888230198
Optimised cartesian coordinates (Angstrom):
N -2.679739 0.651025 -0.709865
C -2.292217 0.008385 0.539592
C -0.885543 -0.550400 0.622269
N -0.069903 -0.477884 -0.362157
C 1.270440 -1.018154 -0.202169
C 2.310545 0.092971 -0.419256
O 2.207907 1.125188 0.548823
H -2.501539 -0.016621 -1.475799
H -2.005564 1.407790 -0.900830
H -2.998457 -0.825078 0.769897
H -2.428160 0.719386 1.389082
H -0.605370 -1.046308 1.590168
H 1.432093 -1.802960 -0.976335
H 1.429436 -1.485922 0.802905
H 2.206650 0.485049 -1.461470
H 3.329937 -0.337797 -0.325704
H 1.305863 1.492161 0.459037
----
toluene/11
Frequencies, energies and thermodynamic properties:
Lowest Vibrational Mode (1/cm) = 48.5223
2nd Lowest Vibrational Mode (1/cm) = 78.1198
E(RB-P86) (a.u.) = -418.072409193
Thermal correction to Enthalpy (a.u.) = 0.158104
Thermal correction to Gibbs Free Energy (a.u.) = 0.113155
Total Entropy (cal/Kmol) = 94.602
E(RPBE1PBE) (a.u.) = -418.082738522
Optimised cartesian coordinates (Angstrom):
N 3.190152 -0.227679 -0.154896
C 1.915385 -0.821103 0.271430
C 0.845889 0.293256 0.329101
O 1.012787 1.130823 -0.810992
N -0.467935 -0.287570 0.414816
C -1.560213 0.653258 0.550917
C -2.864076 0.099947 0.027962
O -2.955925 -0.932411 -0.615412
H 3.693904 0.178250 0.645830
H 3.813964 -0.934046 -0.563017
H 1.943577 -1.369473 1.241585
H 1.599306 -1.548844 -0.507758
H 0.997272 0.895855 1.265275
H 1.982273 1.013118 -1.007024
H -0.658178 -0.888839 -0.401861
H -1.413839 1.612586 -0.017871
H -1.719671 0.970715 1.610590
H -3.770922 0.737979 0.249585
----
toluene/12
Frequencies, energies and thermodynamic properties:
Lowest Vibrational Mode (1/cm) = 33.7267
2nd Lowest Vibrational Mode (1/cm) = 81.3843
E(RB-P86) (a.u.) = -341.678512076
Thermal correction to Enthalpy (a.u.) = 0.128335
Thermal correction to Gibbs Free Energy (a.u.) = 0.085201
Total Entropy (cal/Kmol) = 90.781
E(RPBE1PBE) (a.u.) = -341.673735410
Optimised cartesian coordinates (Angstrom):
N -2.597106 0.755974 0.569325
C -2.202597 -0.098646 -0.542126
C -0.792960 -0.651596 -0.515071
N 0.007738 -0.401959 0.451083
C 1.350728 -0.920402 0.384069
C 2.388895 0.140594 0.031568
O 2.141011 1.257928 -0.374159

H -1.918031 1.530349 0.626329
H -2.430087 0.234614 1.443472
H -2.328470 0.449999 -1.505842
H -2.905034 -0.962280 -0.627437
H -0.496709 -1.294659 -1.387307
H 1.501252 -1.740767 -0.372317
H 1.654539 -1.355951 1.364959
H 3.455628 -0.222527 0.157919

----

toluene/13

Frequencies, energies and thermodynamic properties:
Lowest Vibrational Mode (1/cm) = 36.0753
2nd Lowest Vibrational Mode (1/cm) = 51.8353
E(RB-P86) (a.u.) = -553.151120609
Thermal correction to Enthalpy (a.u.) = 0.257882
Thermal correction to Gibbs Free Energy (a.u.) = 0.203270
Total Entropy (cal/Kmol) = 114.939
E(RPBE1PBE) (a.u.) = -553.160956162

Optimised cartesian coordinates (Angstrom):
N 3.479493 -0.120781 -0.974468
C 3.137720 0.300578 0.391008
C 2.018755 -0.594526 0.939564
N 0.862434 -0.510548 0.043212
C -0.186111 -1.506223 0.262682
C -1.538964 -1.075588 -0.343681
O -2.323914 -2.275681 -0.360131
N -2.231076 -0.031169 0.370017
C -2.370533 1.335238 -0.123146
C -1.234013 2.314846 0.230835
O -0.021243 2.052990 -0.429048
H 4.111148 -0.933497 -0.951188
H 3.989910 0.622894 -1.466552
H 2.749423 1.340606 0.342392
H 3.993177 0.301520 1.110855
H 1.729765 -0.261243 1.960908
H 2.397216 -1.645928 1.041150
H 1.253481 -0.595556 -0.912208
H -0.338956 -1.643691 1.356574
H 0.080673 -2.511904 -0.142970
H -1.368457 -0.698734 -1.384738
H -3.246346 -1.952268 -0.398705
H -2.110597 -0.088167 1.387735
H -3.328632 1.758649 0.263751
H -2.469064 1.292873 -1.230600
H -1.126466 2.327222 1.351667
H -1.563719 3.342278 -0.052164
H 0.271618 1.118016 -0.177369

----

toluene/14

Frequencies, energies and thermodynamic properties:
Lowest Vibrational Mode (1/cm) = 63.0033
2nd Lowest Vibrational Mode (1/cm) = 82.0370
E(RB-P86) (a.u.) = -476.768043253
Thermal correction to Enthalpy (a.u.) = 0.228555
Thermal correction to Gibbs Free Energy (a.u.) = 0.179997
Total Entropy (cal/Kmol) = 102.197
E(RPBE1PBE) (a.u.) = -476.761626926

Optimised cartesian coordinates (Angstrom):
N -0.172984 1.754697 -0.448645
C -1.267658 1.525811 0.505499
C -2.475448 0.735924 -0.053510
N -2.221258 -0.608078 -0.580072
C -1.636342 -1.567717 0.342311
C -0.121211 -1.615431 0.447015
N 0.647353 -1.067103 -0.416628
C 2.088549 -1.135663 -0.227174
C 2.730212 0.271210 -0.276924
O 2.260475 1.146798 0.718102
H -0.441931 2.448705 -1.159953
H 0.009961 0.855562 -0.936987
H -1.620806 2.504504 0.897739
H -0.843794 0.983849 1.380901
H -2.948871 1.326322 -0.870703
H -3.243232 0.655363 0.751056
H -1.653604 -0.567879 -1.436869
H -1.959597 -2.606122 0.073364
H -2.056372 -1.406712 1.360789
H 0.290541 -2.205221 1.307053
H 2.526016 -1.737248 -1.058032
H 2.357564 -1.637627 0.735942
H 2.581625 0.684752 -1.307373
H 3.829226 0.137128 -0.147359
H 1.409092 1.548822 0.359713

----

toluene/2

Frequencies, energies and thermodynamic properties:
Lowest Vibrational Mode (1/cm) = 120.8465
2nd Lowest Vibrational Mode (1/cm) = 252.4293
E(RB-P86) (a.u.) = -209.026055970
Thermal correction to Enthalpy (a.u.) = 0.076923
Thermal correction to Gibbs Free Energy (a.u.) = 0.043691
Total Entropy (cal/Kmol) = 69.943
E(RPBE1PBE) (a.u.) = -209.028706875

Optimised cartesian coordinates (Angstrom):
O 1.400907 -0.658002 0.000123

C 0.887703 0.446986 0.000073
C -0.615552 0.686659 -0.000153
N -1.464442 -0.492823 -0.000102
H 1.521175 1.389480 0.000119
H -0.840840 1.337747 -0.879478
H -0.841167 1.338039 0.878854
H -1.213572 -1.077092 -0.811385
H -1.214657 -1.076265 0.812106
----
toluene/3
Frequencies, energies and thermodynamic properties:
Lowest Vibrational Mode (1/cm) = 52.9305
2nd Lowest Vibrational Mode (1/cm) = 78.1906
E(RB-P86) (a.u.) = -342.894499800
Thermal correction to Enthalpy (a.u.) = 0.152384
Thermal correction to Gibbs Free Energy (a.u.) = 0.109208
Total Entropy (cal/Kmol) = 90.872
E(RPBE1PBE) (a.u.) = -342.892691252
Optimised cartesian coordinates (Angstrom):
N -2.665062 -0.880137 0.591391
C -2.296649 -0.024597 -0.539042
C -1.068849 0.878496 -0.314670
N 0.103354 0.070918 0.007193
C 1.284060 0.800307 0.396599
C 2.551108 -0.003466 0.205162
O 2.597432 -1.059032 -0.403108
H -2.964533 -0.280256 1.375181
H -1.794563 -1.321934 0.928266
H -3.165823 0.608671 -0.821280
H -2.097105 -0.674322 -1.424016
H -1.270892 1.556809 0.546917
H -0.917668 1.547024 -1.206802
H 0.349451 -0.563435 -0.767091
H 1.242911 1.140627 1.460553
H 1.470867 1.751455 -0.186589
H 3.481842 0.467716 0.641335
----
toluene/5
Frequencies, energies and thermodynamic properties:
Lowest Vibrational Mode (1/cm) = 63.3051
2nd Lowest Vibrational Mode (1/cm) = 78.1636
E(RB-P86) (a.u.) = -344.105343878
Thermal correction to Enthalpy (a.u.) = 0.177074
Thermal correction to Gibbs Free Energy (a.u.) = 0.134186
Total Entropy (cal/Kmol) = 90.264
E(RPBE1PBE) (a.u.) = -344.106806278
Optimised cartesian coordinates (Angstrom):
N -2.794974 0.330458 -0.560732
C -2.140280 -0.686171 0.274011
C -0.800543 -0.149291 0.794371
N 0.029690 0.264066 -0.335086
C 1.218222 1.054789 -0.011104
C 2.364699 0.093263 0.363055
O 2.413862 -0.955673 -0.583204
H -3.232544 1.048215 0.034507
H -3.560137 -0.088104 -1.104620
H -1.939064 -1.573683 -0.364104
H -2.748678 -1.043408 1.141858
H -0.291582 -0.940439 1.388270
H -0.994209 0.699780 1.505767
H -0.596181 0.754135 -0.992158
H 1.047269 1.803364 0.805278
H 1.507424 1.616631 -0.925046
H 2.207307 -0.291317 1.406346
H 3.335531 0.637843 0.371244
H 1.458367 -1.014837 -0.852981
----
toluene/6-B
Frequencies, energies and thermodynamic properties:
Lowest Vibrational Mode (1/cm) = 59.6516
2nd Lowest Vibrational Mode (1/cm) = 76.8637
E(RB-P86) (a.u.) = -553.157872649
Thermal correction to Enthalpy (a.u.) = 0.258431
Thermal correction to Gibbs Free Energy (a.u.) = 0.206893
Total Entropy (cal/Kmol) = 108.470
E(RPBE1PBE) (a.u.) = -553.165315237
Optimised cartesian coordinates (Angstrom):
O 0.025802 -0.213936 1.655661
C 0.674042 -0.746108 0.480584
N 0.106620 -0.301280 -0.779089
C -1.121237 -0.974423 -1.215249
C -2.398272 -0.786660 -0.361371
O -2.309551 -1.341665 0.930669
C 0.325954 1.066346 -1.242105
C -0.426375 2.226407 -0.544382
N -0.017023 2.391116 0.863086
C 2.202063 -0.521252 0.565171
N 2.992289 -1.152647 -0.493150
H 0.013323 0.796657 1.522394
H 0.490227 -1.843323 0.525540
H -0.915595 -2.069707 -1.254195
H -1.337215 -0.650674 -2.259182
H -3.231012 -1.303877 -0.892092
H -2.678087 0.300447 -0.341291
H -1.537434 -0.888649 1.378201

H 0.056453 1.105506 -2.321391
H 1.415880 1.292947 -1.201871
H -1.517454 2.024876 -0.561786
H -0.263980 3.150473 -1.150846
H 0.918926 2.819153 0.918744
H -0.651120 3.035209 1.355017
H 2.531870 -0.902627 1.553759
H 2.417105 0.571702 0.585947
H 2.520519 -0.963401 -1.391682
H 2.927337 -2.176087 -0.387721
----
toluene/6-L
Frequencies, energies and thermodynamic properties:
Lowest Vibrational Mode (1/cm) = 17.6485
2nd Lowest Vibrational Mode (1/cm) = 35.5208
E(RB-P86) (a.u.) = -553.145414855
Thermal correction to Enthalpy (a.u.) = 0.258216
Thermal correction to Gibbs Free Energy (a.u.) = 0.201441
Total Entropy (cal/Kmol) = 119.493
E(RPBE1PBE) (a.u.) = -553.157979540
Optimised cartesian coordinates (Angstrom):
N -1.645828 2.300591 -0.003426
C -2.763202 1.357122 -0.006140
C -2.264725 -0.071149 0.244319
O -3.431447 -0.904548 0.294656
N -1.302884 -0.440696 -0.779292
C -0.531451 -1.671616 -0.565299
C 0.600102 -1.471033 0.452806
N 1.467271 -0.373559 0.037797
C 2.504888 0.014527 0.995853
C 3.526642 0.888446 0.252239
O 3.913433 0.245711 -0.947643
H -1.059689 2.110166 -0.828495
H -2.000258 3.259027 -0.122183
H -3.373706 1.333939 -0.948957
H -3.468544 1.615372 0.811288
H -1.730327 -0.088523 1.222996
H -3.179385 -1.743791 0.723201
H -1.777325 -0.469559 -1.691603
H -0.081359 -1.954841 -1.539722
H -1.161362 -2.539946 -0.241885
H 1.192432 -2.410771 0.520223
H 0.165048 -1.309901 1.475493
H 0.855944 0.429727 -0.180139
H 3.010267 -0.914224 1.343037
H 2.114077 0.536492 1.906525
H 4.425558 1.059311 0.885047
H 3.073355 1.898306 0.059348
H 3.075946 -0.222226 -1.198497
----
toluene/7-B
Frequencies, energies and thermodynamic properties:
Lowest Vibrational Mode (1/cm) = 37.1674
2nd Lowest Vibrational Mode (1/cm) = 69.6137
E(RB-P86) (a.u.) = -476.761494764
Thermal correction to Enthalpy (a.u.) = 0.228866
Thermal correction to Gibbs Free Energy (a.u.) = 0.177696
Total Entropy (cal/Kmol) = 107.696
E(RPBE1PBE) (a.u.) = -476.754128413
Optimised cartesian coordinates (Angstrom):
N 3.696250 -0.256694 -0.748491
C 2.485090 0.135235 -0.115613
C 1.423759 -0.694209 0.107863
N 0.169605 -0.371258 0.638982
C -0.812454 -1.437935 0.803843
C -1.708444 -1.727755 -0.417983
O -2.698763 -0.759406 -0.654621
C -0.078913 0.974201 1.112379
C -0.375129 2.024894 0.018449
N -1.697262 1.824776 -0.595231
H 4.522791 0.096075 -0.248284
H 3.786772 -1.278845 -0.810428
H 2.430151 1.214392 0.101344
H 1.525776 -1.762496 -0.155696
H -1.473485 -1.179885 1.661381
H -0.277383 -2.377223 1.081654
H -2.224813 -2.699822 -0.236032
H -1.036983 -1.893384 -1.304114
H -2.249874 0.137937 -0.739554
H 0.809019 1.329647 1.692935
H -0.929522 0.944727 1.828806
H 0.391409 1.932645 -0.778530
H -0.250940 3.042032 0.465444
H -1.778898 2.362246 -1.468592
H -2.437513 2.182852 0.026182
----
toluene/7-L
Frequencies, energies and thermodynamic properties:
Lowest Vibrational Mode (1/cm) = 33.3488
2nd Lowest Vibrational Mode (1/cm) = 38.1244
E(RB-P86) (a.u.) = -476.760026783
Thermal correction to Enthalpy (a.u.) = 0.228528
Thermal correction to Gibbs Free Energy (a.u.) = 0.175098
Total Entropy (cal/Kmol) = 112.454
E(RPBE1PBE) (a.u.) = -476.755193607

Optimised cartesian coordinates (Angstrom):
N 3.655454 -1.033718 -0.973985
C 3.165806 -1.075862 0.398008
C 2.097175 -0.070589 0.780540
N 1.639332 0.782915 -0.055792
C 0.610274 1.713403 0.377472
C -0.640639 1.594375 -0.522711
N -1.321336 0.300851 -0.503855
C -1.976706 -0.092058 0.749454
C -3.013320 -1.178068 0.415075
O -3.812581 -0.754935 -0.670610
H 3.982135 -0.073617 -1.162379
H 2.844081 -1.129952 -1.603414
H 4.018378 -0.947301 1.107007
H 2.773787 -2.095152 0.629022
H 1.739733 -0.110319 1.845219
H 1.006724 2.748691 0.267744
H 0.333067 1.578338 1.454905
H -0.336974 1.809249 -1.570442
H -1.369873 2.380477 -0.228013
H -0.667781 -0.434823 -0.809494
H -2.506320 0.804119 1.141958
H -1.284357 -0.443711 1.554810
H -3.662857 -1.374996 1.296535
H -2.476749 -2.140382 0.192293
H -3.162038 -0.228670 -1.202480
----
toluene/8-B
Frequencies, energies and thermodynamic properties:
Lowest Vibrational Mode (1/cm) = 40.8938
2nd Lowest Vibrational Mode (1/cm) = 50.7642
E(RB-P86) (a.u.) = -477.973979574
Thermal correction to Enthalpy (a.u.) = 0.252200
Thermal correction to Gibbs Free Energy (a.u.) = 0.200913
Total Entropy (cal/Kmol) = 107.944
E(RPBE1PBE) (a.u.) = -477.973603137
Optimised cartesian coordinates (Angstrom):
N -2.101300 -0.259362 1.513986
C -2.538011 0.253514 0.215583
C -1.701891 -0.393724 -0.899024
N -0.269951 -0.132785 -0.751769
C 0.630810 -1.249535 -1.060236
C 1.160758 -1.932457 0.211190
O 1.994949 -1.097950 0.986172
C 0.169764 1.189730 -1.169138
C 1.394950 1.714413 -0.400542
N 1.211163 1.593916 1.054578
H -2.534119 0.275939 2.278215
H -1.084183 -0.095596 1.580455
H -2.484538 1.371211 0.109674
H -3.606597 -0.009944 0.045667
H -2.096702 -0.069719 -1.899303
H -1.852721 -1.491319 -0.833549
H 1.498698 -0.879456 -1.652980
H 0.118003 -2.006034 -1.702821
H 1.756450 -2.829179 -0.078851
H 0.277491 -2.307629 0.792762
H 1.519969 -0.229718 1.146107
H -0.674058 1.894909 -0.997822
H 0.399051 1.250326 -2.268979
H 1.591052 2.758163 -0.749347
H 2.293007 1.115783 -0.666540
H 0.353439 2.081318 1.352127
H 1.988503 2.050503 1.551237
----
toluene/8-L
Frequencies, energies and thermodynamic properties:
Lowest Vibrational Mode (1/cm) = 46.6408
2nd Lowest Vibrational Mode (1/cm) = 72.7506
E(RB-P86) (a.u.) = -477.978628535
Thermal correction to Enthalpy (a.u.) = 0.252837
Thermal correction to Gibbs Free Energy (a.u.) = 0.202573
Total Entropy (cal/Kmol) = 105.790
E(RPBE1PBE) (a.u.) = -477.976862017
Optimised cartesian coordinates (Angstrom):
C -2.080397 -1.106919 0.639289
C -2.520689 0.161908 -0.120255
H -1.457222 -0.821941 1.524355
H -2.990414 -1.606847 1.052212
H -3.166741 0.791487 0.533682
H -3.159264 -0.166038 -0.972794
O -1.406577 -2.017577 -0.209668
H -0.415563 -1.914634 -0.110316
N -1.424347 0.981344 -0.641875
H -0.718513 0.351607 -1.055129
C -0.742077 1.803910 0.350591
H -0.640474 1.308157 1.354422
H -1.323621 2.735631 0.543847
C 0.655091 2.203213 -0.146450
H 0.542388 2.792433 -1.081595
H 1.128275 2.876899 0.614899
N 1.475521 1.027849 -0.466719
H 2.269365 1.309585 -1.055523
C 1.953176 0.262521 0.685137
H 1.100613 0.128217 1.384179

H 2.759431 0.781442 1.266986
C 2.443344 -1.126020 0.261502
H 3.387643 -1.021508 -0.331275
H 2.708187 -1.706817 1.170710
N 1.386279 -1.836259 -0.474610
H 1.212143 -1.322357 -1.351175
H 1.703530 -2.776899 -0.746598
----
toluene/9
Frequencies, energies and thermodynamic properties:
Lowest Vibrational Mode (1/cm) = 66.6992
2nd Lowest Vibrational Mode (1/cm) = 105.2711
E(RB-P86) (a.u.) = -419.286633653
Thermal correction to Enthalpy (a.u.) = 0.182682
Thermal correction to Gibbs Free Energy (a.u.) = 0.138884
Total Entropy (cal/Kmol) = 92.181
E(RPBE1PBE) (a.u.) = -419.297878143
Optimised cartesian coordinates (Angstrom):
N -2.985368 -0.519659 0.115401
C -2.109917 0.653169 -0.030515
C -0.652394 0.219527 0.242299
O -0.476926 -1.054044 -0.419917
N 0.277154 1.216075 -0.188498
C 1.671823 1.107749 0.254815
C 2.485202 -0.106261 -0.243504
O 2.088416 -1.339250 0.318049
H -3.859153 -0.406324 -0.412226
H -3.253771 -0.661101 1.099060
H -2.367397 1.522329 0.617131
H -2.169463 0.999795 -1.085064
H -0.501694 0.083813 1.343227
H -1.401173 -1.436484 -0.382640
H 0.219442 1.336986 -1.209122
H 1.677719 1.093372 1.368251
H 2.196781 2.040095 -0.054310
H 3.550503 0.057763 0.039150
H 2.456791 -0.117221 -1.366493
H 1.148715 -1.486690 0.031086
----
toluene/H2
Frequencies, energies and thermodynamic properties:
Lowest Vibrational Mode (1/cm) = 4274.0873
2nd Lowest Vibrational Mode (1/cm) =
E(RB-P86) (a.u.) = -1.17248072010
Thermal correction to Enthalpy (a.u.) = 0.013042
Thermal correction to Gibbs Free Energy (a.u.) = -0.001812
Total Entropy (cal/Kmol) = 31.262
E(RPBE1PBE) (a.u.) = -1.16806663656
Optimised cartesian coordinates (Angstrom):
H 0.000000 0.000000 0.383762
H 0.000000 0.000000 -0.383762
----
toluene/H2O
Frequencies, energies and thermodynamic properties:
Lowest Vibrational Mode (1/cm) = 1608.9030
2nd Lowest Vibrational Mode (1/cm) = 3672.8499
E(RB-P86) (a.u.) = -76.3627894706
Thermal correction to Enthalpy (a.u.) = 0.024388
Thermal correction to Gibbs Free Energy (a.u.) = 0.002912
Total Entropy (cal/Kmol) = 45.199
E(RPBE1PBE) (a.u.) = -76.3805270982
Optimised cartesian coordinates (Angstrom):
O 0.000000 0.000000 0.123028
H 0.000000 0.758376 -0.492110
H 0.000000 -0.758376 -0.492110
----

3. References

S1. Peña-López, M., Piehl, P., Elangovan, S., Neumann, H. & Beller, M. Manganese-Catalyzed Hydrogen-Autotransfer C−C Bond Formation: α-Alkylation of Ketones with Primary Alcohols. *Angew. Chem. Int. Ed.* **55**, 14967–14971 (2016).

S2. Shaomin, F., Shao, Z., Wang, Y. & Liu, Q. Manganese-Catalyzed Upgrading of Ethanol into 1-Butanol. *J. Am. Chem. Soc.* **139**, 11941–11948 (2017).

S3. Frisch, M. J.; Trucks, G. W.; Schlegel, H. B.; Scuseria, G. E.; Robb, M. A.; Cheeseman, J. R.; Scalmani, G.; Barone, V.; Petersson, G. A.; Nakatsuji, H.; Li, X.; Caricato, M.; Marenich, A. V.; Bloino, J.; Janesko, B. G.; Gomperts, R.; Mennucci, B.; Hratch, D. J. Guassian 16. at (2016).

S4. Brodie, C. N., Owen, A. E., Kolb, J. S., Bühl, M. & Kumar, A. Synthesis of Polyethyleneimines from the Manganese-Catalysed Coupling of Ethylene Glycol and Ethylenediamine. *Angew. Chem. Int. Ed.* **62**, 29, e202306655 (2023).

S5. Owen, A. E., Preiss, A., McLuskie, A., Gao, C., Peters, G., Bühl, M. & Kumar, A. Manganese-Catalyzed Dehydrogenative Synthesis of Urea Derivatives and Polyureas. *ACS Catal.* **12**, 6923–6933 (2022).

S6. Owen, A. E., Preiss, A., McLuskie, A., Gao, C., Peters, G., Bühl, M. & Kumar, A. Correction to "Manganese Catalyzed Dehydrogenative Synthesis of Urea Derivatives and Polyureas". *ACS Catal.* **13**, 10796–10797 (2023).

S7. Oates, C. L., Goodfellow, A. S., Bühl, M. & Clarke, M. L. Rational Design of a Facially Coordinating P N N Ligand for Manganese-Catalysed Enantioselective Hydrogenation of cyclic ketones. *Angew. Chem. Int. Ed.* **62**, e202212479 (2023).

S8. Oates, C. L., Goodfellow, A. S., Bühl, M. & Clarke, M. L. Manganese catalysed enantioselective hydrogenation of in situ -synthesised imines: efficient asymmetric synthesis of amino-indane derivatives. *Green Chem.* **25**, 3864–3868 (2023).

S9. Goodfellow, A. S. & Bühl, M. Hydricity of 3d transition metal complexes from density functional theory: A benchmarking study. *Molecules* **26**, 4072 (2021).

S10. Martin, R. L., Hay, P. J. & Pratt, L. R. Hydrolysis of ferric ion in water and conformational equilibrium. *J. Phys. Chem. A* **102**, 3565–3573 (1998).

S11. Pracht, P., Bohle, F. & Grimme, S. Automated exploration of the low-energy chemical space with fast quantum chemical methods. *Phys. Chem. Chem. Phys.* **22**, 7169–7192 (2020).

S12. Grimme, S. Exploration of Chemical Compound, Conformer, and Reaction Space with Meta-Dynamics Simulations Based on Tight-Binding Quantum Chemical Calculations. *J. Chem. Theory Comput.* **15**, 2847–2862 (2019).

S13. Iribarren, I. & Trujillo, C. Efficiency and Suitability when Exploring the Conformational Space of Phase-Transfer Catalysts. *J. Chem. Inf. Model.* **62**, 5568–5580 (2022).
